# Attributable burden of high BMI-related gastrointestinal tract cancers among middle-aged and elderly populations globally, 1990–2021 and projected to 2050: analysis of GBD 2021

**DOI:** 10.3389/fnut.2025.1674621

**Published:** 2026-01-02

**Authors:** Yierfan Yilihaer, Alimu Tulahong, Rexiati Ruzi, Yanze Lin, Zhongdian Yuan, Ainiwaer Aikebaier, Tiemin Jiang, Yingmei Shao, Tuerganaili Aji

**Affiliations:** 1 Department of Hepatobiliary and Echinococcosis Surgery, Digestive and Vascular Surgery Center, First Affiliated Hospital of Xinjiang Medical University, Ürümqi, China; 2 State Key Laboratory of Pathogenesis, Prevention and Treatment of High Incidence Diseases in Central Asia, Xinjiang Medical University, Ürümqi, China

**Keywords:** global burden, high BMI, health inequalities, mortality, gastrointestinal tract cancers

## Abstract

**Background:**

The global burden of gastrointestinal tract cancers (GICs)—specifically liver cancer (LC), colorectal cancer (CRC), gallbladder and biliary tract cancers (GBTCs), and pancreatic cancer (PC)—attributable to high body mass index (BMI) is of increasing concern, especially among middle-aged and elderly populations.

**Methods:**

Using data from the Global Burden of Disease (GBD) Study 1990–2021, we assessed deaths, disability-adjusted life years (DALYs), age-standardized mortality rates (ASMR), and estimated annual percentage changes (EAPC) for LC, GBTCs, CRC, and PC attributable to high BMI in middle-aged and elderly populations. We analyzed regional, national, and Socio-demographic Index (SDI)-stratified patterns and their association with SDI and Human Development Index (HDI). Forecasts to 2050 were based on historical trends in these age groups.

**Results:**

From 1990 to 2021, LC deaths increased by 363% to 40,958, with ASMR rising from 1.0 to 2.2 per 100,000 (EAPC: 2.4%). GBTC deaths rose by 109% to 19,035, but ASMR slightly declined from 1.1 to 1.0 (EAPC: –0.5%). CRC deaths increased by 140% to 92,013, with stable ASMR (4.9 to 5.0; EAPC: −0.1%). Pancreatic cancer mortality also increased globally (EAPC: 4.4%), with high-SDI regions bearing the highest burden and middle-SDI regions experiencing the fastest growth (EAPC: 18.1%). East Asia bore the highest burden for all cancers, while South Asia showed the fastest mortality increases (EAPC: 5.4% for LC, 2.9% for CRC). At the national level, China and India contributed the largest share of deaths, while Indonesia and Vietnam showed the fastest growth in mortality. High-SDI regions had the largest absolute burden, but middle-SDI regions exhibited the fastest growth. All cancers showed male predominance, peaking at ages 60–79 years (LC: 60–64; GBTCs: 65–69; CRC: 75–79). ASMR followed an inverted U-shaped curve with SDI and HDI, peaking at SDI 0.7–0.9. By 2050, CRC and LC are projected to see rising DALYs and Years Lived with Disability (YLDs) in middle-aged and elderly populations, while GBTCs remain stable, with declining Years of Life Lost (YLLs) rates across all cancers.

**Conclusion:**

The escalating burden of high BMI-related GICs—particularly LC, CRC, GBTCs, and PC—in aging populations, especially in developing regions, highlights the urgent need for targeted interventions in prevention, early detection, and survivorship care.

## 1 Introduction

Digestive tract malignancies—specifically liver cancer (LC), colorectal cancer (CRC), and gallbladder and biliary tract cancers (GBTCs),pancreatic cancer (PC)—continue to pose a significant threat to global public health, marked by their high incidence and considerable mortality risk (1, 2). According to data released by the World Health Organization (WHO) in 2022, global cancer deaths reached approximately 9.7 million, with digestive tract cancers accounting for a substantial proportion of the overall cancer spectrum (3). The pathogenesis of these malignancies is complex, influenced by a multifaceted interplay of genetic predisposition, environmental factors, and lifestyle choices (4–6). Among metabolic risk factors, elevated body mass index (BMI ≥ 25 kg/m^2^) has been widely recognized as one of the most amenable to intervention (7–9). Extensive epidemiological and molecular biology research indicates that high BMI significantly increases the risk of developing liver cancer, CRC, and GBTCs through multiple pathways, including the acceleration of non-alcoholic fatty liver disease (NAFLD), induction of insulin resistance, promotion of chronic inflammation, and disruption of gut microbiota (10–12).

The Global Burden of Disease (GBD) study has further quantified this risk. In 2021, an estimated 356,700 (95% uncertainty interval [UI]: 146,100–581,000) cancer deaths globally were attributable to high BMI (13). The age-standardized population attributable fraction (PAF) was 3.59% (1.45–5.80%), underscoring the severe global health challenge posed by the obesity epidemic and reflecting its escalating prevalence (14, 15).

Concurrently, the WHO 2022 report revealed that the prevalence of overweight and obesity among adults globally has risen to 43 and 16%, respectively (16). Driven by rapid urbanization and profound shifts in lifestyle, obesity rates are surging in many emerging economies, thereby indirectly exacerbating the burden of chronic diseases like cancer, driving up healthcare expenditure, and diminishing labor supply (17). In this context, the socioeconomic impact of cancer and related chronic conditions is becoming increasingly pronounced, encompassing reduced productivity, increased absenteeism, and heightened family caregiving burdens, with growing attention drawn to issues of health equity (18, 19).

Considering the global epidemiological transition and accelerating population aging, digestive tract malignancies present an even more challenging burden, particularly within the elderly population (20). Elderly males (65–74 years) constitute a high-risk group for LC, CRC, GBTCs and PC. Furthermore, the growth trajectories and intervention capacities for cancer burden exhibit significant heterogeneity across different countries and regions, influenced by variations in their Socio-demographic Index (SDI), obesity prevalence, and healthcare resource distribution (13).

This study, utilizing the GBD 2021 database, focuses on LC, CRC, GBTCs, and PC attributable to high BMI. It aims to systematically characterize the global and regional disease burden trends from 1990 to 2021, conduct heterogeneity analyses concerning sex, age, geographical location, and socioeconomic factors, and project the burden trajectory until 2050. The ultimate goal is to provide a robust scientific foundation for the development of policies aimed at obesity prevention, cancer screening, and healthcare resource optimization, thereby addressing the escalating challenge of digestive tract malignancies.

## 2 Methods

### 2.1 Data source

For this investigation, we extracted data from the Global Burden of Disease 2021 (GBD 2021) repository via the Global Health Data Exchange query tool^1^ (21), which systematically quantifies global, regional, and national disease burdens attributable to metabolic risks including high body mass index; specifically selecting “High Body Mass Index” in the Risk factor list and three gastrointestinal cancers in the Cause list—gallbladder/biliary tract cancer (GBTC; ICD-10 C23-C24), liver cancer (LC; ICD-10 C22), colorectal cancer (CRC; ICD-10 C18-C20),and pancreatic cancer (PC; ICD-10 C25) —with age filtering for ≥50 years populations during 1990–2021, collecting absolute numbers and age-standardized rates (ASR per 100,000) of deaths and disability-adjusted life years (DALYs) attributable to high BMI across 204 countries and 21 GBD regions, supplemented by socio-demographic index (SDI) and human development index (HDI) metrics for development-level stratification (22, 23).

In GBD 2021, High BMI was defined as a body mass index above 20–23 kg/m^2^ for adults (aged ≥20 years). The cancer burden attributable to high BMI was estimated using multiple global data sources, including literature reviews, hospital records, population surveys, and cause-of-death reports (22).

### 2.2 SDI and DALYs

The Socio-Demographic Index (SDI) is a composite measure of a country’s development level, calculated as the geometric mean of educational attainment (ages 15+), fertility rate (under 25), and lag-distributed income per capita, with scores ranging from 0 (lowest health-related development) to 1 (highest). In GBD 2021, countries are grouped into five SDI categories: low, low-middle, middle, high-middle, and high SDI. Disability-Adjusted Life Years (DALYs) quantify disease burden by summing years of life lost due to premature mortality and years lived with disability, providing a standard metric for assessing population health outcomes (21).

### 2.3 Statistical analysis

To assess trends in mortality, and Disability-Adjusted Life Years (DALYs) for gallbladder and biliary tract cancer, liver cancer, and colorectal cancer from 1990 to 2021, we calculated percentage changes to quantify variations in these metrics over the study period. The formula for this calculation is as follows:


$$\mathrm{Percentage}=\frac{\mathrm{case}\in 2021-\mathrm{case}\in 1990}{\mathrm{case}\in 1990}$$

We used the age-standardized rate (ASR) and estimated annual percentage change (EAPC) to quantify temporal trends in deaths and DALYs rates. ASR per 100,000 population was calculated using the following formula:


$$\mathrm{Age}\;\mathrm{standardized}\;\;\mathrm{rate}=\frac{\overset{A}{\underset{i=1}{\sum }}a_{i}w_{i}}{\overset{A}{\underset{i=1}{\sum }}w_{i}}$$

(αi: the age-specific rate in $\;\;i^{th}$ age group; w : the number of people in the corresponding $\;\;i^{th}\;$ age group among the standard population; *A*: the number of age groups.)

EAPC is the common metric in epidemiological research, used to determine temporal changes in ASR. The EAPC calculation is based on a linear regression model fitted to the natural logarithm of the ASR, with time as the independent variable (24). By fitting a straight line to the natural log of each observed ASR over the study period, the slope of this line provides the EAPC. This method offers a straightforward way to quantify trends, with positive EAPC values indicating an increasing trend and negative values signaling a decrease in ASR over time. The formula for this calculation is as follows:


Y=α+βX+ε

$$EAPC=100*\left(\exp\left(\beta \right)-1\right)$$

(X: year, Y: the natural logarithm of rates (such as incidence rate), α: the intercept, β: the slope, ε: the random error. The 95% confidence intervals of the EAPC are also derived from this fitted model.)

We conducted Pearson correlation analysis to explore associations between SDI, HDI, and both ASRs/EAPCs across 204 countries. Future burden projections (2022–2050) were generated using autoregressive integrated moving average (ARIMA) models, with optimal (p,d,q) orders determined by Akaike Information Criterion minimization and model residuals validated through Ljung-Box testing (*p* > 0.05). GBD generated the 95% UIs for each measure using advanced Bayesian statistical methods. The posterior distributions were obtained by synthesizing data sources and accounting for measurement uncertainties, with UIs computed from 1,000 posterior draws (2.5th-97.5th percentiles). Changes were significant when 95% UIs excluded zero.

All statistical analyses were conducted using R software (version 4.3.2).

## 3 Results

### 3.1 Global burden of high BMI-related gastrointestinal tract cancers, 1990–2021

The global burden of LC increased significantly from 1990 to 2021. LC deaths rose from 8,853.5 (95% UI: 3,568.3–14,600.1) in 1990 to 40,958 (95% UI: 16,553.4–68,880.6) in 2021, a 363% increase. The ASMR for LC increased from 1.0 (95% UI: 0.4–1.7) to 2.2 (95% UI: 0.9–3.6) per 100,000 population, with an EAPC of 2.4% (95% CI: 2.3–2.5). DALYs for LC rose by 341%, from 223,275.7 (95% UI: 90,024.7–368,412.1) in 1990 to 984,496.2 (95% UI: 400,302.3–1,663,344.8) in 2021, with the age-standardized DALY rate increasing from 25.2 to 51.0 per 100,000 population (Table 1 and Supplementary Table 1).

**Table 1:** High BMI-attributable gastrointestinal cancer mortality: numbers, rates, and trends, 1990–2021.

Region, SDI, and Sex		**1990 death cases**	**1990 ASMR**	**2021 death cases**	**2021 ASMR**	**EAPC (1990–2021)**	**Cancer type**
Global		38385 (16174.6, 62103.4)	4.9 (2.1, 8)	92012.9 (39255.8, 147266.7)	5 (2.1, 8)	–0.1 (–0.1,–0.0)	Colon and rectum cancer
		9104.2 (6231.2, 12425.2)	1.1 (0.8, 1.6)	19035.2 (12758.2, 26584.4)	1 (0.7, 1.4)	–0.5 (–0.5,–0.4)	Gallbladder and biliary tract cancer
		984.1 (–1865.8, 5660.2)	0.1 (–0.2, 0.7)	8753.7 (–2193.6, 25213)	0.5 (–0.1, 1.3)	4.4 (4.2, 4.6)	Pancreatic cancer
		8853.5 (3568.3, 14600.1)	1 (0.4, 1.7)	40958 (16553.4, 68880.6)	2.2 (0.9, 3.6)	2.4 (2.3, 2.5)	Liver cancer
By sex	Female	21068.3 (8884.8, 34133.8)	4.8 (2, 7.8)	45133 (19229.5, 72025)	4.4 (1.9, 7)	–0.4 (–0.5, –0.4)	Colon and rectum cancer
		6287.2 (4253.4, 8612.1)	1.4 (0.9, 1.9)	11544.3 (7523.6, 16414)	1.1 (0.7, 1.6)	–0.9 (–0.9, –0.8)	Gallbladder and biliary tract cancer
		954.5 (–721, 3553)	0.2 (–0.2, 0.8)	5354.7 (–709.8, 14007.5)	0.5 (–0.1, 1.4)	3.0 (2.9, 3.0)	Pancreatic cancer
		3924.3 (1529.3, 6472.2)	0.9 (0.3, 1.4)	16284.5 (6630, 27310.5)	1.6 (0.7, 2.7)	2.1 (2.0, 2.1)	Liver cancer
	Male	17316.7 (7164.9, 28143.9)	5 (2.1, 8.2)	46879.8 (20091.9, 74977.4)	5.7 (2.4, 9.1)	0.3 (0.3, 0.4)	Colon and rectum cancer
		2817 (1944.4, 3879.1)	0.8 (0.6, 1.1)	7490.9 (4841, 10654.1)	0.9 (0.6, 1.3)	0.3 (0.3, 0.4)	Gallbladder and biliary tract cancer
		29.6 (–1219.4, 2155.8)	0 (–0.3, 0.6)	3399.1 (–1577, 11169.5)	0.4 (–0.2, 1.3)	10.3 (8.7, 12.0)	Pancreatic cancer
		4929.2 (2044.1, 8193.4)	1.2 (0.5, 2.1)	24673.5 (10062.2, 42132.6)	2.8 (1.1, 4.8)	2.6 (2.5, 2.7)	Liver cancer
High SDI		20794.1 (8752.9, 33711.1)	8.5 (3.6, 13.8)	34989.2 (14842.1, 56009.5)	7.1 (3, 11.3)	–0.7 (–0.8, –0.7)	Colon and rectum cancer
		4154 (2810.7, 5737.5)	1.7 (1.1, 2.3)	6155.3 (3996.1, 8679)	1.2 (0.8, 1.7)	–1.2 (–1.3, –1.1)	Gallbladder and biliary tract cancer
		832 (–796.7, 3489.4)	0.3 (–0.3, 1.4)	4385.3 (–793.2, 11922.7)	1 (–0.1, 2.5)	3.3 (3.2, 3.5)	Pancreatic cancer
		3134.7 (1294.3, 5335)	1.3 (0.5, 2.2)	13492.8 (5528.9, 22396.5)	2.9 (1.2, 4.8)	2.6 (2.4, 2.8)	Liver cancer
High–middle SDI		12640.2 (5374.8, 20405.3)	6.1 (2.6, 9.9)	31036.7 (13310.4, 50090.6)	7.1 (3, 11.5)	0.4 (0.3, 0.5)	Colon and rectum cancer
		2869.7 (1953.7, 3948)	1.4 (0.9, 1.9)	5348.5 (3521.6, 7622.4)	1.2 (0.8, 1.7)	–0.6 (–0.7, –0.5)	Gallbladder and biliary tract cancer
		419.5 (–571.7, 2046.4)	0.2 (–0.3, 0.9)	2916.3 (–629.5, 8481.7)	0.7 (–0.1, 1.9)	4.0 (3.9, 4.1)	Pancreatic cancer
		2719.9 (1097, 4591.2)	1.2 (0.5, 2.1)	10074.4 (3958.5, 17746)	2.3 (0.9, 4)	1.9 (1.9, 2.0)	Liver cancer
Low SDI		374.6 (134.4, 629.5)	0.8 (0.3, 1.4)	1290.7 (495.4, 2093.1)	1.3 (0.5, 2.1)	1.5 (1.4, 1.6)	Colon and rectum cancer
		94.2 (58.3, 145.5)	0.2 (0.1, 0.3)	343.2 (207.4, 495.7)	0.3 (0.2, 0.5)	1.8 (1.7, 1.9)	Gallbladder and biliary tract cancer
		–25 (–51.8, 4.2)	–0.1 (–0.1, 0)	–3.6 (–78.9, 112.8)	0 (–0.1, 0.1)	NA (NA,NA)	Pancreatic cancer
		342.8 (125.7, 620.2)	0.7 (0.3, 1.3)	1200.6 (444.7, 2072.3)	1.1 (0.4, 1.9)	1.3 (1.2, 1.4)	Liver cancer
Low–middle SDI		1016.4 (382.8, 1645.5)	0.8 (0.3, 1.4)	5421.9 (2255.8, 8636)	1.8 (0.8, 2.9)	2.8 (2.7, 2.8)	Colon and rectum cancer
		518.7 (351.2, 743.8)	0.4 (0.3, 0.6)	2164.7 (1414.8, 3013.9)	0.7 (0.5, 1)	1.9 (1.8, 1.9)	Gallbladder and biliary tract cancer
		–41 (–104.1, 55.8)	0 (–0.1, 0)	406.2 (–122.7, 1249.9)	0.1 (0, 0.4)	14.1 (11.3, 17.0)	Pancreatic cancer
		1081 (405.2, 1999.7)	0.8 (0.3, 1.6)	5458.2 (2236.4, 9174.8)	1.8 (0.7, 2.9)	2.6 (2.5, 2.7)	Liver cancer
Middle SDI		3485.2 (1295.1, 5706.2)	1.7 (0.6, 2.8)	19122.6 (8142.5, 30802.8)	3.4 (1.4, 5.4)	2.2 (2.1, 2.2)	Colon and rectum cancer
		1447.7 (988.9, 2019.3)	0.7 (0.5, 1)	5001.4 (3316.7, 6971.1)	0.9 (0.6, 1.2)	0.5 (0.5, 0.6)	Gallbladder and biliary tract cancer
		–206 (–469.8, 219.4)	–0.1 (–0.2, 0.1)	1033.9 (–644.1, 3657.1)	0.2 (–0.1, 0.6)	18.1 (15.1, 21.1)	Pancreatic cancer
		1560.8 (641.1, 2543.6)	0.7 (0.3, 1.2)	10697.7 (4318.6, 18265)	1.8 (0.7, 3.1)	3.1 (3.0, 3.2)	Liver cancer

In contrast, GBTCs showed a more moderate burden increase, with deaths rising from 9,104.2 (95% UI: 6,231.2–12,425.2) in 1990 to 19,035.2 (95% UI: 12,758.2–26,584.4) in 2021, a 109% increase (Table 1). However, the ASMR for GBTCs slightly declined from 1.1 (95% UI: 0.8–1.6) to 1.0 (95% UI: 0.7–1.4) per 100,000 population, with an EAPC of −0.5% (95% CI: −0.5 to −0.4). Global DALYs for GBTCs doubled from 200,194.9 (95% UI: 137,599.4–272,531.9) in 1990 to 400,865.1 (95% UI: 270,792.7–556,609.9) in 2021, but the age-standardized DALY rate decreased from 23.6 to 21.1 per 100,000 population, with an EAPC of −0.5% (95% CI: −0.6 to −0.4) (Supplementary Table 2).

**Figure 1:**
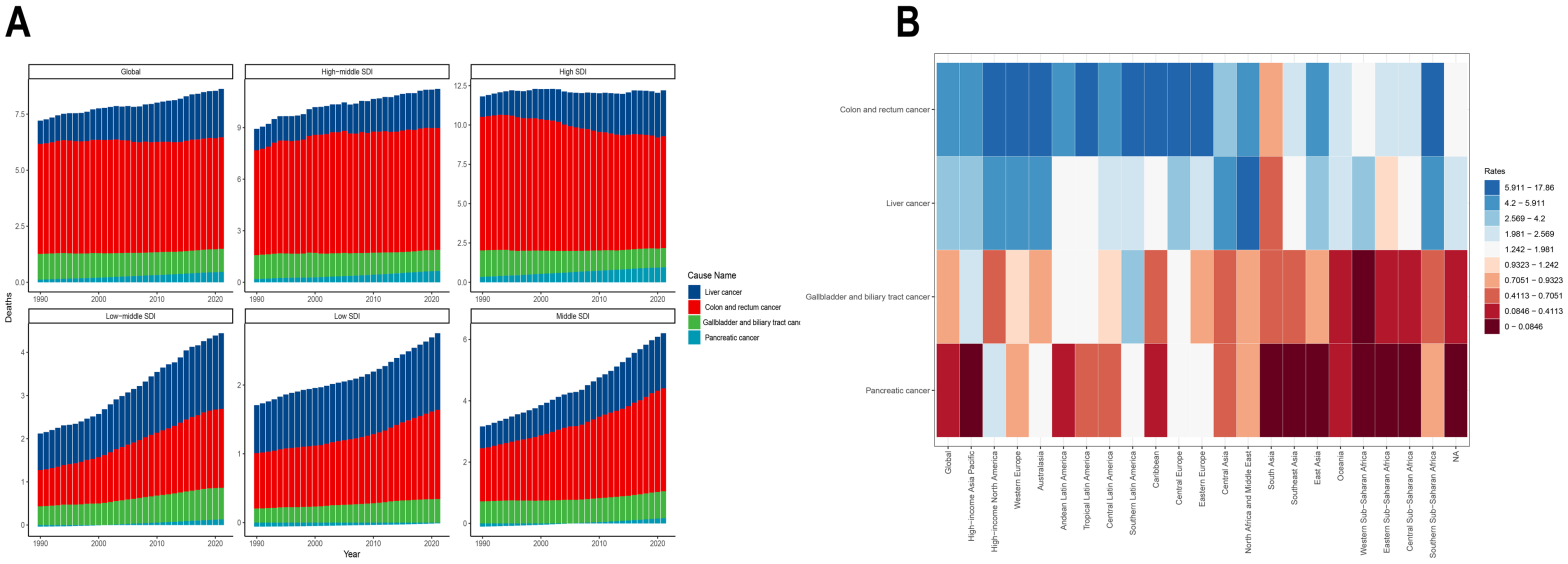
Trends and distribution of high BMI-attributable gastrointestinal cancer burden by SDI and GBD region, 1990–2021. **(A)** Annual number of deaths attributable to high body mass index (BMI) for colon and rectum cancer (red), gallbladder and biliary tract cancer (blue), and liver cancer (green) from 1990 to 2021, stratified by Global and the five SDI regions (High SDI, High-middle SDI, Middle SDI, Low-middle SDI, and Low SDI). **(B)** Heatmap showing high BMI-attributable cancer death rates in 2021 for colon and rectum cancer, gallbladder and biliary tract cancer, and liver cancer across 21 Global Burden of Disease (GBD) regions. Colors indicate the magnitude of death rates, with darker colors representing higher rates.

CRC exhibited a significant global burden increase, with deaths rising from 38,385 (95% UI: 16,174.6–62,103.4) in 1990 to 92,012.9 (95% UI: 39,255.8–147,266.7) in 2021, a 140% increase (Supplementary Table 3). The ASMR for CRC remained stable, increasing marginally from 4.9 (95% UI: 2.1–8.0) to 5.0 (95% UI: 2.1–8.0) per 100,000 population, with an EAPC of −0.1% (95% CI: −0.1 to 0.0). Global DALYs for CRC increased substantially, particularly in high-burden regions, as detailed below (Figure 1).

Similarly, the global burden of pancreatic cancer (PC) attributable to high BMI increased substantially from 1990 to 2021. Global PC deaths rose from 984.1 in 1990 to 8753.7 in 2021, with the age-standardized mortality rate increasing from 1.0 to 0.5 per 100,000 population, corresponding to an EAPC of 4.4% (95% CI: 4.2–4.6). Middle-SDI regions experienced the fastest mortality growth (EAPC: 18.1%), followed by low-middle-SDI regions (EAPC: 14.1%), while high-SDI regions bore the largest absolute mortality burden in 2021. DALYs and YLDs for PC also increased markedly across all SDI groups (Table 1 and Supplementary Table 4).

To minimize the influence of early-period fluctuations, additional analyses for 2000–2021 and 2010–2021 were conducted, and the consistent EAPC trends further support the reliability of our conclusions (Supplementary Tables 1–3).

### 3.2 Regional burden of high BMI-related gastrointestinal tract cancers

**Figure 2:**
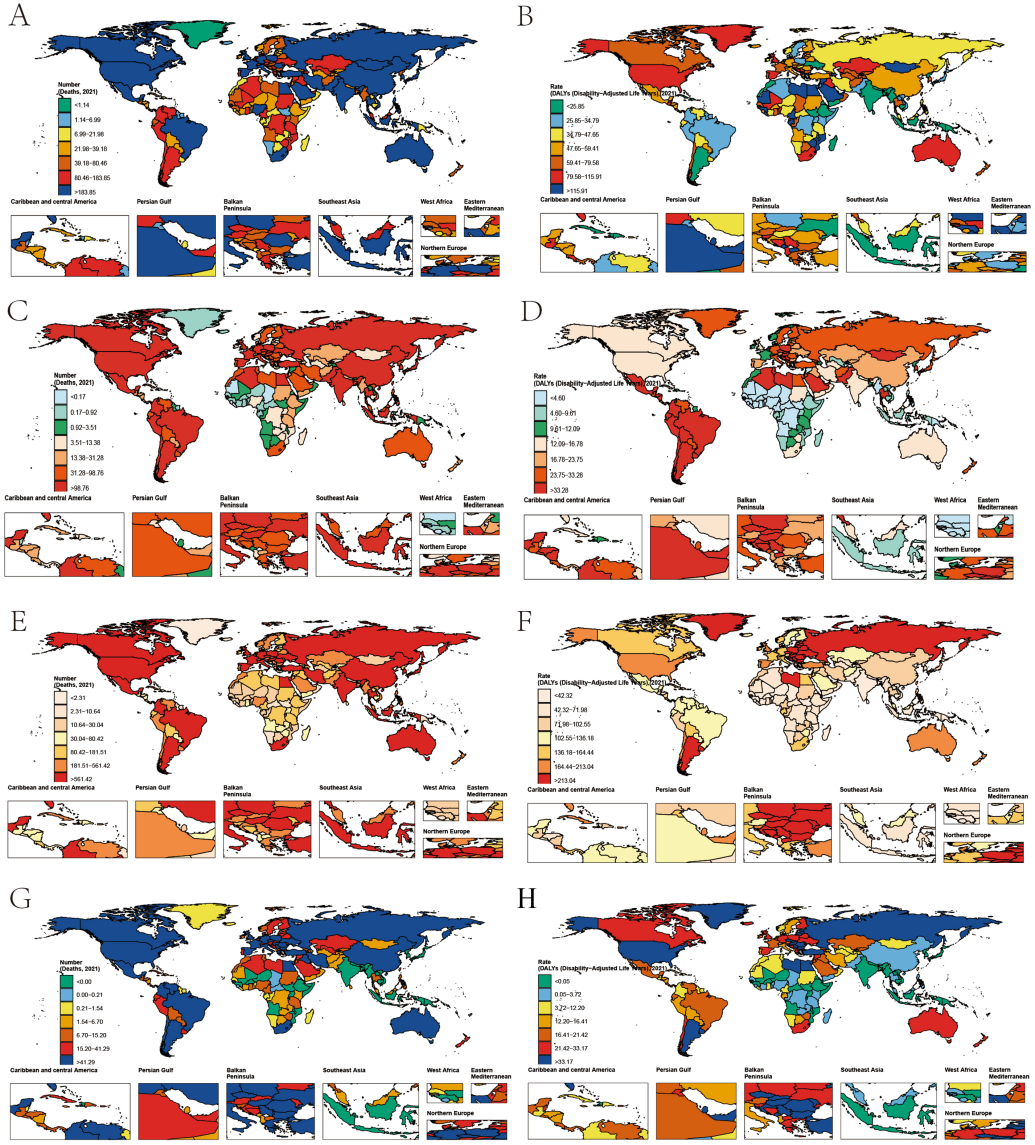
Age-standardized rates of gastrointestinal tract cancer deaths and DALYs attributable to high BMI in 2021 at the country level. **(A)** Age-standardized death rates attributable to high BMI for liver cancer in 2021, by country. **(B)** Age-standardized DALY rates attributable to high BMI for liver cancer in 2021, by country. **(C)** Age-standardized death rates attributable to high BMI for gallbladder and biliary tract cancer in 2021, by country. **(D)** Age-standardized DALY rates attributable to high BMI for gallbladder and biliary tract cancer in 2021, by country. **(E)** Age-standardized death rates attributable to high BMI for colon and rectum cancer in 2021, by country. **(F)** Age-standardized DALY rates attributable to high BMI for colon and rectum cancer in 2021, by country. **(G)** Age-standardized death rates attributable to high BMI for pancreatic cancer in 2021, by country. **(H)** Age-standardized DALY rates attributable to high BMI for pancreatic cancer in 2021, by country. DALYs, disability-adjusted life years; BMI, body mass index.

Across geographic regions, East Asia bore the highest burden of liver cancer mortality in 2021, with 10,838.7 deaths (95% UI: 4,134.9–19,316.8), followed by Western Europe (6,059.2; 95% UI: 2,373.1–1,652.6), High-Income North America (5,517.3; 95% UI: 2,347.1–8,972), and North Africa and Middle East (4,577.7; 95% UI: 1,905.1–7,746.7) (Figure 2). South Asia exhibited the most rapid increase in liver cancer mortality, with an EAPC of 5.4% (95% CI: 5.3–5.5), followed by Southeast Asia (3.1%; 95% CI: 2.9–3.3) and Tropical Latin America (2.8%; 95% CI: 2.6–3.1). In contrast, High-Income Asia Pacific showed a decline in mortality, with an EAPC of −0.3% (95% CI: −0.7 to 0.2). When stratified by socio-demographic index (SDI), high-SDI regions accounted for the largest absolute burden, with 13,492.8 deaths (95% UI: 5,528.9–22,396.5), yet middle-SDI regions displayed the fastest growth (EAPC: 3.1%; 95% CI: 3.0–3.2), followed by low-middle-SDI regions (EAPC: 2.6%; 95% CI: 2.5–2.7) (Supplementary Table 1 and Supplementary Figure 1).

The burden of gallbladder and biliary tract cancer showed distinct regional and national patterns. At the national level, China reported the highest mortality in 2021, with 3,827.7 deaths (95% UI: 2,169.6–5,737.7), followed by Japan (1,742.7; 95% UI: 1,064.3–2,490.3) and India (1,637.2; 95% UI: 954.8–2,397.7) (Figure 2). Low-SDI and low-middle-SDI regions experienced the most significant mortality increases, with EAPCs of 1.8% (95% CI: 1.7–1.9) and 1.9% (95% CI: 1.8–1.9), respectively, while high-SDI regions exhibited a notable decline (EAPC: −1.2%; 95% CI: −1.3 to −1.1). Despite this, high-SDI regions carried the largest absolute burden, with 6,155.3 deaths (95% UI: 3,996.1–8,679) and an ASMR of 1.2 (95% UI: 0.8–1.7) per 100,000 population, reflecting slower growth compared to lower-SDI regions (Supplementary Table 2 and Supplementary Figure 1).

For colorectal cancer, East Asia again reported the highest mortality in 2021, with 18,405.5 deaths (95% UI: 7,530.2–30,739.3), followed by Western Europe (17,613.6; 95% UI: 7,367.8–29,042.1) and High-Income North America (12,047; 95% UI: 5,329.7–18,866.2). South Asia and Southeast Asia showed the most rapid increases in mortality, with EAPCs of 2.9% (95% CI: 2.8–2.9) and 3.2% (95% CI: 3.1–3.3), respectively, whereas High-Income North America and Australasia experienced declines (EAPC: −0.8%; 95% CI: −0.9 to −0.7 and −0.8%; 95% CI: −0.9 to −0.8, respectively). At the national level, China bore the highest burden, with 17,515.7 deaths (95% UI: 7,157.9–29,363.8) and an EAPC of 2.5% (95% CI: 2.4–2.5), followed by the United States (10,734.1; 95% UI: 4,768.7–16,815.2). In China, colorectal cancer DALYs increased by 445%, from 75,537 (95% UI: 25,927.9–128,893.1) in 1990 to 411,982.4 (95% UI: 169,014.4–692,218.2) in 2021, with the age-standardized DALY rate rising from 40.8 to 84.6 per 100,000 population (Figure 2).

For pancreatic cancer (PC), high-SDI regions had the largest mortality burden in 2021, reporting 4,383.5 deaths, whereas middle-SDI regions exhibited the fastest increase in mortality, with an EAPC of 18.1%. Low-middle-SDI regions also showed substantial growth (EAPC: 14.1%), while high-middle-SDI and high-SDI regions demonstrated moderate but steady upward trends (EAPC: 4.0 and 3.3%, respectively). The low-SDI region showed minimal burden throughout the study period (Supplementary Table 4).

### 3.3 Age and sex pattern of high BMI attributable gastrointestinal tract cancer burden

**Figure 3:**
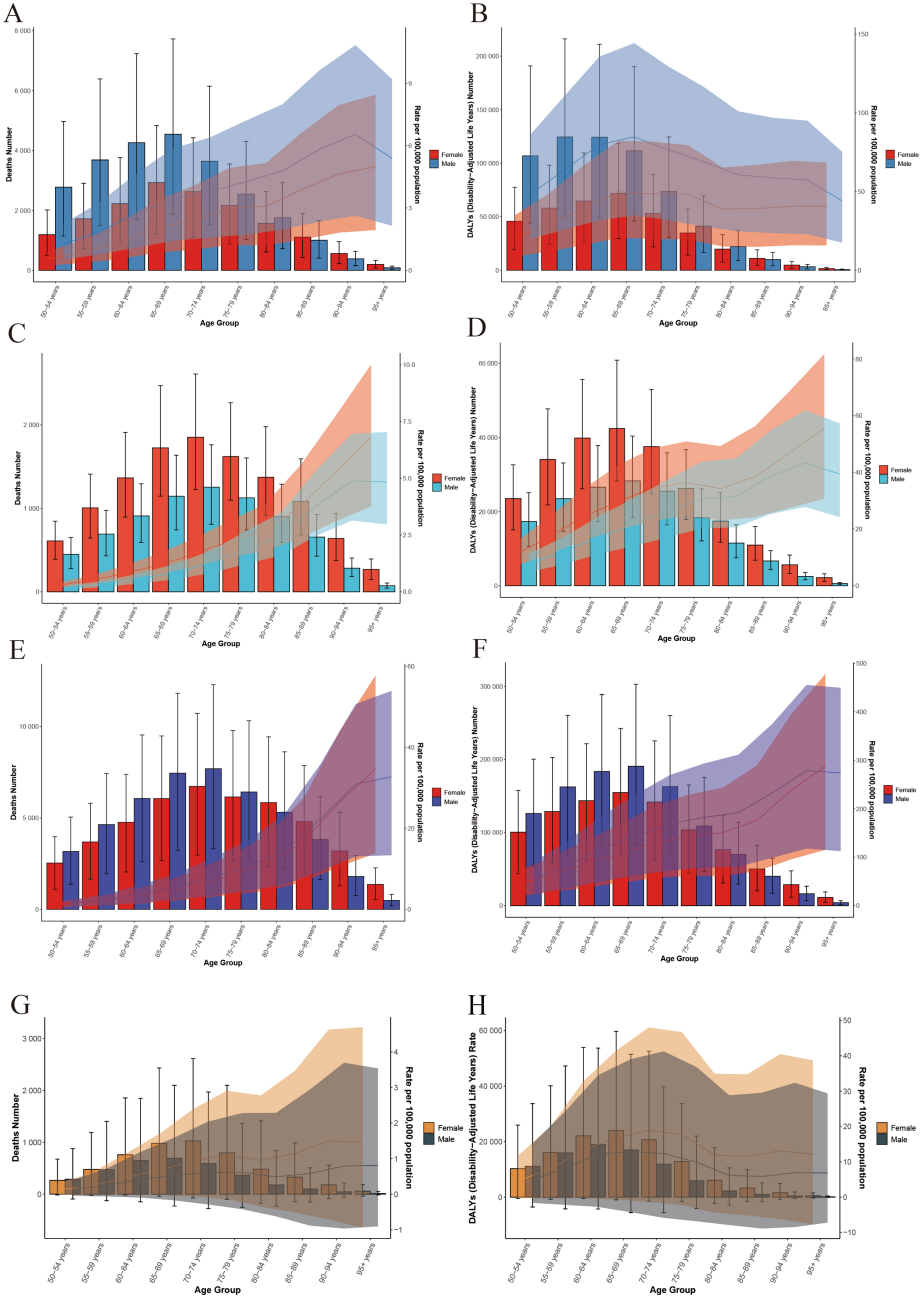
Age-specific deaths and DALYs attributable to high BMI for gastrointestinal tract cancer in 2021. **(A)** Age-specific death counts attributable to high body mass index (BMI) for liver cancer, stratified by sex (red for females, blue for males) across different age groups. **(B)** Age-specific DALY counts attributable to high BMI for liver cancer, stratified by sex across different age groups. **(C)** Age-specific death counts attributable to high BMI for gallbladder and biliary tract cancer, stratified by sex across different age groups. **(D)** Age-specific DALY counts attributable to high BMI for gallbladder and biliary tract cancer, stratified by sex across different age groups. **(E)** Age-specific death counts attributable to high BMI for colorectal cancer, stratified by sex across different age groups. **(F)** Age-specific DALY counts attributable to high BMI for colorectal cancer, stratified by sex across different age groups. **(G)** Age-specific death counts attributable to high BMI for Pancreatic cancer, stratified by sex across different age groups. **(H)** Age-specific DALY counts attributable to high BMI for Pancreatic cancer, stratified by sex across different age groups. DALYs, disability-adjusted life years; BMI, body mass index.

**Figure 4:**
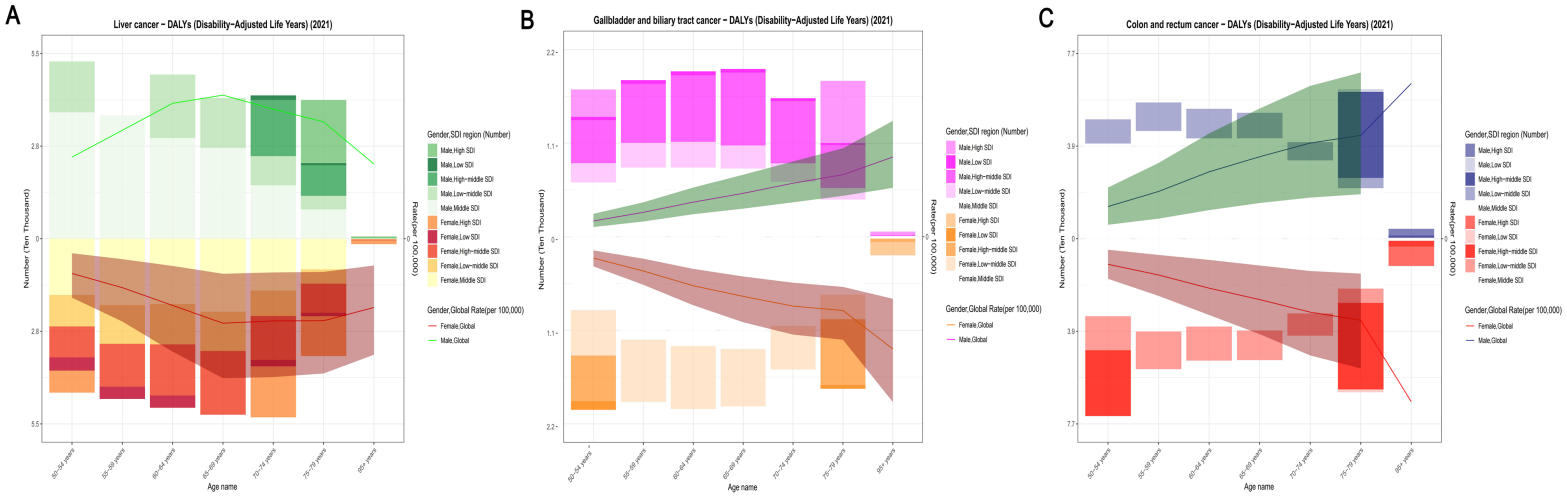
Burden of gastrointestinal tract cancer in 2021 across global and five SDI regions, stratified by age and sex. **(A)** Age-specific DALY rates attributable to high body mass index (BMI) for liver cancer in 2021, stratified by sex (green for females, red for males) across different age groups. The line represents the overall trend across age groups. **(B)** Age-specific DALY rates attributable to high BMI for gallbladder and biliary tract cancer in 2021, stratified by sex across different age groups. **(C)** Age-specific DALY rates attributable to high BMI for colorectal cancer in 2021, stratified by sex across different age groups. DALYs, disability-adjusted life years; BMI, body mass index.

The epidemiological distribution of high BMI-attributable liver, gallbladder and biliary tract, and The epidemiological distribution of high BMI-attributable liver, gallbladder and biliary tract, colorectal, and pancreatic cancer burdens reveals both shared patterns and distinct characteristics. All four cancers exhibit a male-dominated disease burden, with the largest sex disparities peaking in the 65–74-year age window. Specifically, liver cancer mortality peaks in males at 60–64 years, gallbladder and biliary tract cancer health losses are most pronounced at 65–69 years, colorectal cancer reaches its highest burden across all ages at 75–79 years, and pancreatic cancer follows a similar trend, with a peak burden in males aged 70–74 years, reflecting the increasing risk among elderly males in all regions. Regionally, liver and gallbladder cancers follow a high burden in low socio-demographic index (SDI) regions, whereas colorectal and pancreatic cancers display a unique high burden in high SDI regions, particularly evident in North America and Western Europe. Common features include a continuous rise in burden after age 50, with the largest sex disparity at 70–74 years (gallbladder and biliary tract cancer > liver cancer > colorectal cancer > pancreatic cancer), and males aged ≥65 years forming a shared high-risk group. These patterns inform targeted interventions: liver and gallbladder cancers require focus on older males in low-SDI regions, while colorectal and pancreatic cancers demand priority in high-SDI regions among elderly males (Figures 3, 4).

### 3.4 Gastrointestinal tract cancer burden attributable to high BMI was associated with SDI and HDI

**Figure 5:**
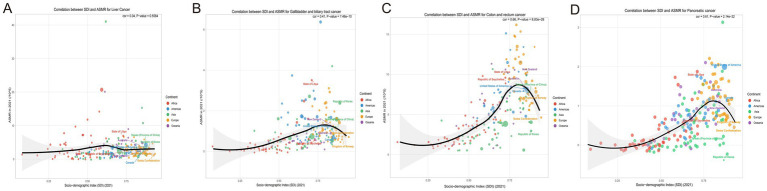
Correlation between socio-demographic index (SDI) and age-standardized mortality rates (ASMR) and for liver cancer, colorectal cancer, and gallbladder and biliary tract cancer in 2021. **(A)** Correlation between SDI and age-standardized mortality rates (ASMR) for liver cancer, categorized by continent. **(B)** Correlation between SDI and ASMR for colorectal cancer, with data points differentiated by continent. **(C)** Correlation between SDI and ASMR for gallbladder and biliary tract cancer, highlighting continent-specific data points. **(D)** Correlation between SDI and ASMR for pancreatic cancer, highlighting continent-specific data points. SDI, Socio-Demographic Index; ASMR, Age-Standardized Mortality Rate; ASDR, Age-Standardized Death Rate.

**Figure 6:**
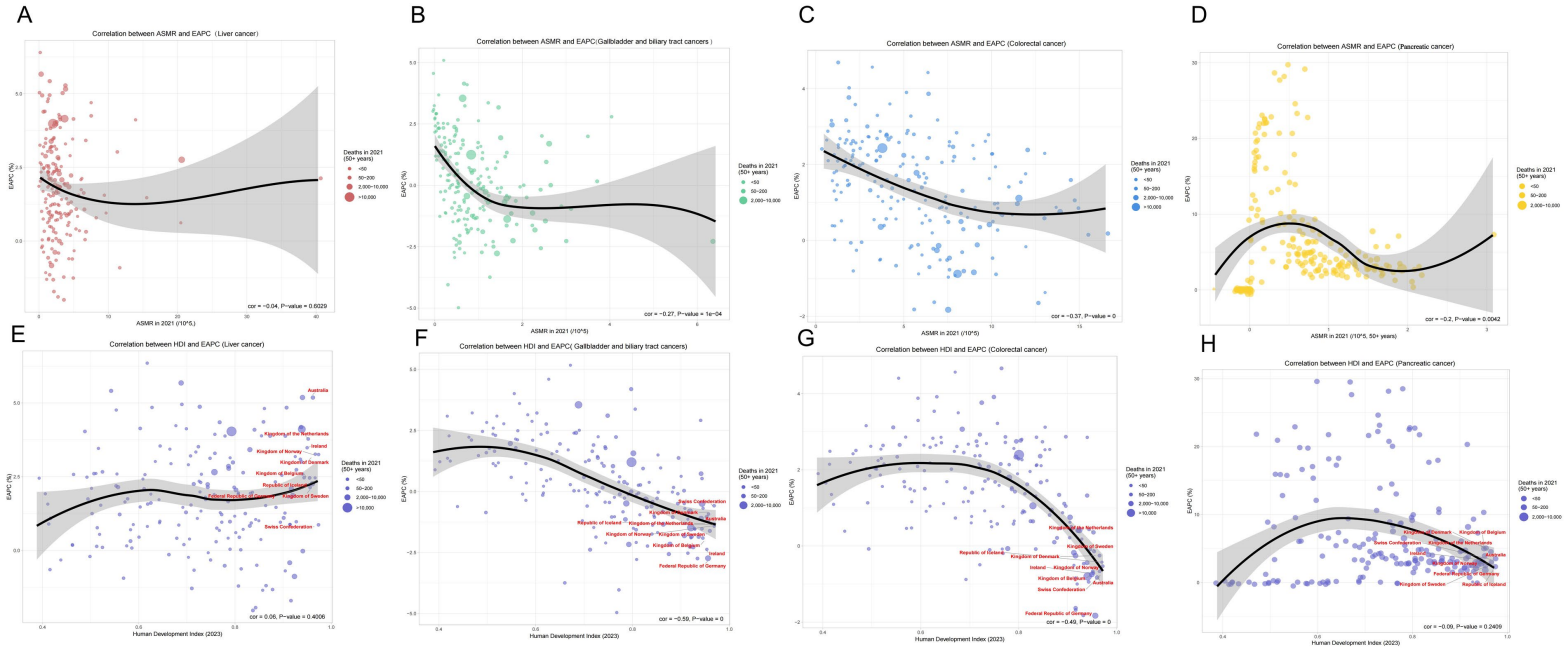
Correlations between age-standardized mortality rate (ASMR), human development index (HDI), and estimated annual percentage change (EAPC) for various cancers in 2021. **(A)** Correlation between ASMR and EAPC for liver cancer. The linear regression demonstrates a negative correlation (correlation coefficient = −0.04, *p*-value = 0.6029). **(B)** Correlation between ASMR and EAPC for gallbladder and biliary tract cancers, indicating a significant negative correlation (correlation coefficient = −0.27, *p*-value = 1e−04). **(C)** Correlation between ASMR and EAPC for colorectal cancer, exhibiting a marked negative correlation (correlation coefficient = −0.37, *p*-value = 0). **(D)** Correlation between ASMR and EAPC for pancreatic cancer, exhibiting a marked negative correlation (correlation coefficient = −0.2, *p*-value = 0.0042). **(E)** Correlation between HDI and EAPC for liver cancer, showing a negative correlation (correlation coefficient = −0.59, *p*-value = 0). **(F)** Correlation between HDI and EAPC for gallbladder and biliary tract cancers, reflecting a strong negative correlation (correlation coefficient = −0.49, *p*-value = 0). **(G)** Correlation between HDI and EAPC for colorectal cancer, illustrating a substantive negative relationship. **(H)** Correlation between HDI and EAPC for Pancreatic cancer, illustrating a substantive negative relationship. ASMR, Age-Standardized Mortality Rate; HDI, Human Development Index; EAPC, Estimated Annual Percentage Change.

Based on the Global Burden of Disease Study 1990–2021, gallbladder and biliary tract cancer (GBTC), liver cancer, colorectal cancer, and pancreatic cancer (PC) exhibited a distinct inverted U-shaped curve relationship between age-standardized mortality rates (ASMR) and both Socio-demographic Index (SDI) and Human Development Index (HDI): The ASMR of all four cancers initially increased with rising SDI, peaked within the SDI range of 0.7–0.9, and subsequently demonstrated persistent declines in regions with SDI > 0.8. Notable geographic anomalies manifested persistently higher-than-predicted burdens: GBTC in Andean Latin America (Bolivia, Chile) and high-income Asia Pacific; liver cancer in high-SDI East Asia (Japan, South Korea); colorectal cancer in Eastern Europe; and pancreatic cancer in high-SDI regions such as Japan and South Korea (Figure 5). A strong negative correlation emerged between annualized mortality change rates (EAPC) and 2021 HDI (all cancers: *R* ≈ –0.60, *p* < 0.001). Quantitative analysis revealed that each 0.1-unit increase in HDI accelerated ASMR decline rates by 1.2–1.8%, underscoring the pivotal regulatory role of regional development levels – mediated through healthcare resource allocation and risk factor control – in shaping cancer mortality trajectories (Figure 6).

### 3.5 Forecasting cancer–related burden (2021–2050)

**Figure 7:**
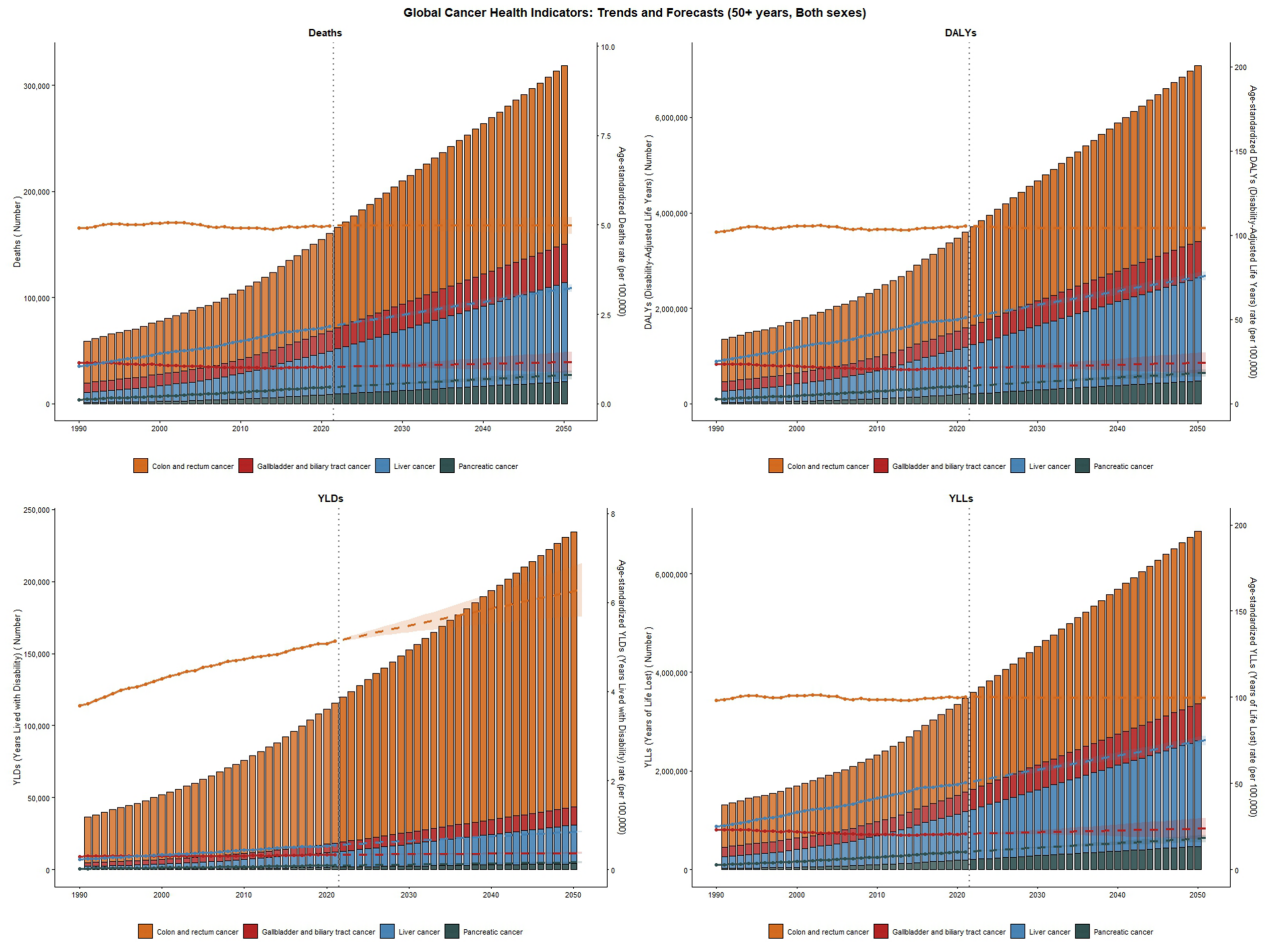
Trends in age-standardized disability-adjusted life years (DALYs), years of life lost (YLLs), and death rates for liver cancer, colorectal cancer, and gallbladder and biliary tract cancer from 1990 to Projections for 2050. This figure illustrates the age-standardized DALY rates (per 100,000 population) and age-standardized YLL rates for liver cancer, colorectal cancer, and gallbladder and biliary tract cancer from 1990 to projections for 2050. Additionally, it displays the corresponding age-standardized death rates (per 100,000 population) across the same time frame.

Based on the predictive data spanning from 1990 to 2050, three major cancer types exhibit distinct disease burden trajectories: for colorectal cancer, the total DALYs are projected to increase substantially, while the age-standardized Years of Life Lost (YLLs) rate shows a sustained decline. However, both the absolute count and age-standardized rate of Years Lived with Disability (YLDs) rise rapidly, indicating a growing quality-of-life burden among survivors. For liver cancer, upward trends are observed in DALYs and YLDs (both counts and age-standardized rates), whereas the age-standardized YLLs rate decreases only marginally. This pattern highlights escalating morbidity pressures despite limited mortality improvements. For gallbladder and biliary tract cancer, both DALYs and YLLs remain relatively stable in absolute counts and age-standardized rates. A modest increase in YLDs, however, underscores emerging needs for long-term survivorship management (Figure 7).

Collectively, these cancers demonstrate converging trends: declining mortality (reflected in falling YLLs rates), rising disability burdens (evident in ascending YLDs), and persistently high aggregate DALYs. These projections emphasize the imperative for targeted interventions addressing survivorship care alongside mortality reduction (Figure 7).

## 4 Discussion

Globally, the total incidence and mortality of cancers have increased markedly from 1990 to 2021. According to GBD 2021 data, the age-standardized incidence rate (ASIR) of all cancers rose slightly from 758.26 to 790.33 per 100,000 population, while total cases nearly doubled (from 34.8 million to 66.5 million) (25). In contrast, the age-standardized mortality rate (ASMR) for cancers attributable to high BMI in 2021 was 4.18 per 100,000 (95% UI: 1.71–6.80), with an estimated annual percentage change (EAPC) of +0.35% (95% CI: 0.32–0.38). This indicates that high BMI contributes to only a portion of total cancer mortality, yet its impact continues to increase globally (13).

This study systematically quantified the contribution of high body mass index (BMI) to the global burden of digestive system cancers (liver, colorectal, and gallbladder/bile duct cancers) in individuals over 50 years old using the GBD 2021 database, providing crucial insights for public health policy. The results indicate a substantial increase in mortality from 1990 to 2021: liver cancer deaths rose by 363%, colorectal cancer by 140%, and gallbladder/bile duct cancer by 109%. These findings underscore that high BMI is a major modifiable risk factor driving the rising burden of digestive system cancers, particularly liver and colorectal cancers (26).

Furthermore, analysis of the disease burden composition revealed that the impact of high BMI on gastrointestinal cancers is mainly reflected in Years of Life Lost (YLLs) rather than Years Lived with Disability (YLDs). For liver, colorectal, and gallbladder cancers, YLLs accounted for over 95% of total Disability-Adjusted Life Years (DALYs), indicating that obesity predominantly increases premature mortality rather than long-term disability. This emphasizes the urgent need for early detection, weight control, and preventive strategies to mitigate the fatal impact of high BMI.

Compared with the global all-age population, the high BMI–attributable burden among middle-aged and older adults is substantially higher, highlighting the cumulative impact of prolonged metabolic exposure. Quantitatively, in 2021 the global ASMRs for high BMI–related colorectal, gallbladder, and liver cancers were 4.18, 0.24, and 2.2 per 100,000, respectively, while the corresponding figures in the middle-aged and elderly populations reached 5.0, 1.0, and 2.2 per 100,000. This pattern indicates that obesity exerts a magnified carcinogenic effect with aging. Among the GICs cancers, liver cancer shows the fastest growth (EAPC = +2.4) and the largest contribution to overall mortality, reflecting the global transition from viral to metabolic etiologies (13).

For pancreatic cancer (PC), high BMI also plays a significant role in increasing mortality rates, with the disease burden following a similar pattern to that of liver and colorectal cancers. The relationship between ASMR and SDI/HDI for pancreatic cancer also follows an inverted U-shaped curve, with the highest mortality burden observed in high-SDI regions, while middle-SDI regions showed the fastest increase in mortality (EAPC: 18.1%), followed by low-middle-SDI regions (EAPC: 14.1%).

Regional variations offer vital guidance for developing tailored intervention strategies. East Asia reported the highest liver and colorectal cancer mortality burdens in 2021 (10,838.7 and 18,405.5 deaths, respectively), with China being particularly prominent. In China, colorectal cancer DALYs increased by 445%, from 75,537 (95% UI: 25,927.9–128,893.1) to 411,982.4 (95% UI: 169,014.4–692,218.2). South Asia and Southeast Asia experienced the fastest growth in mortality rates (liver cancer EAPC: 5.4, 95% CI: 5.3–5.5; colorectal cancer EAPC: 3.2, 95% CI: 3.1–3.3), potentially linked to rapid urbanization, increased consumption of processed foods, and reduced physical activity (27). High socio-demographic index (SDI) regions, such as high-income North America, have effectively reduced mortality rates (colorectal cancer EAPC: –0.8, 95% CI: −0.9 to −0.7) through mature obesity prevention and screening programs, offering replicable experiences for low- and middle-SDI regions. The rapid burden increase in low- and low-middle SDI regions (low SDI gallbladder/bile duct cancer EAPC: 1.8, 95% CI: 1.7–1.9) reflects challenges posed by limited healthcare resources and rising obesity rates (12). Policymakers should integrate data on obesity prevalence, healthcare accessibility, and cultural practices to design targeted strategies, such as promoting low-cost healthy eating programs in South Asia and strengthening colorectal cancer screening (e.g., fecal occult blood testing) in East Asia (27–29).

Gender and age patterns further clarify intervention priorities for high-risk populations. Males aged 65–74 years bear the primary burden, with peak mortality rates for liver cancer occurring at 60–64 years, gallbladder/bile duct cancer at 65–69 years, and colorectal cancer at 75–79 years. This male-dominated pattern may be associated with gender differences in metabolic syndrome (23), suggesting the need for enhanced BMI monitoring and lifestyle interventions among older men, such as community-based weight loss programs and regular health check-ups, to reduce the risk of liver and colorectal cancers (30). The inverse U-shaped relationship between ASMR and SDI/human development index (HDI) (peaking when SDI is 0.7–0.9; ASMR decreases by 1.2–1.8% for every 0.1 unit increase in HDI, *R* ≈ –0.60, *p* < 0.001) indicates that economic development and optimized healthcare resources can significantly alleviate the cancer burden. For instance, the successful experiences of high-SDI regions can serve as models for low-SDI regions, reducing mortality by improving primary healthcare capacity (31).

The findings of this study align with existing literature, confirming that high BMI is a significant risk factor for liver, colorectal, and gallbladder/bile duct cancers. For example, He et al. (32) reported a positive association between high BMI and liver cancer risk, particularly in South Asia, while Zhao et al. (33) quantified the dose–response relationship between obesity and colorectal cancer. This study extends these findings by, for the first time, revealing the differentiated burden of the three digestive system cancers within a unified framework. The rapid increase in liver cancer mortality in South Asia (EAPC: 5.4%) is consistent with regional trends observed by Hu et al. (34), while the anomalous burden pattern of gallbladder/bile duct cancer in Southeast Asia (EAPC: 1.8% in low-SDI areas) was predicted to continue worsening in a study by Hu et al. Compared to Zhao et al. (33) research, this study found more significant improvements in colorectal cancer mortality in high-income regions (North America, EAPC: −0.8%), which may be attributed to increased colonoscopy screening coverage and the implementation of precise prevention and control systems. Conversely, the rapid growth in low- and middle-SDI regions reflects the cumulative effect of unbalanced healthcare resource allocation. Notably, the hypothesis of widening health inequality proposed by Hu et al. (34) received empirical support in this study – the inverse U-shaped inflection point for gallbladder/bile duct cancer in the SDI 0.7–0.9 range suggests a critical threshold for healthcare resource investment.

Projections to 2050 indicate a continuous rise in both DALYs and YLDs (absolute counts and age-standardized rates) for liver and colorectal cancers, with a particularly pronounced increase in colorectal cancer YLDs, reflecting an escalating quality-of-life burden for survivors. While DALYs and YLLs for gallbladder/bile duct cancer remain relatively stable, a moderate increase in YLDs still warrants attention for long-term care needs. These trends emphasize the necessity of comprehensive interventions, including obesity prevention and control, early diagnosis, and survivor care. For instance, implementing multidisciplinary rehabilitation programs in high-burden regions (e.g., East Asia) can improve outcomes for colorectal cancer survivors, and strengthening healthcare infrastructure in low-SDI regions can address future burden increases.

Recent analyses from GBD 2019 and 2021 reveal that early-onset gastrointestinal cancers (ages 15–49) follow epidemiological trajectories markedly different from those observed in our ≥50-year population. Early-onset colorectal cancer shows a persistent rise in ASIR (AAPC: +0.37%) while experiencing declines in mortality and DALYs, whereas early-onset pancreatic, liver, and gallbladder/biliary cancers have demonstrated consistent decreases in ASIR, ASMR, and ASDR (e.g., early-onset pancreatic cancer ASMR AAPC: −0.44%; early-onset liver cancer ASMR AAPC: −0.97%). These trends reflect the impact of early-life lifestyle transitions—including adolescent obesity, Westernized diets, and reduced physical activity—on disease onset, while improvements in screening and treatment have contributed to declining mortality (35).

In contrast, our findings among adults aged ≥50 years highlight the cumulative metabolic consequences of long-term high BMI. Mortality attributable to high BMI continues to rise across liver cancer (EAPC: +2.4%), colorectal cancer (EAPC: −0.1%), gallbladder/biliary tract cancer (EAPC: −0.5%), and particularly pancreatic cancer in middle-SDI regions (EAPC reaching 18.1%). Moreover, YLLs account for more than 95% of DALYs across all cancers, indicating substantial fatal burden. The divergence between early-onset and older-age patterns suggests a life-course accumulation model, where early-onset cancers signal lifestyle-related risks emerging earlier in life, whereas older-age cancers reflect the long-term metabolic impact of sustained obesity. Thus, effective prevention requires integrating early-life lifestyle modification with mid- and late-life metabolic risk management and cancer screening to mitigate the growing burden of gastrointestinal cancers.

High BMI exacerbates the burden of digestive system cancers through multiple mechanisms, consistent with existing research (36). In liver cancer, high BMI leads to non-alcoholic fatty liver disease (NAFLD), which, upon progression to non-alcoholic steatohepatitis (NASH), increases hepatocellular carcinoma risk through chronic inflammation and hepatocyte damage (10). Furthermore, obesity-induced gut microbiota dysbiosis and chronic inflammation further elevate colorectal cancer risk (37, 38). These mechanistic insights provide a basis for personalized prevention. For example, identifying biomarkers for insulin resistance and gut microbiota dysbiosis can inform tailored nutritional counseling and colorectal screening programs for high-risk individuals, thereby reducing incidence (39, 40).

## 5 Conclusion

In summary, this study provides a scientific basis for precise public health policies by quantifying and forecasting the global and regional burden of high BMI-related digestive system cancers. In high-burden or rapidly growing regions like East Asia and South Asia, obesity prevention and control, early screening, and healthcare resource optimization can significantly reduce the cancer burden. Interventions targeting older men and low- to middle-SDI regions are particularly crucial, while the successful experiences of high-SDI regions offer valuable lessons globally. Future policies should integrate prevention, diagnosis, and survivor care to address the disease burden challenges projected until 2050.

## Data Availability

The original contributions presented in the study are publicly available. This data can be found here: http://ghdx.healthdata.org/gbd-results-tool.

## Footnotes

1
https://ghdx.healthdata.org/
