# Supplementary figures and images for: Attributable burden of high BMI-related gastrointestinal tract cancers among middle-aged and elderly populations globally, 1990–2021 and projected to 2050: analysis of GBD 2021
Source: Front Nutr. 2026 Jan 2;12:1674621. doi: 10.3389/fnut.2025.1674621 (PMC12807987; doi:10.3389/fnut.2025.1674621)

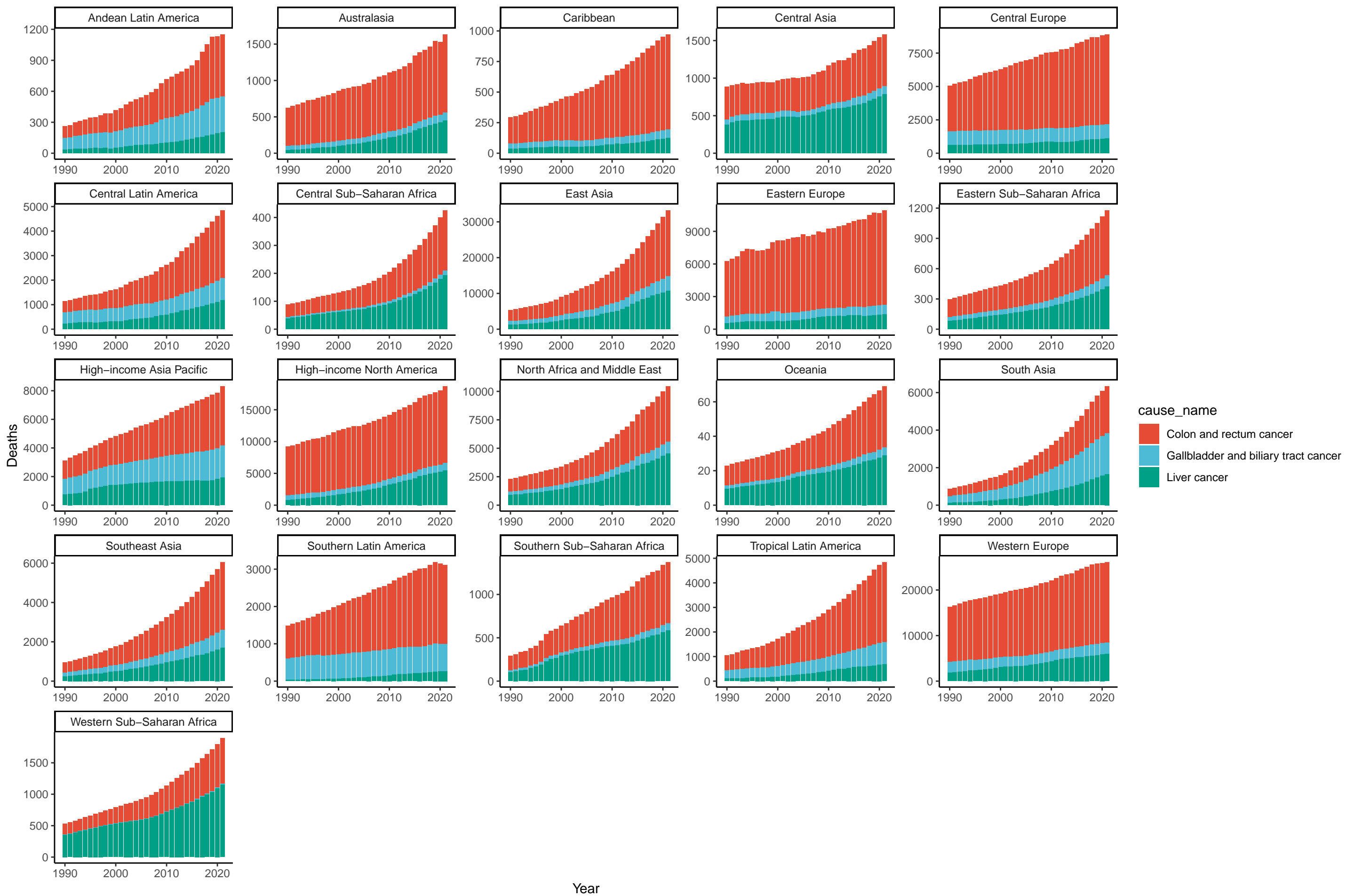

Supplement: Supplementary file 1 [file Image_1.pdf]
