# Supplementary material for: Attributable burden of high BMI-related gastrointestinal tract cancers among middle-aged and elderly populations globally, 1990–2021 and projected to 2050: analysis of GBD 2021
Source: Front Nutr. 2026 Jan 2;12:1674621. doi: 10.3389/fnut.2025.1674621 (PMC12807987; doi:10.3389/fnut.2025.1674621)
Supplement: Supplementary file 2 [file Table_1.doc]

****Table 1 High BMI-Attributable Gastrointestinal Cancer Mortality: Numbers, Rates, and Trends, 1990–2021****

|  | | **1990 Death cases** | **1990 ASMR** | **2021 Death cases** | **2021 ASMR** | **EAPC (1990–2021)** | **Cancer Type** |
| --- | --- | --- | --- | --- | --- | --- | --- |
| **Global** | | 38385 (16174.6, 62103.4) | 4.9 (2.1, 8) | 92012.9 (39255.8, 147266.7) | 5 (2.1, 8) | -0.1 (-0.1,-0.0) | Colon and rectum cancer |
|  |  | 9104.2 (6231.2, 12425.2) | 1.1 (0.8, 1.6) | 19035.2 (12758.2, 26584.4) | 1 (0.7, 1.4) | -0.5 (-0.5,-0.4) | Gallbladder and biliary tract cancer |
|  |  | 984.1 (-1865.8, 5660.2) | 0.1 (-0.2, 0.7) | 8753.7 (-2193.6, 25213) | 0.5 (-0.1, 1.3) | 4.4 (4.2,4.6) | Pancreatic cancer |
|  |  | 8853.5 (3568.3, 14600.1) | 1 (0.4, 1.7) | 40958 (16553.4, 68880.6) | 2.2 (0.9, 3.6) | 2.4 (2.3,2.5) | Liver cancer |
| **By**  **Sex** | **Female** | 21068.3 (8884.8, 34133.8) | 4.8 (2, 7.8) | 45133 (19229.5, 72025) | 4.4 (1.9, 7) | -0.4 (-0.5,-0.4) | Colon and rectum cancer |
|  |  | 6287.2 (4253.4, 8612.1) | 1.4 (0.9, 1.9) | 11544.3 (7523.6, 16414) | 1.1 (0.7, 1.6) | -0.9 (-0.9,-0.8) | Gallbladder and biliary tract cancer |
|  |  | 954.5 (-721, 3553) | 0.2 (-0.2, 0.8) | 5354.7 (-709.8, 14007.5) | 0.5 (-0.1, 1.4) | 3.0 (2.9,3.0) | Pancreatic cancer |
|  |  | 3924.3 (1529.3, 6472.2) | 0.9 (0.3, 1.4) | 16284.5 (6630, 27310.5) | 1.6 (0.7, 2.7) | 2.1 (2.0,2.1) | Liver cancer |
|  | **Male** | 17316.7 (7164.9, 28143.9) | 5 (2.1, 8.2) | 46879.8 (20091.9, 74977.4) | 5.7 (2.4, 9.1) | 0.3 (0.3,0.4) | Colon and rectum cancer |
|  |  | 2817 (1944.4, 3879.1) | 0.8 (0.6, 1.1) | 7490.9 (4841, 10654.1) | 0.9 (0.6, 1.3) | 0.3 (0.3,0.4) | Gallbladder and biliary tract cancer |
|  |  | 29.6 (-1219.4, 2155.8) | 0 (-0.3, 0.6) | 3399.1 (-1577, 11169.5) | 0.4 (-0.2, 1.3) | 10.3 (8.7,12.0) | Pancreatic cancer |
|  |  | 4929.2 (2044.1, 8193.4) | 1.2 (0.5, 2.1) | 24673.5 (10062.2, 42132.6) | 2.8 (1.1, 4.8) | 2.6 (2.5,2.7 | Liver cancer |
| **High SDI** | | 20794.1 (8752.9, 33711.1) | 8.5 (3.6, 13.8) | 34989.2 (14842.1, 56009.5) | 7.1 (3, 11.3) | -0.7 (-0.8,-0.7) | Colon and rectum cancer |
|  |  | 4154 (2810.7, 5737.5) | 1.7 (1.1, 2.3) | 6155.3 (3996.1, 8679) | 1.2 (0.8, 1.7) | -1.2 (-1.3,-1.1) | Gallbladder and biliary tract cancer |
|  |  | 832 (-796.7, 3489.4) | 0.3 (-0.3, 1.4) | 4385.3 (-793.2, 11922.7) | 1 (-0.1, 2.5) | 3.3 (3.2,3.5) | Pancreatic cancer |
|  |  | 3134.7 (1294.3, 5335) | 1.3 (0.5, 2.2) | 13492.8 (5528.9, 22396.5) | 2.9 (1.2, 4.8) | 2.6 (2.4,2.8) | Liver cancer |
| **High-middle SDI** | | 12640.2 (5374.8, 20405.3) | 6.1 (2.6, 9.9) | 31036.7 (13310.4, 50090.6) | 7.1 (3, 11.5) | 0.4 (0.3,0.5) | Colon and rectum cancer |
|  |  | 2869.7 (1953.7, 3948) | 1.4 (0.9, 1.9) | 5348.5 (3521.6, 7622.4) | 1.2 (0.8, 1.7) | -0.6 (-0.7,-0.5) | Gallbladder and biliary tract cancer |
|  |  | 419.5 (-571.7, 2046.4) | 0.2 (-0.3, 0.9) | 2916.3 (-629.5, 8481.7) | 0.7 (-0.1, 1.9) | 4.0 (3.9,4.1) | Pancreatic cancer |
|  |  | 2719.9 (1097, 4591.2) | 1.2 (0.5, 2.1) | 10074.4 (3958.5, 17746) | 2.3 (0.9, 4) | 1.9 (1.9,2.0) | Liver cancer |
| **Low SDI** | | 374.6 (134.4, 629.5) | 0.8 (0.3, 1.4) | 1290.7 (495.4, 2093.1) | 1.3 (0.5, 2.1) | 1.5 (1.4,1.6) | Colon and rectum cancer |
|  |  | 94.2 (58.3, 145.5) | 0.2 (0.1, 0.3) | 343.2 (207.4, 495.7) | 0.3 (0.2, 0.5) | 1.8 (1.7,1.9) | Gallbladder and biliary tract cancer |
|  |  | -25 (-51.8, 4.2) | -0.1 (-0.1, 0) | -3.6 (-78.9, 112.8) | 0 (-0.1, 0.1) | NA (NA,NA) | Pancreatic cancer |
|  |  | 342.8 (125.7, 620.2) | 0.7 (0.3, 1.3) | 1200.6 (444.7, 2072.3) | 1.1 (0.4, 1.9) | 1.3 (1.2,1.4) | Liver cancer |
| **Low-middle SDI** | | 1016.4 (382.8, 1645.5) | 0.8 (0.3, 1.4) | 5421.9 (2255.8, 8636) | 1.8 (0.8, 2.9) | 2.8 (2.7,2.8) | Colon and rectum cancer |
|  |  | 518.7 (351.2, 743.8) | 0.4 (0.3, 0.6) | 2164.7 (1414.8, 3013.9) | 0.7 (0.5, 1) | 1.9 (1.8,1.9) | Gallbladder and biliary tract cancer |
|  |  | -41 (-104.1, 55.8) | 0 (-0.1, 0) | 406.2 (-122.7, 1249.9) | 0.1 (0, 0.4) | 14.1 (11.3,17.0) | Pancreatic cancer |
|  |  | 1081 (405.2, 1999.7) | 0.8 (0.3, 1.6) | 5458.2 (2236.4, 9174.8) | 1.8 (0.7, 2.9) | 2.6 (2.5,2.7) | Liver cancer |
| **Middle SDI** | | 3485.2 (1295.1, 5706.2) | 1.7 (0.6, 2.8) | 19122.6 (8142.5, 30802.8) | 3.4 (1.4, 5.4) | 2.2 (2.1,2.2) | Colon and rectum cancer |
|  |  | 1447.7 (988.9, 2019.3) | 0.7 (0.5, 1) | 5001.4 (3316.7, 6971.1) | 0.9 (0.6, 1.2) | 0.5 (0.5,0.6) | Gallbladder and biliary tract cancer |
|  |  | -206 (-469.8, 219.4) | -0.1 (-0.2, 0.1) | 1033.9 (-644.1, 3657.1) | 0.2 (-0.1, 0.6) | 18.1 (15.1,21.1) | Pancreatic cancer |
|  |  | 1560.8 (641.1, 2543.6) | 0.7 (0.3, 1.2) | 10697.7 (4318.6, 18265) | 1.8 (0.7, 3.1) | 3.1 (3.0,3.2) | Liver cancer |
