# Supplementary material for: Attributable burden of high BMI-related gastrointestinal tract cancers among middle-aged and elderly populations globally, 1990–2021 and projected to 2050: analysis of GBD 2021
Source: Front Nutr. 2026 Jan 2;12:1674621. doi: 10.3389/fnut.2025.1674621 (PMC12807987; doi:10.3389/fnut.2025.1674621)
Supplement: Supplementary file 3 [file Table_2.docx]

| **Measure** | **Location** | **Number 1990** | **ASMR 1990** | **Number 2021** | **ASMR 2021** | **EAPC (95% CI)** |
| --- | --- | --- | --- | --- | --- | --- |
| Deaths | Global | 9104.2 (6231.2, 12425.2) | 1.1 (0.8, 1.6) | 19035.2 (12758.2, 26584.4) | 1 (0.7, 1.4) | -0.5 (-0.5,-0.4) |
| Deaths | Low SDI | 94.2 (58.3, 145.5) | 0.2 (0.1, 0.3) | 343.2 (207.4, 495.7) | 0.3 (0.2, 0.5) | 1.8 (1.7,1.9) |
| Deaths | Low-middle SDI | 518.7 (351.2, 743.8) | 0.4 (0.3, 0.6) | 2164.7 (1414.8, 3013.9) | 0.7 (0.5, 1) | 1.9 (1.8,1.9) |
| Deaths | Middle SDI | 1447.7 (988.9, 2019.3) | 0.7 (0.5, 1) | 5001.4 (3316.7, 6971.1) | 0.9 (0.6, 1.2) | 0.5 (0.5,0.6) |
| Deaths | High-middle SDI | 2869.7 (1953.7, 3948) | 1.4 (0.9, 1.9) | 5348.5 (3521.6, 7622.4) | 1.2 (0.8, 1.7) | -0.6 (-0.7,-0.5) |
| Deaths | High SDI | 4154 (2810.7, 5737.5) | 1.7 (1.1, 2.3) | 6155.3 (3996.1, 8679) | 1.2 (0.8, 1.7) | -1.2 (-1.3,-1.1) |

****Global and SDI-Based Trends in High BMI-Attributable Gallbladder and Biliary Tract Cancer Burden, 1990–2021****

****Global and SDI-Based Trends in High BMI-Attributable Gallbladder and Biliary Tract Cancer Burden, 2000–2021****

| **Measure** | **Location** | **Number 2000** | **ASMR 2000** | **Number 2021** | **ASMR 2021** | **EAPC (95% CI)** |
| --- | --- | --- | --- | --- | --- | --- |
| Deaths | Global | 10947.4 (7502.3, 15010.9) | 1.1 (0.7, 1.5) | 19035.2 (12758.2, 26584.4) | 1 (0.7, 1.4) | -0.5 (-0.5,-0.4) |
| Deaths | Low SDI | 130.5 (80.6, 198.2) | 0.2 (0.1, 0.4) | 343.2 (207.4, 495.7) | 0.3 (0.2, 0.5) | 1.8 (1.7,1.9) |
| Deaths | Low-middle SDI | 774.5 (516.2, 1099.9) | 0.5 (0.3, 0.7) | 2164.7 (1414.8, 3013.9) | 0.7 (0.5, 1) | 1.9 (1.8,1.9) |
| Deaths | Middle SDI | 1985.4 (1368.9, 2757.1) | 0.7 (0.5, 1) | 5001.4 (3316.7, 6971.1) | 0.9 (0.6, 1.2) | 0.5 (0.5,0.6) |
| Deaths | High-middle SDI | 3610.1 (2464.5, 4979.3) | 1.4 (1, 2) | 5348.5 (3521.6, 7622.4) | 1.2 (0.8, 1.7) | -0.6 (-0.7,-0.5) |
| Deaths | High SDI | 4426.9 (2991.1, 6127.9) | 1.5 (1, 2) | 6155.3 (3996.1, 8679) | 1.2 (0.8, 1.7) | -1.2 (-1.3,-1.1) |

| **Measure** | **Location** | **Number 2010** | **ASMR 2010** | **Number 2021** | **ASMR 2021** | **EAPC (95% CI)** |
| --- | --- | --- | --- | --- | --- | --- |
| Deaths | Global | 13491.6 (9250.3, 18613.6) | 1 (0.7, 1.4) | 19035.2 (12758.2, 26584.4) | 1 (0.7, 1.4) | -0.5 (-0.5,-0.4) |
| Deaths | Low SDI | 205.1 (125.9, 301.5) | 0.3 (0.2, 0.4) | 343.2 (207.4, 495.7) | 0.3 (0.2, 0.5) | 1.8 (1.7,1.9) |
| Deaths | Low-middle SDI | 1308.1 (855.7, 1841) | 0.6 (0.4, 0.9) | 2164.7 (1414.8, 3013.9) | 0.7 (0.5, 1) | 1.9 (1.8,1.9) |
| Deaths | Middle SDI | 2934.3 (2001.5, 4051.6) | 0.8 (0.5, 1.1) | 5001.4 (3316.7, 6971.1) | 0.9 (0.6, 1.2) | 0.5 (0.5,0.6) |
| Deaths | High-middle SDI | 4120.1 (2798.7, 5728.4) | 1.3 (0.9, 1.8) | 5348.5 (3521.6, 7622.4) | 1.2 (0.8, 1.7) | -0.6 (-0.7,-0.5) |
| Deaths | High SDI | 4903.5 (3276.2, 6845.2) | 1.3 (0.9, 1.8) | 6155.3 (3996.1, 8679) | 1.2 (0.8, 1.7) | -1.2 (-1.3,-1.1) |

****Global and SDI-Based Trends in High BMI-Attributable Gallbladder and Biliary Tract Cancer Burden, 2010–2021****

| **Measure** | **Location** | **Number 1990** | **ASR 1990** | **Number 2021** | **ASR 2021** | **EAPC (95% CI)** |
| --- | --- | --- | --- | --- | --- | --- |
| DALYs (Disability-Adjusted Life Years) | Global | 200194.9 (137599.4, 272531.9) | 23.6 (16.2, 32.1) | 400865.1 (270792.7, 556609.9) | 21.1 (14.2, 29.3) | -0.5 (-0.6,-0.4) |
| DALYs (Disability-Adjusted Life Years) | Low SDI | 2477.5 (1520.7, 3830.7) | 4.9 (3, 7.6) | 8599.2 (5186.3, 12429.5) | 7.8 (4.7, 11.3) | 1.6 (1.6,1.7) |
| DALYs (Disability-Adjusted Life Years) | Low-middle SDI | 12940.5 (8749.5, 18517) | 9.8 (6.6, 14) | 52667.1 (34164.4, 73055.3) | 16.6 (10.8, 23.1) | 1.9 (1.8,1.9) |
| DALYs (Disability-Adjusted Life Years) | Middle SDI | 35435.1 (24178.3, 49423.2) | 15.8 (10.8, 22.1) | 115706.6 (77055.9, 160820.3) | 19.1 (12.7, 26.6) | 0.5 (0.4,0.6) |
| DALYs (Disability-Adjusted Life Years) | High-middle SDI | 64571.5 (44060.2, 88802.5) | 29.4 (20, 40.5) | 112760.9 (74210.3, 160455.3) | 25.4 (16.7, 36.1) | -0.7 (-0.8,-0.6) |
| DALYs (Disability-Adjusted Life Years) | High SDI | 84336.4 (57355.3, 115893.1) | 34.7 (23.6, 47.7) | 110675.7 (73231.4, 154615.1) | 23.8 (15.8, 33.1) | -1.4 (-1.5,-1.3) |

****Global and SDI-Based Trends in High BMI-Attributable Gallbladder and Biliary Tract Cancer Burden, 1990–2021****

| **Measure** | **Location** | **Number 1990** | **ASR 1990** | **Number 2021** | **ASR 2021** | **EAPC (95% CI)** |
| --- | --- | --- | --- | --- | --- | --- |
| YLDs (Years Lived with Disability) | Global | 2433.3 (1503.6, 3654) | 0.3 (0.2, 0.4) | 6274.4 (3866.7, 9467.9) | 0.3 (0.2, 0.5) | 0.3 (0.2,0.4) |
| YLDs (Years Lived with Disability) | Low SDI | 21.4 (12.2, 35.5) | 0 (0, 0.1) | 77.8 (43.9, 124.8) | 0.1 (0, 0.1) | 1.8 (1.7,1.9) |
| YLDs (Years Lived with Disability) | Low-middle SDI | 115.7 (69.8, 183.6) | 0.1 (0.1, 0.1) | 496.6 (299, 774) | 0.2 (0.1, 0.3) | 2.0 (1.9,2.0) |
| YLDs (Years Lived with Disability) | Middle SDI | 326.8 (197.6, 499.3) | 0.2 (0.1, 0.2) | 1365.4 (821.7, 2163.5) | 0.2 (0.1, 0.4) | 1.3 (1.1,1.4) |
| YLDs (Years Lived with Disability) | High-middle SDI | 709.1 (430.3, 1069.5) | 0.3 (0.2, 0.5) | 1782.5 (1077.8, 2745.5) | 0.4 (0.2, 0.6) | 0.5 (0.4,0.5) |
| YLDs (Years Lived with Disability) | High SDI | 1256 (776.7, 1894.2) | 0.5 (0.3, 0.8) | 2546.1 (1555.1, 3838.5) | 0.5 (0.3, 0.8) | 0.1 (0.0,0.1) |

****Global and SDI-Based Trends in High BMI-Attributable Gallbladder and Biliary Tract Cancer Burden, 1990–2021****

****Global and SDI-Based Trends in High BMI-Attributable Gallbladder and Biliary Tract Cancer Burden, 1990–2021****

| **Measure** | **Location** | **Number 1990** | **ASR 1990** | **Number 2021** | **ASR 2021** | **EAPC (95% CI)** |
| --- | --- | --- | --- | --- | --- | --- |
| YLLs (Years of Life Lost) | Global | 197761.5 (135965.1, 269158) | 23.3 (16, 31.7) | 394590.7 (266106, 547982.8) | 20.7 (14, 28.8) | -0.5 (-0.6,-0.4) |
| YLLs (Years of Life Lost) | Low SDI | 2456.1 (1507.5, 3794.4) | 4.8 (3, 7.5) | 8521.4 (5139.8, 12315.1) | 7.8 (4.7, 11.2) | 1.6 (1.6,1.7) |
| YLLs (Years of Life Lost) | Low-middle SDI | 12824.9 (8667.7, 18358) | 9.7 (6.5, 13.9) | 52170.5 (33840.4, 72339.3) | 16.5 (10.7, 22.9) | 1.9 (1.8,1.9) |
| YLLs (Years of Life Lost) | Middle SDI | 35108.3 (23945, 48967.7) | 15.7 (10.7, 21.8) | 114341.2 (76051.5, 158818) | 18.9 (12.5, 26.3) | 0.5 (0.4,0.6) |
| YLLs (Years of Life Lost) | High-middle SDI | 63862.4 (43557.9, 87805.1) | 29.1 (19.8, 40) | 110978.4 (73011.1, 157967.6) | 25 (16.4, 35.6) | -0.7 (-0.8,-0.6) |
| YLLs (Years of Life Lost) | High SDI | 83080.4 (56451.6, 114171.2) | 34.2 (23.2, 47) | 108129.6 (71461.6, 151198.8) | 23.2 (15.4, 32.4) | -1.4 (-1.5,-1.3) |

****Burden of Gallbladder and Biliary Tract Cancer Deaths Attributable to High BMI in 21 GBD Regions, 1990–2021****

| Measure | Location | Number 1990 | ASMR 1990 | Number 2021 | ASMR 2021 | EAPC (95% CI) |
| --- | --- | --- | --- | --- | --- | --- |
| Deaths | Global | 2817 (1944.4, 3879.1) | 0.8 (0.6, 1.1) | 7490.9 (4841, 10654.1) | 0.9 (0.6, 1.3) | 0.3 (0.3,0.4) |
| Deaths | Andean Latin America | 28.3 (17.4, 44) | 1.4 (0.9, 2.2) | 96.9 (57.6, 152.7) | 1.6 (1, 2.6) | 0.5 (0.4,0.6) |
| Deaths | Australasia | 18.3 (12.1, 25.7) | 0.8 (0.6, 1.2) | 43.2 (27.9, 61.6) | 0.8 (0.5, 1.1) | -0.5 (-0.8,-0.2) |
| Deaths | Caribbean | 13.3 (9.1, 18.6) | 0.5 (0.3, 0.7) | 27.2 (17.8, 39.1) | 0.5 (0.3, 0.7) | -0.3 (-0.5,-0.1) |
| Deaths | Central Asia | 18.6 (12.4, 25.8) | 0.5 (0.3, 0.7) | 34.2 (22.7, 49.9) | 0.5 (0.3, 0.7) | -0.5 (-0.9,-0.1) |
| Deaths | Central Europe | 253.4 (170.6, 355.4) | 1.9 (1.3, 2.7) | 379.4 (253.2, 537.2) | 1.8 (1.2, 2.6) | -0.2 (-0.3,-0.1) |
| Deaths | Central Latin America | 104.7 (70.9, 145.4) | 1.3 (0.9, 1.8) | 275.8 (181.4, 393.4) | 1.1 (0.7, 1.6) | -0.8 (-1.0,-0.6) |
| Deaths | Central Sub-Saharan Africa | 1.1 (0.5, 1.9) | 0.1 (0, 0.1) | 4.8 (2.1, 8.4) | 0.1 (0, 0.2) | 2.4 (2.1,2.6) |
| Deaths | East Asia | 409.6 (240.4, 650.8) | 0.5 (0.3, 0.9) | 1814.3 (949.2, 2963.8) | 0.8 (0.4, 1.4) | 1.5 (1.4,1.6) |
| Deaths | Eastern Europe | 139 (95.5, 188.8) | 0.7 (0.5, 0.9) | 241.3 (154.8, 341) | 0.9 (0.5, 1.2) | 0.2 (-0.1,0.5) |
| Deaths | Eastern Sub-Saharan Africa | 9.6 (5.1, 14.8) | 0.1 (0.1, 0.2) | 29.4 (14.7, 44.6) | 0.2 (0.1, 0.3) | 1.2 (1.1,1.2) |
| Deaths | High-income Asia Pacific | 424.8 (296.6, 592.7) | 2.4 (1.7, 3.4) | 1072 (698.3, 1517.8) | 2.2 (1.4, 3.1) | -0.5 (-0.6,-0.5) |
| Deaths | High-income North America | 245.3 (164.7, 340.9) | 0.8 (0.5, 1.1) | 465.9 (306.6, 639.7) | 0.7 (0.5, 1) | -0.3 (-0.4,-0.2) |
| Deaths | North Africa and Middle East | 82.2 (48.3, 129.6) | 0.5 (0.3, 0.8) | 363 (202.2, 554.5) | 0.8 (0.5, 1.3) | 1.7 (1.6,1.8) |
| Deaths | Oceania | 0.7 (0.4, 1.1) | 0.2 (0.1, 0.4) | 2.1 (1.1, 3.4) | 0.3 (0.1, 0.4) | 0.6 (0.6,0.7) |
| Deaths | South Asia | 105.5 (61.3, 162.2) | 0.2 (0.1, 0.3) | 638.6 (333.8, 957.4) | 0.4 (0.2, 0.7) | 3.0 (2.9,3.1) |
| Deaths | Southeast Asia | 74.4 (39.6, 120.8) | 0.3 (0.2, 0.5) | 409.8 (185.7, 655.4) | 0.7 (0.3, 1.1) | 2.5 (2.4,2.6) |
| Deaths | Southern Latin America | 125.3 (82.4, 179.6) | 2.9 (1.9, 4.2) | 232.6 (152, 334) | 2.8 (1.8, 4) | -0.2 (-0.3,-0.0) |
| Deaths | Southern Sub-Saharan Africa | 5.6 (2.6, 9.7) | 0.2 (0.1, 0.4) | 22.3 (11.3, 33.8) | 0.5 (0.2, 0.7) | 2.2 (2.0,2.4) |
| Deaths | Tropical Latin America | 79.4 (53.4, 111.6) | 0.9 (0.6, 1.3) | 302.9 (202.5, 424.2) | 1.2 (0.8, 1.7) | 0.8 (0.7,1.0) |
| Deaths | Western Europe | 677.3 (462.4, 938.4) | 1.3 (0.9, 1.8) | 1033.1 (681.1, 1452.2) | 1.1 (0.7, 1.5) | -0.8 (-0.9,-0.6) |

****Burden of Gallbladder and Biliary Tract Cancer Deaths Attributable to High BMI in Country 1990–2021****

| Measure | Location | Number 1990 | ASMR 1990 | Number 2021 | ASMR 2021 | EAPC (95% CI) |
| --- | --- | --- | --- | --- | --- | --- |
| Deaths | China | 918.6 (595.5, 1327.3) | 0.6 (0.4, 0.8) | 3827.7 (2169.6, 5737.7) | 0.8 (0.5, 1.3) | 1.2 (1.2,1.3) |
| Deaths | Democratic People's Republic of Korea | 12 (6.3, 22) | 0.4 (0.2, 0.7) | 35.9 (17.6, 71.2) | 0.5 (0.3, 1) | 0.9 (0.9,1.0) |
| Deaths | Taiwan (Province of China) | 27.4 (18.6, 37.3) | 0.8 (0.6, 1.1) | 104.6 (68.3, 150.3) | 1.1 (0.7, 1.6) | -0.2 (-0.9,0.5) |
| Deaths | Kingdom of Cambodia | 1.7 (0.8, 3.3) | 0.2 (0.1, 0.4) | 6.2 (3, 14.3) | 0.2 (0.1, 0.6) | 0.8 (0.5,1.1) |
| Deaths | Republic of Indonesia | 26.5 (15, 49.2) | 0.1 (0.1, 0.3) | 99.4 (53.9, 216.9) | 0.2 (0.1, 0.4) | 1.3 (1.3,1.4) |
| Deaths | Lao People's Democratic Republic | 0.9 (0.4, 1.7) | 0.2 (0.1, 0.4) | 2.3 (1.1, 5.1) | 0.2 (0.1, 0.6) | 0.4 (0.3,0.6) |
| Deaths | Malaysia | 8.2 (4.2, 12.8) | 0.4 (0.2, 0.7) | 32.3 (18, 50.7) | 0.5 (0.3, 0.8) | 0.4 (0.2,0.5) |
| Deaths | Republic of Maldives | 0.1 (0, 0.1) | 0.3 (0.1, 0.5) | 0.1 (0.1, 0.2) | 0.2 (0.1, 0.4) | -1.0 (-1.2,-0.9) |
| Deaths | Republic of the Union of Myanmar | 9.7 (4.7, 18.7) | 0.2 (0.1, 0.4) | 20.1 (9.9, 46.4) | 0.2 (0.1, 0.4) | -0.2 (-0.4,-0.1) |
| Deaths | Republic of the Philippines | 6.6 (4, 11.3) | 0.1 (0.1, 0.2) | 31.7 (19.6, 55.1) | 0.2 (0.1, 0.3) | 1.7 (1.6,1.8) |
| Deaths | Democratic Socialist Republic of Sri Lanka | 12.8 (4.8, 21.5) | 0.6 (0.2, 1) | 16.9 (8.1, 33.8) | 0.3 (0.1, 0.6) | -4.0 (-4.8,-3.2) |
| Deaths | Kingdom of Thailand | 109.4 (60.5, 171.3) | 1.5 (0.8, 2.4) | 633.5 (314.4, 1030.2) | 2.6 (1.3, 4.2) | 1.6 (1.5,1.7) |
| Deaths | Democratic Republic of Timor-Leste | 0 (0, 0.1) | 0.1 (0, 0.2) | 0.2 (0.1, 0.4) | 0.1 (0.1, 0.2) | 0.5 (0.2,0.8) |
| Deaths | Socialist Republic of Viet Nam | 10.3 (5.7, 16.7) | 0.1 (0.1, 0.2) | 48.1 (25.2, 83.6) | 0.2 (0.1, 0.4) | 2.3 (2.2,2.4) |
| Deaths | Republic of Fiji | 0.4 (0.2, 0.7) | 0.6 (0.4, 1) | 1.5 (0.8, 2.4) | 1 (0.5, 1.5) | 1.5 (1.4,1.7) |
| Deaths | Republic of Kiribati | 0 (0, 0) | 0.3 (0.2, 0.6) | 0.1 (0, 0.1) | 0.5 (0.2, 0.8) | 1.2 (1.1,1.3) |
| Deaths | Republic of the Marshall Islands | 0 (0, 0) | 0.5 (0.3, 0.8) | 0 (0, 0.1) | 0.5 (0.3, 0.8) | 0.0 (-0.1,0.1) |
| Deaths | Federated States of Micronesia | 0.1 (0, 0.1) | 0.5 (0.3, 0.9) | 0.1 (0, 0.1) | 0.5 (0.3, 0.9) | -0.1 (-0.1,-0.0) |
| Deaths | Independent State of Papua New Guinea | 0.7 (0.4, 1.3) | 0.2 (0.1, 0.3) | 1.9 (1.1, 3.3) | 0.2 (0.1, 0.3) | -0.3 (-0.4,-0.3) |
| Deaths | Independent State of Samoa | 0.1 (0.1, 0.1) | 0.5 (0.3, 0.8) | 0.2 (0.1, 0.3) | 0.5 (0.3, 0.9) | 0.1 (0.0,0.1) |
| Deaths | Solomon Islands | 0.1 (0, 0.1) | 0.3 (0.1, 0.5) | 0.2 (0.1, 0.4) | 0.3 (0.2, 0.5) | 0.3 (0.2,0.5) |
| Deaths | Kingdom of Tonga | 0 (0, 0.1) | 0.4 (0.2, 0.7) | 0.1 (0, 0.1) | 0.5 (0.3, 0.7) | 0.3 (0.2,0.4) |
| Deaths | Republic of Vanuatu | 0 (0, 0.1) | 0.3 (0.1, 0.4) | 0.1 (0.1, 0.2) | 0.3 (0.2, 0.5) | 0.2 (0.1,0.3) |
| Deaths | Republic of Armenia | 3.1 (2, 4.5) | 0.5 (0.3, 0.8) | 12.2 (8, 17.4) | 1.3 (0.8, 1.8) | 3.3 (2.5,4.2) |
| Deaths | Republic of Azerbaijan | 5.8 (3.2, 9.7) | 0.5 (0.3, 0.9) | 11.2 (5.6, 19.1) | 0.5 (0.3, 0.9) | -0.2 (-0.3,-0.1) |
| Deaths | Georgia | 16.2 (9.9, 24.5) | 1.2 (0.7, 1.8) | 14.4 (9.2, 21.5) | 1.1 (0.7, 1.6) | -1.1 (-2.2,0.1) |
| Deaths | Republic of Kazakhstan | 22 (14, 34) | 0.8 (0.5, 1.3) | 30.8 (20.4, 43.7) | 0.8 (0.5, 1.2) | -0.4 (-0.6,-0.1) |
| Deaths | Kyrgyz Republic | 4.6 (2.9, 6.8) | 0.7 (0.5, 1.1) | 8.1 (4.9, 11.9) | 0.8 (0.5, 1.2) | -1.8 (-2.9,-0.6) |
| Deaths | Mongolia | 4.1 (2.1, 7) | 1.8 (0.9, 3.1) | 7.7 (4.5, 12.9) | 1.7 (1, 2.8) | -0.5 (-0.6,-0.3) |
| Deaths | Republic of Tajikistan | 0.6 (0.3, 1.1) | 0.1 (0.1, 0.2) | 1 (0.5, 1.6) | 0.1 (0, 0.1) | -1.2 (-1.4,-1.0) |
| Deaths | Turkmenistan | 6.8 (4.5, 9.8) | 1.7 (1.1, 2.4) | 4.5 (2.7, 7) | 0.5 (0.3, 0.8) | -4.9 (-6.1,-3.7) |
| Deaths | Republic of Uzbekistan | 3.8 (2, 6.3) | 0.2 (0.1, 0.3) | 15.5 (9.8, 24) | 0.3 (0.2, 0.4) | 1.9 (1.3,2.5) |
| Deaths | Republic of Albania | 4 (2.3, 6.5) | 1 (0.6, 1.6) | 9.5 (5, 16.1) | 1 (0.5, 1.7) | 0.2 (0.0,0.4) |
| Deaths | Bosnia and Herzegovina | 26.5 (15.2, 40.4) | 3.1 (1.8, 4.8) | 31.5 (18.2, 56.3) | 2.2 (1.3, 4) | -1.6 (-1.9,-1.4) |
| Deaths | Republic of Bulgaria | 28.3 (18.6, 40.3) | 1.1 (0.7, 1.6) | 33.1 (21.1, 49.4) | 1 (0.6, 1.5) | -0.2 (-0.4,0.1) |
| Deaths | Republic of Croatia | 31.4 (20.5, 45.1) | 2.5 (1.6, 3.6) | 46 (29.4, 66.1) | 2.2 (1.4, 3.1) | -0.5 (-0.6,-0.4) |
| Deaths | Czech Republic | 174.4 (112.6, 252) | 5.6 (3.6, 8.1) | 149.1 (92.7, 219.4) | 3 (1.8, 4.3) | -2.4 (-2.4,-2.3) |
| Deaths | Hungary | 171.1 (111.1, 244) | 5.2 (3.4, 7.5) | 108.9 (70.4, 155.1) | 2.4 (1.6, 3.5) | -2.6 (-2.8,-2.5) |
| Deaths | North Macedonia | 7.5 (4.5, 11.2) | 1.9 (1.2, 2.9) | 13.5 (7.7, 21.1) | 2 (1.1, 3.1) | -0.2 (-0.4,0.1) |
| Deaths | Montenegro | 1.4 (0.8, 2.2) | 1 (0.6, 1.6) | 2.9 (1.7, 4.6) | 1.4 (0.8, 2.2) | 0.9 (0.8,1.1) |
| Deaths | Republic of Poland | 339.5 (228.5, 474.9) | 3.6 (2.4, 5) | 380.5 (249, 536.1) | 2.3 (1.5, 3.2) | -1.8 (-1.9,-1.6) |
| Deaths | Romania | 91.9 (60.8, 131) | 1.5 (1, 2.1) | 97.7 (63.5, 140.5) | 1.2 (0.8, 1.7) | -0.6 (-0.9,-0.4) |
| Deaths | Republic of Serbia | 42.4 (24.3, 68.2) | 1.9 (1.1, 3.1) | 69.7 (40.3, 110.8) | 1.8 (1.1, 2.9) | -0.6 (-0.7,-0.4) |
| Deaths | Slovak Republic | 54.7 (32.8, 84.9) | 4.1 (2.5, 6.4) | 68.4 (38.5, 112.2) | 3.2 (1.8, 5.2) | -1.0 (-1.0,-0.9) |
| Deaths | Republic of Slovenia | 15.7 (10.2, 22.6) | 2.9 (1.9, 4.1) | 20.3 (13, 29.3) | 1.9 (1.2, 2.8) | -1.6 (-1.7,-1.4) |
| Deaths | Republic of Belarus | 22 (14, 32.3) | 0.8 (0.5, 1.1) | 39.4 (24.6, 59.5) | 1.1 (0.7, 1.6) | 1.0 (0.8,1.2) |
| Deaths | Republic of Estonia | 6.4 (4.1, 9.3) | 1.4 (0.9, 2.1) | 8.6 (5.5, 12.6) | 1.3 (0.9, 1.9) | -0.6 (-0.8,-0.4) |
| Deaths | Republic of Latvia | 6.8 (4.3, 9.7) | 0.9 (0.5, 1.2) | 9.4 (6, 13.9) | 1 (0.6, 1.5) | 0.2 (-0.0,0.4) |
| Deaths | Republic of Lithuania | 12.3 (8, 17.5) | 1.2 (0.8, 1.8) | 20.3 (13, 29.8) | 1.5 (1, 2.2) | -0.1 (-0.5,0.4) |
| Deaths | Republic of Moldova | 9.6 (6.3, 13.4) | 1 (0.7, 1.4) | 10 (6.5, 14.2) | 0.8 (0.5, 1.1) | -0.9 (-1.2,-0.6) |
| Deaths | Russian Federation | 426.1 (289.7, 586.1) | 1.1 (0.7, 1.5) | 630.9 (414.4, 886.3) | 1.2 (0.8, 1.7) | -0.3 (-0.7,0.0) |
| Deaths | Ukraine | 116.6 (68.6, 182.1) | 0.7 (0.4, 1.1) | 142.5 (84.2, 219.9) | 0.8 (0.5, 1.3) | 0.5 (0.2,0.7) |
| Deaths | Brunei Darussalam | 0.2 (0.1, 0.3) | 1.1 (0.6, 1.8) | 0.8 (0.4, 1.2) | 1.1 (0.6, 1.8) | 0.4 (0.3,0.6) |
| Deaths | Japan | 905.6 (621.2, 1236.3) | 2.5 (1.7, 3.4) | 1742.7 (1064.3, 2490.3) | 1.7 (1.1, 2.4) | -1.4 (-1.5,-1.4) |
| Deaths | Republic of Korea | 167.6 (96.7, 263.7) | 3.1 (1.8, 4.8) | 472.1 (257.3, 760.1) | 2.3 (1.2, 3.7) | -1.1 (-1.2,-1.0) |
| Deaths | Republic of Singapore | 2.3 (1.5, 3.2) | 0.5 (0.3, 0.7) | 8.6 (5.3, 12.6) | 0.5 (0.3, 0.7) | -0.6 (-0.8,-0.5) |
| Deaths | Australia | 44.1 (29.3, 62.2) | 1 (0.7, 1.5) | 85 (55, 120.6) | 0.8 (0.5, 1.1) | -1.0 (-1.1,-0.9) |
| Deaths | New Zealand | 8.4 (5.5, 12) | 1 (0.6, 1.4) | 23.6 (15, 33.7) | 1.2 (0.8, 1.7) | 0.6 (0.1,1.1) |
| Deaths | Principality of Andorra | 0.2 (0.1, 0.3) | 1.6 (0.8, 2.6) | 0.4 (0.2, 0.7) | 1.1 (0.6, 1.9) | -0.8 (-0.9,-0.6) |
| Deaths | Republic of Austria | 67.2 (43.8, 96.4) | 2.5 (1.6, 3.5) | 47.1 (29.9, 68.4) | 1.1 (0.7, 1.6) | -2.6 (-2.9,-2.4) |
| Deaths | Kingdom of Belgium | 33.2 (21.7, 47.6) | 0.9 (0.6, 1.4) | 30.6 (19.4, 45) | 0.5 (0.3, 0.8) | -2.0 (-2.2,-1.8) |
| Deaths | Republic of Cyprus | 2.1 (1.2, 3.3) | 1.4 (0.8, 2.2) | 4 (2.3, 6.5) | 0.9 (0.5, 1.5) | -1.4 (-1.5,-1.3) |
| Deaths | Kingdom of Denmark | 13.1 (8.7, 18.5) | 0.7 (0.5, 1) | 17.4 (11.2, 24.9) | 0.6 (0.4, 0.9) | -0.8 (-1.0,-0.6) |
| Deaths | Republic of Finland | 29.3 (19.1, 42.1) | 1.8 (1.2, 2.6) | 38.8 (24.5, 56.8) | 1.3 (0.8, 1.8) | -1.5 (-1.7,-1.2) |
| Deaths | French Republic | 224.7 (149.5, 314.8) | 1.2 (0.8, 1.6) | 216.8 (135, 313.8) | 0.6 (0.4, 0.9) | -2.4 (-2.4,-2.3) |
| Deaths | Federal Republic of Germany | 902.5 (579.2, 1282.8) | 3.1 (2, 4.4) | 654.8 (413.7, 946.9) | 1.4 (0.9, 2) | -2.8 (-3.2,-2.4) |
| Deaths | Hellenic Republic | 34.6 (23.4, 48.6) | 1 (0.7, 1.5) | 55.8 (35.7, 80.5) | 1 (0.6, 1.4) | -0.1 (-0.7,0.6) |
| Deaths | Republic of Iceland | 0.7 (0.4, 1) | 1 (0.7, 1.5) | 0.9 (0.6, 1.3) | 0.7 (0.4, 1) | -1.4 (-1.5,-1.3) |
| Deaths | Ireland | 8.9 (5.8, 12.7) | 1 (0.6, 1.4) | 9.4 (5.8, 13.6) | 0.5 (0.3, 0.7) | -2.3 (-2.7,-2.0) |
| Deaths | State of Israel | 12.3 (7.8, 17.7) | 1.1 (0.7, 1.7) | 16.1 (10, 23.7) | 0.6 (0.4, 0.8) | -2.5 (-2.7,-2.4) |
| Deaths | Republic of Italy | 375.3 (255, 522.7) | 1.9 (1.3, 2.6) | 574.6 (363.4, 822.1) | 1.6 (1, 2.3) | -0.8 (-0.9,-0.6) |
| Deaths | Grand Duchy of Luxembourg | 1.5 (1, 2.1) | 1.3 (0.8, 1.8) | 1.7 (1.1, 2.4) | 0.7 (0.4, 1) | -1.8 (-2.0,-1.7) |
| Deaths | Republic of Malta | 0.7 (0.4, 0.9) | 0.7 (0.5, 1) | 1.1 (0.7, 1.6) | 0.5 (0.3, 0.7) | -1.5 (-1.6,-1.4) |
| Deaths | Kingdom of the Netherlands | 63.5 (42.2, 90.1) | 1.4 (0.9, 2) | 73.2 (46.8, 105.7) | 0.9 (0.6, 1.3) | -1.3 (-1.6,-1.1) |
| Deaths | Kingdom of Norway | 11.6 (7.8, 16.1) | 0.7 (0.5, 1) | 13 (8.5, 18.5) | 0.6 (0.4, 0.8) | -1.4 (-1.7,-1.1) |
| Deaths | Portuguese Republic | 39.4 (26.3, 56.3) | 1.3 (0.9, 1.9) | 54.8 (34.7, 79.8) | 0.9 (0.6, 1.3) | -1.3 (-1.5,-1.2) |
| Deaths | Kingdom of Spain | 208.9 (136.7, 297) | 1.7 (1.1, 2.4) | 249 (155.3, 366.5) | 1 (0.7, 1.5) | -1.9 (-2.0,-1.7) |
| Deaths | Kingdom of Sweden | 82.2 (53.8, 118) | 2.4 (1.6, 3.4) | 82.4 (52.2, 119.3) | 1.6 (1, 2.3) | -1.7 (-2.1,-1.3) |
| Deaths | Swiss Confederation | 16.3 (10.7, 23.1) | 0.7 (0.4, 1) | 27 (17, 39.3) | 0.6 (0.4, 0.9) | -0.7 (-0.9,-0.4) |
| Deaths | United Kingdom of Great Britain and Northern Ireland | 151.9 (102.2, 211.5) | 0.7 (0.5, 1) | 239.9 (155.8, 336.9) | 0.8 (0.5, 1.1) | 0.6 (0.2,1.0) |
| Deaths | Argentine Republic | 287.3 (186, 414.9) | 4.1 (2.7, 6) | 314.3 (202.9, 447.8) | 2.5 (1.6, 3.6) | -1.4 (-1.6,-1.3) |
| Deaths | Republic of Chile | 246.5 (160.7, 355.9) | 11.7 (7.6, 16.9) | 368.3 (234.5, 529.2) | 6.4 (4.1, 9.2) | -2.2 (-2.4,-2.0) |
| Deaths | Eastern Republic of Uruguay | 30.4 (19.7, 43.5) | 3.5 (2.3, 5) | 36.9 (23.2, 53.7) | 2.9 (1.8, 4.2) | -0.9 (-1.0,-0.8) |
| Deaths | Canada | 89.2 (58, 126.8) | 1.2 (0.8, 1.8) | 121.7 (77.1, 176.4) | 0.7 (0.5, 1) | -1.8 (-1.9,-1.7) |
| Deaths | United States of America | 623.4 (415, 872) | 0.9 (0.6, 1.2) | 997 (653.9, 1367.8) | 0.7 (0.5, 1) | -0.5 (-0.6,-0.4) |
| Deaths | Antigua and Barbuda | 0.1 (0.1, 0.2) | 1.1 (0.7, 1.6) | 0.2 (0.1, 0.2) | 0.8 (0.5, 1.1) | -1.7 (-2.0,-1.4) |
| Deaths | Commonwealth of the Bahamas | 0.4 (0.3, 0.6) | 1.4 (0.9, 2) | 1 (0.6, 1.4) | 1.1 (0.7, 1.6) | -0.9 (-1.1,-0.8) |
| Deaths | Barbados | 0.7 (0.4, 1) | 1.1 (0.7, 1.5) | 1.2 (0.7, 1.8) | 1 (0.6, 1.5) | -0.1 (-0.3,0.0) |
| Deaths | Belize | 0.2 (0.1, 0.3) | 1.1 (0.7, 1.7) | 0.5 (0.3, 0.7) | 0.8 (0.6, 1.2) | -1.4 (-1.8,-1.0) |
| Deaths | Republic of Cuba | 16.3 (10.5, 23.3) | 0.7 (0.5, 1.1) | 25.7 (16.5, 38) | 0.6 (0.4, 0.9) | -1.1 (-1.2,-0.9) |
| Deaths | Commonwealth of Dominica | 0.2 (0.1, 0.3) | 1.5 (0.8, 2.5) | 0.3 (0.2, 0.4) | 1.5 (0.9, 2.3) | -0.3 (-0.3,-0.2) |
| Deaths | Dominican Republic | 3.2 (1.7, 5.2) | 0.4 (0.2, 0.7) | 10.2 (5.9, 16.7) | 0.5 (0.3, 0.8) | 0.7 (0.5,0.9) |
| Deaths | Grenada | 0.1 (0.1, 0.2) | 0.9 (0.6, 1.4) | 0.2 (0.1, 0.3) | 0.9 (0.6, 1.3) | -0.2 (-0.3,-0.0) |
| Deaths | Republic of Guyana | 0.6 (0.4, 0.9) | 0.8 (0.5, 1.2) | 0.9 (0.6, 1.4) | 0.7 (0.4, 1) | -0.7 (-0.9,-0.5) |
| Deaths | Republic of Haiti | 2.9 (1.3, 5.2) | 0.4 (0.2, 0.8) | 7.1 (3.4, 12.8) | 0.5 (0.2, 0.9) | 0.5 (0.4,0.6) |
| Deaths | Jamaica | 2.9 (1.9, 4.3) | 0.7 (0.5, 1.1) | 5.3 (3.2, 7.9) | 0.8 (0.5, 1.2) | -0.1 (-0.3,0.2) |
| Deaths | Saint Lucia | 0.1 (0.1, 0.2) | 0.8 (0.5, 1.1) | 0.3 (0.2, 0.4) | 0.5 (0.3, 0.7) | -2.1 (-2.5,-1.8) |
| Deaths | Saint Vincent and the Grenadines | 0.1 (0.1, 0.1) | 0.7 (0.4, 1) | 0.1 (0.1, 0.2) | 0.5 (0.3, 0.7) | -1.5 (-1.8,-1.3) |
| Deaths | Republic of Suriname | 0.3 (0.2, 0.5) | 0.5 (0.3, 0.8) | 0.7 (0.4, 1.1) | 0.5 (0.3, 0.8) | -0.2 (-0.3,-0.0) |
| Deaths | Republic of Trinidad and Tobago | 2 (1.3, 2.9) | 1.1 (0.8, 1.6) | 3.1 (2, 4.7) | 0.7 (0.5, 1.1) | -2.0 (-2.4,-1.7) |
| Deaths | Plurinational State of Bolivia | 23.2 (11.3, 39.9) | 3.5 (1.7, 6.1) | 71.4 (37.1, 121.7) | 3.8 (2, 6.4) | 0.1 (0.0,0.2) |
| Deaths | Republic of Ecuador | 28.6 (18.5, 41.2) | 2.6 (1.7, 3.8) | 82.5 (50.6, 123.8) | 2.4 (1.5, 3.6) | -0.2 (-0.4,-0.0) |
| Deaths | Republic of Peru | 57.8 (34.2, 90.5) | 2.3 (1.4, 3.7) | 191.4 (106.8, 313.1) | 2.7 (1.5, 4.4) | -0.0 (-0.3,0.3) |
| Deaths | Republic of Colombia | 82.8 (54, 119.3) | 2.3 (1.5, 3.4) | 206.8 (131.7, 307.2) | 1.7 (1.1, 2.5) | -1.2 (-1.4,-1.0) |
| Deaths | Republic of Costa Rica | 7.8 (5.1, 11.3) | 2.2 (1.4, 3.1) | 16.5 (10.6, 24.1) | 1.4 (0.9, 2) | -2.2 (-2.5,-1.9) |
| Deaths | Republic of El Salvador | 11.5 (6.8, 17.4) | 1.8 (1.1, 2.8) | 27.3 (15.7, 43) | 2 (1.2, 3.2) | -0.1 (-0.2,0.1) |
| Deaths | Republic of Guatemala | 18.7 (12.5, 26.3) | 2.9 (1.9, 4.1) | 27.3 (17.7, 39.6) | 1.2 (0.8, 1.7) | -3.7 (-4.0,-3.4) |
| Deaths | Republic of Honduras | 5.4 (3, 9.2) | 1.3 (0.7, 2.2) | 31.2 (16.8, 51.9) | 2.4 (1.3, 4) | 2.0 (1.9,2.2) |
| Deaths | United Mexican States | 255.2 (171.7, 355.3) | 3 (2, 4.2) | 470.3 (305, 669.3) | 1.7 (1.1, 2.5) | -2.2 (-2.4,-2.0) |
| Deaths | Republic of Nicaragua | 6.3 (3.5, 10) | 2.1 (1.1, 3.2) | 17.9 (10.3, 29.9) | 1.8 (1, 2.9) | -0.5 (-0.7,-0.4) |
| Deaths | Republic of Panama | 5.5 (3.5, 7.8) | 1.8 (1.2, 2.5) | 10.1 (6.2, 14.7) | 1 (0.6, 1.5) | -1.8 (-1.9,-1.6) |
| Deaths | Bolivarian Republic of Venezuela | 48.7 (32.2, 68.8) | 2.5 (1.6, 3.5) | 84.2 (50.2, 129.5) | 1.3 (0.8, 2) | -2.4 (-2.8,-2.0) |
| Deaths | Federative Republic of Brazil | 314.1 (208.6, 439.7) | 1.8 (1.2, 2.5) | 872.6 (573.2, 1213.8) | 1.6 (1.1, 2.2) | -0.4 (-0.6,-0.3) |
| Deaths | Republic of Paraguay | 5 (2.8, 8.1) | 1.1 (0.6, 1.8) | 16.7 (9.3, 27.6) | 1.4 (0.8, 2.3) | 0.8 (0.7,0.9) |
| Deaths | People's Democratic Republic of Algeria | 45.3 (25.1, 74.6) | 2 (1.1, 3.3) | 167.4 (91.9, 277.8) | 2.5 (1.4, 4.1) | 1.0 (0.9,1.2) |
| Deaths | Kingdom of Bahrain | 0.2 (0.1, 0.3) | 0.7 (0.4, 1.2) | 1.1 (0.6, 1.9) | 0.8 (0.5, 1.4) | 0.6 (0.3,0.9) |
| Deaths | Arab Republic of Egypt | 36.1 (20.5, 65.7) | 0.7 (0.4, 1.4) | 169 (96.9, 263.4) | 1.5 (0.8, 2.3) | 3.1 (2.8,3.5) |
| Deaths | Islamic Republic of Iran | 12.3 (7.3, 19) | 0.3 (0.1, 0.4) | 97.9 (51.2, 144.2) | 0.6 (0.3, 0.9) | 4.1 (3.7,4.6) |
| Deaths | Republic of Iraq | 11.7 (6.5, 18.8) | 0.7 (0.4, 1.2) | 38.7 (21.5, 62.2) | 0.9 (0.5, 1.4) | 0.5 (0.4,0.7) |
| Deaths | Hashemite Kingdom of Jordan | 3.5 (2, 5.6) | 1.4 (0.8, 2.2) | 17.1 (9.5, 28.3) | 1.3 (0.7, 2.1) | -0.4 (-0.7,-0.1) |
| Deaths | State of Kuwait | 1.3 (0.8, 1.9) | 1.3 (0.8, 1.8) | 5.7 (3.5, 8.5) | 1.1 (0.7, 1.7) | -0.0 (-1.1,1.1) |
| Deaths | Lebanese Republic | 5.3 (2.6, 8.9) | 1.2 (0.6, 2) | 16.8 (9, 27.1) | 1.2 (0.7, 2) | 0.2 (0.1,0.4) |
| Deaths | State of Libya | 8.4 (4.5, 13.9) | 2.2 (1.2, 3.7) | 35.4 (19.7, 58.4) | 3.5 (1.9, 5.8) | 2.0 (1.8,2.2) |
| Deaths | Kingdom of Morocco | 10.4 (5.3, 17) | 0.4 (0.2, 0.6) | 35.6 (18.5, 60) | 0.5 (0.3, 0.8) | 1.3 (1.2,1.3) |
| Deaths | Palestine | 2 (1.1, 3.3) | 1.2 (0.6, 1.9) | 5.7 (3.2, 8.8) | 1.2 (0.7, 1.9) | 0.3 (0.1,0.5) |
| Deaths | Sultanate of Oman | 0.6 (0.3, 0.9) | 0.4 (0.2, 0.7) | 2.1 (1.2, 3.4) | 0.6 (0.3, 1) | 1.5 (1.3,1.7) |
| Deaths | State of Qatar | 0.2 (0.1, 0.4) | 1.6 (0.9, 2.6) | 1.7 (0.9, 3) | 1.4 (0.8, 2.4) | -0.0 (-0.6,0.6) |
| Deaths | Kingdom of Saudi Arabia | 11.5 (6.3, 20.7) | 1.1 (0.6, 1.9) | 52 (29.5, 84.6) | 1.6 (0.9, 2.7) | 1.4 (1.2,1.7) |
| Deaths | Syrian Arab Republic | 0.5 (0.3, 0.9) | 0 (0, 0.1) | 2.2 (1.2, 3.6) | 0.1 (0, 0.1) | 2.1 (1.8,2.3) |
| Deaths | Republic of Tunisia | 11.6 (6.5, 18.5) | 1.1 (0.6, 1.8) | 45.4 (24.4, 76.7) | 1.6 (0.9, 2.7) | 1.1 (1.1,1.1) |
| Deaths | Republic of Turkey | 92.8 (52.8, 148.8) | 1.3 (0.8, 2.2) | 234 (139.5, 372.2) | 1.2 (0.7, 1.9) | -0.6 (-0.8,-0.3) |
| Deaths | United Arab Emirates | 2.1 (1, 3.6) | 3.1 (1.5, 5.2) | 16.8 (9.3, 28.1) | 4.1 (2.3, 6.7) | 2.9 (2.3,3.5) |
| Deaths | Republic of Yemen | 2.9 (1.3, 5.9) | 0.3 (0.1, 0.6) | 14.6 (7.6, 25.2) | 0.5 (0.3, 0.9) | 2.2 (2.0,2.4) |
| Deaths | Islamic Republic of Afghanistan | 9.9 (3, 21.9) | 0.6 (0.2, 1.4) | 17.6 (6.7, 33.2) | 1 (0.4, 1.8) | 1.7 (1.6,1.8) |
| Deaths | People's Republic of Bangladesh | 21.4 (11.7, 36.8) | 0.2 (0.1, 0.4) | 129.5 (68.3, 238.6) | 0.4 (0.2, 0.8) | 2.4 (2.3,2.5) |
| Deaths | Kingdom of Bhutan | 0.4 (0.2, 0.6) | 0.7 (0.3, 1.3) | 1.3 (0.7, 2.3) | 1 (0.6, 1.8) | 1.2 (1.2,1.3) |
| Deaths | Republic of India | 225.4 (140.8, 346.6) | 0.2 (0.1, 0.4) | 1637.2 (954.8, 2397.7) | 0.7 (0.4, 1) | 3.5 (3.4,3.6) |
| Deaths | Federal Democratic Republic of Nepal | 4.9 (2.4, 9.1) | 0.2 (0.1, 0.5) | 25.9 (14.7, 43.5) | 0.5 (0.3, 0.9) | 2.7 (2.5,2.9) |
| Deaths | Islamic Republic of Pakistan | 94.2 (56.3, 146.7) | 0.8 (0.5, 1.3) | 366.5 (212.6, 593.8) | 1.5 (0.9, 2.5) | 2.0 (1.8,2.2) |
| Deaths | Republic of Angola | 0.6 (0.3, 1) | 0.1 (0, 0.1) | 3.4 (1.8, 5.9) | 0.1 (0.1, 0.3) | 2.3 (2.2,2.4) |
| Deaths | Central African Republic | 0.2 (0.1, 0.3) | 0.1 (0, 0.1) | 0.5 (0.2, 1) | 0.1 (0.1, 0.2) | 1.4 (1.3,1.4) |
| Deaths | Republic of the Congo | 0.3 (0.1, 0.5) | 0.1 (0.1, 0.2) | 1.1 (0.6, 1.9) | 0.2 (0.1, 0.4) | 1.6 (1.5,1.7) |
| Deaths | Democratic Republic of the Congo | 2.2 (1.2, 4.1) | 0.1 (0, 0.1) | 9.6 (4.7, 17.4) | 0.1 (0.1, 0.3) | 2.3 (2.1,2.5) |
| Deaths | Republic of Equatorial Guinea | 0 (0, 0.1) | 0.1 (0.1, 0.2) | 0.2 (0.1, 0.4) | 0.2 (0.1, 0.4) | 2.5 (2.4,2.6) |
| Deaths | Gabonese Republic | 0.3 (0.1, 0.4) | 0.2 (0.1, 0.4) | 0.6 (0.3, 1.1) | 0.3 (0.2, 0.5) | 1.1 (0.9,1.2) |
| Deaths | Republic of Burundi | 0.9 (0.4, 1.7) | 0.2 (0.1, 0.4) | 1.7 (0.8, 3) | 0.2 (0.1, 0.3) | -0.6 (-0.8,-0.4) |
| Deaths | Union of the Comoros | 0.1 (0, 0.1) | 0.2 (0.1, 0.4) | 0.4 (0.2, 0.7) | 0.4 (0.2, 0.7) | 1.7 (1.6,1.7) |
| Deaths | Republic of Djibouti | 0 (0, 0.1) | 0.1 (0.1, 0.3) | 0.2 (0.1, 0.5) | 0.2 (0.1, 0.4) | 1.1 (1.1,1.1) |
| Deaths | State of Eritrea | 0.3 (0.1, 0.5) | 0.1 (0.1, 0.3) | 1.2 (0.6, 2.3) | 0.2 (0.1, 0.4) | 1.7 (1.7,1.8) |
| Deaths | Federal Democratic Republic of Ethiopia | 15.4 (8.1, 27.6) | 0.4 (0.2, 0.7) | 28.8 (16.7, 45.7) | 0.3 (0.2, 0.5) | -0.8 (-1.1,-0.6) |
| Deaths | Republic of Kenya | 4.5 (2.7, 7.6) | 0.3 (0.2, 0.5) | 22.8 (13.3, 37) | 0.5 (0.3, 0.8) | 2.4 (2.2,2.5) |
| Deaths | Republic of Madagascar | 1.4 (0.8, 2.5) | 0.1 (0.1, 0.2) | 4.6 (2.2, 8.4) | 0.2 (0.1, 0.4) | 1.4 (1.3,1.6) |
| Deaths | Republic of Malawi | 0.4 (0.2, 0.7) | 0.1 (0, 0.1) | 1.2 (0.6, 2.1) | 0.1 (0, 0.1) | 1.1 (1.0,1.2) |
| Deaths | Republic of Mauritius | 1 (0.6, 1.3) | 0.6 (0.4, 0.9) | 1.8 (1.2, 2.5) | 0.4 (0.3, 0.6) | -2.8 (-4.3,-1.2) |
| Deaths | Republic of Mozambique | 2 (1, 3.5) | 0.2 (0.1, 0.3) | 6.8 (3.3, 12.6) | 0.3 (0.2, 0.6) | 2.4 (2.2,2.6) |
| Deaths | Republic of Rwanda | 1.6 (0.8, 2.7) | 0.3 (0.1, 0.5) | 3.8 (1.9, 6.8) | 0.3 (0.2, 0.6) | -0.2 (-0.5,0.0) |
| Deaths | Republic of Seychelles | 0.1 (0, 0.1) | 0.7 (0.4, 1.1) | 0.1 (0.1, 0.2) | 0.6 (0.3, 0.9) | -1.1 (-1.4,-0.7) |
| Deaths | Federal Republic of Somalia | 0.8 (0.4, 1.5) | 0.2 (0.1, 0.3) | 2.8 (1.3, 5.6) | 0.2 (0.1, 0.5) | 1.2 (1.1,1.2) |
| Deaths | United Republic of Tanzania | 5.7 (3, 9.5) | 0.3 (0.1, 0.4) | 21.5 (11.4, 36.6) | 0.4 (0.2, 0.7) | 1.8 (1.7,1.8) |
| Deaths | Republic of Uganda | 2.1 (1.2, 3.4) | 0.2 (0.1, 0.3) | 7.4 (4, 12.3) | 0.3 (0.1, 0.4) | 1.1 (0.9,1.3) |
| Deaths | Republic of Zambia | 1.3 (0.7, 2.1) | 0.2 (0.1, 0.4) | 5.7 (2.9, 9.9) | 0.4 (0.2, 0.8) | 2.0 (1.9,2.1) |
| Deaths | Republic of Botswana | 0.3 (0.1, 0.6) | 0.3 (0.1, 0.5) | 1.2 (0.6, 2.3) | 0.5 (0.2, 0.9) | 2.1 (1.8,2.5) |
| Deaths | Kingdom of Lesotho | 0.5 (0.2, 0.9) | 0.3 (0.1, 0.5) | 1.5 (0.8, 2.7) | 0.7 (0.4, 1.3) | 4.1 (3.6,4.6) |
| Deaths | Republic of Namibia | 0.3 (0.1, 0.4) | 0.2 (0.1, 0.4) | 1 (0.5, 1.6) | 0.4 (0.2, 0.6) | 1.7 (1.5,2.0) |
| Deaths | Republic of South Africa | 16.8 (9.5, 26.8) | 0.4 (0.2, 0.6) | 64.6 (38.1, 93.9) | 0.7 (0.4, 1) | 1.8 (1.6,2.0) |
| Deaths | Kingdom of Eswatini | 0.3 (0.1, 0.4) | 0.5 (0.3, 0.9) | 0.9 (0.5, 1.7) | 0.9 (0.5, 1.6) | 2.6 (2.1,3.0) |
| Deaths | Republic of Zimbabwe | 2.7 (1.4, 4.5) | 0.3 (0.2, 0.6) | 9.6 (5.1, 16.2) | 0.7 (0.4, 1.2) | 3.2 (2.8,3.6) |
| Deaths | Republic of Benin | 0.1 (0, 0.1) | 0 (0, 0) | 0.2 (0.1, 0.4) | 0 (0, 0) | 2.4 (1.8,3.1) |
| Deaths | Burkina Faso | 0.1 (0, 0.1) | 0 (0, 0) | 0.2 (0.1, 0.3) | 0 (0, 0) | 2.2 (1.5,2.8) |
| Deaths | Republic of Cameroon | 0.2 (0.1, 0.3) | 0 (0, 0) | 0.9 (0.4, 1.5) | 0 (0, 0.1) | 2.9 (2.3,3.5) |
| Deaths | Republic of Cabo Verde | 0 (0, 0) | 0 (0, 0.1) | 0.2 (0.1, 0.4) | 0.2 (0.1, 0.4) | 5.2 (4.5,5.9) |
| Deaths | Republic of Chad | 0.1 (0, 0.1) | 0 (0, 0) | 0.2 (0.1, 0.3) | 0 (0, 0) | 3.1 (2.6,3.7) |
| Deaths | Republic of Côte d'Ivoire | 0.1 (0.1, 0.2) | 0 (0, 0) | 0.5 (0.2, 0.8) | 0 (0, 0) | 3.2 (2.7,3.8) |
| Deaths | Republic of the Gambia | 0 (0, 0) | 0 (0, 0) | 0 (0, 0) | 0 (0, 0) | 1.1 (1.0,1.2) |
| Deaths | Republic of Ghana | 0.1 (0.1, 0.2) | 0 (0, 0) | 0.8 (0.3, 1.4) | 0 (0, 0.1) | 4.5 (3.9,5.2) |
| Deaths | Republic of Guinea | 0.1 (0, 0.1) | 0 (0, 0) | 0.2 (0.1, 0.4) | 0 (0, 0) | 2.8 (2.2,3.3) |
| Deaths | Republic of Guinea-Bissau | 0 (0, 0) | 0 (0, 0) | 0 (0, 0.1) | 0 (0, 0) | 3.0 (2.4,3.6) |
| Deaths | Republic of Liberia | 0 (0, 0.1) | 0 (0, 0) | 0.1 (0, 0.2) | 0 (0, 0.1) | 2.6 (1.9,3.3) |
| Deaths | Republic of Mali | 0.5 (0.3, 0.9) | 0.1 (0, 0.1) | 1.3 (0.7, 2.3) | 0.1 (0, 0.1) | 1.2 (0.9,1.5) |
| Deaths | Islamic Republic of Mauritania | 0 (0, 0.1) | 0 (0, 0) | 0.1 (0.1, 0.2) | 0 (0, 0.1) | 2.1 (1.5,2.8) |
| Deaths | Republic of the Niger | 0 (0, 0.1) | 0 (0, 0) | 0.2 (0.1, 0.4) | 0 (0, 0) | 2.3 (1.6,2.9) |
| Deaths | Federal Republic of Nigeria | 1.2 (0.7, 2) | 0 (0, 0) | 4.4 (2.4, 7) | 0 (0, 0) | 2.4 (2.1,2.7) |
| Deaths | Democratic Republic of Sao Tome and Principe | 0.1 (0, 0.1) | 0.6 (0.3, 1.1) | 0.2 (0.1, 0.4) | 1 (0.5, 1.7) | 1.6 (1.6,1.7) |
| Deaths | Republic of Senegal | 0.1 (0, 0.1) | 0 (0, 0) | 0.3 (0.1, 0.6) | 0 (0, 0) | 2.8 (2.2,3.5) |
| Deaths | Republic of Sierra Leone | 0 (0, 0.1) | 0 (0, 0) | 0.1 (0.1, 0.3) | 0 (0, 0) | 3.2 (2.6,3.8) |
| Deaths | Togolese Republic | 0 (0, 0) | 0 (0, 0) | 0.2 (0.1, 0.3) | 0 (0, 0) | 3.6 (3.0,4.3) |
| Deaths | American Samoa | 0 (0, 0) | 0.5 (0.3, 0.8) | 0 (0, 0.1) | 0.4 (0.3, 0.8) | -0.3 (-0.7,0.1) |
| Deaths | Bermuda | 0.1 (0.1, 0.2) | 1.1 (0.7, 1.6) | 0.2 (0.1, 0.2) | 0.5 (0.3, 0.7) | -3.0 (-3.2,-2.8) |
| Deaths | Cook Islands | 0 (0, 0) | 0.6 (0.3, 0.9) | 0 (0, 0) | 0.4 (0.2, 0.7) | -1.2 (-1.4,-1.0) |
| Deaths | Greenland | 0.2 (0.1, 0.2) | 2.6 (1.4, 4.1) | 0.2 (0.1, 0.3) | 1.2 (0.7, 2) | -2.3 (-2.5,-2.1) |
| Deaths | Guam | 0 (0, 0.1) | 0.3 (0.2, 0.5) | 0.1 (0.1, 0.1) | 0.2 (0.1, 0.3) | -1.1 (-1.3,-0.9) |
| Deaths | Principality of Monaco | 0.1 (0, 0.1) | 0.4 (0.2, 0.7) | 0.1 (0.1, 0.2) | 0.4 (0.2, 0.7) | -0.1 (-0.2,-0.0) |
| Deaths | Republic of Nauru | 0 (0, 0) | 0.7 (0.4, 1.2) | 0 (0, 0) | 0.8 (0.4, 1.3) | 0.1 (-0.0,0.2) |
| Deaths | Republic of Niue | 0 (0, 0) | 0.5 (0.2, 0.7) | 0 (0, 0) | 0.5 (0.3, 0.8) | 0.2 (0.1,0.2) |
| Deaths | Northern Mariana Islands | 0 (0, 0) | 0.3 (0.2, 0.6) | 0 (0, 0.1) | 0.4 (0.2, 0.6) | 0.9 (0.3,1.5) |
| Deaths | Republic of Palau | 0 (0, 0) | 0.3 (0.2, 0.6) | 0 (0, 0) | 0.4 (0.2, 0.6) | 0.2 (0.2,0.3) |
| Deaths | Puerto Rico | 7.2 (4.7, 10.5) | 0.9 (0.6, 1.3) | 8.5 (5.4, 12.5) | 0.5 (0.3, 0.8) | -2.4 (-2.6,-2.2) |
| Deaths | Saint Kitts and Nevis | 0.1 (0.1, 0.1) | 1.2 (0.7, 1.7) | 0.1 (0.1, 0.2) | 0.7 (0.5, 1.1) | -1.4 (-1.6,-1.3) |
| Deaths | Republic of San Marino | 0.1 (0.1, 0.2) | 1.7 (1, 2.7) | 0.2 (0.1, 0.3) | 0.9 (0.5, 1.5) | -1.2 (-1.5,-0.9) |
| Deaths | Tokelau | 0 (0, 0) | 0.4 (0.2, 0.7) | 0 (0, 0) | 0.4 (0.2, 0.7) | -0.1 (-0.2,-0.1) |
| Deaths | Tuvalu | 0 (0, 0) | 0.4 (0.2, 0.7) | 0 (0, 0) | 0.5 (0.3, 0.7) | 0.1 (0.0,0.2) |
| Deaths | United States Virgin Islands | 0.2 (0.1, 0.4) | 1.4 (0.8, 2.3) | 0.3 (0.2, 0.5) | 0.7 (0.4, 1.1) | -2.7 (-3.0,-2.5) |
| Deaths | Republic of South Sudan | 0.7 (0.4, 1.2) | 0.1 (0.1, 0.2) | 1.3 (0.7, 2.3) | 0.2 (0.1, 0.3) | 1.0 (0.8,1.1) |
| Deaths | Republic of Sudan | 8.3 (3.5, 19.1) | 0.4 (0.2, 1) | 25.7 (13.7, 44.2) | 0.7 (0.4, 1.2) | 1.6 (1.5,1.7) |

****Burden of Gallbladder and Biliary Tract Cancer Deaths Attributable to High BMI in Country 1990–2021****

| **Measure** | **Location** | **Number 1990** | **ASR 1990** | **Number 2021** | **ASR 2021** | **EAPC (95% CI)** |
| --- | --- | --- | --- | --- | --- | --- |
| DALYs (Disability-Adjusted Life Years) | China | 22356.5 (14508.7, 32292.1) | 12.3 (7.9, 17.7) | 84831.2 (48002.7, 127241.3) | 17.5 (9.9, 26.2) | 1.2 (1.1,1.2) |
| DALYs (Disability-Adjusted Life Years) | Democratic People's Republic of Korea | 289 (151.9, 524.3) | 8.4 (4.4, 15.3) | 772.3 (384.1, 1507.6) | 10.6 (5.3, 20.8) | 0.8 (0.7,0.9) |
| DALYs (Disability-Adjusted Life Years) | Taiwan (Province of China) | 688.5 (470.7, 938.6) | 19 (13, 26) | 2192.3 (1440.2, 3150.4) | 23.1 (15.2, 33.2) | -0.5 (-1.2,0.2) |
| DALYs (Disability-Adjusted Life Years) | Kingdom of Cambodia | 45.2 (22.2, 88.2) | 4.4 (2.2, 8.7) | 161.5 (78.9, 363.2) | 5.6 (2.7, 12.8) | 0.7 (0.4,0.9) |
| DALYs (Disability-Adjusted Life Years) | Republic of Indonesia | 708.3 (407.3, 1283.8) | 3.2 (1.8, 5.9) | 2612.2 (1440.6, 5615.6) | 4.6 (2.5, 10.1) | 1.3 (1.2,1.3) |
| DALYs (Disability-Adjusted Life Years) | Lao People's Democratic Republic | 23.4 (10, 44.1) | 5 (2.2, 9.5) | 58.6 (27.6, 131.6) | 5.6 (2.6, 12.7) | 0.4 (0.3,0.5) |
| DALYs (Disability-Adjusted Life Years) | Malaysia | 208.4 (106.4, 326.3) | 10.4 (5.3, 16.3) | 783 (435.6, 1225.8) | 12.3 (6.8, 19.3) | 0.2 (-0.0,0.3) |
| DALYs (Disability-Adjusted Life Years) | Republic of Maldives | 1.5 (0.6, 2.7) | 6.9 (3, 12) | 3.7 (2, 6.1) | 5 (2.7, 8.4) | -1.4 (-1.5,-1.2) |
| DALYs (Disability-Adjusted Life Years) | Republic of the Union of Myanmar | 259.8 (125, 502) | 4.9 (2.3, 9.4) | 517.4 (258, 1173.5) | 4.6 (2.3, 10.5) | -0.4 (-0.5,-0.3) |
| DALYs (Disability-Adjusted Life Years) | Republic of the Philippines | 171.2 (104.8, 285) | 2.6 (1.6, 4.5) | 817.4 (508.8, 1387.3) | 4.4 (2.7, 7.5) | 1.7 (1.6,1.8) |
| DALYs (Disability-Adjusted Life Years) | Democratic Socialist Republic of Sri Lanka | 310.6 (114.9, 520.9) | 13.4 (5, 22.5) | 384.5 (183.4, 764.1) | 6.2 (3, 12.4) | -4.1 (-4.9,-3.3) |
| DALYs (Disability-Adjusted Life Years) | Kingdom of Thailand | 2811.8 (1547.3, 4408.7) | 35.3 (19.5, 55.3) | 14928.6 (7395.9, 24240.7) | 60 (29.7, 97.4) | 1.6 (1.5,1.7) |
| DALYs (Disability-Adjusted Life Years) | Democratic Republic of Timor-Leste | 1.3 (0.7, 2.5) | 2.2 (1.1, 4.2) | 4.8 (2.3, 10.5) | 2.6 (1.2, 5.6) | 0.5 (0.2,0.8) |
| DALYs (Disability-Adjusted Life Years) | Socialist Republic of Viet Nam | 240.5 (134.7, 390) | 2.7 (1.5, 4.4) | 1160.3 (607.8, 2019.7) | 5.2 (2.7, 9) | 2.4 (2.2,2.5) |
| DALYs (Disability-Adjusted Life Years) | Republic of Fiji | 11 (6.3, 17.5) | 14.4 (8.3, 22.9) | 36.8 (20, 59.6) | 21 (11.5, 34.2) | 1.4 (1.3,1.6) |
| DALYs (Disability-Adjusted Life Years) | Republic of Kiribati | 0.7 (0.3, 1.1) | 8 (3.9, 13.4) | 1.7 (0.8, 2.9) | 10.7 (5, 18) | 1.0 (1.0,1.1) |
| DALYs (Disability-Adjusted Life Years) | Republic of the Marshall Islands | 0.4 (0.2, 0.6) | 11.4 (6, 18.9) | 1 (0.5, 1.6) | 12 (6.4, 19.8) | 0.0 (-0.1,0.1) |
| DALYs (Disability-Adjusted Life Years) | Federated States of Micronesia | 1.4 (0.7, 2.2) | 12.9 (6.8, 21.2) | 2.3 (1.2, 3.7) | 12.7 (6.9, 20.8) | -0.1 (-0.2,-0.1) |
| DALYs (Disability-Adjusted Life Years) | Independent State of Papua New Guinea | 20.1 (9.7, 34.8) | 4.8 (2.4, 8.3) | 53.7 (29.1, 92.4) | 4.6 (2.5, 7.8) | -0.3 (-0.4,-0.3) |
| DALYs (Disability-Adjusted Life Years) | Independent State of Samoa | 2.4 (1.3, 3.8) | 12.3 (6.6, 19.6) | 4.2 (2.4, 6.6) | 12.8 (7.4, 20.2) | 0.0 (-0.0,0.1) |
| DALYs (Disability-Adjusted Life Years) | Solomon Islands | 2.2 (0.9, 3.9) | 6.8 (3, 12.1) | 6 (3.2, 10.3) | 7.8 (4.2, 13.3) | 0.4 (0.2,0.5) |
| DALYs (Disability-Adjusted Life Years) | Kingdom of Tonga | 1.3 (0.7, 2) | 9.9 (5.3, 16) | 1.9 (1, 3) | 10.5 (5.9, 17) | 0.1 (0.0,0.3) |
| DALYs (Disability-Adjusted Life Years) | Republic of Vanuatu | 0.8 (0.4, 1.4) | 6 (3.1, 10.2) | 2.6 (1.5, 4.3) | 6.8 (3.8, 11.1) | 0.2 (0.1,0.3) |
| DALYs (Disability-Adjusted Life Years) | Republic of Armenia | 76.4 (48.7, 110.8) | 12.4 (7.8, 18) | 271.7 (177.7, 384.8) | 27.7 (18.1, 39.2) | 3.2 (2.4,4.0) |
| DALYs (Disability-Adjusted Life Years) | Republic of Azerbaijan | 150.7 (82.3, 249.3) | 13.1 (7.1, 21.7) | 285.2 (141.1, 485.1) | 12 (6, 20.4) | -0.5 (-0.6,-0.3) |
| DALYs (Disability-Adjusted Life Years) | Georgia | 399.6 (244.3, 603.3) | 27.5 (16.8, 41.5) | 324.1 (206, 485.9) | 24.5 (15.6, 36.7) | -1.2 (-2.4,-0.1) |
| DALYs (Disability-Adjusted Life Years) | Republic of Kazakhstan | 534.7 (339.1, 824.3) | 18.9 (12, 29.1) | 748.3 (497.1, 1064.8) | 18.5 (12.3, 26.3) | -0.5 (-0.7,-0.2) |
| DALYs (Disability-Adjusted Life Years) | Kyrgyz Republic | 111.1 (69.7, 163.3) | 16.6 (10.4, 24.4) | 206.3 (125.2, 302.7) | 19.1 (11.6, 28) | -1.8 (-3.0,-0.6) |
| DALYs (Disability-Adjusted Life Years) | Mongolia | 106.7 (54.9, 183.3) | 45.8 (23.6, 78.7) | 200.3 (116, 331.8) | 38.7 (22.4, 65) | -0.8 (-1.0,-0.6) |
| DALYs (Disability-Adjusted Life Years) | Republic of Tajikistan | 15.5 (8.2, 26.7) | 2.6 (1.4, 4.5) | 24.7 (13.4, 41.4) | 1.9 (1, 3.2) | -1.2 (-1.4,-1.1) |
| DALYs (Disability-Adjusted Life Years) | Turkmenistan | 175.2 (114.7, 251.1) | 40.7 (26.7, 58.3) | 117.9 (70.5, 183) | 12.7 (7.6, 19.7) | -4.9 (-6.1,-3.6) |
| DALYs (Disability-Adjusted Life Years) | Republic of Uzbekistan | 90 (50.8, 146.3) | 3.6 (2, 5.9) | 399.6 (253.5, 622.1) | 6.6 (4.2, 10.3) | 2.0 (1.4,2.6) |
| DALYs (Disability-Adjusted Life Years) | Republic of Albania | 86.6 (49.7, 139.1) | 20.2 (11.6, 32.4) | 183.1 (96, 311.1) | 18.4 (9.6, 31.3) | 0.0 (-0.1,0.2) |
| DALYs (Disability-Adjusted Life Years) | Bosnia and Herzegovina | 651 (374.2, 986.3) | 70.4 (40.5, 106.8) | 654 (376.6, 1174) | 46.6 (26.8, 83.5) | -1.8 (-2.1,-1.6) |
| DALYs (Disability-Adjusted Life Years) | Republic of Bulgaria | 659.6 (433.2, 939) | 23.5 (15.4, 33.5) | 674.3 (430.1, 1005.3) | 21.5 (13.7, 32.1) | -0.2 (-0.5,-0.0) |
| DALYs (Disability-Adjusted Life Years) | Republic of Croatia | 683.4 (446, 978.3) | 50.9 (33.2, 72.9) | 854.5 (548.5, 1226.4) | 42.3 (27.2, 60.7) | -0.7 (-0.8,-0.6) |
| DALYs (Disability-Adjusted Life Years) | Czech Republic | 3615.6 (2343.3, 5190.2) | 117.3 (76.1, 168.1) | 2873.6 (1797.4, 4215) | 60 (37.7, 87.8) | -2.4 (-2.5,-2.3) |
| DALYs (Disability-Adjusted Life Years) | Hungary | 3626.4 (2352.7, 5138) | 109.8 (71.1, 155.4) | 2224.2 (1444.4, 3167.4) | 53 (34.5, 75.4) | -2.5 (-2.6,-2.4) |
| DALYs (Disability-Adjusted Life Years) | North Macedonia | 171.2 (104.7, 257) | 41.5 (25.4, 62.4) | 286.4 (162.4, 448.8) | 38.9 (22, 60.9) | -0.5 (-0.7,-0.3) |
| DALYs (Disability-Adjusted Life Years) | Montenegro | 31.1 (19, 48.6) | 22.4 (13.7, 35.1) | 59.6 (34.7, 93.6) | 27.1 (15.7, 42.5) | 0.6 (0.4,0.8) |
| DALYs (Disability-Adjusted Life Years) | Republic of Poland | 7376.1 (4969.7, 10286.6) | 75.5 (50.9, 105.4) | 7554.7 (4969.6, 10625.1) | 47.4 (31.2, 66.6) | -1.8 (-1.9,-1.6) |
| DALYs (Disability-Adjusted Life Years) | Romania | 2194.9 (1453.1, 3132.2) | 34.1 (22.6, 48.7) | 2057.5 (1341.3, 2963.1) | 25.7 (16.8, 37) | -0.7 (-1.0,-0.4) |
| DALYs (Disability-Adjusted Life Years) | Republic of Serbia | 980.4 (557, 1571.2) | 39.7 (22.7, 63.8) | 1430.8 (831.8, 2270.4) | 38.9 (22.6, 61.7) | -0.4 (-0.6,-0.3) |
| DALYs (Disability-Adjusted Life Years) | Slovak Republic | 1210.6 (731, 1863.6) | 90.9 (54.7, 140) | 1420.4 (805.7, 2312.6) | 66.6 (37.8, 108.1) | -1.1 (-1.2,-1.0) |
| DALYs (Disability-Adjusted Life Years) | Republic of Slovenia | 336.7 (219.1, 483.1) | 61.4 (39.9, 87.9) | 374.1 (240.2, 539.8) | 37.8 (24.3, 54.6) | -1.8 (-2.0,-1.7) |
| DALYs (Disability-Adjusted Life Years) | Republic of Belarus | 513.4 (327.6, 747.9) | 17.4 (11.1, 25.4) | 861.2 (536.4, 1300.4) | 23.7 (14.7, 35.8) | 0.8 (0.6,1.0) |
| DALYs (Disability-Adjusted Life Years) | Republic of Estonia | 140.7 (90.6, 202.3) | 30.7 (19.8, 44.1) | 158.1 (102, 230.7) | 26.7 (17.3, 38.9) | -0.9 (-1.1,-0.6) |
| DALYs (Disability-Adjusted Life Years) | Republic of Latvia | 151.1 (96.1, 215.1) | 18.8 (12, 26.8) | 183.9 (117.6, 269.7) | 21.4 (13.7, 31.3) | 0.0 (-0.2,0.2) |
| DALYs (Disability-Adjusted Life Years) | Republic of Lithuania | 265.1 (173.5, 378.9) | 26.4 (17.3, 37.7) | 392.8 (253.2, 577.5) | 31.1 (20.1, 45.8) | -0.2 (-0.6,0.2) |
| DALYs (Disability-Adjusted Life Years) | Republic of Moldova | 234.7 (152.8, 326.6) | 23.2 (15.1, 32.4) | 227.6 (148.5, 323.6) | 17.1 (11.1, 24.3) | -0.9 (-1.2,-0.6) |
| DALYs (Disability-Adjusted Life Years) | Russian Federation | 10003.7 (6806.1, 13761.9) | 24.2 (16.4, 33.3) | 13430.3 (8845.1, 18774.8) | 25.1 (16.5, 35.1) | -0.6 (-0.9,-0.2) |
| DALYs (Disability-Adjusted Life Years) | Ukraine | 2758.7 (1615.4, 4329.3) | 16.7 (9.8, 26.2) | 3286.8 (1933.9, 5088.5) | 19.1 (11.2, 29.6) | 0.5 (0.2,0.7) |
| DALYs (Disability-Adjusted Life Years) | Brunei Darussalam | 5.2 (3, 8.5) | 25.2 (14.4, 41.1) | 19.3 (11.2, 30.4) | 24.5 (14.2, 38.9) | 0.3 (0.2,0.4) |
| DALYs (Disability-Adjusted Life Years) | Japan | 18613.5 (12949.5, 25283.4) | 49.5 (34.3, 67.3) | 25439.5 (16169.7, 35906.9) | 29.8 (19.6, 41.5) | -1.8 (-1.9,-1.7) |
| DALYs (Disability-Adjusted Life Years) | Republic of Korea | 3990.9 (2276.7, 6283.3) | 63.3 (36.3, 99.6) | 8751.8 (4854.2, 14019.8) | 41.4 (22.9, 66.4) | -1.6 (-1.7,-1.5) |
| DALYs (Disability-Adjusted Life Years) | Republic of Singapore | 53.8 (35.7, 76.3) | 11.4 (7.5, 16.2) | 173.3 (109.6, 252) | 9.1 (5.7, 13.3) | -1.0 (-1.2,-0.8) |
| DALYs (Disability-Adjusted Life Years) | Australia | 927.6 (617.3, 1299.4) | 21.7 (14.5, 30.5) | 1578.6 (1041, 2207.8) | 15.9 (10.5, 22.1) | -1.2 (-1.3,-1.1) |
| DALYs (Disability-Adjusted Life Years) | New Zealand | 172.8 (113.7, 247.9) | 20.1 (13.2, 28.9) | 462.3 (298.1, 656.5) | 24.8 (16, 35.1) | 0.5 (-0.0,0.9) |
| DALYs (Disability-Adjusted Life Years) | Principality of Andorra | 4 (2.1, 6.7) | 31.7 (16.6, 53.7) | 7.5 (4, 12.8) | 21.8 (11.7, 37.4) | -0.9 (-1.0,-0.7) |
| DALYs (Disability-Adjusted Life Years) | Republic of Austria | 1262.8 (828.5, 1810.7) | 48.1 (31.6, 68.9) | 845.2 (543.7, 1218.8) | 21.1 (13.6, 30.4) | -2.6 (-2.9,-2.4) |
| DALYs (Disability-Adjusted Life Years) | Kingdom of Belgium | 631.3 (415.4, 901.4) | 18.4 (12.1, 26.3) | 552.8 (356.5, 804.9) | 10.9 (7.1, 15.8) | -1.9 (-2.1,-1.7) |
| DALYs (Disability-Adjusted Life Years) | Republic of Cyprus | 40.9 (23.7, 65.1) | 24.5 (13.8, 39) | 74.8 (42.9, 122.9) | 16.6 (9.5, 27.4) | -1.3 (-1.4,-1.2) |
| DALYs (Disability-Adjusted Life Years) | Kingdom of Denmark | 265.2 (176.9, 373.3) | 15.5 (10.3, 21.8) | 320.2 (208, 456.9) | 12.1 (7.9, 17.2) | -1.1 (-1.2,-0.9) |
| DALYs (Disability-Adjusted Life Years) | Republic of Finland | 571.9 (377.7, 819.7) | 36.3 (24, 51.9) | 689.4 (441.1, 1000.6) | 24.7 (15.9, 35.7) | -1.5 (-1.7,-1.3) |
| DALYs (Disability-Adjusted Life Years) | French Republic | 4172.2 (2813, 5826.1) | 22.9 (15.4, 32) | 3550.9 (2243.5, 5144.5) | 11.2 (7.2, 16.3) | -2.5 (-2.5,-2.4) |
| DALYs (Disability-Adjusted Life Years) | Federal Republic of Germany | 17287.6 (11231.1, 24536.5) | 61.1 (39.7, 86.7) | 11767.8 (7548, 16868.9) | 27.9 (18, 39.8) | -2.7 (-3.1,-2.4) |
| DALYs (Disability-Adjusted Life Years) | Hellenic Republic | 711 (481.3, 1004.2) | 21 (14.2, 29.6) | 976 (629.8, 1400.1) | 19.3 (12.5, 27.6) | -0.0 (-0.7,0.6) |
| DALYs (Disability-Adjusted Life Years) | Republic of Iceland | 13.2 (8.5, 19.1) | 21.3 (13.7, 30.8) | 16.7 (10.6, 24.4) | 12.9 (8.2, 18.8) | -1.6 (-1.7,-1.5) |
| DALYs (Disability-Adjusted Life Years) | Ireland | 180.1 (117.5, 258.2) | 20 (13, 28.7) | 174.8 (109.6, 252.4) | 10 (6.3, 14.4) | -2.5 (-2.9,-2.2) |
| DALYs (Disability-Adjusted Life Years) | State of Israel | 249.3 (159.8, 359.5) | 23.3 (14.9, 33.6) | 295.4 (185.5, 431.1) | 11 (6.9, 15.9) | -2.7 (-2.8,-2.5) |
| DALYs (Disability-Adjusted Life Years) | Republic of Italy | 7630.1 (5214.6, 10612.9) | 38.4 (26.3, 53.5) | 9710.9 (6279.5, 13790.2) | 30.1 (19.7, 42.6) | -1.0 (-1.2,-0.9) |
| DALYs (Disability-Adjusted Life Years) | Grand Duchy of Luxembourg | 30.4 (19.9, 42.5) | 25.2 (16.5, 35.2) | 30.9 (20.2, 44.2) | 13.2 (8.6, 18.9) | -1.9 (-2.1,-1.8) |
| DALYs (Disability-Adjusted Life Years) | Republic of Malta | 13.5 (8.8, 19.2) | 14.4 (9.4, 20.5) | 20.5 (13, 29.7) | 9.5 (6.1, 13.8) | -1.6 (-1.7,-1.4) |
| DALYs (Disability-Adjusted Life Years) | Kingdom of the Netherlands | 1229.3 (822.7, 1737.5) | 28 (18.8, 39.6) | 1376.3 (888, 1978.4) | 17.6 (11.4, 25.3) | -1.3 (-1.5,-1.0) |
| DALYs (Disability-Adjusted Life Years) | Kingdom of Norway | 220.9 (150.9, 305.9) | 15.1 (10.3, 20.9) | 240.9 (158.4, 339.3) | 10.9 (7.2, 15.4) | -1.5 (-1.8,-1.2) |
| DALYs (Disability-Adjusted Life Years) | Portuguese Republic | 810.8 (542.2, 1157.8) | 26 (17.4, 37.2) | 975.6 (624.6, 1422.1) | 18.4 (11.8, 26.7) | -1.3 (-1.5,-1.2) |
| DALYs (Disability-Adjusted Life Years) | Kingdom of Spain | 4120.5 (2697.2, 5818.4) | 33.5 (22, 47.4) | 4368.5 (2786.3, 6362.7) | 20.4 (13.1, 29.5) | -1.9 (-2.1,-1.7) |
| DALYs (Disability-Adjusted Life Years) | Kingdom of Sweden | 1581.3 (1043.7, 2258.6) | 49.7 (32.9, 70.8) | 1449.6 (926.2, 2092.7) | 30.9 (19.8, 44.5) | -1.9 (-2.2,-1.5) |
| DALYs (Disability-Adjusted Life Years) | Swiss Confederation | 315.4 (208.3, 445.7) | 14 (9.3, 19.8) | 470.2 (301.5, 680.4) | 11.5 (7.4, 16.6) | -0.9 (-1.1,-0.6) |
| DALYs (Disability-Adjusted Life Years) | United Kingdom of Great Britain and Northern Ireland | 2993.3 (2023.6, 4156.5) | 15.2 (10.3, 21.1) | 4351.7 (2863, 6062.2) | 15.2 (10.1, 21.1) | 0.5 (0.1,0.9) |
| DALYs (Disability-Adjusted Life Years) | Argentine Republic | 6219.2 (4052.3, 8930.4) | 87.2 (56.7, 125.3) | 6841.6 (4453.4, 9700.1) | 56.8 (37.1, 80.5) | -1.2 (-1.4,-1.1) |
| DALYs (Disability-Adjusted Life Years) | Republic of Chile | 5771.1 (3772.9, 8326.6) | 264.1 (172.6, 381.1) | 7951.7 (5094.9, 11347.9) | 139.3 (89.2, 198.7) | -2.3 (-2.6,-2.1) |
| DALYs (Disability-Adjusted Life Years) | Eastern Republic of Uruguay | 645.2 (419.4, 919.1) | 74.7 (48.5, 106.4) | 717.7 (455, 1037) | 60.7 (38.6, 87.4) | -0.9 (-1.1,-0.8) |
| DALYs (Disability-Adjusted Life Years) | Canada | 1829.4 (1199.2, 2590.9) | 25.8 (16.9, 36.5) | 2201.3 (1413.7, 3152.8) | 13.6 (8.8, 19.5) | -2.0 (-2.1,-1.9) |
| DALYs (Disability-Adjusted Life Years) | United States of America | 12523.8 (8452.2, 17379.3) | 18.1 (12.3, 25.1) | 20526 (13671.7, 27815.1) | 15.9 (10.6, 21.5) | -0.5 (-0.5,-0.4) |
| DALYs (Disability-Adjusted Life Years) | Antigua and Barbuda | 2.8 (1.9, 4) | 25.6 (16.9, 36.3) | 4 (2.6, 5.5) | 16.4 (10.6, 22.9) | -1.9 (-2.1,-1.6) |
| DALYs (Disability-Adjusted Life Years) | Commonwealth of the Bahamas | 10.9 (7.1, 15.9) | 32.8 (21.3, 47.8) | 23.3 (14.6, 34.2) | 25 (15.6, 36.6) | -1.1 (-1.3,-0.9) |
| DALYs (Disability-Adjusted Life Years) | Barbados | 14.4 (9.2, 20.5) | 24 (15.4, 34.2) | 25.5 (15.8, 38.1) | 22 (13.6, 32.8) | -0.3 (-0.4,-0.1) |
| DALYs (Disability-Adjusted Life Years) | Belize | 5.2 (3.4, 7.6) | 26.3 (16.9, 38.4) | 13.1 (8.7, 18.3) | 19.7 (12.9, 27.5) | -1.4 (-1.8,-1.0) |
| DALYs (Disability-Adjusted Life Years) | Republic of Cuba | 369.8 (236.8, 526.7) | 16.7 (10.7, 23.8) | 566.7 (363.4, 843.1) | 13 (8.3, 19.4) | -1.1 (-1.3,-0.9) |
| DALYs (Disability-Adjusted Life Years) | Commonwealth of Dominica | 4.3 (2.3, 7.1) | 33.1 (18.3, 54.5) | 6.1 (3.6, 9.6) | 32.1 (18.9, 50.1) | -0.3 (-0.4,-0.2) |
| DALYs (Disability-Adjusted Life Years) | Dominican Republic | 79.9 (43.2, 128.8) | 10 (5.4, 16.1) | 240.3 (140.4, 391.8) | 11 (6.4, 18) | 0.7 (0.5,0.9) |
| DALYs (Disability-Adjusted Life Years) | Grenada | 3.2 (1.9, 4.9) | 21.7 (12.9, 33.4) | 5.3 (3.4, 7.8) | 19.9 (12.7, 29.4) | -0.3 (-0.4,-0.2) |
| DALYs (Disability-Adjusted Life Years) | Republic of Guyana | 15.5 (10, 22.6) | 18.9 (12.2, 27.7) | 23.9 (15, 35.9) | 16 (10, 23.9) | -0.7 (-0.9,-0.5) |
| DALYs (Disability-Adjusted Life Years) | Republic of Haiti | 76.8 (34.5, 138.1) | 10.5 (4.8, 18.8) | 187.9 (88.4, 339) | 11.6 (5.5, 20.9) | 0.5 (0.4,0.5) |
| DALYs (Disability-Adjusted Life Years) | Jamaica | 65.5 (41.8, 95.5) | 17.4 (11.1, 25.3) | 120.3 (73, 182.2) | 17.8 (10.8, 26.9) | -0.1 (-0.4,0.1) |
| DALYs (Disability-Adjusted Life Years) | Saint Lucia | 3.2 (2, 4.6) | 17 (10.8, 24.9) | 5.9 (3.7, 8.7) | 10.9 (6.8, 16) | -2.1 (-2.4,-1.7) |
| DALYs (Disability-Adjusted Life Years) | Saint Vincent and the Grenadines | 2.3 (1.6, 3.3) | 15.2 (10.1, 21.7) | 3.4 (2.2, 4.8) | 10.4 (6.9, 14.8) | -1.6 (-1.8,-1.3) |
| DALYs (Disability-Adjusted Life Years) | Republic of Suriname | 7.1 (4.1, 11.1) | 12.5 (7.2, 19.5) | 17 (9.5, 28.1) | 11.7 (6.5, 19.3) | -0.2 (-0.4,-0.0) |
| DALYs (Disability-Adjusted Life Years) | Republic of Trinidad and Tobago | 47.9 (31.8, 68) | 26.6 (17.6, 37.8) | 74 (45.7, 111.3) | 16.8 (10.4, 25.3) | -2.1 (-2.4,-1.7) |
| DALYs (Disability-Adjusted Life Years) | Plurinational State of Bolivia | 578.3 (280.1, 997.7) | 83 (40.4, 143.2) | 1689.1 (874.5, 2881.3) | 83.8 (43.5, 142.8) | -0.1 (-0.2,0.0) |
| DALYs (Disability-Adjusted Life Years) | Republic of Ecuador | 675.5 (439, 968.6) | 59.9 (38.9, 86.1) | 1819.8 (1118, 2719) | 51.2 (31.4, 76.5) | -0.4 (-0.6,-0.2) |
| DALYs (Disability-Adjusted Life Years) | Republic of Peru | 1397 (823.9, 2187) | 54.2 (32, 84.9) | 4368.6 (2438.5, 7138.6) | 60.2 (33.6, 98.4) | -0.1 (-0.4,0.2) |
| DALYs (Disability-Adjusted Life Years) | Republic of Colombia | 1999.2 (1307.3, 2874.5) | 52.9 (34.5, 76.1) | 4590.7 (2919.9, 6805.2) | 37.7 (24, 55.8) | -1.3 (-1.6,-1.1) |
| DALYs (Disability-Adjusted Life Years) | Republic of Costa Rica | 175.1 (114.8, 252) | 47.3 (31.1, 68.1) | 372.2 (239.4, 543.2) | 30.5 (19.6, 44.5) | -2.2 (-2.5,-1.9) |
| DALYs (Disability-Adjusted Life Years) | Republic of El Salvador | 275.4 (163.8, 415.4) | 43.1 (25.7, 65.2) | 609.2 (351.1, 955.5) | 46.3 (26.7, 72.5) | -0.1 (-0.3,0.1) |
| DALYs (Disability-Adjusted Life Years) | Republic of Guatemala | 460.6 (306.9, 646.7) | 62.3 (41.5, 87.7) | 634.6 (409.1, 914.4) | 26.7 (17.2, 38.5) | -3.6 (-3.9,-3.3) |
| DALYs (Disability-Adjusted Life Years) | Republic of Honduras | 136.6 (75, 230.3) | 30.7 (16.9, 51.8) | 762.4 (410.2, 1274.8) | 54.8 (29.5, 91.4) | 2.0 (1.8,2.1) |
| DALYs (Disability-Adjusted Life Years) | United Mexican States | 6062.5 (4072.9, 8415.5) | 66.6 (44.8, 92.6) | 11087.7 (7212.8, 15779.5) | 39.4 (25.6, 56.1) | -2.1 (-2.4,-1.9) |
| DALYs (Disability-Adjusted Life Years) | Republic of Nicaragua | 153.6 (86.1, 241.2) | 46.9 (26.3, 73.8) | 427.4 (246.4, 714) | 40.2 (23.2, 67.3) | -0.6 (-0.7,-0.5) |
| DALYs (Disability-Adjusted Life Years) | Republic of Panama | 118 (76.5, 166.5) | 37.6 (24.4, 53) | 212.5 (132.5, 308.7) | 22.1 (13.8, 32.1) | -1.8 (-1.9,-1.6) |
| DALYs (Disability-Adjusted Life Years) | Bolivarian Republic of Venezuela | 1162.6 (771.6, 1632.8) | 56.6 (37.5, 79.6) | 1934.7 (1147.6, 2994.5) | 28.8 (17.1, 44.5) | -2.6 (-3.0,-2.2) |
| DALYs (Disability-Adjusted Life Years) | Federative Republic of Brazil | 7516.6 (5022.4, 10513.8) | 39 (26, 54.6) | 19708.4 (13071, 27344.4) | 35.4 (23.5, 49.2) | -0.5 (-0.7,-0.4) |
| DALYs (Disability-Adjusted Life Years) | Republic of Paraguay | 112.8 (64.2, 183.2) | 23.9 (13.6, 38.9) | 376.8 (210.1, 622) | 29.8 (16.6, 49.1) | 0.7 (0.6,0.8) |
| DALYs (Disability-Adjusted Life Years) | People's Democratic Republic of Algeria | 1076.4 (593.9, 1769.8) | 41.6 (23, 68.3) | 3707.3 (2040.9, 6153.6) | 49.1 (27, 81.5) | 0.7 (0.6,0.9) |
| DALYs (Disability-Adjusted Life Years) | Kingdom of Bahrain | 5.4 (3.2, 8.6) | 15.3 (8.9, 24.4) | 28.8 (15.5, 48.8) | 16.1 (8.7, 26.9) | 0.2 (-0.0,0.5) |
| DALYs (Disability-Adjusted Life Years) | Arab Republic of Egypt | 935.5 (535.5, 1626.3) | 15.9 (9, 28.9) | 4216.7 (2415.2, 6584.1) | 30.8 (17.6, 48.1) | 2.9 (2.5,3.2) |
| DALYs (Disability-Adjusted Life Years) | Islamic Republic of Iran | 309.3 (184.5, 477.5) | 5.4 (3.2, 8.3) | 2171.1 (1139, 3188.8) | 13.2 (6.9, 19.4) | 4.0 (3.5,4.5) |
| DALYs (Disability-Adjusted Life Years) | Republic of Iraq | 284.7 (158.3, 455.2) | 17 (9.4, 27.2) | 939.7 (516.7, 1516.8) | 18.5 (10.3, 29.8) | 0.2 (0.1,0.3) |
| DALYs (Disability-Adjusted Life Years) | Hashemite Kingdom of Jordan | 87.9 (49.9, 140.8) | 30.7 (17.4, 49.5) | 407.8 (224.3, 673.5) | 25.8 (14.3, 42.6) | -0.7 (-1.1,-0.4) |
| DALYs (Disability-Adjusted Life Years) | State of Kuwait | 33.2 (21.1, 47.4) | 27.7 (17.5, 39.6) | 133.2 (84.1, 197.6) | 22.2 (13.9, 33.1) | -0.3 (-1.4,0.8) |
| DALYs (Disability-Adjusted Life Years) | Lebanese Republic | 123.7 (61.4, 208.2) | 26.1 (13, 43.8) | 315.8 (171.5, 505.3) | 24.2 (13.2, 38.7) | -0.1 (-0.2,0.1) |
| DALYs (Disability-Adjusted Life Years) | State of Libya | 202.8 (109.5, 334.3) | 50 (27, 82.5) | 864 (483.5, 1422.9) | 77.4 (43.2, 127.5) | 1.8 (1.7,2.0) |
| DALYs (Disability-Adjusted Life Years) | Kingdom of Morocco | 249.7 (128.5, 409.8) | 8.1 (4.2, 13.3) | 852.8 (443.2, 1442.2) | 11.1 (5.7, 18.7) | 1.2 (1.1,1.2) |
| DALYs (Disability-Adjusted Life Years) | Palestine | 45.8 (24.7, 76.1) | 24.8 (13.4, 41.2) | 133.8 (75.9, 206.3) | 25.2 (14.3, 39.1) | 0.2 (-0.0,0.4) |
| DALYs (Disability-Adjusted Life Years) | Sultanate of Oman | 14.5 (7.8, 23.9) | 10.5 (5.7, 17.2) | 52.2 (29.3, 86) | 13.1 (7.3, 21.5) | 1.2 (1.0,1.4) |
| DALYs (Disability-Adjusted Life Years) | State of Qatar | 5.9 (3.3, 9.6) | 31.5 (17.6, 51.2) | 44.3 (22.7, 80.1) | 27.6 (14.7, 47.4) | -0.1 (-0.7,0.4) |
| DALYs (Disability-Adjusted Life Years) | Kingdom of Saudi Arabia | 280.1 (152.7, 499.7) | 23.6 (12.8, 42.2) | 1406.2 (800.6, 2272.3) | 34.8 (19.8, 56.9) | 1.4 (1.1,1.6) |
| DALYs (Disability-Adjusted Life Years) | Syrian Arab Republic | 12.4 (7.1, 20.6) | 1.1 (0.6, 1.8) | 53.2 (27.3, 86.2) | 1.8 (0.9, 2.9) | 1.9 (1.6,2.1) |
| DALYs (Disability-Adjusted Life Years) | Republic of Tunisia | 276.7 (155.8, 440.6) | 24.9 (14, 39.8) | 1011.3 (541.9, 1716.6) | 34.2 (18.4, 58) | 1.0 (1.0,1.0) |
| DALYs (Disability-Adjusted Life Years) | Republic of Turkey | 2228.4 (1266.6, 3546) | 29.5 (16.7, 47.1) | 5109.5 (3064.6, 8113.1) | 24.7 (14.8, 39.2) | -0.8 (-1.0,-0.6) |
| DALYs (Disability-Adjusted Life Years) | United Arab Emirates | 54.9 (25.5, 94.9) | 69.7 (33, 119) | 454.6 (251.4, 764.1) | 77.6 (43.7, 128.3) | 2.0 (1.5,2.5) |
| DALYs (Disability-Adjusted Life Years) | Republic of Yemen | 76 (34.4, 151.9) | 7 (3.2, 14.2) | 360.3 (187.6, 620.3) | 12.1 (6.3, 20.9) | 2.0 (1.8,2.2) |
| DALYs (Disability-Adjusted Life Years) | Islamic Republic of Afghanistan | 269.2 (77.8, 595.3) | 16.1 (4.8, 35.6) | 475 (170.3, 908.6) | 24 (8.9, 45.4) | 1.5 (1.4,1.6) |
| DALYs (Disability-Adjusted Life Years) | People's Republic of Bangladesh | 540.2 (296.5, 927.5) | 5.3 (2.9, 9.2) | 3222.6 (1697.5, 5938.2) | 10.5 (5.5, 19.3) | 2.5 (2.3,2.6) |
| DALYs (Disability-Adjusted Life Years) | Kingdom of Bhutan | 9.6 (4.7, 17.2) | 17.2 (8.5, 31) | 31.4 (16.8, 55.1) | 24 (12.9, 42.2) | 1.0 (1.0,1.0) |
| DALYs (Disability-Adjusted Life Years) | Republic of India | 6011.9 (3751.6, 9197.6) | 5.8 (3.6, 8.8) | 40331.4 (23462.1, 58924.9) | 15.2 (8.8, 22.2) | 3.4 (3.3,3.5) |
| DALYs (Disability-Adjusted Life Years) | Federal Democratic Republic of Nepal | 133.7 (65.3, 250.4) | 6.2 (3, 11.5) | 666.9 (377.5, 1121.9) | 12.6 (7.1, 21.2) | 2.6 (2.4,2.8) |
| DALYs (Disability-Adjusted Life Years) | Islamic Republic of Pakistan | 2329.5 (1389.3, 3621.8) | 19 (11.3, 29.6) | 9309.5 (5373.3, 15119.1) | 35 (20.3, 56.7) | 1.9 (1.7,2.1) |
| DALYs (Disability-Adjusted Life Years) | Republic of Angola | 16.2 (8.5, 28.2) | 1.8 (1, 3.2) | 91.3 (49.6, 159.8) | 3.4 (1.9, 6) | 2.2 (2.1,2.4) |
| DALYs (Disability-Adjusted Life Years) | Central African Republic | 5.2 (2.5, 9.6) | 1.9 (1, 3.6) | 14.7 (6.9, 28.1) | 2.9 (1.4, 5.5) | 1.3 (1.3,1.4) |
| DALYs (Disability-Adjusted Life Years) | Republic of the Congo | 7.9 (3.9, 14) | 3.2 (1.6, 5.7) | 30.6 (16.6, 50.7) | 5.1 (2.8, 8.5) | 1.5 (1.4,1.5) |
| DALYs (Disability-Adjusted Life Years) | Democratic Republic of the Congo | 59.2 (30.7, 108.1) | 1.7 (0.9, 3) | 249.7 (122.9, 447.9) | 3.1 (1.5, 5.7) | 2.2 (2.0,2.4) |
| DALYs (Disability-Adjusted Life Years) | Republic of Equatorial Guinea | 1.3 (0.6, 2.3) | 2.8 (1.4, 5.1) | 5.5 (2.7, 10.2) | 5.2 (2.5, 9.6) | 2.1 (2.0,2.2) |
| DALYs (Disability-Adjusted Life Years) | Gabonese Republic | 6.4 (3.4, 10.9) | 5 (2.6, 8.5) | 15.7 (8.3, 26.9) | 6.8 (3.6, 11.6) | 0.9 (0.7,1.0) |
| DALYs (Disability-Adjusted Life Years) | Republic of Burundi | 22.4 (10.4, 41.5) | 4.5 (2.1, 8.3) | 42.7 (21, 77.5) | 4.1 (2, 7.3) | -0.8 (-1.0,-0.6) |
| DALYs (Disability-Adjusted Life Years) | Union of the Comoros | 2.1 (1.1, 3.7) | 4.9 (2.5, 8.7) | 8.6 (4.5, 15.6) | 8.2 (4.2, 14.8) | 1.6 (1.5,1.6) |
| DALYs (Disability-Adjusted Life Years) | Republic of Djibouti | 1 (0.5, 1.7) | 3.3 (1.7, 5.9) | 6.4 (3.1, 12.5) | 4.6 (2.3, 8.9) | 1.0 (1.0,1.1) |
| DALYs (Disability-Adjusted Life Years) | State of Eritrea | 8.2 (4.1, 15.3) | 3.2 (1.6, 6.1) | 31.8 (15.6, 60.6) | 5.3 (2.6, 10.1) | 1.6 (1.6,1.6) |
| DALYs (Disability-Adjusted Life Years) | Federal Democratic Republic of Ethiopia | 411.6 (213, 744.5) | 9.3 (4.9, 16.7) | 695.5 (404.2, 1103.9) | 7.7 (4.4, 12.1) | -1.1 (-1.3,-0.9) |
| DALYs (Disability-Adjusted Life Years) | Republic of Kenya | 113 (67.4, 191.8) | 6.4 (3.8, 10.8) | 573.8 (333.3, 931.5) | 11.5 (6.7, 18.8) | 2.3 (2.1,2.5) |
| DALYs (Disability-Adjusted Life Years) | Republic of Madagascar | 36 (19.4, 61.2) | 3.3 (1.8, 5.5) | 120.8 (59.2, 219.8) | 5 (2.4, 9) | 1.4 (1.2,1.5) |
| DALYs (Disability-Adjusted Life Years) | Republic of Malawi | 11.2 (6.1, 18.9) | 1.3 (0.7, 2.2) | 30.5 (15.4, 54.4) | 1.9 (1, 3.4) | 1.1 (1.0,1.2) |
| DALYs (Disability-Adjusted Life Years) | Republic of Mauritius | 23.5 (15.8, 32.8) | 14.6 (9.8, 20.4) | 41.7 (27.5, 59.5) | 9.7 (6.4, 13.9) | -2.9 (-4.4,-1.3) |
| DALYs (Disability-Adjusted Life Years) | Republic of Mozambique | 51.9 (26.5, 90.7) | 3.9 (2, 6.9) | 177.1 (84.2, 326) | 7.3 (3.5, 13.5) | 2.4 (2.2,2.6) |
| DALYs (Disability-Adjusted Life Years) | Republic of Rwanda | 40.6 (20.8, 70.5) | 6.4 (3.3, 11.1) | 93.9 (47.2, 166.8) | 6.9 (3.5, 12.3) | -0.5 (-0.8,-0.2) |
| DALYs (Disability-Adjusted Life Years) | Republic of Seychelles | 2 (1.1, 3.2) | 16.6 (9.1, 26) | 3.4 (2, 5.5) | 12.8 (7.4, 20.7) | -1.3 (-1.7,-0.9) |
| DALYs (Disability-Adjusted Life Years) | Federal Republic of Somalia | 22.4 (11, 42.4) | 4.2 (2.1, 7.9) | 76.7 (36, 153) | 5.9 (2.8, 11.7) | 1.1 (1.1,1.2) |
| DALYs (Disability-Adjusted Life Years) | United Republic of Tanzania | 143 (76.1, 240.2) | 6 (3.2, 10) | 522.7 (276.5, 888.1) | 9.6 (5.1, 16.4) | 1.6 (1.6,1.7) |
| DALYs (Disability-Adjusted Life Years) | Republic of Uganda | 52.6 (29.2, 84.5) | 3.8 (2.1, 6) | 185.9 (98.5, 312.1) | 5.9 (3.1, 9.8) | 1.0 (0.7,1.2) |
| DALYs (Disability-Adjusted Life Years) | Republic of Zambia | 33 (18.6, 54) | 5.3 (3, 8.7) | 146.2 (75, 251.1) | 10 (5.1, 17.2) | 1.9 (1.8,2.0) |
| DALYs (Disability-Adjusted Life Years) | Republic of Botswana | 7.5 (3.5, 14.2) | 6.2 (2.9, 11.8) | 29.2 (14.3, 54.3) | 9.7 (4.8, 17.9) | 1.9 (1.6,2.3) |
| DALYs (Disability-Adjusted Life Years) | Kingdom of Lesotho | 11.2 (5.5, 21.8) | 6.1 (3, 11.9) | 37.5 (19.1, 67.1) | 16 (8.2, 28.7) | 4.2 (3.6,4.7) |
| DALYs (Disability-Adjusted Life Years) | Republic of Namibia | 6.3 (3.4, 10.7) | 4.5 (2.4, 7.8) | 22.5 (11.6, 38.3) | 7.8 (4, 13.2) | 1.6 (1.3,1.8) |
| DALYs (Disability-Adjusted Life Years) | Republic of South Africa | 396.9 (225.8, 627) | 9 (5.1, 14.3) | 1536.2 (909.6, 2217) | 15.2 (9, 22) | 1.8 (1.6,2.0) |
| DALYs (Disability-Adjusted Life Years) | Kingdom of Eswatini | 6 (3.2, 10.4) | 10.3 (5.4, 17.9) | 23.1 (11.5, 40.7) | 20 (10, 35.1) | 2.6 (2.1,3.0) |
| DALYs (Disability-Adjusted Life Years) | Republic of Zimbabwe | 67.4 (35.9, 111.7) | 7.6 (4, 12.6) | 249.1 (130.9, 419.3) | 16.5 (8.7, 27.8) | 3.3 (2.8,3.9) |
| DALYs (Disability-Adjusted Life Years) | Republic of Benin | 1.3 (0.7, 2.3) | 0.3 (0.2, 0.5) | 4.3 (2, 7.6) | 0.4 (0.2, 0.8) | 1.7 (1.1,2.4) |
| DALYs (Disability-Adjusted Life Years) | Burkina Faso | 1.5 (0.8, 2.4) | 0.2 (0.1, 0.3) | 4 (2, 6.9) | 0.2 (0.1, 0.4) | 1.7 (1.0,2.3) |
| DALYs (Disability-Adjusted Life Years) | Republic of Cameroon | 4.7 (2.7, 7.7) | 0.5 (0.3, 0.8) | 18.6 (8.6, 33) | 0.8 (0.3, 1.4) | 2.3 (1.7,2.9) |
| DALYs (Disability-Adjusted Life Years) | Republic of Cabo Verde | 0.5 (0.2, 1) | 1 (0.5, 2) | 4.2 (1.4, 7.6) | 4.5 (1.5, 8.2) | 4.9 (4.2,5.6) |
| DALYs (Disability-Adjusted Life Years) | Republic of Chad | 1.2 (0.6, 2.1) | 0.2 (0.1, 0.4) | 3.9 (1.8, 7.1) | 0.3 (0.2, 0.6) | 2.6 (2.0,3.2) |
| DALYs (Disability-Adjusted Life Years) | Republic of Côte d'Ivoire | 2.4 (1.4, 4) | 0.3 (0.2, 0.5) | 10.4 (4.7, 18.4) | 0.5 (0.2, 0.9) | 2.6 (2.0,3.2) |
| DALYs (Disability-Adjusted Life Years) | Republic of the Gambia | 0 (0, 0) | 0 (0, 0) | 0 (0, 0) | 0 (0, 0) | 1.0 (0.8,1.2) |
| DALYs (Disability-Adjusted Life Years) | Republic of Ghana | 2.8 (1.5, 4.7) | 0.2 (0.1, 0.4) | 16.7 (7.8, 29.9) | 0.5 (0.2, 0.9) | 3.8 (3.1,4.5) |
| DALYs (Disability-Adjusted Life Years) | Republic of Guinea | 1.7 (0.9, 3.1) | 0.2 (0.1, 0.4) | 4.2 (2, 7.7) | 0.4 (0.2, 0.7) | 2.2 (1.6,2.8) |
| DALYs (Disability-Adjusted Life Years) | Republic of Guinea-Bissau | 0.3 (0.1, 0.5) | 0.3 (0.2, 0.6) | 0.7 (0.4, 1.3) | 0.5 (0.3, 1) | 2.4 (1.8,3.0) |
| DALYs (Disability-Adjusted Life Years) | Republic of Liberia | 0.9 (0.5, 1.6) | 0.4 (0.2, 0.6) | 2.2 (1, 4.1) | 0.6 (0.3, 1) | 2.1 (1.3,2.8) |
| DALYs (Disability-Adjusted Life Years) | Republic of Mali | 13 (7.3, 21.7) | 1.5 (0.8, 2.5) | 33.4 (17.5, 58.9) | 1.7 (0.9, 3.1) | 1.1 (0.8,1.4) |
| DALYs (Disability-Adjusted Life Years) | Islamic Republic of Mauritania | 1 (0.5, 1.6) | 0.4 (0.2, 0.7) | 2.6 (1.2, 4.6) | 0.6 (0.3, 1.1) | 1.6 (0.9,2.3) |
| DALYs (Disability-Adjusted Life Years) | Republic of the Niger | 1.2 (0.6, 2) | 0.2 (0.1, 0.3) | 4.5 (2, 8.6) | 0.3 (0.1, 0.5) | 1.7 (1.1,2.4) |
| DALYs (Disability-Adjusted Life Years) | Federal Republic of Nigeria | 28.1 (16.1, 45.1) | 0.3 (0.2, 0.5) | 105.6 (55.8, 170.1) | 0.5 (0.3, 0.9) | 2.4 (2.1,2.6) |
| DALYs (Disability-Adjusted Life Years) | Democratic Republic of Sao Tome and Principe | 2.1 (1.1, 3.5) | 14.2 (7.3, 24) | 5.2 (2.6, 9) | 22.2 (11.2, 38.1) | 1.5 (1.4,1.6) |
| DALYs (Disability-Adjusted Life Years) | Republic of Senegal | 1.9 (1, 3) | 0.3 (0.2, 0.4) | 6.7 (3.2, 12.5) | 0.4 (0.2, 0.8) | 2.3 (1.6,3.0) |
| DALYs (Disability-Adjusted Life Years) | Republic of Sierra Leone | 1 (0.5, 1.7) | 0.2 (0.1, 0.4) | 2.9 (1.3, 5.3) | 0.4 (0.2, 0.7) | 2.7 (2.1,3.3) |
| DALYs (Disability-Adjusted Life Years) | Togolese Republic | 0.7 (0.4, 1.1) | 0.3 (0.1, 0.4) | 3.6 (1.7, 6.6) | 0.5 (0.2, 0.9) | 3.0 (2.3,3.7) |
| DALYs (Disability-Adjusted Life Years) | American Samoa | 0.5 (0.3, 0.9) | 11.3 (6.3, 18) | 1.1 (0.7, 1.9) | 10.2 (5.9, 17) | -0.3 (-0.6,0.1) |
| DALYs (Disability-Adjusted Life Years) | Bermuda | 3.1 (1.9, 4.7) | 23.1 (14, 34.7) | 3.1 (1.9, 4.6) | 10.2 (6.5, 15.2) | -3.1 (-3.4,-2.9) |
| DALYs (Disability-Adjusted Life Years) | Cook Islands | 0.3 (0.2, 0.6) | 12.6 (6.5, 20.7) | 0.6 (0.3, 1) | 9.3 (5, 15.9) | -1.2 (-1.4,-1.0) |
| DALYs (Disability-Adjusted Life Years) | Greenland | 3.9 (2.1, 6.1) | 56.1 (30.2, 87.7) | 4.3 (2.4, 7) | 26 (14.4, 42.3) | -2.4 (-2.6,-2.2) |
| DALYs (Disability-Adjusted Life Years) | Guam | 1.1 (0.7, 1.7) | 6.6 (4, 10.1) | 2.3 (1.4, 3.5) | 4.7 (2.8, 7.3) | -0.7 (-0.8,-0.5) |
| DALYs (Disability-Adjusted Life Years) | Principality of Monaco | 1.4 (0.7, 2.3) | 9.1 (4.9, 14.9) | 1.8 (1, 3) | 8.5 (4.7, 13.9) | -0.2 (-0.3,-0.1) |
| DALYs (Disability-Adjusted Life Years) | Republic of Nauru | 0.2 (0.1, 0.3) | 17.8 (8.6, 30.4) | 0.2 (0.1, 0.4) | 18.7 (9.1, 31.5) | 0.0 (-0.1,0.2) |
| DALYs (Disability-Adjusted Life Years) | Republic of Niue | 0 (0, 0.1) | 10.4 (5.6, 16.8) | 0.1 (0, 0.1) | 11.3 (6.3, 17.8) | 0.1 (-0.0,0.1) |
| DALYs (Disability-Adjusted Life Years) | Northern Mariana Islands | 0.2 (0.1, 0.5) | 6.8 (3.6, 13.5) | 1 (0.6, 1.8) | 7.9 (4.4, 14.2) | 0.9 (0.3,1.5) |
| DALYs (Disability-Adjusted Life Years) | Republic of Palau | 0.2 (0.1, 0.3) | 8.1 (4.3, 14.1) | 0.5 (0.2, 0.8) | 8.6 (4.4, 14.9) | 0.1 (0.1,0.2) |
| DALYs (Disability-Adjusted Life Years) | Puerto Rico | 157.7 (102.4, 227) | 20.1 (13, 28.9) | 171.6 (108.7, 250) | 11.7 (7.4, 17) | -2.3 (-2.5,-2.1) |
| DALYs (Disability-Adjusted Life Years) | Saint Kitts and Nevis | 2.2 (1.3, 3.2) | 26.6 (16.4, 39.5) | 2.6 (1.6, 3.7) | 16.1 (10.2, 23.3) | -1.6 (-1.8,-1.4) |
| DALYs (Disability-Adjusted Life Years) | Republic of San Marino | 2.6 (1.5, 4.1) | 32.9 (19.3, 51.7) | 2.9 (1.5, 5) | 17.7 (8.9, 31) | -1.2 (-1.5,-0.9) |
| DALYs (Disability-Adjusted Life Years) | Tokelau | 0 (0, 0.1) | 10.6 (5.7, 17.2) | 0 (0, 0.1) | 10.2 (5.6, 17) | -0.2 (-0.3,-0.2) |
| DALYs (Disability-Adjusted Life Years) | Tuvalu | 0.2 (0.1, 0.3) | 10.6 (5.7, 17.9) | 0.3 (0.1, 0.4) | 11 (6.1, 17.4) | 0.0 (-0.1,0.1) |
| DALYs (Disability-Adjusted Life Years) | United States Virgin Islands | 5.8 (3.1, 9.2) | 31.8 (17, 50.4) | 6 (3.3, 9.9) | 14.5 (8.1, 24.2) | -2.9 (-3.1,-2.6) |
| DALYs (Disability-Adjusted Life Years) | Republic of South Sudan | 16.5 (8.6, 28) | 3 (1.6, 5.1) | 35 (18.1, 61.8) | 4.1 (2.1, 7.2) | 1.0 (0.8,1.2) |
| DALYs (Disability-Adjusted Life Years) | Republic of Sudan | 208 (87.7, 475) | 10.3 (4.4, 23.6) | 635.8 (334.1, 1099.9) | 15.2 (8.1, 26.2) | 1.4 (1.3,1.5) |
