# Supplementary material for: Attributable burden of high BMI-related gastrointestinal tract cancers among middle-aged and elderly populations globally, 1990–2021 and projected to 2050: analysis of GBD 2021
Source: Front Nutr. 2026 Jan 2;12:1674621. doi: 10.3389/fnut.2025.1674621 (PMC12807987; doi:10.3389/fnut.2025.1674621)
Supplement: Supplementary file 4 [file Table_3.docx]

****Global and SDI-Based Trends in High BMI-Attributable** Colon and rectum cancer **Burden, 1990–2021****

| **Measure** | **Location** | **Number 1990** | **ASMR 1990** | **Number 2021** | **ASMR 2021** | **EAPC (95% CI)** |
| --- | --- | --- | --- | --- | --- | --- |
| Deaths | Global | 38385 (16174.6, 62103.4) | 4.9 (2.1, 8) | 92012.9 (39255.8, 147266.7) | 5 (2.1, 8) | -0.1 (-0.1,-0.0) |
| Deaths | Low SDI | 374.6 (134.4, 629.5) | 0.8 (0.3, 1.4) | 1290.7 (495.4, 2093.1) | 1.3 (0.5, 2.1) | 1.5 (1.4,1.6) |
| Deaths | Low-middle SDI | 1016.4 (382.8, 1645.5) | 0.8 (0.3, 1.4) | 5421.9 (2255.8, 8636) | 1.8 (0.8, 2.9) | 2.8 (2.7,2.8) |
| Deaths | Middle SDI | 3485.2 (1295.1, 5706.2) | 1.7 (0.6, 2.8) | 19122.6 (8142.5, 30802.8) | 3.4 (1.4, 5.4) | 2.2 (2.1,2.2) |
| Deaths | High-middle SDI | 12640.2 (5374.8, 20405.3) | 6.1 (2.6, 9.9) | 31036.7 (13310.4, 50090.6) | 7.1 (3, 11.5) | 0.4 (0.3,0.5) |
| Deaths | High SDI | 20794.1 (8752.9, 33711.1) | 8.5 (3.6, 13.8) | 34989.2 (14842.1, 56009.5) | 7.1 (3, 11.3) | -0.7 (-0.8,-0.7) |

****Global and SDI-Based Trends in High BMI-Attributable** Colon and rectum cancer **Burden, 2000–2021****

****Global and SDI-Based Trends in High BMI-Attributable** Colon and rectum cancer **Burden, 2010–2021****

| **Measure** | **Location** | **Number 2000** | **ASMR 2000** | **Number 2021** | **ASMR 2021** | **EAPC (95% CI)** |
| --- | --- | --- | --- | --- | --- | --- |
| Deaths | Global | 50288.3 (21418.8, 81005.2) | 5.1 (2.1, 8.2) | 92012.9 (39255.8, 147266.7) | 5 (2.1, 8) | -0.1 (-0.1,-0.0) |
| Deaths | Low SDI | 498.5 (179.5, 823.1) | 0.9 (0.3, 1.5) | 1290.7 (495.4, 2093.1) | 1.3 (0.5, 2.1) | 1.5 (1.4,1.6) |
| Deaths | Low-middle SDI | 1669.4 (654.3, 2685.3) | 1.1 (0.4, 1.7) | 5421.9 (2255.8, 8636) | 1.8 (0.8, 2.9) | 2.8 (2.7,2.8) |
| Deaths | Middle SDI | 5661.1 (2239.4, 9164.2) | 2.1 (0.8, 3.5) | 19122.6 (8142.5, 30802.8) | 3.4 (1.4, 5.4) | 2.2 (2.1,2.2) |
| Deaths | High-middle SDI | 17471.5 (7522.9, 28002.8) | 6.9 (3, 11) | 31036.7 (13310.4, 50090.6) | 7.1 (3, 11.5) | 0.4 (0.3,0.5) |
| Deaths | High SDI | 24891.9 (10535, 40221) | 8.4 (3.5, 13.5) | 34989.2 (14842.1, 56009.5) | 7.1 (3, 11.3) | -0.7 (-0.8,-0.7) |

| **Measure** | **Location** | **Number 2010** | **ASMR 2010** | **Number 2021** | **ASMR 2021** | **EAPC (95% CI)** |
| --- | --- | --- | --- | --- | --- | --- |
| Deaths | Global | 65038.7 (27539, 104627.4) | 4.9 (2.1, 7.9) | 92012.9 (39255.8, 147266.7) | 5 (2.1, 8) | -0.1 (-0.1,-0.0) |
| Deaths | Low SDI | 727.7 (277.9, 1174.8) | 1 (0.4, 1.6) | 1290.7 (495.4, 2093.1) | 1.3 (0.5, 2.1) | 1.5 (1.4,1.6) |
| Deaths | Low-middle SDI | 3061.2 (1227.2, 4895.6) | 1.5 (0.6, 2.3) | 5421.9 (2255.8, 8636) | 1.8 (0.8, 2.9) | 2.8 (2.7,2.8) |
| Deaths | Middle SDI | 9982.5 (4049.2, 16032.4) | 2.7 (1.1, 4.3) | 19122.6 (8142.5, 30802.8) | 3.4 (1.4, 5.4) | 2.2 (2.1,2.2) |
| Deaths | High-middle SDI | 22704.6 (9724.1, 36595.3) | 7 (3, 11.4) | 31036.7 (13310.4, 50090.6) | 7.1 (3, 11.5) | 0.4 (0.3,0.5) |
| Deaths | High SDI | 28438.6 (12078.6, 45831.2) | 7.5 (3.2, 12.1) | 34989.2 (14842.1, 56009.5) | 7.1 (3, 11.3) | -0.7 (-0.8,-0.7) |

| **Measure** | **Location** | **Number 1990** | **ASR 1990** | **Number 2021** | **ASR 2021** | **EAPC (95% CI)** |
| --- | --- | --- | --- | --- | --- | --- |
| DALYs (Disability-Adjusted Life Years) | Global | 859376.6 (363038.2, 1385148.2) | 101.8 (42.9, 164.3) | 2005125.3 (858739.6, 3189675.9) | 105.5 (45.1, 167.9) | 0.0 (-0.0,0.1) |
| DALYs (Disability-Adjusted Life Years) | Low SDI | 10101.5 (3678.8, 17015) | 19.7 (7.1, 33.2) | 33407.1 (12953.2, 54094.9) | 30.1 (11.6, 48.8) | 1.3 (1.2,1.4) |
| DALYs (Disability-Adjusted Life Years) | Low-middle SDI | 26316 (9985, 42574.9) | 19.6 (7.4, 31.7) | 136599.8 (57045.5, 216731.6) | 42.8 (17.9, 68) | 2.8 (2.7,2.8) |
| DALYs (Disability-Adjusted Life Years) | Middle SDI | 87961.4 (32871.1, 143972.6) | 39 (14.5, 63.8) | 459064.4 (195169.7, 736565) | 75.5 (32.1, 121.3) | 2.2 (2.1,2.2) |
| DALYs (Disability-Adjusted Life Years) | High-middle SDI | 295098.6 (125414.1, 475751) | 133.7 (56.7, 215.7) | 673847.7 (290683.2, 1080376.7) | 151.8 (65.4, 243.5) | 0.3 (0.2,0.4) |
| DALYs (Disability-Adjusted Life Years) | High SDI | 438221.7 (185893.6, 707607.1) | 181.3 (76.9, 292.7) | 699012.8 (300250.2, 1110307.2) | 154.6 (66.7, 244.9) | -0.6 (-0.7,-0.6) |

****Global and SDI-Based Trends in High BMI-Attributable** Colon and rectum cancer **Burden, 1990–2021****

****Global and SDI-Based Trends in High BMI-Attributable** Colon and rectum cancer **Burden, 1990–2021****

| **Measure** | **Location** | **Number 1990** | **ASR 1990** | **Number 2021** | **ASR 2021** | **EAPC (95% CI)** |
| --- | --- | --- | --- | --- | --- | --- |
| YLDs (Years Lived with Disability) | Global | 30543.6 (12609.5, 51466.7) | 3.7 (1.5, 6.2) | 97294.1 (41186.4, 162335.7) | 5.1 (2.2, 8.6) | 1.0 (1.0,1.1) |
| YLDs (Years Lived with Disability) | Low SDI | 145.2 (51.3, 255.3) | 0.3 (0.1, 0.5) | 560.6 (213.5, 960.3) | 0.5 (0.2, 0.9) | 1.8 (1.6,1.9) |
| YLDs (Years Lived with Disability) | Low-middle SDI | 450.7 (172.8, 770.7) | 0.3 (0.1, 0.6) | 3024.9 (1237.4, 5124.2) | 1 (0.4, 1.6) | 3.6 (3.5,3.6) |
| YLDs (Years Lived with Disability) | Middle SDI | 1785.3 (667.4, 3076.4) | 0.8 (0.3, 1.4) | 16655.9 (6749.7, 27916.3) | 2.7 (1.1, 4.6) | 4.2 (4.1,4.2) |
| YLDs (Years Lived with Disability) | High-middle SDI | 8131 (3398.1, 13631) | 3.7 (1.6, 6.2) | 31003.2 (13046.7, 52527.1) | 7 (2.9, 11.8) | 2.1 (2.0,2.1) |
| YLDs (Years Lived with Disability) | High SDI | 19987.3 (8292.6, 33535) | 8.2 (3.4, 13.7) | 45920 (19578.6, 76198.4) | 10.1 (4.3, 16.7) | 0.6 (0.5,0.7) |

| **Measure** | **Location** | **Number 1990** | **ASR 1990** | **Number 2021** | **ASR 2021** | **EAPC (95% CI)** |
| --- | --- | --- | --- | --- | --- | --- |
| YLLs (Years of Life Lost) | Global | 828833 (350245.9, 1336831) | 98.1 (41.4, 158.5) | 1907831.2 (819525.4, 3035602.5) | 100.3 (43, 159.8) | -0.0 (-0.1,0.0) |
| YLLs (Years of Life Lost) | Low SDI | 9956.3 (3626.6, 16770.2) | 19.4 (7, 32.7) | 32846.5 (12723.6, 53153.2) | 29.6 (11.4, 48) | 1.3 (1.2,1.4) |
| YLLs (Years of Life Lost) | Low-middle SDI | 25865.3 (9796.4, 41831.6) | 19.2 (7.3, 31.1) | 133574.9 (55657.3, 211950.9) | 41.8 (17.4, 66.5) | 2.8 (2.7,2.8) |
| YLLs (Years of Life Lost) | Middle SDI | 86176.1 (32098.5, 141063.9) | 38.2 (14.2, 62.5) | 442408.5 (188646.4, 710628.8) | 72.8 (31, 117) | 2.1 (2.1,2.1) |
| YLLs (Years of Life Lost) | High-middle SDI | 286967.6 (122209.5, 463004.7) | 130 (55.3, 209.9) | 642844.5 (277543.2, 1032095.5) | 144.8 (62.5, 232.7) | 0.2 (0.1,0.3) |
| YLLs (Years of Life Lost) | High SDI | 418234.4 (177225.4, 675840.7) | 173.1 (73.3, 279.7) | 653092.9 (281560.5, 1037062.8) | 144.5 (62.6, 228.8) | -0.7 (-0.8,-0.7) |

****Global and SDI-Based Trends in High BMI-Attributable** Colon and rectum cancer **Burden, 1990–2021****

****Burden of** Colon and rectum cancer **Deaths Attributable to High BMI in 21 GBD Regions, 1990–2021****

| **Measure** | **Location** | **Number 1990** | **ASMR 1990** | **Number 2021** | **ASMR 2021** | **EAPC (95% CI)** |
| --- | --- | --- | --- | --- | --- | --- |
| Deaths | Global | 38385 (16174.6, 62103.4) | 4.9 (2.1, 8) | 92012.9 (39255.8, 147266.7) | 5 (2.1, 8) | -0.1 (-0.1,-0.0) |
| Deaths | Andean Latin America | 115.6 (46.1, 194.3) | 2.8 (1.1, 4.7) | 603 (256.3, 1025.5) | 4.8 (2, 8.2) | 1.8 (1.7,2.0) |
| Deaths | Australasia | 528.5 (217.1, 852.9) | 10.4 (4.3, 16.7) | 1076.1 (454.5, 1739.4) | 8.6 (3.6, 13.8) | -0.8 (-0.9,-0.8) |
| Deaths | Caribbean | 217.1 (88.8, 347.6) | 4 (1.6, 6.4) | 778.4 (325, 1281.6) | 6.5 (2.7, 10.8) | 1.7 (1.7,1.8) |
| Deaths | Central Asia | 432.1 (178.4, 698.2) | 4.3 (1.8, 6.9) | 685.7 (293.1, 1095.5) | 4 (1.7, 6.5) | 0.2 (0.1,0.4) |
| Deaths | Central Europe | 3428 (1480.8, 5547.7) | 10.6 (4.6, 17.1) | 6739.8 (2947.3, 10854.5) | 13.2 (5.8, 21.3) | 0.6 (0.5,0.7) |
| Deaths | Central Latin America | 449.5 (187.4, 726.9) | 2.8 (1.1, 4.5) | 2757.4 (1210.9, 4443.4) | 5.1 (2.3, 8.3) | 2.0 (2.0,2.1) |
| Deaths | Central Sub-Saharan Africa | 44.4 (15.6, 78.3) | 1 (0.3, 1.8) | 215.9 (80.1, 384.7) | 2.1 (0.8, 3.7) | 2.4 (2.2,2.6) |
| Deaths | East Asia | 3081.8 (1053.6, 5225.9) | 1.8 (0.6, 3.1) | 18405.5 (7530.2, 30739.3) | 3.9 (1.6, 6.5) | 2.5 (2.4,2.5) |
| Deaths | Eastern Europe | 5062.2 (2175, 8080.4) | 8.1 (3.5, 13) | 8688.6 (3730.3, 13841.1) | 11 (4.7, 17.4) | 0.8 (0.7,0.9) |
| Deaths | Eastern Sub-Saharan Africa | 176.9 (61.2, 298.4) | 1.2 (0.4, 1.9) | 644.4 (239.7, 1071.8) | 2 (0.7, 3.3) | 1.7 (1.6,1.8) |
| Deaths | High-income Asia Pacific | 1301.8 (476.6, 2134.2) | 3.1 (1.1, 5) | 4121.3 (1558.7, 6757.6) | 3.5 (1.4, 5.8) | 0.4 (0.3,0.4) |
| Deaths | High-income North America | 7656.1 (3270.1, 12354.4) | 9.8 (4.2, 15.7) | 12047 (5329.7, 18866.2) | 8.1 (3.6, 12.6) | -0.8 (-0.9,-0.7) |
| Deaths | North Africa and Middle East | 1115.9 (462.1, 1816.6) | 3.4 (1.4, 5.5) | 4863.1 (2085.2, 7768.8) | 5.5 (2.4, 8.8) | 1.8 (1.6,2.0) |
| Deaths | Oceania | 11.7 (4.7, 19.9) | 2.1 (0.8, 3.5) | 35.3 (14.9, 57.4) | 2.4 (1, 3.9) | 0.6 (0.5,0.7) |
| Deaths | South Asia | 398.5 (125, 679.8) | 0.3 (0.1, 0.6) | 2519.1 (972, 4082.3) | 0.8 (0.3, 1.3) | 2.9 (2.8,2.9) |
| Deaths | Southeast Asia | 509.3 (173.5, 841.6) | 1 (0.3, 1.6) | 3440.9 (1380.4, 5708.3) | 2.5 (1, 4.1) | 3.2 (3.1,3.3) |
| Deaths | Southern Latin America | 874.3 (370.9, 1443.7) | 8.9 (3.8, 14.7) | 2108 (912.6, 3483.2) | 10.8 (4.7, 17.8) | 0.9 (0.7,1.1) |
| Deaths | Southern Sub-Saharan Africa | 163.5 (66.5, 267.1) | 3.1 (1.3, 5.2) | 704.1 (295.6, 1117.9) | 6.3 (2.6, 10) | 2.4 (2.1,2.7) |

****Burden of** Colon and rectum cancer **Deaths Attributable to High BMI in Country 1990–2021****

| **Measure** | **Location** | **Number 1990** | **ASMR 1990** | **Number 2021** | **ASMR 2021** | **EAPC (95% CI)** |
| --- | --- | --- | --- | --- | --- | --- |
| Deaths | China | 2941.3 (1001.8, 5008.3) | 1.8 (0.6, 3.1) | 17515.7 (7157.9, 29363.8) | 3.8 (1.6, 6.4) | 2.5 (2.4,2.5) |
| Deaths | Democratic People's Republic of Korea | 34.4 (9.3, 68.6) | 1.1 (0.3, 2.3) | 150.6 (50.2, 305.2) | 2.2 (0.7, 4.5) | 2.3 (2.2,2.4) |
| Deaths | Taiwan (Province of China) | 106.1 (39.1, 176.2) | 3.2 (1.2, 5.4) | 739.2 (298.9, 1233) | 7.7 (3.1, 12.9) | 2.4 (2.1,2.8) |
| Deaths | Kingdom of Cambodia | 9.1 (2.3, 18.4) | 0.9 (0.2, 1.9) | 45.7 (14.4, 86.2) | 1.7 (0.5, 3.2) | 2.0 (1.8,2.1) |
| Deaths | Republic of Indonesia | 138.1 (41.4, 247.8) | 0.7 (0.2, 1.2) | 1003 (376.5, 1806.4) | 2 (0.7, 3.6) | 3.7 (3.6,3.9) |
| Deaths | Lao People's Democratic Republic | 4.9 (1.3, 10.1) | 1.1 (0.3, 2.3) | 20.5 (6.7, 39.9) | 2.1 (0.7, 4.2) | 2.2 (2.1,2.2) |
| Deaths | Malaysia | 62.5 (23.1, 107.3) | 3.2 (1.2, 5.6) | 339.3 (135.9, 578.3) | 5.8 (2.3, 9.8) | 1.7 (1.5,1.8) |
| Deaths | Republic of Maldives | 0.2 (0.1, 0.4) | 0.9 (0.3, 1.8) | 0.7 (0.3, 1.3) | 1 (0.4, 1.9) | -0.0 (-0.2,0.1) |
| Deaths | Republic of the Union of Myanmar | 54.1 (15.5, 111.6) | 1.1 (0.3, 2.2) | 185 (62.6, 347.6) | 1.7 (0.6, 3.3) | 1.4 (1.4,1.5) |
| Deaths | Republic of the Philippines | 75.4 (26.2, 126.3) | 1.3 (0.4, 2.2) | 517.5 (196.7, 857.2) | 3 (1.1, 4.9) | 3.0 (2.8,3.1) |
| Deaths | Democratic Socialist Republic of Sri Lanka | 13.7 (4.6, 24.8) | 0.6 (0.2, 1.2) | 65.5 (24.6, 126.3) | 1.1 (0.4, 2.1) | 2.1 (2.0,2.3) |
| Deaths | Kingdom of Thailand | 116.4 (38.7, 211.8) | 1.6 (0.5, 2.9) | 958 (357.7, 1732.7) | 3.9 (1.5, 7.1) | 2.8 (2.6,2.9) |
| Deaths | Democratic Republic of Timor-Leste | 0.2 (0, 0.4) | 0.3 (0.1, 0.7) | 1.3 (0.4, 2.6) | 0.7 (0.2, 1.4) | 2.7 (2.4,2.9) |
| Deaths | Socialist Republic of Viet Nam | 30.7 (6.4, 59.7) | 0.4 (0.1, 0.7) | 276.3 (89.3, 523.7) | 1.3 (0.4, 2.4) | 4.7 (4.5,4.8) |
| Deaths | Republic of Fiji | 2.6 (1.1, 4.7) | 3.9 (1.6, 7) | 9.2 (3.8, 15.9) | 6.2 (2.5, 10.7) | 1.5 (1.3,1.7) |
| Deaths | Republic of Kiribati | 0.2 (0.1, 0.4) | 3.2 (1.3, 5.7) | 0.7 (0.3, 1.2) | 4.7 (1.9, 8.2) | 1.1 (1.0,1.2) |
| Deaths | Republic of the Marshall Islands | 0.2 (0.1, 0.3) | 5.2 (2.1, 9.3) | 0.5 (0.2, 0.8) | 7 (2.9, 12.1) | 0.9 (0.9,1.0) |
| Deaths | Federated States of Micronesia | 0.6 (0.2, 1) | 5.5 (2.2, 9.9) | 1.1 (0.4, 2) | 7.3 (2.8, 13) | 0.9 (0.8,0.9) |
| Deaths | Independent State of Papua New Guinea | 3 (1, 5.9) | 0.8 (0.3, 1.5) | 10.1 (3.7, 18.3) | 0.9 (0.3, 1.7) | 0.4 (0.4,0.5) |
| Deaths | Independent State of Samoa | 0.9 (0.4, 1.5) | 4.9 (2, 8.4) | 1.9 (0.8, 3.2) | 6.2 (2.6, 10.6) | 0.7 (0.7,0.8) |
| Deaths | Solomon Islands | 0.7 (0.2, 1.4) | 2.4 (0.9, 4.7) | 2.5 (0.9, 4.5) | 3.5 (1.3, 6.5) | 1.2 (1.1,1.4) |
| Deaths | Kingdom of Tonga | 0.4 (0.2, 0.7) | 3.7 (1.5, 6.4) | 0.9 (0.4, 1.5) | 5.1 (2.2, 8.6) | 1.0 (0.9,1.2) |
| Deaths | Republic of Vanuatu | 0.3 (0.1, 0.6) | 2.6 (1, 5) | 1.3 (0.5, 2.3) | 3.7 (1.4, 6.7) | 1.1 (1.0,1.1) |
| Deaths | Republic of Armenia | 36.7 (15.3, 59.4) | 6.2 (2.6, 10.1) | 70.1 (29.8, 112.8) | 7.3 (3.1, 11.7) | 0.9 (0.7,1.0) |
| Deaths | Republic of Azerbaijan | 40.4 (16.3, 68) | 3.7 (1.5, 6.2) | 86.1 (36.8, 146.5) | 3.9 (1.6, 6.6) | 0.6 (0.4,0.8) |
| Deaths | Georgia | 58 (23.7, 94) | 4.1 (1.7, 6.7) | 86.4 (36.8, 142.6) | 6.5 (2.7, 10.7) | 2.8 (2.3,3.3) |
| Deaths | Republic of Kazakhstan | 169.5 (69.2, 281.8) | 6.3 (2.6, 10.4) | 205 (88.4, 331.5) | 5.5 (2.4, 8.9) | -0.1 (-0.4,0.1) |
| Deaths | Kyrgyz Republic | 30.1 (12.2, 49.6) | 4.6 (1.9, 7.7) | 36.9 (15.3, 60.6) | 3.7 (1.5, 6.2) | -0.5 (-0.6,-0.3) |
| Deaths | Mongolia | 6 (2.5, 10.3) | 2.7 (1.1, 4.7) | 15.4 (6.1, 26.6) | 3.4 (1.3, 5.8) | 0.5 (0.4,0.7) |
| Deaths | Republic of Tajikistan | 17 (6.8, 28.3) | 2.9 (1.2, 4.8) | 25.6 (10.3, 44.7) | 2.2 (0.9, 3.8) | -0.8 (-1.1,-0.6) |
| Deaths | Turkmenistan | 10.7 (4.3, 17.7) | 2.6 (1, 4.3) | 20.5 (8.2, 34.8) | 2.4 (1, 4.1) | -0.2 (-0.7,0.3) |
| Deaths | Republic of Uzbekistan | 63.8 (26.3, 106.7) | 2.6 (1.1, 4.4) | 139.6 (55.1, 240.2) | 2.5 (1, 4.3) | -0.0 (-0.5,0.4) |
| Deaths | Republic of Albania | 14.2 (5.7, 24.3) | 3.6 (1.4, 6.1) | 41.1 (17.2, 71.7) | 4.2 (1.8, 7.4) | 0.9 (0.7,1.1) |
| Deaths | Bosnia and Herzegovina | 47.3 (19.2, 79.5) | 5.5 (2.2, 9.2) | 141 (58.8, 238.8) | 9.9 (4.1, 16.8) | 2.2 (2.0,2.5) |
| Deaths | Republic of Bulgaria | 265 (113, 436.1) | 10 (4.2, 16.5) | 471.8 (198.5, 803.9) | 14.5 (6.1, 24.7) | 1.7 (1.5,1.9) |
| Deaths | Republic of Croatia | 139.5 (59.6, 230.1) | 11 (4.7, 18.3) | 309.6 (132.7, 505.4) | 14.7 (6.3, 24) | 1.1 (0.9,1.3) |
| Deaths | Czech Republic | 589 (247.5, 957.5) | 19.2 (8, 31.1) | 623.3 (267.4, 1028.9) | 12.5 (5.4, 20.7) | -1.7 (-1.9,-1.5) |
| Deaths | Hungary | 503.7 (203.8, 806.5) | 15.5 (6.3, 24.9) | 728.8 (322.2, 1189.1) | 16.5 (7.3, 26.9) | 0.1 (-0.1,0.4) |
| Deaths | North Macedonia | 28.8 (11.9, 47.2) | 7.4 (3, 12.1) | 75.9 (32.6, 130.1) | 11.1 (4.8, 19) | 1.4 (1.1,1.7) |
| Deaths | Montenegro | 9.9 (4.1, 16.4) | 7.4 (3, 12.2) | 25.3 (11.2, 42) | 11.9 (5.3, 19.9) | 1.7 (1.6,1.8) |
| Deaths | Republic of Poland | 955.5 (404.8, 1556.7) | 10 (4.2, 16.3) | 2287.8 (973.1, 3747.8) | 13.8 (5.9, 22.6) | 0.8 (0.6,1.0) |
| Deaths | Romania | 332.3 (134.6, 547.3) | 5.4 (2.2, 8.9) | 1011.2 (411.2, 1720.6) | 12 (4.9, 20.3) | 2.3 (2.1,2.6) |
| Deaths | Republic of Serbia | 235.2 (97.9, 409.5) | 10.5 (4.3, 18.3) | 486.3 (214, 809.2) | 12.9 (5.7, 21.4) | 0.6 (0.5,0.7) |
| Deaths | Slovak Republic | 193.2 (80.7, 326.2) | 14.6 (6.1, 24.7) | 332.2 (143.4, 556.4) | 15.5 (6.7, 26) | 0.2 (0.1,0.2) |
| Deaths | Republic of Slovenia | 59.7 (25.5, 97.2) | 10.9 (4.7, 17.8) | 107.4 (45.6, 177.9) | 10 (4.3, 16.6) | -0.4 (-0.7,-0.1) |
| Deaths | Republic of Belarus | 204 (82.1, 338) | 7 (2.8, 11.7) | 380.6 (157.8, 656.5) | 10.5 (4.4, 18.1) | 0.6 (0.3,0.9) |
| Deaths | Republic of Estonia | 40.6 (16.7, 65.9) | 8.9 (3.7, 14.4) | 67.4 (28.6, 113.3) | 10.4 (4.4, 17.3) | 0.3 (0.1,0.4) |
| Deaths | Republic of Latvia | 71.9 (30.2, 118.1) | 9 (3.8, 14.8) | 95.1 (40.7, 159.9) | 10.1 (4.3, 17) | 0.5 (0.3,0.7) |
| Deaths | Republic of Lithuania | 75.2 (31.7, 123.7) | 7.5 (3.2, 12.4) | 140.5 (60.1, 234.4) | 10.3 (4.4, 17.1) | 1.0 (0.8,1.2) |
| Deaths | Republic of Moldova | 81.1 (35.3, 132.1) | 8.5 (3.7, 13.9) | 155.3 (65.9, 251.1) | 11.6 (4.9, 18.8) | 1.5 (1.0,1.9) |
| Deaths | Russian Federation | 3118.9 (1332.2, 4972.6) | 7.8 (3.3, 12.4) | 6231 (2647.3, 9898.7) | 11.6 (4.9, 18.4) | 1.1 (1.0,1.3) |
| Deaths | Ukraine | 1470.5 (613.6, 2389.8) | 9.1 (3.8, 14.8) | 1618.7 (691.4, 2764.4) | 9.2 (3.9, 15.8) | -0.1 (-0.2,0.0) |
| Deaths | Brunei Darussalam | 0.9 (0.3, 1.6) | 4.4 (1.5, 8.1) | 4.2 (1.6, 7.2) | 5.6 (2.1, 9.6) | 1.3 (1.1,1.5) |
| Deaths | Japan | 1177.1 (434.3, 1932.7) | 3.2 (1.2, 5.3) | 3417.3 (1286.3, 5641) | 3.7 (1.4, 6.1) | 0.4 (0.3,0.4) |
| Deaths | Republic of Korea | 109.1 (34.7, 192.1) | 1.9 (0.6, 3.4) | 626.1 (222.5, 1086.4) | 3 (1.1, 5.3) | 1.3 (1.2,1.5) |
| Deaths | Republic of Singapore | 14.7 (5, 25.3) | 3.2 (1.1, 5.6) | 73.7 (28.5, 124.6) | 3.9 (1.5, 6.7) | 0.4 (0.2,0.6) |
| Deaths | Australia | 415.2 (168.4, 666.5) | 9.8 (4, 15.7) | 877 (371.1, 1412.7) | 8.3 (3.5, 13.3) | -0.8 (-0.9,-0.7) |
| Deaths | New Zealand | 113.4 (45.7, 186.7) | 13.3 (5.4, 21.9) | 199.2 (83.2, 329.3) | 10.2 (4.3, 16.8) | -0.9 (-1.0,-0.8) |
| Deaths | Principality of Andorra | 1.1 (0.4, 2.1) | 9.2 (3.4, 17.6) | 2.5 (1, 4.7) | 7.2 (2.7, 13.3) | -0.5 (-0.7,-0.2) |
| Deaths | Republic of Austria | 267.5 (106.3, 447.1) | 9.9 (4, 16.6) | 249.6 (101.2, 428.6) | 5.7 (2.3, 9.8) | -1.9 (-1.9,-1.8) |
| Deaths | Kingdom of Belgium | 297.3 (120.1, 488.9) | 8.5 (3.4, 14.1) | 378.6 (152.8, 643.1) | 6.6 (2.7, 11.2) | -0.8 (-0.9,-0.6) |
| Deaths | Republic of Cyprus | 8.7 (3.3, 15.1) | 5.8 (2.2, 10.2) | 26 (10.4, 45.9) | 6 (2.4, 10.7) | 0.4 (0.3,0.6) |
| Deaths | Kingdom of Denmark | 149.3 (60.4, 246.7) | 8.1 (3.3, 13.5) | 238.4 (96.1, 406.1) | 8.4 (3.4, 14.4) | -0.3 (-0.5,0.0) |
| Deaths | Republic of Finland | 94.8 (38.3, 158.8) | 5.9 (2.4, 10) | 181.3 (76.1, 306.7) | 5.9 (2.5, 9.9) | -0.1 (-0.2,0.0) |
| Deaths | French Republic | 1473.3 (569.6, 2442.7) | 7.8 (3, 12.9) | 2519.7 (1037.7, 4232.7) | 7 (2.9, 11.8) | -0.3 (-0.4,-0.2) |
| Deaths | Federal Republic of Germany | 3457.2 (1409, 5654.4) | 11.9 (4.8, 19.5) | 3552.8 (1434.2, 6015.5) | 7.6 (3.1, 12.8) | -1.8 (-1.9,-1.6) |
| Deaths | Hellenic Republic | 200.9 (80.5, 332.8) | 6 (2.4, 10) | 457.8 (185.8, 762.7) | 7.7 (3.1, 12.7) | 0.5 (0.2,0.7) |
| Deaths | Republic of Iceland | 4.6 (1.8, 7.6) | 7.1 (2.9, 11.8) | 8.3 (3.4, 14) | 6 (2.5, 10.2) | -0.4 (-0.6,-0.3) |
| Deaths | Ireland | 87 (35, 145) | 9.8 (3.9, 16.4) | 134.5 (55.7, 227.2) | 7.5 (3.1, 12.6) | -0.7 (-0.8,-0.6) |
| Deaths | State of Israel | 94.7 (39.1, 156.4) | 9.1 (3.8, 15.1) | 192.7 (81.7, 325.4) | 6.7 (2.8, 11.2) | -1.6 (-2.0,-1.3) |
| Deaths | Republic of Italy | 1402.8 (565.4, 2332.7) | 7 (2.8, 11.7) | 2495.1 (1034.7, 4129.7) | 6.8 (2.9, 11.3) | -0.2 (-0.3,-0.0) |
| Deaths | Grand Duchy of Luxembourg | 12.5 (5.1, 20.5) | 10.4 (4.2, 17.1) | 18.9 (8, 31.1) | 7.6 (3.2, 12.4) | -1.0 (-1.2,-0.8) |
| Deaths | Republic of Malta | 5.6 (2.2, 9.3) | 6.1 (2.4, 10.2) | 15.2 (6.4, 25.6) | 6.6 (2.8, 11) | 0.2 (0.0,0.3) |
| Deaths | Kingdom of the Netherlands | 389.3 (156.2, 648.6) | 8.7 (3.5, 14.5) | 765.9 (313.5, 1285.9) | 9.3 (3.8, 15.6) | 0.3 (0.1,0.5) |
| Deaths | Kingdom of Norway | 133.2 (53.9, 220.6) | 8.5 (3.5, 14.1) | 182.9 (76.8, 299.3) | 7.6 (3.2, 12.4) | -0.5 (-0.6,-0.4) |
| Deaths | Portuguese Republic | 238.4 (94, 402) | 8 (3.1, 13.5) | 507.4 (210.9, 847.6) | 8.5 (3.6, 14.1) | 0.2 (0.0,0.5) |
| Deaths | Kingdom of Spain | 1033.8 (426.1, 1730.6) | 8.5 (3.5, 14.2) | 2321.3 (971.7, 3880.7) | 9.7 (4.1, 16.1) | 0.5 (0.3,0.6) |
| Deaths | Kingdom of Sweden | 246.5 (98.4, 415.3) | 7.1 (2.8, 12) | 351.2 (144.9, 588.2) | 6.6 (2.7, 11) | -0.2 (-0.3,-0.0) |
| Deaths | Swiss Confederation | 129.7 (52.8, 213.3) | 5.4 (2.2, 8.9) | 199 (79, 334.8) | 4.5 (1.8, 7.5) | -0.7 (-0.9,-0.6) |
| Deaths | United Kingdom of Great Britain and Northern Ireland | 2304.7 (981, 3754.8) | 11.2 (4.8, 18.3) | 2794.8 (1205.1, 4546.3) | 9 (3.9, 14.5) | -0.8 (-0.9,-0.7) |
| Deaths | Argentine Republic | 657.2 (276.8, 1098.5) | 9.5 (4, 15.9) | 1441.6 (623.3, 2407.9) | 11.6 (5, 19.3) | 0.9 (0.7,1.2) |
| Deaths | Republic of Chile | 120.6 (49.5, 201.3) | 5.9 (2.4, 9.9) | 472.5 (203.6, 777.6) | 8.2 (3.5, 13.5) | 1.4 (1.2,1.5) |
| Deaths | Eastern Republic of Uruguay | 96.4 (39.1, 161.2) | 11.1 (4.5, 18.6) | 193.9 (82.7, 325.2) | 14.9 (6.4, 24.8) | 0.8 (0.7,0.9) |
| Deaths | Canada | 669.7 (283.8, 1088.8) | 9.4 (4, 15.3) | 1310.8 (571.2, 2149.2) | 7.7 (3.4, 12.6) | -0.5 (-0.6,-0.4) |
| Deaths | United States of America | 6985 (2967.6, 11267.6) | 9.8 (4.2, 15.8) | 10734.1 (4768.7, 16815.2) | 8.1 (3.6, 12.6) | -0.8 (-1.0,-0.7) |
| Deaths | Antigua and Barbuda | 0.5 (0.2, 0.8) | 4 (1.6, 6.7) | 1.7 (0.7, 2.8) | 7.7 (3.2, 12.5) | 2.1 (1.9,2.3) |
| Deaths | Commonwealth of the Bahamas | 1.9 (0.8, 3.2) | 6 (2.5, 10) | 8.6 (3.5, 14.3) | 10.1 (4.1, 16.9) | 2.0 (1.8,2.1) |
| Deaths | Barbados | 4.7 (1.8, 7.8) | 7.2 (2.9, 12) | 15.3 (6.5, 25.8) | 13.1 (5.5, 22.2) | 2.3 (2.0,2.6) |
| Deaths | Belize | 0.5 (0.2, 0.9) | 2.8 (1.1, 4.6) | 3.3 (1.5, 5.2) | 5.4 (2.4, 8.6) | 2.2 (1.7,2.7) |
| Deaths | Republic of Cuba | 94.4 (36.8, 154.6) | 4.3 (1.7, 7.1) | 357.4 (143.6, 617.9) | 8 (3.2, 13.8) | 2.2 (2.1,2.3) |
| Deaths | Commonwealth of Dominica | 0.8 (0.3, 1.4) | 6.3 (2.5, 10.8) | 1.8 (0.8, 3) | 10 (4.2, 16.7) | 1.6 (1.5,1.7) |
| Deaths | Dominican Republic | 12.8 (4.7, 22.8) | 1.8 (0.6, 3.2) | 76.6 (29.4, 139) | 3.6 (1.4, 6.5) | 2.8 (2.6,3.0) |
| Deaths | Grenada | 0.6 (0.2, 1) | 3.9 (1.5, 6.6) | 1.8 (0.7, 3.1) | 7.6 (3, 12.9) | 2.5 (2.4,2.7) |
| Deaths | Republic of Guyana | 2.6 (1, 4.5) | 3.4 (1.3, 5.8) | 7.6 (3.1, 13.2) | 5.6 (2.3, 9.8) | 2.0 (1.7,2.2) |
| Deaths | Republic of Haiti | 9.9 (2.9, 19.1) | 1.5 (0.4, 2.9) | 37.7 (12.8, 71.9) | 2.6 (0.9, 5) | 2.1 (2.0,2.2) |
| Deaths | Jamaica | 14.2 (5.7, 23.9) | 3.6 (1.4, 6) | 53 (22.5, 89.5) | 7.7 (3.3, 13.1) | 2.6 (2.3,2.9) |
| Deaths | Saint Lucia | 0.6 (0.2, 1) | 3.4 (1.3, 5.7) | 2.4 (1, 4.1) | 4.6 (1.8, 7.8) | 0.6 (0.4,0.8) |
| Deaths | Saint Vincent and the Grenadines | 0.4 (0.1, 0.7) | 2.7 (1, 4.7) | 1.5 (0.6, 2.5) | 4.8 (2, 8.2) | 1.9 (1.8,2.1) |
| Deaths | Republic of Suriname | 1.6 (0.6, 2.8) | 3 (1.1, 5.2) | 7 (2.7, 12.5) | 5.1 (1.9, 9.1) | 2.1 (1.9,2.3) |
| Deaths | Republic of Trinidad and Tobago | 9.4 (3.8, 15.5) | 5.6 (2.3, 9.2) | 32 (13.2, 53.9) | 7.5 (3.1, 12.6) | 0.9 (0.8,1.0) |
| Deaths | Plurinational State of Bolivia | 24.5 (8.1, 47.4) | 3.8 (1.3, 7.3) | 116.3 (43.8, 214) | 6.3 (2.4, 11.6) | 1.7 (1.6,1.7) |
| Deaths | Republic of Ecuador | 25.7 (10.1, 43.1) | 2.4 (1, 4.1) | 180.4 (73.7, 306.5) | 5.2 (2.1, 8.9) | 2.9 (2.6,3.2) |
| Deaths | Republic of Peru | 65.4 (25.9, 111.8) | 2.7 (1.1, 4.6) | 306.3 (127.1, 545.2) | 4.3 (1.8, 7.6) | 1.4 (1.2,1.6) |
| Deaths | Republic of Colombia | 107.8 (42.5, 180.6) | 3.1 (1.2, 5.2) | 637.5 (267.8, 1076.3) | 5.3 (2.2, 8.9) | 1.5 (1.4,1.7) |
| Deaths | Republic of Costa Rica | 13.1 (5.3, 22) | 3.6 (1.5, 6.1) | 95.6 (39.5, 158.5) | 7.9 (3.3, 13.1) | 2.8 (2.6,3.0) |
| Deaths | Republic of El Salvador | 12.8 (5.2, 21.3) | 2.1 (0.8, 3.4) | 62.9 (25.8, 108.1) | 4.6 (1.9, 7.9) | 2.6 (2.4,2.8) |
| Deaths | Republic of Guatemala | 10.7 (4.3, 17.7) | 1.8 (0.7, 3) | 78 (32.9, 130.7) | 3.5 (1.5, 5.8) | 2.1 (1.8,2.4) |
| Deaths | Republic of Honduras | 5.8 (2.2, 10.1) | 1.4 (0.5, 2.4) | 40.1 (15.6, 71.5) | 3.1 (1.2, 5.6) | 2.8 (2.6,3.0) |
| Deaths | United Mexican States | 208.2 (87.1, 341.4) | 2.5 (1.1, 4.2) | 1356.3 (596.5, 2164.4) | 5 (2.2, 8) | 2.3 (2.2,2.4) |
| Deaths | Republic of Nicaragua | 5.7 (2.3, 9.6) | 1.9 (0.7, 3.2) | 33.9 (13.8, 58.2) | 3.4 (1.4, 5.8) | 2.4 (2.1,2.7) |
| Deaths | Republic of Panama | 12.3 (5, 20.2) | 4 (1.7, 6.6) | 69 (30.2, 114.8) | 7.1 (3.1, 11.8) | 2.1 (2.0,2.2) |
| Deaths | Bolivarian Republic of Venezuela | 73.1 (29.9, 121.4) | 3.8 (1.5, 6.3) | 384.3 (159.5, 658.3) | 6 (2.5, 10.3) | 1.5 (1.4,1.6) |
| Deaths | Federative Republic of Brazil | 591.8 (238.8, 963) | 3.3 (1.3, 5.4) | 3174.5 (1341.4, 5091.3) | 5.8 (2.5, 9.4) | 1.8 (1.7,1.9) |
| Deaths | Republic of Paraguay | 10.3 (4.1, 17.9) | 2.3 (0.9, 3.9) | 67.4 (27.4, 120.7) | 5.6 (2.3, 10) | 3.3 (3.1,3.4) |
| Deaths | People's Democratic Republic of Algeria | 32.3 (12.3, 57.8) | 1.5 (0.6, 2.7) | 162 (66.3, 283.6) | 2.5 (1, 4.4) | 2.0 (1.8,2.2) |
| Deaths | Kingdom of Bahrain | 1.6 (0.6, 2.7) | 5.3 (2.1, 9.1) | 10.2 (4.2, 17.7) | 7.3 (3, 12.6) | 0.6 (0.3,0.9) |
| Deaths | Arab Republic of Egypt | 133.4 (54.8, 221.4) | 2.6 (1, 4.3) | 783.9 (340.3, 1303.7) | 6.6 (2.8, 10.9) | 3.9 (3.6,4.2) |
| Deaths | Islamic Republic of Iran | 106 (43.5, 175.7) | 2.1 (0.8, 3.4) | 644.5 (275.7, 1028.8) | 4.1 (1.8, 6.6) | 2.7 (2.5,2.9) |
| Deaths | Republic of Iraq | 40.7 (16.2, 69.6) | 2.5 (1, 4.3) | 178.3 (74.1, 311.5) | 3.9 (1.6, 6.8) | 1.5 (1.2,1.7) |
| Deaths | Hashemite Kingdom of Jordan | 15 (6.1, 26) | 5.7 (2.3, 9.8) | 92.8 (39.1, 159.4) | 6.6 (2.8, 11.4) | 0.6 (0.3,0.8) |
| Deaths | State of Kuwait | 3.3 (1.4, 5.4) | 3.2 (1.3, 5.2) | 36.1 (16.5, 59.9) | 7.1 (3.2, 11.7) | 3.2 (2.7,3.7) |
| Deaths | Lebanese Republic | 26.4 (9.8, 47.7) | 6.1 (2.3, 11) | 100.2 (40.7, 168.6) | 7.3 (3, 12.3) | 1.1 (0.8,1.3) |
| Deaths | State of Libya | 20.6 (7.9, 37) | 5.4 (2.1, 9.7) | 99.5 (40.4, 176) | 9.9 (4, 17.4) | 2.3 (2.2,2.5) |
| Deaths | Kingdom of Morocco | 54.3 (19.5, 98.2) | 1.8 (0.7, 3.3) | 287.4 (105.2, 522.3) | 4 (1.5, 7.2) | 2.8 (2.7,2.9) |
| Deaths | Palestine | 15.1 (6.1, 26.9) | 8.8 (3.5, 15.6) | 49.7 (20.4, 82) | 10.6 (4.3, 17.6) | 0.7 (0.5,0.9) |
| Deaths | Sultanate of Oman | 1.7 (0.7, 3.1) | 1.3 (0.5, 2.3) | 8.4 (3.4, 14.5) | 2.4 (1, 4.2) | 2.6 (2.3,2.8) |
| Deaths | State of Qatar | 1.2 (0.5, 2) | 7.5 (3, 13.2) | 10.7 (4.6, 18.5) | 9.1 (3.9, 15.6) | 0.8 (0.2,1.5) |
| Deaths | Kingdom of Saudi Arabia | 26.9 (10.5, 48.4) | 2.5 (1, 4.4) | 189.4 (80.3, 309.9) | 5.6 (2.3, 9.2) | 2.9 (2.4,3.3) |
| Deaths | Syrian Arab Republic | 28.3 (11.3, 48.5) | 2.7 (1.1, 4.6) | 121.6 (48.4, 210.7) | 4.7 (1.9, 8.1) | 1.7 (1.5,1.8) |
| Deaths | Republic of Tunisia | 23.9 (9.1, 41.9) | 2.4 (0.9, 4.2) | 113.3 (45.1, 205.4) | 4.1 (1.6, 7.3) | 1.6 (1.6,1.7) |
| Deaths | Republic of Turkey | 481.6 (194.2, 815.7) | 6.9 (2.8, 11.7) | 1641.8 (697.9, 2809.2) | 8.3 (3.5, 14.2) | 0.5 (0.2,0.9) |
| Deaths | United Arab Emirates | 5.2 (1.8, 9.8) | 7.2 (2.5, 13.4) | 43.4 (17.4, 76.2) | 9.9 (4, 17.2) | 2.9 (2.3,3.4) |
| Deaths | Republic of Yemen | 11.9 (3.9, 23.4) | 1.2 (0.4, 2.4) | 67.2 (23.8, 122.8) | 2.5 (0.9, 4.5) | 2.5 (2.3,2.6) |
| Deaths | Islamic Republic of Afghanistan | 49.5 (11.4, 102) | 3.1 (0.7, 6.4) | 81.4 (25.1, 161.3) | 4.4 (1.4, 8.6) | 1.2 (1.1,1.3) |
| Deaths | People's Republic of Bangladesh | 22.4 (5.7, 45.2) | 0.2 (0.1, 0.5) | 133.4 (42.3, 254.1) | 0.5 (0.1, 0.9) | 2.4 (2.3,2.5) |
| Deaths | Kingdom of Bhutan | 0.5 (0.2, 1) | 0.9 (0.3, 1.9) | 1.8 (0.7, 3.3) | 1.4 (0.5, 2.6) | 1.2 (1.1,1.3) |
| Deaths | Republic of India | 293.4 (88.6, 518.6) | 0.3 (0.1, 0.5) | 1936.5 (737.4, 3215.2) | 0.8 (0.3, 1.3) | 3.1 (3.0,3.2) |
| Deaths | Federal Democratic Republic of Nepal | 5.6 (1.5, 11.6) | 0.3 (0.1, 0.6) | 29.6 (10.1, 56.2) | 0.6 (0.2, 1.1) | 2.8 (2.4,3.2) |
| Deaths | Islamic Republic of Pakistan | 76.6 (24.2, 139.3) | 0.7 (0.2, 1.2) | 417.8 (163.9, 714.4) | 1.7 (0.7, 2.9) | 3.2 (2.9,3.4) |
| Deaths | Republic of Angola | 6.9 (2.2, 13.3) | 0.9 (0.3, 1.7) | 44.7 (15.8, 85.4) | 1.9 (0.7, 3.7) | 2.5 (2.4,2.7) |
| Deaths | Central African Republic | 2.2 (0.7, 4.6) | 0.9 (0.3, 1.9) | 8.1 (2.7, 16.1) | 1.8 (0.6, 3.5) | 2.2 (2.1,2.2) |
| Deaths | Republic of the Congo | 3.9 (1.3, 7.4) | 1.7 (0.5, 3.2) | 16.4 (6, 29.6) | 3 (1.1, 5.5) | 1.7 (1.6,1.8) |
| Deaths | Democratic Republic of the Congo | 26.4 (8.4, 49.6) | 0.9 (0.3, 1.6) | 129.6 (45, 243.9) | 1.9 (0.6, 3.6) | 2.6 (2.4,2.9) |
| Deaths | Republic of Equatorial Guinea | 0.6 (0.2, 1.2) | 1.6 (0.5, 3) | 4 (1.5, 7.7) | 4.3 (1.6, 8.3) | 3.5 (3.4,3.7) |
| Deaths | Gabonese Republic | 4.4 (1.4, 9) | 3.6 (1.2, 7.3) | 13.1 (5.1, 23.7) | 6.3 (2.4, 11.4) | 1.8 (1.7,1.8) |
| Deaths | Republic of Burundi | 3.6 (0.9, 7.2) | 0.8 (0.2, 1.5) | 9 (2.8, 17.7) | 1 (0.3, 1.9) | 0.4 (0.2,0.6) |
| Deaths | Union of the Comoros | 0.5 (0.2, 0.9) | 1.3 (0.4, 2.4) | 2.8 (1, 5.2) | 2.9 (1, 5.3) | 2.7 (2.6,2.8) |
| Deaths | Republic of Djibouti | 0.2 (0.1, 0.4) | 0.7 (0.2, 1.5) | 2 (0.6, 3.9) | 1.6 (0.5, 3.2) | 2.6 (2.6,2.6) |
| Deaths | State of Eritrea | 1.5 (0.4, 3) | 0.6 (0.2, 1.3) | 7.5 (2.4, 14.6) | 1.4 (0.5, 2.8) | 2.7 (2.6,2.7) |
| Deaths | Federal Democratic Republic of Ethiopia | 74.9 (21.6, 139.4) | 1.9 (0.5, 3.5) | 170.7 (55.7, 300.3) | 2 (0.7, 3.6) | -0.1 (-0.3,0.2) |
| Deaths | Republic of Kenya | 11.7 (3.9, 20.7) | 0.7 (0.2, 1.2) | 89.8 (34.7, 153.1) | 2 (0.7, 3.4) | 4.0 (3.7,4.2) |
| Deaths | Republic of Madagascar | 8.3 (2.5, 16.2) | 0.8 (0.2, 1.6) | 31.1 (10.7, 58.5) | 1.5 (0.5, 2.9) | 2.0 (1.9,2.2) |
| Deaths | Republic of Malawi | 3.3 (0.9, 6.2) | 0.5 (0.1, 0.8) | 13.7 (4.6, 26.1) | 1 (0.3, 1.8) | 2.3 (2.1,2.5) |
| Deaths | Republic of Mauritius | 2.8 (1.1, 4.7) | 1.9 (0.7, 3.1) | 21.2 (8.7, 34.7) | 5.2 (2.1, 8.5) | 2.8 (2.6,3.0) |
| Deaths | Republic of Mozambique | 3.8 (1.1, 7.3) | 0.3 (0.1, 0.7) | 16 (5.2, 29.5) | 0.8 (0.2, 1.5) | 3.2 (3.0,3.4) |
| Deaths | Republic of Rwanda | 6.4 (1.9, 12.7) | 1.1 (0.3, 2.2) | 19.3 (5.9, 38.1) | 1.6 (0.5, 3.1) | 0.5 (0.1,0.8) |
| Deaths | Republic of Seychelles | 0.5 (0.2, 0.9) | 4.4 (1.6, 7.5) | 2.2 (0.9, 3.7) | 9 (3.7, 15.1) | 2.5 (2.2,2.8) |
| Deaths | Federal Republic of Somalia | 5.1 (1.5, 10.5) | 1 (0.3, 2.1) | 19.5 (5.9, 39) | 1.6 (0.5, 3.3) | 1.6 (1.6,1.7) |
| Deaths | United Republic of Tanzania | 32.5 (11, 59.9) | 1.5 (0.5, 2.7) | 144.8 (54.8, 262.3) | 2.9 (1.1, 5.4) | 2.3 (2.2,2.3) |
| Deaths | Republic of Uganda | 14.1 (4.3, 26.4) | 1.1 (0.3, 2) | 59.1 (20.5, 108.3) | 2 (0.7, 3.7) | 1.5 (1.3,1.8) |
| Deaths | Republic of Zambia | 7.4 (2.4, 13.8) | 1.3 (0.4, 2.4) | 49.1 (16.4, 105.4) | 3.6 (1.2, 7.6) | 3.3 (3.2,3.4) |
| Deaths | Republic of Botswana | 2.3 (0.8, 4.5) | 2.2 (0.7, 4.4) | 11.5 (4.3, 21.2) | 4.4 (1.6, 8.1) | 2.7 (2.4,2.9) |
| Deaths | Kingdom of Lesotho | 3.7 (1.4, 6.9) | 2.2 (0.8, 4.1) | 13.2 (4.8, 25.5) | 6.3 (2.3, 12.2) | 4.4 (3.9,4.9) |
| Deaths | Republic of Namibia | 1.6 (0.6, 2.9) | 1.3 (0.5, 2.3) | 7.4 (2.8, 12.9) | 2.8 (1.1, 4.9) | 2.5 (2.3,2.8) |
| Deaths | Republic of South Africa | 138.5 (56.2, 231) | 3.4 (1.4, 5.7) | 594.4 (250.5, 942.1) | 6.5 (2.7, 10.3) | 2.1 (1.8,2.4) |
| Deaths | Kingdom of Eswatini | 2.4 (0.9, 4.6) | 4.8 (1.8, 9.1) | 9.7 (3.7, 17.9) | 9.7 (3.7, 17.7) | 2.7 (2.2,3.2) |
| Deaths | Republic of Zimbabwe | 15 (5.3, 27.3) | 1.9 (0.6, 3.4) | 67.9 (27, 119.8) | 5.1 (2, 9) | 3.9 (3.3,4.4) |
| Deaths | Republic of Benin | 3.9 (1.4, 7.2) | 1 (0.3, 1.8) | 16.3 (5.9, 30.1) | 1.6 (0.6, 3) | 1.9 (1.8,2.0) |
| Deaths | Burkina Faso | 3.8 (1, 7.2) | 0.4 (0.1, 0.8) | 14 (4.2, 27.1) | 0.8 (0.2, 1.5) | 2.1 (2.0,2.3) |
| Deaths | Republic of Cameroon | 17.3 (6.5, 30.4) | 2 (0.8, 3.5) | 84.1 (30.7, 155.5) | 3.7 (1.4, 6.8) | 2.2 (2.1,2.2) |
| Deaths | Republic of Cabo Verde | 0.3 (0.1, 0.6) | 0.7 (0.2, 1.2) | 2.9 (1.1, 5.1) | 3.2 (1.2, 5.6) | 4.6 (4.2,5.0) |
| Deaths | Republic of Chad | 3.6 (1.2, 6.9) | 0.6 (0.2, 1.2) | 13.9 (4.9, 25.7) | 1.3 (0.4, 2.4) | 2.5 (2.4,2.6) |
| Deaths | Republic of Côte d'Ivoire | 6.3 (2.4, 11.5) | 0.9 (0.3, 1.6) | 27.5 (10.2, 51.3) | 1.3 (0.5, 2.5) | 1.5 (1.4,1.5) |
| Deaths | Republic of the Gambia | 0.4 (0.1, 0.8) | 0.6 (0.2, 1.1) | 1.9 (0.7, 3.5) | 1 (0.4, 1.8) | 1.6 (1.4,1.7) |
| Deaths | Republic of Ghana | 9.6 (3.3, 18.1) | 0.8 (0.3, 1.5) | 69.1 (24.8, 126.5) | 2.3 (0.8, 4.2) | 3.6 (3.5,3.7) |
| Deaths | Republic of Guinea | 4.4 (1.5, 8.3) | 0.6 (0.2, 1.2) | 12.1 (4.1, 22.6) | 1.1 (0.4, 2) | 1.8 (1.8,1.8) |
| Deaths | Republic of Guinea-Bissau | 0.9 (0.3, 1.7) | 1.1 (0.4, 2.2) | 2.8 (1, 5.3) | 2.1 (0.8, 4) | 2.3 (2.2,2.3) |
| Deaths | Republic of Liberia | 3.2 (1.1, 5.7) | 1.3 (0.5, 2.4) | 8.9 (3.1, 17.2) | 2.3 (0.8, 4.4) | 2.0 (1.8,2.3) |
| Deaths | Republic of Mali | 7.7 (2.5, 14.1) | 0.9 (0.3, 1.7) | 22.1 (7.4, 41.6) | 1.3 (0.4, 2.4) | 1.1 (1.0,1.2) |
| Deaths | Islamic Republic of Mauritania | 4.2 (1.6, 7.7) | 2.1 (0.8, 3.9) | 14.1 (5.3, 25) | 3.5 (1.3, 6.1) | 1.6 (1.4,1.8) |
| Deaths | Republic of the Niger | 3.3 (1.1, 6.3) | 0.6 (0.2, 1.2) | 14.3 (4.7, 28.3) | 0.9 (0.3, 1.8) | 1.4 (1.3,1.5) |
| Deaths | Federal Republic of Nigeria | 89.5 (33.4, 152.9) | 1 (0.4, 1.8) | 366.6 (143.9, 617) | 2.2 (0.9, 3.7) | 2.6 (2.5,2.7) |
| Deaths | Democratic Republic of Sao Tome and Principe | 0.2 (0.1, 0.4) | 1.6 (0.5, 2.8) | 0.7 (0.3, 1.3) | 3.7 (1.4, 6.4) | 2.9 (2.8,2.9) |
| Deaths | Republic of Senegal | 6.8 (2.3, 12.5) | 1.1 (0.4, 1.9) | 29.6 (10.6, 53.8) | 2 (0.7, 3.6) | 2.2 (2.0,2.3) |
| Deaths | Republic of Sierra Leone | 2.9 (0.9, 5.5) | 0.7 (0.2, 1.3) | 9.7 (3.3, 18.2) | 1.3 (0.4, 2.5) | 2.4 (2.3,2.6) |
| Deaths | Togolese Republic | 1.9 (0.7, 3.6) | 0.8 (0.3, 1.5) | 12.8 (4.5, 24) | 1.9 (0.7, 3.6) | 2.8 (2.8,2.9) |
| Deaths | American Samoa | 0.4 (0.1, 0.6) | 8.2 (3.3, 13.9) | 1 (0.5, 1.7) | 10.4 (4.6, 17.2) | 0.9 (0.8,1.0) |
| Deaths | Bermuda | 1.7 (0.7, 2.8) | 12.7 (5.2, 21.4) | 3.8 (1.6, 6.4) | 11.8 (5.1, 19.6) | -0.1 (-0.3,0.0) |
| Deaths | Cook Islands | 0.1 (0, 0.2) | 4.1 (1.7, 7.2) | 0.2 (0.1, 0.4) | 3.8 (1.6, 6.4) | -0.5 (-0.6,-0.3) |
| Deaths | Greenland | 1.2 (0.5, 2.1) | 20 (8.2, 34.1) | 1.9 (0.8, 3.3) | 13.2 (5.4, 22.9) | -1.4 (-1.5,-1.3) |
| Deaths | Guam | 1 (0.4, 1.6) | 6.9 (2.8, 11.9) | 2.6 (1.1, 4.3) | 5.3 (2.2, 8.9) | -0.3 (-0.6,0.1) |
| Deaths | Principality of Monaco | 1.8 (0.7, 3.2) | 11 (4.2, 19.3) | 3.3 (1.4, 5.9) | 13.7 (5.7, 24.4) | 0.8 (0.7,0.9) |
| Deaths | Republic of Nauru | 0.1 (0, 0.2) | 8.9 (3.2, 17) | 0.1 (0, 0.2) | 11 (4.2, 19.6) | 0.6 (0.6,0.7) |
| Deaths | Republic of Niue | 0 (0, 0) | 4.9 (2, 8.7) | 0 (0, 0.1) | 7.2 (3.1, 12.3) | 1.2 (1.1,1.2) |
| Deaths | Northern Mariana Islands | 0.2 (0.1, 0.4) | 7.6 (3.1, 13.5) | 1.1 (0.5, 1.8) | 10.3 (4.4, 16.9) | 1.0 (0.8,1.2) |
| Deaths | Republic of Palau | 0.1 (0.1, 0.3) | 7.5 (2.9, 13.5) | 0.4 (0.1, 0.7) | 8.7 (3.5, 15.6) | 0.6 (0.6,0.7) |
| Deaths | Puerto Rico | 50.9 (21.5, 83.9) | 6.6 (2.8, 10.9) | 136.1 (59.7, 224.4) | 8.2 (3.6, 13.5) | 0.7 (0.4,0.9) |
| Deaths | Saint Kitts and Nevis | 0.5 (0.2, 0.8) | 5.7 (2.2, 9.5) | 1.3 (0.5, 2.1) | 9 (3.7, 14.9) | 2.1 (1.9,2.3) |
| Deaths | Republic of San Marino | 0.8 (0.3, 1.4) | 10.2 (4, 17.6) | 1.1 (0.4, 2.1) | 5.9 (2.2, 11.1) | -0.8 (-1.2,-0.5) |
| Deaths | Tokelau | 0 (0, 0) | 4.6 (1.8, 8.2) | 0 (0, 0) | 5.9 (2.5, 10.2) | 0.8 (0.8,0.9) |
| Deaths | Tuvalu | 0.1 (0, 0.1) | 4.4 (1.7, 7.7) | 0.1 (0.1, 0.2) | 6.1 (2.5, 10.5) | 1.1 (1.1,1.1) |
| Deaths | United States Virgin Islands | 1.7 (0.7, 2.9) | 10.6 (4.3, 18.2) | 3.2 (1.3, 5.5) | 7.9 (3.3, 13.5) | -0.9 (-1.1,-0.7) |
| Deaths | Republic of South Sudan | 3.7 (0.9, 7.6) | 0.7 (0.2, 1.5) | 9.5 (2.8, 18.4) | 1.2 (0.4, 2.4) | 1.9 (1.7,2.1) |
| Deaths | Republic of Sudan | 36.7 (13.3, 70.2) | 1.9 (0.7, 3.7) | 136.7 (52.5, 251.8) | 3.6 (1.4, 6.5) | 2.1 (2.0,2.1) |

****Burden of** Colon and rectum cancer **Deaths Attributable to High BMI in Country 1990–2021****

| **Measure** | **Location** | **Number 1990** | **ASR 1990** | **Number 2021** | **ASR 2021** | **EAPC (95% CI)** |
| --- | --- | --- | --- | --- | --- | --- |
| DALYs (Disability-Adjusted Life Years) | China | 75537 (25927.9, 128893.1) | 40.8 (13.9, 69.6) | 411982.4 (169014.4, 692218.2) | 84.6 (34.6, 142.1) | 2.4 (2.3,2.5) |
| DALYs (Disability-Adjusted Life Years) | Democratic People's Republic of Korea | 836.1 (218, 1670.9) | 24.2 (6.5, 48.1) | 3208.9 (1039.7, 6473.4) | 44.5 (14.5, 89.8) | 2.1 (2.0,2.2) |
| DALYs (Disability-Adjusted Life Years) | Taiwan (Province of China) | 2648.3 (979.3, 4400.8) | 74.1 (27.3, 123.5) | 15686 (6350.4, 26346.3) | 166 (67.2, 279) | 2.2 (1.9,2.6) |
| DALYs (Disability-Adjusted Life Years) | Kingdom of Cambodia | 253.5 (67.4, 512.2) | 24.3 (6.3, 49.1) | 1233 (399.3, 2315.9) | 42.1 (13.4, 79.5) | 1.8 (1.7,1.9) |
| DALYs (Disability-Adjusted Life Years) | Republic of Indonesia | 3867.7 (1201.4, 6892.8) | 16.8 (5.1, 30.2) | 27372 (10495.2, 48861.2) | 47.6 (18, 85.7) | 3.6 (3.4,3.8) |
| DALYs (Disability-Adjusted Life Years) | Lao People's Democratic Republic | 133.3 (36.1, 274.2) | 28.1 (7.5, 57.9) | 550.9 (182.4, 1073.7) | 51.8 (17, 100.9) | 2.1 (2.0,2.2) |
| DALYs (Disability-Adjusted Life Years) | Malaysia | 1640.4 (622.1, 2800.8) | 80.7 (30.3, 138) | 8315.8 (3362.1, 14245.1) | 131.4 (52.9, 225) | 1.4 (1.3,1.5) |
| DALYs (Disability-Adjusted Life Years) | Republic of Maldives | 6.1 (1.8, 11.6) | 25.8 (7.4, 48.9) | 19 (7.3, 34.2) | 25.1 (9.6, 45.5) | -0.4 (-0.6,-0.3) |
| DALYs (Disability-Adjusted Life Years) | Republic of the Union of Myanmar | 1524 (448, 3138.9) | 27.9 (8.1, 57.6) | 4971.8 (1709.5, 9277.1) | 43.3 (14.8, 81.1) | 1.3 (1.2,1.4) |
| DALYs (Disability-Adjusted Life Years) | Republic of the Philippines | 2012.6 (712.1, 3348.9) | 30.6 (10.7, 51.2) | 13689.2 (5250.2, 22626.3) | 72.2 (27.6, 119.5) | 3.0 (2.8,3.1) |
| DALYs (Disability-Adjusted Life Years) | Democratic Socialist Republic of Sri Lanka | 346.4 (116.4, 620.9) | 14.8 (4.9, 26.6) | 1568.5 (587.5, 3033.5) | 25.4 (9.5, 49.1) | 2.1 (1.9,2.3) |
| DALYs (Disability-Adjusted Life Years) | Kingdom of Thailand | 3104.4 (1053.7, 5554.3) | 38.3 (12.8, 69.2) | 23167.7 (8752.8, 41634.2) | 93 (35.1, 167.1) | 2.7 (2.6,2.9) |
| DALYs (Disability-Adjusted Life Years) | Democratic Republic of Timor-Leste | 5.2 (1, 10.6) | 8.3 (1.5, 17) | 34.1 (9.6, 67.1) | 18 (5, 35.4) | 2.8 (2.5,3.1) |
| DALYs (Disability-Adjusted Life Years) | Socialist Republic of Viet Nam | 790.9 (172.7, 1532.1) | 8.8 (1.9, 17) | 7430.7 (2457.1, 13967.3) | 31.6 (10.3, 59.6) | 4.8 (4.6,5.0) |
| DALYs (Disability-Adjusted Life Years) | Republic of Fiji | 70.5 (28.6, 124.1) | 90.8 (36.6, 161.1) | 233.7 (96.2, 403.6) | 135.6 (55.7, 234.1) | 1.3 (1.2,1.5) |
| DALYs (Disability-Adjusted Life Years) | Republic of Kiribati | 7 (2.8, 12.1) | 83 (32.9, 145.3) | 18.9 (7.6, 32.6) | 112.5 (44.8, 194.4) | 0.9 (0.8,0.9) |
| DALYs (Disability-Adjusted Life Years) | Republic of the Marshall Islands | 4.3 (1.8, 7.5) | 126 (52.4, 220.2) | 13.1 (5.5, 22.9) | 163.8 (67.9, 284.7) | 0.8 (0.8,0.9) |
| DALYs (Disability-Adjusted Life Years) | Federated States of Micronesia | 14.4 (5.7, 25.4) | 137.7 (53.8, 244.1) | 30.9 (12.1, 54.7) | 174.4 (68.2, 309.4) | 0.7 (0.7,0.8) |
| DALYs (Disability-Adjusted Life Years) | Independent State of Papua New Guinea | 89.9 (31.2, 175.9) | 20.4 (7, 40) | 300.5 (112.3, 540.5) | 24 (8.8, 43.5) | 0.4 (0.4,0.5) |
| DALYs (Disability-Adjusted Life Years) | Independent State of Samoa | 22.9 (9.6, 38.1) | 118.6 (49.2, 198.7) | 47.9 (20.8, 81.8) | 146.7 (63.3, 250.8) | 0.7 (0.6,0.7) |
| DALYs (Disability-Adjusted Life Years) | Solomon Islands | 20 (7.1, 39) | 61.5 (21.7, 119.7) | 69.4 (25.8, 125.9) | 87.9 (32.4, 160.2) | 1.2 (1.0,1.4) |
| DALYs (Disability-Adjusted Life Years) | Kingdom of Tonga | 11.5 (4.8, 19.4) | 90.5 (37.1, 153.3) | 20.4 (9.1, 34.2) | 115.8 (51.6, 193.9) | 0.8 (0.7,0.9) |
| DALYs (Disability-Adjusted Life Years) | Republic of Vanuatu | 8.7 (3.2, 16.6) | 63.5 (23.3, 120.4) | 35.6 (13.6, 62.9) | 89.9 (34.4, 159.2) | 1.0 (0.9,1.2) |
| DALYs (Disability-Adjusted Life Years) | Republic of Armenia | 959.2 (399.2, 1544.6) | 150.4 (62.7, 242.5) | 1556.1 (666.4, 2498.3) | 159.5 (68.2, 256.5) | 0.5 (0.3,0.6) |
| DALYs (Disability-Adjusted Life Years) | Republic of Azerbaijan | 1103.3 (447.2, 1854.1) | 92.5 (37.6, 155.8) | 2294.5 (985.7, 3901.1) | 93 (39.7, 158.6) | 0.3 (0.1,0.5) |
| DALYs (Disability-Adjusted Life Years) | Georgia | 1493.4 (613, 2415.8) | 101.7 (41.7, 164.6) | 1988.1 (846.5, 3282.9) | 152.2 (64.7, 251.2) | 2.5 (2.1,3.0) |
| DALYs (Disability-Adjusted Life Years) | Republic of Kazakhstan | 4335.8 (1775.4, 7198.7) | 150.3 (61.5, 249.9) | 5075.1 (2189.9, 8213) | 125 (53.9, 202.4) | -0.3 (-0.6,-0.1) |
| DALYs (Disability-Adjusted Life Years) | Kyrgyz Republic | 777.8 (318.4, 1280.5) | 114.4 (46.8, 188.5) | 956.4 (399.3, 1559.5) | 87.6 (36.5, 143.8) | -0.7 (-0.8,-0.5) |
| DALYs (Disability-Adjusted Life Years) | Mongolia | 156.1 (64, 267.7) | 67.5 (27.7, 115.9) | 407.9 (161.1, 702.8) | 78.7 (30.9, 135.7) | 0.4 (0.2,0.5) |
| DALYs (Disability-Adjusted Life Years) | Republic of Tajikistan | 440.8 (178.1, 734.4) | 71 (28.7, 118.5) | 686 (279.6, 1205.7) | 51.3 (20.7, 89.8) | -1.0 (-1.2,-0.8) |
| DALYs (Disability-Adjusted Life Years) | Turkmenistan | 284.6 (115.4, 470.1) | 65 (26.3, 107.6) | 545.9 (218, 924.8) | 58.7 (23.4, 99.6) | -0.3 (-0.8,0.2) |
| DALYs (Disability-Adjusted Life Years) | Republic of Uzbekistan | 1652 (685.6, 2754.4) | 64.7 (26.8, 108.1) | 3773.7 (1497.7, 6495.7) | 61.1 (24.1, 105.1) | -0.2 (-0.6,0.3) |
| DALYs (Disability-Adjusted Life Years) | Republic of Albania | 311.2 (125.5, 533) | 72 (29.1, 123.4) | 812.3 (339.1, 1416.7) | 81.9 (34.2, 143) | 0.7 (0.5,0.9) |
| DALYs (Disability-Adjusted Life Years) | Bosnia and Herzegovina | 1207.4 (494.2, 2028.6) | 127.5 (52, 213.9) | 3068.8 (1286, 5202.1) | 219.6 (92.1, 372.2) | 2.1 (1.8,2.3) |
| DALYs (Disability-Adjusted Life Years) | Republic of Bulgaria | 6362.1 (2728.9, 10460.6) | 224.3 (95.6, 370) | 9986.6 (4226.2, 16960.9) | 321.6 (136.2, 544.8) | 1.5 (1.4,1.7) |
| DALYs (Disability-Adjusted Life Years) | Republic of Croatia | 3125.4 (1343.5, 5115.1) | 230.9 (99.2, 379) | 6100.2 (2619.4, 9888.1) | 309 (132.7, 500.1) | 1.0 (0.8,1.3) |
| DALYs (Disability-Adjusted Life Years) | Czech Republic | 12775.1 (5393.6, 20635.6) | 419.2 (176.9, 677) | 12522.4 (5388.4, 20636.5) | 266.6 (114.6, 439.8) | -1.8 (-2.0,-1.6) |
| DALYs (Disability-Adjusted Life Years) | Hungary | 11014.6 (4483.4, 17559.4) | 335.4 (136.7, 535.6) | 15685.7 (6980.4, 25550.8) | 377.5 (168.7, 614.9) | 0.3 (0.1,0.5) |
| DALYs (Disability-Adjusted Life Years) | North Macedonia | 687.4 (285.2, 1123.9) | 164.6 (68.3, 269.3) | 1691.8 (727.1, 2913.4) | 228.4 (98, 392.6) | 1.1 (0.8,1.4) |
| DALYs (Disability-Adjusted Life Years) | Montenegro | 233.4 (95.9, 384.4) | 166.2 (68.4, 274.1) | 540.6 (238.8, 895) | 245.6 (108.4, 407.3) | 1.4 (1.3,1.5) |
| DALYs (Disability-Adjusted Life Years) | Republic of Poland | 21205.1 (8964.5, 34466.2) | 217.3 (92, 353.7) | 45223.1 (19337.7, 73500.4) | 283.7 (121.2, 461.4) | 0.7 (0.5,0.9) |
| DALYs (Disability-Adjusted Life Years) | Romania | 8234.3 (3359.3, 13559.9) | 127.3 (51.9, 209.8) | 21829 (8964.9, 36925.8) | 272.3 (112.2, 459.7) | 2.2 (2.0,2.4) |
| DALYs (Disability-Adjusted Life Years) | Republic of Serbia | 5668.9 (2358.9, 9865.1) | 225.5 (93.5, 393.3) | 10373.4 (4582.4, 17090.3) | 285.1 (125.9, 469.2) | 0.7 (0.6,0.8) |
| DALYs (Disability-Adjusted Life Years) | Slovak Republic | 4480.8 (1886.9, 7517.9) | 337.4 (142.6, 565.9) | 7289.3 (3171, 12220.9) | 344.1 (149.7, 578.3) | 0.0 (-0.1,0.1) |
| DALYs (Disability-Adjusted Life Years) | Republic of Slovenia | 1302.1 (557.7, 2114.5) | 237.2 (101.6, 384.8) | 1983 (849.4, 3268.3) | 201.1 (86.4, 331.1) | -0.7 (-1.1,-0.4) |
| DALYs (Disability-Adjusted Life Years) | Republic of Belarus | 4955.9 (1992.2, 8154) | 166.6 (67, 274.6) | 8769.5 (3663.6, 15156.1) | 242.3 (101.2, 419.2) | 0.5 (0.2,0.8) |
| DALYs (Disability-Adjusted Life Years) | Republic of Estonia | 935.4 (385.7, 1511.5) | 202.5 (83.5, 327.2) | 1279.1 (542.6, 2130.6) | 215.8 (91.6, 357.9) | -0.1 (-0.2,0.1) |
| DALYs (Disability-Adjusted Life Years) | Republic of Latvia | 1645.2 (695.3, 2687.7) | 204.6 (86.4, 334.2) | 1881.5 (808.4, 3151.3) | 218.8 (94.1, 366.6) | 0.3 (0.1,0.4) |
| DALYs (Disability-Adjusted Life Years) | Republic of Lithuania | 1711.3 (724.6, 2807) | 170.1 (72, 278.4) | 2750 (1179.6, 4561.2) | 217.3 (93.3, 360.2) | 0.7 (0.6,0.9) |
| DALYs (Disability-Adjusted Life Years) | Republic of Moldova | 2040.3 (888.5, 3308.6) | 201.2 (87.5, 327) | 3703.7 (1582.9, 5959.1) | 277.1 (118.4, 446.3) | 1.6 (1.1,2.0) |
| DALYs (Disability-Adjusted Life Years) | Russian Federation | 75761.6 (32539.4, 120952.2) | 182.5 (78.3, 291) | 135067.5 (57648.1, 213076.3) | 251.8 (107.4, 396.8) | 0.8 (0.6,1.0) |
| DALYs (Disability-Adjusted Life Years) | Ukraine | 35451 (14759, 57749.6) | 215 (89.6, 350.7) | 37712.9 (16115.1, 64855.8) | 219.2 (93.5, 377) | -0.1 (-0.2,0.0) |
| DALYs (Disability-Adjusted Life Years) | Brunei Darussalam | 24.3 (8.8, 43.8) | 111.3 (39.6, 201.3) | 119.3 (46.4, 203.8) | 140.5 (53.9, 240.6) | 1.2 (1.0,1.4) |
| DALYs (Disability-Adjusted Life Years) | Japan | 27530 (10217.1, 45002.4) | 73.1 (27.1, 119.5) | 59930.4 (22839.7, 98389.2) | 81.8 (31.4, 133.8) | 0.2 (0.2,0.3) |
| DALYs (Disability-Adjusted Life Years) | Republic of Korea | 2755.2 (892.4, 4814.3) | 42.4 (13.6, 74.6) | 13039.4 (4720.7, 22452.9) | 61.7 (22.3, 106.4) | 1.1 (0.9,1.4) |
| DALYs (Disability-Adjusted Life Years) | Republic of Singapore | 379.2 (132.3, 649.3) | 78 (26.9, 133.9) | 1671.4 (663.5, 2779.6) | 87 (34.4, 145) | 0.2 (-0.0,0.4) |
| DALYs (Disability-Adjusted Life Years) | Australia | 9307.7 (3783.6, 14855.9) | 220.5 (89.5, 352.1) | 17250.6 (7371.1, 27585.8) | 175.7 (75.1, 280.5) | -1.0 (-1.1,-0.9) |
| DALYs (Disability-Adjusted Life Years) | New Zealand | 2550.7 (1035.3, 4180.4) | 303.1 (123.2, 496.4) | 3919.7 (1672.8, 6427.8) | 209.4 (89.6, 342.4) | -1.3 (-1.4,-1.2) |
| DALYs (Disability-Adjusted Life Years) | Principality of Andorra | 24.9 (9, 47.4) | 199.2 (72.2, 378.7) | 52 (19.8, 96.5) | 151.9 (57.7, 281.6) | -0.5 (-0.8,-0.3) |
| DALYs (Disability-Adjusted Life Years) | Republic of Austria | 5323.6 (2137.3, 8909.4) | 206.9 (83.4, 345.8) | 4670.8 (1916.7, 7970.7) | 117.4 (48.3, 199.8) | -1.9 (-1.9,-1.8) |
| DALYs (Disability-Adjusted Life Years) | Kingdom of Belgium | 5842.7 (2357.3, 9586.1) | 171.7 (69.2, 282) | 6947.1 (2836.1, 11746.9) | 135.2 (55.4, 228.2) | -0.7 (-0.8,-0.6) |
| DALYs (Disability-Adjusted Life Years) | Republic of Cyprus | 184.7 (71.2, 319.4) | 110.7 (42.1, 192.8) | 521.8 (210.2, 923.1) | 117.3 (47.3, 207.9) | 0.5 (0.4,0.7) |
| DALYs (Disability-Adjusted Life Years) | Kingdom of Denmark | 3041.7 (1233.4, 5025.3) | 176.7 (71.7, 292.4) | 4455.1 (1830.7, 7575.2) | 169.6 (69.9, 288.2) | -0.5 (-0.7,-0.2) |
| DALYs (Disability-Adjusted Life Years) | Republic of Finland | 1927.6 (779.8, 3228.4) | 123.6 (49.9, 207.2) | 3383.8 (1437.5, 5703.5) | 122.3 (52.2, 206) | -0.1 (-0.2,-0.0) |
| DALYs (Disability-Adjusted Life Years) | French Republic | 28395.6 (11040.3, 47000.3) | 156.9 (60.9, 260) | 44400.4 (18450, 74735.7) | 143.4 (59.9, 241.7) | -0.2 (-0.3,-0.1) |
| DALYs (Disability-Adjusted Life Years) | Federal Republic of Germany | 69270.4 (28489.4, 113480.4) | 246.1 (101.3, 403.2) | 66836.5 (27331, 112147.4) | 159.4 (65.5, 266.2) | -1.7 (-1.8,-1.6) |
| DALYs (Disability-Adjusted Life Years) | Hellenic Republic | 4137.6 (1656.1, 6883.2) | 122 (48.8, 203.2) | 8156.8 (3348.7, 13482.7) | 159.1 (65.8, 262.1) | 0.6 (0.4,0.8) |
| DALYs (Disability-Adjusted Life Years) | Republic of Iceland | 94.5 (38.4, 155.5) | 153.2 (62.3, 252.1) | 159.6 (67.2, 269.4) | 123.8 (52.2, 208.5) | -0.6 (-0.7,-0.5) |
| DALYs (Disability-Adjusted Life Years) | Ireland | 1857.3 (747.4, 3088.8) | 211.9 (85.1, 352.7) | 2693.4 (1128.5, 4528.9) | 155.6 (65.2, 261.7) | -0.9 (-1.0,-0.8) |
| DALYs (Disability-Adjusted Life Years) | State of Israel | 1933.6 (801.4, 3188.6) | 183.5 (76, 303.4) | 3508 (1502.8, 5913.1) | 129.6 (55.6, 218.1) | -1.7 (-2.0,-1.4) |
| DALYs (Disability-Adjusted Life Years) | Republic of Italy | 29327.6 (11840.2, 48680.6) | 148.7 (60, 246.9) | 44580.7 (18686.9, 73368.8) | 139.9 (58.9, 229.4) | -0.3 (-0.5,-0.2) |
| DALYs (Disability-Adjusted Life Years) | Grand Duchy of Luxembourg | 258.4 (104.7, 424.3) | 216.4 (87.6, 355.8) | 354.7 (150.5, 583.2) | 150.2 (63.8, 247.1) | -1.1 (-1.3,-0.9) |
| DALYs (Disability-Adjusted Life Years) | Republic of Malta | 121.3 (48.5, 202.1) | 130.3 (51.9, 217.1) | 291.9 (124, 487.8) | 136.8 (58.4, 227.9) | 0.1 (-0.0,0.2) |
| DALYs (Disability-Adjusted Life Years) | Kingdom of the Netherlands | 8041 (3257.3, 13372.6) | 185 (75, 308.1) | 15628.4 (6434.5, 26170) | 201.3 (82.9, 336.7) | 0.4 (0.2,0.6) |
| DALYs (Disability-Adjusted Life Years) | Kingdom of Norway | 2609.7 (1065, 4312.3) | 180.3 (74, 298.1) | 3379.5 (1428.5, 5508.4) | 152 (64.2, 247.5) | -0.7 (-0.8,-0.6) |
| DALYs (Disability-Adjusted Life Years) | Portuguese Republic | 5030.7 (2004.5, 8442.3) | 163.5 (65, 274.8) | 9369.8 (3958.5, 15585) | 178.7 (76.2, 296.7) | 0.3 (0.1,0.6) |
| DALYs (Disability-Adjusted Life Years) | Kingdom of Spain | 21338.4 (8844.8, 35602.4) | 176.4 (73.2, 294.3) | 43334.9 (18308, 71688.4) | 205.6 (87, 338.6) | 0.5 (0.3,0.7) |
| DALYs (Disability-Adjusted Life Years) | Kingdom of Sweden | 4804.4 (1927.2, 8077.2) | 149.9 (60.4, 252) | 6212.3 (2575.7, 10401.8) | 130.6 (54.3, 218.4) | -0.3 (-0.5,-0.1) |
| DALYs (Disability-Adjusted Life Years) | Swiss Confederation | 2571.5 (1054.9, 4211.4) | 114.5 (47, 187.6) | 3635.1 (1462.1, 6114.3) | 90.7 (36.7, 152.3) | -0.9 (-1.0,-0.7) |
| DALYs (Disability-Adjusted Life Years) | United Kingdom of Great Britain and Northern Ireland | 46748.6 (19960.8, 75880) | 239.3 (102.1, 388) | 51599.8 (22330.6, 83175.9) | 180.7 (78.5, 290.1) | -1.0 (-1.1,-0.9) |
| DALYs (Disability-Adjusted Life Years) | Argentine Republic | 14503.1 (6090.9, 24162.5) | 203.7 (85.5, 339.7) | 30286.7 (13222.5, 50256.5) | 249.5 (109, 413.9) | 1.0 (0.8,1.2) |
| DALYs (Disability-Adjusted Life Years) | Republic of Chile | 2670.1 (1107.3, 4438.8) | 124.3 (51.4, 206.8) | 9909.1 (4330.8, 16175.4) | 173.7 (75.9, 283.5) | 1.4 (1.2,1.5) |
| DALYs (Disability-Adjusted Life Years) | Eastern Republic of Uruguay | 2065.9 (842.6, 3449.5) | 239.9 (97.9, 401) | 3716.9 (1596.8, 6177.9) | 310.9 (134, 514.8) | 0.7 (0.6,0.8) |
| DALYs (Disability-Adjusted Life Years) | Canada | 14540.2 (6153.6, 23563.6) | 206.5 (87.4, 335) | 25696 (11282.4, 41849) | 161.5 (71, 262.3) | -0.6 (-0.8,-0.5) |
| DALYs (Disability-Adjusted Life Years) | United States of America | 149311.2 (64329.1, 238941.1) | 219.2 (94.7, 350) | 234963.6 (105817.6, 362953.8) | 186 (83.8, 286.6) | -0.7 (-0.8,-0.6) |
| DALYs (Disability-Adjusted Life Years) | Antigua and Barbuda | 10 (4, 16.7) | 89.2 (35.8, 148.4) | 39.3 (16.7, 63.4) | 164.4 (69.8, 266.3) | 2.0 (1.8,2.1) |
| DALYs (Disability-Adjusted Life Years) | Commonwealth of the Bahamas | 48.5 (20.1, 79.4) | 146.4 (60.7, 240.2) | 208.8 (86.1, 345.3) | 226.7 (93, 375.6) | 1.8 (1.6,1.9) |
| DALYs (Disability-Adjusted Life Years) | Barbados | 95.9 (38.4, 159.7) | 157.6 (63.5, 261.2) | 322.5 (138.9, 543) | 277.5 (119.6, 467.1) | 2.2 (1.9,2.5) |
| DALYs (Disability-Adjusted Life Years) | Belize | 12.3 (5.1, 20.3) | 61.9 (25.8, 102.4) | 78.9 (35.9, 125.5) | 120.8 (54.8, 192.7) | 2.2 (1.7,2.7) |
| DALYs (Disability-Adjusted Life Years) | Republic of Cuba | 2157.7 (849.9, 3520.3) | 98 (38.6, 159.8) | 7725.5 (3143, 13339.6) | 176.1 (71.6, 303.8) | 2.1 (2.0,2.2) |
| DALYs (Disability-Adjusted Life Years) | Commonwealth of Dominica | 17.2 (7, 29.1) | 133.9 (54.1, 226.5) | 39.4 (16.9, 65.6) | 208.5 (89, 347.2) | 1.5 (1.5,1.6) |
| DALYs (Disability-Adjusted Life Years) | Dominican Republic | 316.5 (118.5, 561.3) | 39.8 (14.8, 70.8) | 1794.8 (703.1, 3208.3) | 82.4 (32.2, 147.4) | 2.7 (2.6,2.9) |
| DALYs (Disability-Adjusted Life Years) | Grenada | 13.8 (5.3, 23.3) | 93.8 (36, 158.3) | 44.1 (18.3, 74.5) | 169.3 (69.6, 286.5) | 2.3 (2.2,2.4) |
| DALYs (Disability-Adjusted Life Years) | Republic of Guyana | 65.3 (25, 110.9) | 80.3 (30.6, 136.6) | 192.4 (77.8, 333.8) | 130.8 (52.9, 227.3) | 1.9 (1.7,2.2) |
| DALYs (Disability-Adjusted Life Years) | Republic of Haiti | 261.5 (76.8, 503.5) | 36.1 (10.5, 69.6) | 989.3 (336.5, 1884.3) | 62 (21, 118.1) | 2.0 (1.9,2.2) |
| DALYs (Disability-Adjusted Life Years) | Jamaica | 304.3 (122.6, 507.1) | 79.7 (32.2, 132.6) | 1197.3 (510.6, 2017.9) | 176.7 (75.3, 297.5) | 2.7 (2.3,3.1) |
| DALYs (Disability-Adjusted Life Years) | Saint Lucia | 13.7 (5.3, 23.2) | 74.4 (28.5, 125.5) | 54.3 (22.2, 91.6) | 100.3 (41, 169.3) | 0.7 (0.5,0.9) |
| DALYs (Disability-Adjusted Life Years) | Saint Vincent and the Grenadines | 9.1 (3.4, 15.6) | 59.2 (22.1, 101.8) | 34 (13.9, 57.3) | 106 (43.3, 178.7) | 1.9 (1.7,2.0) |
| DALYs (Disability-Adjusted Life Years) | Republic of Suriname | 39.8 (15, 69) | 70.1 (26.2, 121.6) | 171.2 (67, 302.2) | 118.1 (46, 208.9) | 2.0 (1.8,2.2) |
| DALYs (Disability-Adjusted Life Years) | Republic of Trinidad and Tobago | 217.2 (88.9, 355.9) | 122.5 (50.2, 200.8) | 758.6 (318.1, 1275.9) | 173.1 (72.6, 291.3) | 1.0 (0.9,1.2) |
| DALYs (Disability-Adjusted Life Years) | Plurinational State of Bolivia | 610.6 (202.5, 1180.4) | 88.2 (29.2, 170.6) | 2713.4 (1030.1, 4971.5) | 136.4 (51.7, 250.4) | 1.4 (1.4,1.5) |
| DALYs (Disability-Adjusted Life Years) | Republic of Ecuador | 581.1 (234.5, 967.7) | 52.3 (21, 87.3) | 4012.4 (1666.7, 6736) | 113 (46.9, 189.9) | 2.8 (2.5,3.2) |
| DALYs (Disability-Adjusted Life Years) | Republic of Peru | 1541.7 (619.2, 2631.9) | 60.3 (24.1, 103) | 6758.9 (2825.4, 12061.6) | 93.2 (39, 166.3) | 1.3 (1.1,1.4) |
| DALYs (Disability-Adjusted Life Years) | Republic of Colombia | 2510.5 (1000.4, 4213.9) | 67.7 (26.8, 113.4) | 14354.1 (6020.3, 24103.4) | 117.7 (49.4, 197.7) | 1.6 (1.5,1.8) |
| DALYs (Disability-Adjusted Life Years) | Republic of Costa Rica | 295.2 (119.4, 493.4) | 79.8 (32.2, 133.5) | 2184.1 (911.5, 3604.2) | 178.9 (74.7, 295.3) | 2.9 (2.7,3.1) |
| DALYs (Disability-Adjusted Life Years) | Republic of El Salvador | 301.9 (123.7, 501.1) | 47.4 (19.4, 78.6) | 1405.9 (584.5, 2413.1) | 106.1 (44.2, 182.2) | 2.6 (2.4,2.8) |
| DALYs (Disability-Adjusted Life Years) | Republic of Guatemala | 255.3 (103.4, 420) | 36 (14.6, 59.5) | 1777.7 (755.4, 2977.7) | 75.4 (32, 126.4) | 2.4 (2.1,2.7) |
| DALYs (Disability-Adjusted Life Years) | Republic of Honduras | 147 (55.9, 253.9) | 33.1 (12.6, 57.2) | 975.8 (381.6, 1737.1) | 70.6 (27.6, 125.8) | 2.7 (2.5,2.8) |
| DALYs (Disability-Adjusted Life Years) | United Mexican States | 4775 (2010.6, 7784.3) | 53.5 (22.5, 87.4) | 32963.8 (14620, 52498.3) | 116.8 (51.7, 186.2) | 2.6 (2.5,2.7) |
| DALYs (Disability-Adjusted Life Years) | Republic of Nicaragua | 135.7 (54.4, 227.7) | 41.8 (16.7, 70.4) | 810.8 (331.1, 1384.5) | 76.6 (31.3, 131.1) | 2.4 (2.1,2.7) |
| DALYs (Disability-Adjusted Life Years) | Republic of Panama | 263.9 (108.7, 431.8) | 84.2 (34.7, 137.8) | 1472.2 (646.1, 2452.3) | 152.7 (67, 254.3) | 2.2 (2.1,2.3) |
| DALYs (Disability-Adjusted Life Years) | Bolivarian Republic of Venezuela | 1733.3 (712.6, 2861.2) | 84.7 (34.8, 140.2) | 9070 (3771.8, 15494.6) | 134.9 (56.1, 230.6) | 1.4 (1.3,1.6) |
| DALYs (Disability-Adjusted Life Years) | Federative Republic of Brazil | 14268.5 (5813.7, 23181.9) | 74.3 (30.1, 120.7) | 73726.9 (31392.1, 117926.2) | 132.3 (56.3, 211.7) | 1.9 (1.7,2.0) |
| DALYs (Disability-Adjusted Life Years) | Republic of Paraguay | 235.9 (95, 407.6) | 50 (20.1, 86.5) | 1537.1 (630.3, 2748.1) | 121.4 (49.7, 217.2) | 3.2 (3.0,3.4) |
| DALYs (Disability-Adjusted Life Years) | People's Democratic Republic of Algeria | 771.9 (297.5, 1377.2) | 30.6 (11.7, 54.9) | 3653 (1500.9, 6366.7) | 49.2 (20.2, 85.9) | 1.7 (1.6,1.8) |
| DALYs (Disability-Adjusted Life Years) | Kingdom of Bahrain | 42.6 (17.1, 71.7) | 117.1 (46.7, 198) | 267 (109.4, 467.2) | 146 (59.6, 253) | 0.2 (-0.0,0.5) |
| DALYs (Disability-Adjusted Life Years) | Arab Republic of Egypt | 3614.8 (1495.3, 5954.5) | 59.4 (24.4, 98.7) | 20445.3 (8864.4, 33925.9) | 145.8 (63.3, 242.1) | 3.6 (3.3,3.9) |
| DALYs (Disability-Adjusted Life Years) | Islamic Republic of Iran | 2804 (1161.1, 4644.7) | 47.3 (19.4, 78.6) | 15110.9 (6495.6, 24009.2) | 90.3 (38.8, 143.7) | 2.5 (2.3,2.8) |
| DALYs (Disability-Adjusted Life Years) | Republic of Iraq | 1035 (416.6, 1765.7) | 61 (24.5, 104.1) | 4568.5 (1899.5, 8057.4) | 87.8 (36.6, 154.2) | 1.2 (1.0,1.4) |
| DALYs (Disability-Adjusted Life Years) | Hashemite Kingdom of Jordan | 404.1 (165.8, 698.7) | 134.1 (54.8, 232.9) | 2349.1 (993.2, 4019.1) | 144.1 (60.8, 247) | 0.2 (-0.0,0.5) |
| DALYs (Disability-Adjusted Life Years) | State of Kuwait | 86.5 (36.9, 140.7) | 70.6 (29.8, 115.8) | 878.4 (405.2, 1451.5) | 146.7 (67.3, 243.2) | 2.9 (2.4,3.4) |
| DALYs (Disability-Adjusted Life Years) | Lebanese Republic | 632.4 (234.5, 1142.2) | 132.1 (49.1, 239) | 1948.2 (805.1, 3256.8) | 149.7 (62.1, 250) | 0.8 (0.6,1.0) |
| DALYs (Disability-Adjusted Life Years) | State of Libya | 503.9 (195.8, 902.2) | 123.8 (47.9, 221.9) | 2475.1 (1010.8, 4386.5) | 219.3 (89.4, 388.3) | 2.2 (2.0,2.3) |
| DALYs (Disability-Adjusted Life Years) | Kingdom of Morocco | 1356.6 (489.2, 2457.2) | 43.8 (15.8, 79.3) | 7133.3 (2626.6, 13034.5) | 91.9 (33.8, 167.5) | 2.7 (2.5,2.8) |
| DALYs (Disability-Adjusted Life Years) | Palestine | 358 (147.1, 639.5) | 192.8 (78.9, 344.1) | 1217.4 (499.5, 2008.7) | 224.7 (92, 371.2) | 0.6 (0.4,0.7) |
| DALYs (Disability-Adjusted Life Years) | Sultanate of Oman | 45 (17.2, 81.3) | 31.7 (12.1, 57) | 219.3 (89.8, 379.6) | 53.7 (21.6, 92.6) | 2.2 (1.9,2.5) |
| DALYs (Disability-Adjusted Life Years) | State of Qatar | 31.5 (12.6, 53.6) | 156.3 (62.5, 269.9) | 292.8 (125.8, 507.5) | 178.7 (77.4, 308.3) | 0.7 (0.1,1.4) |
| DALYs (Disability-Adjusted Life Years) | Kingdom of Saudi Arabia | 689.1 (269.8, 1235.3) | 56.4 (22.1, 101.2) | 5432.4 (2328.8, 8879.2) | 128.5 (54.5, 210.7) | 2.9 (2.5,3.3) |
| DALYs (Disability-Adjusted Life Years) | Syrian Arab Republic | 714.8 (285.4, 1227.6) | 62.1 (24.9, 106.6) | 3026.2 (1212.3, 5295.2) | 101.2 (40.4, 176) | 1.4 (1.2,1.6) |
| DALYs (Disability-Adjusted Life Years) | Republic of Tunisia | 584.5 (224.1, 1018.2) | 52.6 (20, 92) | 2624.3 (1046.5, 4788.8) | 88.7 (35.4, 161.6) | 1.6 (1.5,1.6) |
| DALYs (Disability-Adjusted Life Years) | Republic of Turkey | 11857 (4771.5, 20096.2) | 155.4 (62.6, 263) | 37358.7 (16000.1, 63594.8) | 180 (76.9, 306.9) | 0.3 (0.0,0.7) |
| DALYs (Disability-Adjusted Life Years) | United Arab Emirates | 144.3 (50.8, 272.9) | 169.9 (59.7, 319.3) | 1235.9 (492.5, 2178.1) | 192.5 (79, 334.8) | 1.9 (1.4,2.3) |
| DALYs (Disability-Adjusted Life Years) | Republic of Yemen | 321.4 (105.6, 634.2) | 29.3 (9.6, 57.9) | 1707.6 (601.4, 3136) | 56.9 (20.1, 104.3) | 2.3 (2.2,2.4) |
| DALYs (Disability-Adjusted Life Years) | Islamic Republic of Afghanistan | 1383.7 (305.4, 2856.4) | 81.9 (18.5, 168.9) | 2291.2 (677.3, 4599.2) | 109.7 (33.6, 218.9) | 1.0 (0.9,1.1) |
| DALYs (Disability-Adjusted Life Years) | People's Republic of Bangladesh | 603.2 (156.8, 1218.4) | 5.9 (1.5, 11.8) | 3544.6 (1151.7, 6707.8) | 11.4 (3.7, 21.7) | 2.5 (2.4,2.7) |
| DALYs (Disability-Adjusted Life Years) | Kingdom of Bhutan | 14.4 (4.9, 28.4) | 25 (8.4, 49.2) | 44.1 (16.7, 84.1) | 33.5 (12.7, 63.9) | 0.9 (0.9,1.0) |
| DALYs (Disability-Adjusted Life Years) | Republic of India | 8229.7 (2505.3, 14512.7) | 7.6 (2.3, 13.5) | 50737.9 (19362.7, 83838.3) | 18.8 (7.2, 31.2) | 3.0 (2.9,3.1) |
| DALYs (Disability-Adjusted Life Years) | Federal Democratic Republic of Nepal | 160.6 (44.5, 335.4) | 7.2 (1.9, 14.9) | 805.9 (279.3, 1517.1) | 15 (5.2, 28.3) | 2.7 (2.3,3.1) |
| DALYs (Disability-Adjusted Life Years) | Islamic Republic of Pakistan | 1948 (628.6, 3521.7) | 15.8 (5.1, 28.6) | 10947 (4300.8, 18722.9) | 40.5 (15.9, 69.1) | 3.1 (2.9,3.4) |
| DALYs (Disability-Adjusted Life Years) | Republic of Angola | 195.6 (62.5, 378.8) | 21.6 (6.7, 41.7) | 1234.5 (439.9, 2356.3) | 45.8 (16.2, 87.7) | 2.4 (2.3,2.5) |
| DALYs (Disability-Adjusted Life Years) | Central African Republic | 64.7 (20.1, 133.1) | 23.7 (7.2, 48.6) | 237.5 (79.4, 469.5) | 44.6 (14.8, 88.2) | 2.1 (2.1,2.1) |
| DALYs (Disability-Adjusted Life Years) | Republic of the Congo | 108 (35.6, 208) | 43.2 (14, 83.2) | 453.9 (168.1, 820.5) | 73.1 (26.9, 132.8) | 1.5 (1.4,1.6) |
| DALYs (Disability-Adjusted Life Years) | Democratic Republic of the Congo | 704.6 (225.1, 1316.1) | 19.9 (6.3, 37.5) | 3411 (1194.2, 6379.2) | 42.5 (14.8, 80.2) | 2.6 (2.3,2.8) |
| DALYs (Disability-Adjusted Life Years) | Republic of Equatorial Guinea | 18.1 (6.1, 34.9) | 39.6 (13.3, 76.5) | 101.9 (37.4, 198) | 95.9 (35.1, 185.4) | 3.0 (2.9,3.2) |
| DALYs (Disability-Adjusted Life Years) | Gabonese Republic | 112.8 (37, 230.8) | 87.2 (28.5, 178.3) | 338.3 (132.2, 611.7) | 143.4 (56, 259.6) | 1.5 (1.5,1.6) |
| DALYs (Disability-Adjusted Life Years) | Republic of Burundi | 93.3 (25.1, 187.9) | 18.4 (4.9, 37.1) | 236.4 (73.9, 463.7) | 22.2 (6.9, 43.6) | 0.1 (-0.1,0.4) |
| DALYs (Disability-Adjusted Life Years) | Union of the Comoros | 12.7 (4.1, 23.9) | 29 (9.3, 54.8) | 67.5 (23.9, 126.2) | 63.5 (22.4, 118.6) | 2.5 (2.4,2.6) |
| DALYs (Disability-Adjusted Life Years) | Republic of Djibouti | 5.3 (1.5, 10.9) | 17.4 (4.8, 35.7) | 53.3 (16, 104.9) | 37.2 (11.1, 73.5) | 2.4 (2.4,2.5) |
| DALYs (Disability-Adjusted Life Years) | State of Eritrea | 44 (13.2, 88.5) | 16.2 (4.7, 32.9) | 210 (68, 404.6) | 33.7 (10.8, 65.4) | 2.4 (2.4,2.5) |
| DALYs (Disability-Adjusted Life Years) | Federal Democratic Republic of Ethiopia | 2093.4 (613.2, 3894.9) | 46.3 (13.3, 86.3) | 4237.8 (1427.9, 7386.4) | 46.3 (15.4, 81) | -0.4 (-0.7,-0.2) |
| DALYs (Disability-Adjusted Life Years) | Republic of Kenya | 317.7 (107.8, 563.1) | 17.3 (5.8, 30.7) | 2382.5 (931.2, 4052.8) | 46.5 (18, 79.3) | 3.7 (3.5,3.9) |
| DALYs (Disability-Adjusted Life Years) | Republic of Madagascar | 211.8 (65.7, 417) | 19 (5.8, 37.3) | 832.3 (286.2, 1565.5) | 33.8 (11.6, 63.7) | 1.9 (1.7,2.1) |
| DALYs (Disability-Adjusted Life Years) | Republic of Malawi | 84.6 (24, 158.9) | 10.3 (2.9, 19.3) | 349.5 (117.2, 669.3) | 22.2 (7.4, 42.3) | 2.3 (2.1,2.5) |
| DALYs (Disability-Adjusted Life Years) | Republic of Mauritius | 71 (27.8, 118.1) | 44.2 (17.2, 73.8) | 517.7 (214.3, 846.2) | 121.1 (50.1, 198.1) | 2.7 (2.5,2.9) |
| DALYs (Disability-Adjusted Life Years) | Republic of Mozambique | 95.5 (29.2, 181.6) | 7.5 (2.2, 14.3) | 408.5 (137, 750.3) | 17.3 (5.7, 32) | 3.2 (3.1,3.4) |
| DALYs (Disability-Adjusted Life Years) | Republic of Rwanda | 173.9 (50.9, 346.7) | 27 (7.8, 53.7) | 494.5 (152.9, 973) | 35.8 (11, 70.6) | 0.1 (-0.3,0.4) |
| DALYs (Disability-Adjusted Life Years) | Republic of Seychelles | 13.3 (4.8, 22.4) | 108.6 (39.6, 183.3) | 55.1 (22.7, 90.8) | 205.5 (84.1, 340.2) | 2.2 (1.9,2.5) |
| DALYs (Disability-Adjusted Life Years) | Federal Republic of Somalia | 153.3 (44.7, 316.3) | 26.9 (7.7, 55.3) | 557.1 (169.9, 1119.5) | 41.6 (12.6, 83.6) | 1.5 (1.4,1.5) |
| DALYs (Disability-Adjusted Life Years) | United Republic of Tanzania | 851.2 (290.2, 1579.3) | 35.2 (11.9, 65.2) | 3581 (1361.5, 6472.5) | 65.4 (24.8, 118.4) | 2.0 (2.0,2.1) |
| DALYs (Disability-Adjusted Life Years) | Republic of Uganda | 370.8 (116.4, 691.5) | 25.8 (8, 48.4) | 1578.1 (554.2, 2893.4) | 48.1 (16.7, 88.2) | 1.4 (1.2,1.7) |
| DALYs (Disability-Adjusted Life Years) | Republic of Zambia | 199.5 (67, 375.1) | 31.2 (10.3, 58.4) | 1334.2 (446.3, 2931.9) | 87.4 (29.1, 188) | 3.2 (3.1,3.3) |
| DALYs (Disability-Adjusted Life Years) | Republic of Botswana | 55.8 (18.8, 111.8) | 46.9 (15.8, 94.4) | 270.5 (99.9, 505.8) | 90.4 (33.5, 167.6) | 2.5 (2.2,2.7) |
| DALYs (Disability-Adjusted Life Years) | Kingdom of Lesotho | 87.1 (32.3, 161.4) | 48 (17.7, 89.4) | 331.2 (121.6, 632.6) | 142.1 (51.9, 272) | 4.5 (4.0,5.0) |
| DALYs (Disability-Adjusted Life Years) | Republic of Namibia | 41.5 (15.1, 75.3) | 29.3 (10.6, 53.3) | 184.5 (70, 322.6) | 62 (23.6, 108.4) | 2.4 (2.2,2.7) |
| DALYs (Disability-Adjusted Life Years) | Republic of South Africa | 3166.7 (1300.5, 5235.3) | 72.8 (29.8, 120.8) | 14016.3 (5935.5, 22125.6) | 139.7 (59, 220.9) | 2.3 (2.0,2.5) |
| DALYs (Disability-Adjusted Life Years) | Kingdom of Eswatini | 58.6 (22.4, 109.1) | 100.8 (38.3, 188.2) | 243 (91.5, 449) | 209.4 (79.7, 385) | 2.8 (2.2,3.3) |
| DALYs (Disability-Adjusted Life Years) | Republic of Zimbabwe | 378.5 (134.7, 685.9) | 42.7 (15, 77.7) | 1763.8 (704.3, 3118.6) | 117.9 (46.7, 208.5) | 4.0 (3.4,4.5) |
| DALYs (Disability-Adjusted Life Years) | Republic of Benin | 100 (36.9, 181) | 23.5 (8.6, 42.5) | 404.5 (147.6, 749) | 36.7 (13.3, 67.9) | 1.6 (1.5,1.7) |
| DALYs (Disability-Adjusted Life Years) | Burkina Faso | 97.5 (27.8, 185.8) | 10.1 (2.8, 19.3) | 356.4 (107.9, 684.6) | 17.7 (5.3, 34) | 2.1 (1.9,2.2) |
| DALYs (Disability-Adjusted Life Years) | Republic of Cameroon | 436.4 (166, 769.8) | 45.1 (17, 79.3) | 2056.1 (751.7, 3827.9) | 78.9 (28.8, 146.1) | 2.0 (1.9,2.0) |
| DALYs (Disability-Adjusted Life Years) | Republic of Cabo Verde | 7.1 (2.5, 13) | 14.1 (4.9, 25.8) | 57.7 (21.8, 102.7) | 60.8 (22.8, 107.9) | 4.2 (3.8,4.6) |
| DALYs (Disability-Adjusted Life Years) | Republic of Chad | 87.9 (29.6, 167.2) | 14.6 (4.9, 27.7) | 350.4 (126.3, 645) | 28.3 (10.1, 52.4) | 2.3 (2.2,2.4) |
| DALYs (Disability-Adjusted Life Years) | Republic of Côte d'Ivoire | 173.5 (64.7, 314.8) | 19.7 (7.3, 35.8) | 716.2 (265, 1347.9) | 29.8 (11.1, 55.6) | 1.3 (1.2,1.4) |
| DALYs (Disability-Adjusted Life Years) | Republic of the Gambia | 10.7 (3.7, 19.8) | 14.1 (4.8, 26.1) | 47.9 (17.3, 88) | 23 (8.3, 42.4) | 1.4 (1.3,1.6) |
| DALYs (Disability-Adjusted Life Years) | Republic of Ghana | 254.3 (88.8, 478.7) | 18.5 (6.4, 34.8) | 1689.2 (614.4, 3101.3) | 48.2 (17.3, 88.4) | 3.3 (3.2,3.3) |
| DALYs (Disability-Adjusted Life Years) | Republic of Guinea | 111.3 (38.8, 208.6) | 15.3 (5.3, 28.6) | 303.2 (103.3, 567.5) | 24.9 (8.5, 46.5) | 1.7 (1.6,1.7) |
| DALYs (Disability-Adjusted Life Years) | Republic of Guinea-Bissau | 23.9 (7.9, 46.9) | 27.3 (8.9, 53.4) | 76.4 (28.3, 142.7) | 48.8 (17.9, 91) | 2.1 (2.0,2.1) |
| DALYs (Disability-Adjusted Life Years) | Republic of Liberia | 80.1 (29.6, 143.5) | 32.1 (11.8, 57.6) | 226.4 (79.9, 440.5) | 51.6 (18.2, 100.2) | 1.8 (1.6,2.0) |
| DALYs (Disability-Adjusted Life Years) | Republic of Mali | 205.1 (68.9, 376) | 22.7 (7.5, 41.6) | 573.3 (193.3, 1075.5) | 29.5 (9.8, 55.4) | 1.0 (0.8,1.1) |
| DALYs (Disability-Adjusted Life Years) | Islamic Republic of Mauritania | 98.1 (36.8, 179.3) | 46.3 (17.3, 84.7) | 313.5 (117.8, 555) | 69.8 (26.2, 123.3) | 1.3 (1.2,1.5) |
| DALYs (Disability-Adjusted Life Years) | Republic of the Niger | 87.9 (28.8, 168.7) | 14.6 (4.7, 28.2) | 369.4 (122.5, 727.8) | 20.5 (6.7, 40.4) | 1.2 (1.1,1.3) |
| DALYs (Disability-Adjusted Life Years) | Federal Republic of Nigeria | 2099.8 (785.2, 3586.3) | 22.4 (8.4, 38.2) | 8617.5 (3367.7, 14550.2) | 45.6 (17.9, 76.9) | 2.5 (2.4,2.5) |
| DALYs (Disability-Adjusted Life Years) | Democratic Republic of Sao Tome and Principe | 4.8 (1.7, 8.6) | 34.1 (11.9, 60.8) | 17.6 (6.6, 30.9) | 75.9 (28.5, 133.3) | 2.7 (2.6,2.7) |
| DALYs (Disability-Adjusted Life Years) | Republic of Senegal | 169.1 (59, 309.2) | 24.2 (8.4, 44.3) | 707 (252.5, 1288) | 42.6 (15.3, 77.5) | 2.0 (1.8,2.1) |
| DALYs (Disability-Adjusted Life Years) | Republic of Sierra Leone | 67.7 (20.6, 129.2) | 15.6 (4.7, 29.8) | 233.8 (80.2, 441.1) | 29.4 (10, 55.4) | 2.4 (2.2,2.5) |
| DALYs (Disability-Adjusted Life Years) | Togolese Republic | 48.6 (16.7, 90.2) | 18.6 (6.4, 34.6) | 321.8 (113.7, 604.9) | 40 (14, 75.5) | 2.6 (2.6,2.7) |
| DALYs (Disability-Adjusted Life Years) | American Samoa | 9.5 (4, 15.9) | 191.7 (80.1, 322.3) | 26.3 (12, 43.6) | 236.3 (107.2, 391.5) | 0.8 (0.7,0.9) |
| DALYs (Disability-Adjusted Life Years) | Bermuda | 36.9 (15.1, 61.8) | 273 (111.6, 457.1) | 74.6 (32.5, 122.9) | 244.5 (107, 402) | -0.2 (-0.4,-0.1) |
| DALYs (Disability-Adjusted Life Years) | Cook Islands | 2.6 (1.1, 4.5) | 95.6 (41, 164.9) | 5 (2.2, 8.4) | 84.3 (37.5, 142.6) | -0.6 (-0.7,-0.4) |
| DALYs (Disability-Adjusted Life Years) | Greenland | 31.3 (13, 53.1) | 441.4 (182.6, 749.8) | 48.6 (20.2, 84.6) | 290 (119.9, 504.3) | -1.4 (-1.5,-1.3) |
| DALYs (Disability-Adjusted Life Years) | Guam | 25.1 (10.3, 42.3) | 151.7 (61.7, 258.2) | 65.9 (27.9, 109.3) | 136.8 (57.9, 226.6) | 0.2 (-0.2,0.5) |
| DALYs (Disability-Adjusted Life Years) | Principality of Monaco | 34.8 (13.5, 60.8) | 232.2 (89.7, 405.8) | 60.6 (25.3, 107.4) | 282.1 (118.2, 497.2) | 0.7 (0.6,0.8) |
| DALYs (Disability-Adjusted Life Years) | Republic of Nauru | 2.4 (0.9, 4.5) | 228.6 (82.1, 433.8) | 3.5 (1.4, 6.3) | 272 (104.3, 482.7) | 0.5 (0.4,0.5) |
| DALYs (Disability-Adjusted Life Years) | Republic of Niue | 0.5 (0.2, 1) | 116.6 (47.9, 204.6) | 0.8 (0.3, 1.4) | 163.2 (70, 277.7) | 1.0 (1.0,1.0) |
| DALYs (Disability-Adjusted Life Years) | Northern Mariana Islands | 6.4 (2.6, 11.3) | 177.9 (73.2, 312.8) | 29.2 (12.6, 47.6) | 232.9 (99.6, 380.6) | 0.9 (0.7,1.1) |
| DALYs (Disability-Adjusted Life Years) | Republic of Palau | 3.4 (1.4, 6.2) | 168.4 (67.7, 302.2) | 9.1 (3.7, 16) | 180.1 (73.2, 320.6) | 0.3 (0.2,0.4) |
| DALYs (Disability-Adjusted Life Years) | Puerto Rico | 1135.3 (486.4, 1855.9) | 145.8 (62.5, 238.4) | 2860.1 (1275.5, 4672.8) | 194.1 (87.1, 316.2) | 0.9 (0.7,1.2) |
| DALYs (Disability-Adjusted Life Years) | Saint Kitts and Nevis | 10.2 (4.1, 17.1) | 126.5 (50.2, 211.2) | 30.6 (13, 50.3) | 193.9 (81.7, 319.2) | 2.0 (1.8,2.1) |
| DALYs (Disability-Adjusted Life Years) | Republic of San Marino | 15.9 (6.3, 27.3) | 203.9 (80.1, 350.1) | 20.2 (7.4, 37.9) | 122.9 (44.8, 231.4) | -0.8 (-1.1,-0.4) |
| DALYs (Disability-Adjusted Life Years) | Tokelau | 0.3 (0.1, 0.6) | 109.2 (44.4, 195.8) | 0.4 (0.2, 0.8) | 134.2 (57, 232.1) | 0.7 (0.7,0.7) |
| DALYs (Disability-Adjusted Life Years) | Tuvalu | 1.7 (0.7, 3) | 108.9 (42.9, 190.7) | 3.4 (1.4, 5.9) | 143.7 (59, 247.2) | 0.9 (0.9,0.9) |
| DALYs (Disability-Adjusted Life Years) | United States Virgin Islands | 39.4 (16.3, 66.5) | 222.1 (91.4, 376.7) | 66.8 (28, 114.2) | 163.3 (68.8, 279.3) | -0.9 (-1.2,-0.7) |
| DALYs (Disability-Adjusted Life Years) | Republic of South Sudan | 93.4 (24.1, 193.5) | 16.8 (4.3, 35) | 258.1 (78.8, 502.2) | 29.3 (8.8, 57.1) | 1.8 (1.6,2.0) |
| DALYs (Disability-Adjusted Life Years) | Republic of Sudan | 937.7 (338.5, 1804.2) | 46.6 (16.8, 89.6) | 3491.1 (1345.6, 6514.5) | 82.3 (31.7, 152.4) | 1.9 (1.8,2.0) |
