# Supplementary material for: Attributable burden of high BMI-related gastrointestinal tract cancers among middle-aged and elderly populations globally, 1990–2021 and projected to 2050: analysis of GBD 2021
Source: Front Nutr. 2026 Jan 2;12:1674621. doi: 10.3389/fnut.2025.1674621 (PMC12807987; doi:10.3389/fnut.2025.1674621)
Supplement: Supplementary file 5 [file Table_4.docx]

****Global and SDI-Based Trends in High BMI-Attributable Pancreatic C**ancer **Burden, 1990–2021****

| **Measure** | **Location** | **Number 1990** | **ASMR 1990** | **Number 2021** | **ASMR 2021** | **EAPC (95% CI)** |
| --- | --- | --- | --- | --- | --- | --- |
| Deaths | Global | 984.1 (-1865.8, 5660.2) | 0.1 (-0.2, 0.7) | 8753.7 (-2193.6, 25213) | 0.5 (-0.1, 1.3) | 4.4 (4.2,4.6) |
| Deaths | Low SDI | -25 (-51.8, 4.2) | -0.1 (-0.1, 0) | -3.6 (-78.9, 112.8) | 0 (-0.1, 0.1) | NA (NA,NA) |
| Deaths | Low-middle SDI | -41 (-104.1, 55.8) | 0 (-0.1, 0) | 406.2 (-122.7, 1249.9) | 0.1 (0, 0.4) | 14.1 (11.3,17.0) |
| Deaths | Middle SDI | -206 (-469.8, 219.4) | -0.1 (-0.2, 0.1) | 1033.9 (-644.1, 3657.1) | 0.2 (-0.1, 0.6) | 18.1 (15.1,21.1) |
| Deaths | High-middle SDI | 419.5 (-571.7, 2046.4) | 0.2 (-0.3, 0.9) | 2916.3 (-629.5, 8481.7) | 0.7 (-0.1, 1.9) | 4.0 (3.9,4.1) |
| Deaths | High SDI | 832 (-796.7, 3489.4) | 0.3 (-0.3, 1.4) | 4385.3 (-793.2, 11922.7) | 1 (-0.1, 2.5) | 3.3 (3.2,3.5) |

****Global and SDI-Based Trends in High BMI-Attributable Pancreatic C**ancer **Burden, 2000–2021****

| **Measure** | **Location** | **Number 2000** | **ASMR 2000** | **Number 2021** | **ASMR 2021** | **EAPC (95% CI)** |
| --- | --- | --- | --- | --- | --- | --- |
| Deaths | Global | 2181 (-1962.8, 8812.7) | 0.2 (-0.2, 0.9) | 8753.7 (-2193.6, 25213) | 0.5 (-0.1, 1.3) | 4.4 (4.2,4.6) |
| Deaths | Low SDI | -25.4 (-57.8, 12.2) | 0 (-0.1, 0) | -3.6 (-78.9, 112.8) | 0 (-0.1, 0.1) | NA (NA,NA) |
| Deaths | Low-middle SDI | -5.5 (-118.5, 188.3) | 0 (-0.1, 0.1) | 406.2 (-122.7, 1249.9) | 0.1 (0, 0.4) | 14.1 (11.3,17.0) |
| Deaths | Middle SDI | -108.9 (-532.3, 602.6) | 0 (-0.2, 0.2) | 1033.9 (-644.1, 3657.1) | 0.2 (-0.1, 0.6) | 18.1 (15.1,21.1) |
| Deaths | High-middle SDI | 762.4 (-615.5, 2964.1) | 0.3 (-0.2, 1.1) | 2916.3 (-629.5, 8481.7) | 0.7 (-0.1, 1.9) | 4.0 (3.9,4.1) |
| Deaths | High SDI | 1551.4 (-787.4, 5220.6) | 0.5 (-0.3, 1.8) | 4385.3 (-793.2, 11922.7) | 1 (-0.1, 2.5) | 3.3 (3.2,3.5) |

****Global and SDI-Based Trends in High BMI-Attributable Pancreatic C**ancer **Burden, 2010–2021****

| **Measure** | **Location** | **Number 2010** | **ASMR 2010** | **Number 2021** | **ASMR 2021** | **EAPC (95% CI)** |
| --- | --- | --- | --- | --- | --- | --- |
| Deaths | Global | 4386.7 (-2245.3, 14440.1) | 0.3 (-0.2, 1.1) | 8753.7 (-2193.6, 25213) | 0.5 (-0.1, 1.3) | 4.4 (4.2,4.6) |
| Deaths | Low SDI | -21.2 (-62.7, 38.4) | 0 (-0.1, 0) | -3.6 (-78.9, 112.8) | 0 (-0.1, 0.1) | NA (NA,NA) |
| Deaths | Low-middle SDI | 128.7 (-123.7, 528.1) | 0.1 (-0.1, 0.2) | 406.2 (-122.7, 1249.9) | 0.1 (0, 0.4) | 14.1 (11.3,17.0) |
| Deaths | Middle SDI | 187.4 (-589.6, 1472.2) | 0 (-0.2, 0.4) | 1033.9 (-644.1, 3657.1) | 0.2 (-0.1, 0.6) | 18.1 (15.1,21.1) |
| Deaths | High-middle SDI | 1447.9 (-704.2, 4797.5) | 0.4 (-0.2, 1.5) | 2916.3 (-629.5, 8481.7) | 0.7 (-0.1, 1.9) | 4.0 (3.9,4.1) |
| Deaths | High SDI | 2633 (-833, 7852) | 0.7 (-0.2, 2.2) | 4385.3 (-793.2, 11922.7) | 1 (-0.1, 2.5) | 3.3 (3.2,3.5) |

| **Measure** | **Location** | **Number 1990** | **ASR 1990** | **Number 2021** | **ASR 2021** | **EAPC (95% CI)** |
| --- | --- | --- | --- | --- | --- | --- |
| DALYs (Disability-Adjusted Life Years) | Global | 23757.2 (-41099.6, 130903.3) | 2.7 (-4.8, 15.1) | 201967.6 (-40488, 565231.7) | 10.5 (-2.2, 29.4) | 4.4 (4.2,4.6) |
| DALYs (Disability-Adjusted Life Years) | Low SDI | -607.9 (-1276.7, 137) | -1.3 (-2.6, 0.2) | 107.1 (-1795.7, 3121.6) | 0 (-1.7, 2.7) | NA (NA,NA) |
| DALYs (Disability-Adjusted Life Years) | Low-middle SDI | -939.7 (-2511.9, 1522.6) | -0.8 (-1.9, 1.1) | 10709.9 (-2591.7, 31996) | 3.3 (-0.9, 9.9) | 13.7 (10.9,16.6) |
| DALYs (Disability-Adjusted Life Years) | Middle SDI | -4947.9 (-11519.5, 5778.7) | -2.2 (-5.1, 2.5) | 26895.1 (-13978.9, 91015.1) | 4.3 (-2.4, 14.7) | 17.2 (14.0,20.5) |
| DALYs (Disability-Adjusted Life Years) | High-middle SDI | 10510.6 (-13246.5, 49770.2) | 4.6 (-6, 22) | 67548.3 (-12474.8, 192890.1) | 15.1 (-2.8, 43.1) | 3.8 (3.7,3.9) |
| DALYs (Disability-Adjusted Life Years) | High SDI | 19626.1 (-15777.9, 77788.7) | 8.2 (-6.5, 32.4) | 96356.7 (-11436.8, 251374.1) | 22.1 (-2.2, 57.1) | 3.3 (3.1,3.5) |

****Global and SDI-Based Trends in High BMI-Attributable Pancreatic C**ancer **Burden, 1990–2021****

| **Measure** | **Location** | **Number 1990** | **ASR 1990** | **Number 2021** | **ASR 2021** | **EAPC (95% CI)** |
| --- | --- | --- | --- | --- | --- | --- |
| YLDs (Years Lived with Disability) | Global | 205 (-358.2, 1147.5) | 0 (0, 0.1) | 1802 (-421.5, 5213.2) | 0.1 (0, 0.3) | 4.5 (4.3,4.7) |
| YLDs (Years Lived with Disability) | Low SDI | -5 (-11.2, 0.8) | 0 (0, 0) | -0.1 (-15.5, 24.3) | 0 (0, 0) | NA (NA,NA) |
| YLDs (Years Lived with Disability) | Low-middle SDI | -7.9 (-22, 11.6) | 0 (0, 0) | 85.2 (-22.6, 257.3) | 0 (0, 0.1) | 16.1 (11.9,20.5) |
| YLDs (Years Lived with Disability) | Middle SDI | -40.9 (-99.5, 46) | 0 (0, 0) | 214.1 (-131.5, 774.2) | 0 (0, 0.1) | 26.9 (16.3,38.6) |
| YLDs (Years Lived with Disability) | High-middle SDI | 85.8 (-106.3, 407.7) | 0 (0, 0.2) | 577 (-114.3, 1659.5) | 0.1 (0, 0.4) | 3.9 (3.8,4.0) |
| YLDs (Years Lived with Disability) | High SDI | 172.1 (-148.4, 721.4) | 0.1 (-0.1, 0.3) | 922.8 (-160.2, 2539.3) | 0.2 (0, 0.6) | 3.5 (3.3,3.7) |

****Global and SDI-Based Trends in High BMI-Attributable Pancreatic C**ancer **Burden, 1990–2021****

****Global and SDI-Based Trends in High BMI-Attributable Pancreatic C**ancer **Burden, 1990–2021****

| **Measure** | **Location** | **Number 1990** | **ASR 1990** | **Number 2021** | **ASR 2021** | **EAPC (95% CI)** |
| --- | --- | --- | --- | --- | --- | --- |
| YLLs (Years of Life Lost) | Global | 23552.2 (-40744.2, 129769.4) | 2.7 (-4.8, 15) | 200165.7 (-40037.8, 560548.3) | 10.4 (-2.1, 29.2) | 4.4 (4.2,4.6) |
| YLLs (Years of Life Lost) | Low SDI | -603 (-1266.1, 136.2) | -1.3 (-2.6, 0.2) | 107.2 (-1780.9, 3098.9) | 0 (-1.7, 2.6) | NA (NA,NA) |
| YLLs (Years of Life Lost) | Low-middle SDI | -931.8 (-2490.7, 1511.1) | -0.7 (-1.9, 1.1) | 10624.8 (-2568.4, 31727.1) | 3.2 (-0.9, 9.8) | 13.7 (10.9,16.5) |
| YLLs (Years of Life Lost) | Middle SDI | -4907.1 (-11432.8, 5732.9) | -2.2 (-5.1, 2.4) | 26681 (-13846, 90324.3) | 4.2 (-2.3, 14.5) | 17.2 (14.0,20.5) |
| YLLs (Years of Life Lost) | High-middle SDI | 10424.8 (-13139.4, 49397) | 4.5 (-6, 21.8) | 66971.3 (-12352.6, 191361.9) | 15 (-2.8, 42.8) | 3.8 (3.7,3.9) |
| YLLs (Years of Life Lost) | High SDI | 19454 (-15636.2, 77080.9) | 8.1 (-6.4, 32.1) | 95433.9 (-11275.8, 248974.8) | 21.9 (-2.2, 56.5) | 3.3 (3.1,3.4) |

****Burden of Pancreatic C**ancer **Deaths Attributable to High BMI in 21 GBD Regions, 1990–2021****

| **Measure** | **Location** | **Number 1990** | **ASMR 1990** | **Number 2021** | **ASMR 2021** | **EAPC (95% CI)** |
| --- | --- | --- | --- | --- | --- | --- |
| Deaths | Global | 984.1 (-1865.8, 5660.2) | 0.1 (-0.2, 0.7) | 8753.7 (-2193.6, 25213) | 0.5 (-0.1, 1.3) | 4.4 (4.2,4.6) |
| Deaths | Andean Latin America | 1 (-13, 25.4) | 0 (-0.3, 0.6) | 74.2 (-12, 208.1) | 0.6 (-0.1, 1.6) | 12.3 (8.9,15.8) |
| Deaths | Australasia | 21.5 (-14.2, 80.6) | 0.4 (-0.3, 1.6) | 153.7 (-7.6, 378.8) | 1.3 (-0.1, 3.1) | 3.7 (3.5,3.9) |
| Deaths | Caribbean | 1.3 (-14.1, 26.4) | 0 (-0.3, 0.5) | 59.4 (-10.6, 168) | 0.5 (-0.1, 1.4) | 8.6 (7.2,10.0) |
| Deaths | Central Asia | 24.7 (-6.5, 74.4) | 0.2 (-0.1, 0.7) | 100.1 (-6.8, 260.4) | 0.6 (0, 1.5) | 2.8 (2.6,3.1) |
| Deaths | Central Europe | 270.5 (-50.9, 780.2) | 0.8 (-0.2, 2.3) | 756.3 (-33.2, 1870.5) | 1.5 (-0.1, 3.7) | 2.1 (2.0,2.2) |
| Deaths | Central Latin America | 38.1 (-28.1, 151.5) | 0.2 (-0.2, 0.9) | 398.7 (-19.5, 997.8) | 0.7 (0, 1.8) | 3.8 (3.5,4.1) |
| Deaths | Central Sub-Saharan Africa | -4.1 (-9.5, 1.5) | -0.1 (-0.2, 0) | 7.4 (-9.9, 36.3) | 0.1 (-0.1, 0.3) | 43.2 (31.0,56.4) |
| Deaths | East Asia | -584.2 (-1042.4, -47.9) | -0.3 (-0.6, 0) | 119.7 (-1452.1, 2738.9) | 0 (-0.3, 0.5) | NA (NA,NA) |
| Deaths | Eastern Europe | 364.3 (-84.5, 1050.3) | 0.6 (-0.1, 1.7) | 1121.4 (-27.4, 2733.8) | 1.4 (0, 3.5) | 2.9 (2.7,3.0) |
| Deaths | Eastern Sub-Saharan Africa | -12.2 (-25.2, 1.1) | -0.1 (-0.2, 0) | 1.6 (-34.8, 57.2) | 0 (-0.1, 0.2) | NA (NA,NA) |
| Deaths | High-income Asia Pacific | -267.6 (-489.9, -7.2) | -0.6 (-1.1, 0) | -475 (-1148.7, 313.1) | -0.4 (-0.9, 0.3) | NA (NA,NA) |
| Deaths | High-income North America | 624.7 (-164.1, 1874.8) | 0.8 (-0.2, 2.4) | 2766 (-11.8, 6363.7) | 1.9 (0, 4.3) | 2.6 (2.4,2.9) |
| Deaths | North Africa and Middle East | 61.1 (-28.6, 205) | 0.2 (-0.1, 0.6) | 871.2 (10.1, 2008.7) | 0.9 (0, 2.2) | 5.9 (5.8,6.0) |
| Deaths | Oceania | 0.2 (-0.6, 1.5) | 0 (-0.1, 0.2) | 2.7 (-0.9, 8.3) | 0.2 (-0.1, 0.5) | 8.0 (7.0,9.1) |
| Deaths | South Asia | -49.9 (-94.9, -2) | 0 (-0.1, 0) | -26.7 (-205.3, 258.1) | 0 (-0.1, 0.1) | NA (NA,NA) |
| Deaths | Southeast Asia | -57.3 (-114.9, -0.4) | -0.1 (-0.2, 0) | -39 (-259.5, 291) | 0 (-0.2, 0.2) | NA (NA,NA) |
| Deaths | Southern Latin America | 69.2 (-29.3, 229.2) | 0.7 (-0.3, 2.3) | 294.2 (-14.9, 731) | 1.5 (-0.1, 3.8) | 2.9 (2.7,3.1) |
| Deaths | Southern Sub-Saharan Africa | 14 (-4.4, 41.5) | 0.2 (-0.1, 0.7) | 121.3 (-0.2, 285.1) | 1 (0, 2.4) | 4.8 (4.4,5.2) |
| Deaths | Tropical Latin America | 23.7 (-44.5, 139.3) | 0.1 (-0.3, 0.7) | 419.6 (-47.2, 1090.8) | 0.7 (-0.1, 1.9) | 6.3 (5.7,6.8) |
| Deaths | Western Europe | 449.8 (-431.7, 1859) | 0.3 (-0.3, 1.4) | 1988.5 (-414.3, 5719) | 0.9 (-0.2, 2.7) | 3.3 (3.1,3.5) |
| Deaths | Western Sub-Saharan Africa | -4.5 (-12.8, 7.6) | 0 (-0.1, 0) | 38.4 (-20, 137.5) | 0.1 (-0.1, 0.3) | 24.0 (18.8,29.5) |

****Burden of Pancreatic Cancer Deaths Attributable to High BMI in Country 1990–2021****

| **Measure** | **Location** | **Number 1990** | **ASMR 1990** | **Number 2021** | **ASMR 2021** | **EAPC (95% CI)** |
| --- | --- | --- | --- | --- | --- | --- |
| Deaths | China | -569.6 (-1014, -47.8) | -0.3 (-0.6, 0) | 112.1 (-1416.1, 2645.3) | 0 (-0.3, 0.5) | NA (NA,NA) |
| Deaths | Democratic People's Republic of Korea | -8.7 (-16.7, -1.2) | -0.3 (-0.5, 0) | -10.8 (-29.8, 11.8) | -0.1 (-0.4, 0.2) | NA (NA,NA) |
| Deaths | Taiwan (Province of China) | -5.9 (-14.3, 4.2) | -0.2 (-0.4, 0.1) | 18.4 (-31.1, 103.5) | 0.2 (-0.3, 1.1) | 33.9 (20.7,48.6) |
| Deaths | Kingdom of Cambodia | -1.1 (-2.3, -0.1) | -0.1 (-0.3, 0) | -2.9 (-7.4, 1.5) | -0.1 (-0.3, 0) | NA (NA,NA) |
| Deaths | Republic of Indonesia | -25.5 (-48.5, -3.3) | -0.1 (-0.2, 0) | -41.5 (-146.3, 87.6) | -0.1 (-0.3, 0.1) | NA (NA,NA) |
| Deaths | Lao People's Democratic Republic | -0.5 (-1.2, 0) | -0.1 (-0.3, 0) | -0.4 (-1.9, 1.7) | -0.1 (-0.2, 0.2) | NA (NA,NA) |
| Deaths | Malaysia | -0.8 (-2.9, 1.8) | 0 (-0.2, 0.1) | 5.8 (-5.6, 25) | 0.1 (-0.1, 0.4) | 17.2 (12.0,22.8) |
| Deaths | Republic of Maldives | 0 (0, 0) | -0.1 (-0.2, 0) | 0 (-0.1, 0.1) | 0 (-0.2, 0.2) | NA (NA,NA) |
| Deaths | Republic of the Union of Myanmar | -5.6 (-12.8, 0.2) | -0.1 (-0.3, 0) | -8.4 (-26.2, 12.4) | -0.1 (-0.3, 0.1) | NA (NA,NA) |
| Deaths | Republic of the Philippines | -4.1 (-10.3, 2.8) | -0.1 (-0.2, 0) | 8.2 (-25.1, 61.5) | 0 (-0.2, 0.3) | 31.7 (22.4,41.7) |
| Deaths | Democratic Socialist Republic of Sri Lanka | -0.8 (-2.2, 0.8) | 0 (-0.1, 0) | 1.7 (-3.9, 10.5) | 0 (-0.1, 0.2) | 39.7 (28.3,52.2) |
| Deaths | Kingdom of Thailand | -12.8 (-27.8, 1.9) | -0.2 (-0.4, 0) | 15.1 (-58, 122.5) | 0.1 (-0.2, 0.5) | 59.4 (38.6,83.3) |
| Deaths | Democratic Republic of Timor-Leste | 0 (-0.1, 0) | -0.1 (-0.2, 0) | -0.2 (-0.4, 0) | -0.1 (-0.2, 0) | NA (NA,NA) |
| Deaths | Socialist Republic of Viet Nam | -5.9 (-11.4, -1.3) | -0.1 (-0.1, 0) | -17.4 (-40.6, 3.6) | -0.1 (-0.2, 0) | NA (NA,NA) |
| Deaths | Republic of Fiji | 0.1 (0, 0.5) | 0.2 (-0.1, 0.6) | 1.1 (0, 2.6) | 0.7 (0, 1.6) | 4.3 (4.2,4.5) |
| Deaths | Republic of Kiribati | 0 (0, 0) | 0 (-0.1, 0.2) | 0 (0, 0.1) | 0.2 (0, 0.5) | 5.4 (5.1,5.8) |
| Deaths | Republic of the Marshall Islands | 0 (0, 0) | 0.2 (-0.1, 0.7) | 0 (0, 0.1) | 0.6 (0, 1.7) | 4.1 (3.9,4.2) |
| Deaths | Federated States of Micronesia | 0 (0, 0.1) | 0.2 (-0.1, 0.8) | 0.1 (0, 0.3) | 0.8 (0, 2) | 4.0 (3.8,4.2) |
| Deaths | Independent State of Papua New Guinea | -0.3 (-0.6, 0.2) | -0.1 (-0.2, 0) | -0.2 (-1.6, 1.9) | -0.1 (-0.2, 0.1) | NA (NA,NA) |
| Deaths | Independent State of Samoa | 0.1 (0, 0.2) | 0.4 (-0.1, 1.2) | 0.3 (0, 0.7) | 0.8 (0, 2.1) | 2.3 (2.3,2.4) |
| Deaths | Solomon Islands | 0 (0, 0) | -0.1 (-0.2, 0.1) | 0.1 (-0.1, 0.4) | 0.1 (-0.2, 0.5) | 29.0 (19.6,39.1) |
| Deaths | Kingdom of Tonga | 0.1 (0, 0.2) | 0.5 (-0.1, 1.5) | 0.3 (0, 0.6) | 1.5 (0.1, 3.4) | 3.1 (2.8,3.3) |
| Deaths | Republic of Vanuatu | 0 (0, 0) | -0.1 (-0.2, 0.1) | 0 (0, 0.2) | 0.1 (-0.2, 0.5) | 22.9 (17.4,28.6) |
| Deaths | Republic of Armenia | 4.1 (-0.8, 11.8) | 0.7 (-0.1, 2) | 13.4 (-1, 34.3) | 1.4 (-0.1, 3.5) | 2.3 (2.1,2.5) |
| Deaths | Republic of Azerbaijan | 2.1 (-0.9, 7.2) | 0.2 (-0.1, 0.6) | 14.2 (-0.9, 41.1) | 0.6 (0, 1.8) | 4.5 (4.1,5.0) |
| Deaths | Georgia | 2.2 (-0.8, 7.1) | 0.2 (-0.1, 0.5) | 9.5 (-1.5, 27.5) | 0.7 (-0.1, 2.1) | 6.3 (5.9,6.7) |
| Deaths | Republic of Kazakhstan | 11 (-2.8, 34.4) | 0.4 (-0.1, 1.3) | 30.5 (-1.1, 78.5) | 0.8 (0, 2) | 1.7 (1.3,2.0) |
| Deaths | Kyrgyz Republic | 2 (-0.6, 6.3) | 0.3 (-0.1, 0.9) | 8 (-0.4, 20.3) | 0.8 (0, 2) | 4.1 (3.6,4.6) |
| Deaths | Mongolia | 0.1 (-0.1, 0.6) | 0 (-0.1, 0.2) | 2.3 (-1.1, 8.1) | 0.5 (-0.3, 1.7) | 8.7 (7.9,9.4) |
| Deaths | Republic of Tajikistan | 0.6 (-0.3, 2.3) | 0.1 (-0.1, 0.4) | 2 (-0.3, 6) | 0.2 (0, 0.5) | 1.0 (0.7,1.2) |
| Deaths | Turkmenistan | 0 (0, 0.2) | 0 (0, 0) | 2.4 (-0.5, 7.2) | 0.3 (-0.1, 0.8) | 13.0 (11.3,14.7) |
| Deaths | Republic of Uzbekistan | 2.5 (-1.2, 8.9) | 0.1 (-0.1, 0.4) | 17.7 (-1.6, 49.8) | 0.3 (0, 0.9) | 3.6 (3.4,3.8) |
| Deaths | Republic of Albania | 1 (-0.7, 4.1) | 0.2 (-0.2, 1) | 6.3 (-1.1, 19.2) | 0.6 (-0.1, 1.9) | 3.4 (3.2,3.6) |
| Deaths | Bosnia and Herzegovina | 3.7 (-2.3, 13.2) | 0.4 (-0.3, 1.5) | 15.9 (-1.4, 41.6) | 1.1 (-0.1, 2.9) | 3.3 (3.1,3.5) |
| Deaths | Republic of Bulgaria | 20 (-3.1, 56.7) | 0.7 (-0.1, 2.1) | 50.1 (-2.4, 130.8) | 1.6 (-0.1, 4.1) | 3.0 (2.8,3.2) |
| Deaths | Republic of Croatia | 9.4 (-3, 30.1) | 0.7 (-0.2, 2.3) | 30.6 (-1.5, 76.3) | 1.5 (-0.1, 3.7) | 2.9 (2.7,3.1) |
| Deaths | Czech Republic | 47.2 (-5.8, 126.4) | 1.5 (-0.2, 4.1) | 98.9 (-3.8, 253.1) | 2 (-0.1, 5.2) | 1.0 (1.0,1.1) |
| Deaths | Hungary | 41.9 (-3.3, 109.4) | 1.3 (-0.1, 3.3) | 92.7 (-1.2, 216.8) | 2.2 (0, 5) | 1.8 (1.8,1.9) |
| Deaths | North Macedonia | 2.4 (-0.7, 7.4) | 0.6 (-0.2, 1.8) | 9.7 (-0.7, 25.3) | 1.3 (-0.1, 3.5) | 2.9 (2.6,3.2) |
| Deaths | Montenegro | 1.4 (-0.1, 3.7) | 1 (-0.1, 2.7) | 4.7 (0, 11.3) | 2.2 (0, 5.2) | 2.9 (2.8,3.0) |
| Deaths | Republic of Poland | 69.6 (-23.1, 226.6) | 0.7 (-0.2, 2.3) | 203.8 (-17.2, 534.7) | 1.2 (-0.1, 3.3) | 1.6 (1.4,1.8) |
| Deaths | Romania | 34.9 (-7.2, 99.7) | 0.5 (-0.1, 1.6) | 119.8 (-5.3, 303.2) | 1.5 (-0.1, 3.7) | 3.0 (2.8,3.2) |
| Deaths | Republic of Serbia | 12 (-7.1, 45.6) | 0.5 (-0.3, 1.9) | 58.3 (-1.8, 145.2) | 1.6 (0, 3.9) | 3.8 (3.6,4.0) |
| Deaths | Slovak Republic | 17.5 (-1.2, 46.5) | 1.3 (-0.1, 3.5) | 39.5 (-0.6, 96.4) | 1.8 (0, 4.5) | 1.3 (1.2,1.4) |
| Deaths | Republic of Slovenia | 5.4 (-0.6, 14.7) | 1 (-0.1, 2.7) | 14.9 (-0.6, 36.7) | 1.5 (0, 3.6) | 1.5 (1.3,1.6) |
| Deaths | Republic of Belarus | 10.3 (-5.4, 36.1) | 0.3 (-0.2, 1.2) | 41.3 (-1.9, 105.9) | 1.1 (-0.1, 2.9) | 3.6 (3.5,3.8) |
| Deaths | Republic of Estonia | 4.1 (-0.6, 11.4) | 0.9 (-0.1, 2.5) | 9.9 (-0.4, 25.4) | 1.6 (-0.1, 4.2) | 1.9 (1.7,2.1) |
| Deaths | Republic of Latvia | 7.2 (-1.2, 20.6) | 0.9 (-0.1, 2.6) | 15.9 (-0.7, 39.7) | 1.8 (-0.1, 4.4) | 2.1 (1.9,2.3) |
| Deaths | Republic of Lithuania | 6.5 (-2.4, 20.7) | 0.6 (-0.2, 2.1) | 21.7 (-0.7, 55.3) | 1.7 (-0.1, 4.3) | 3.0 (2.9,3.2) |
| Deaths | Republic of Moldova | 7 (-1.2, 20.1) | 0.7 (-0.1, 2.1) | 20.2 (-0.2, 48.2) | 1.5 (0, 3.6) | 2.9 (2.5,3.3) |
| Deaths | Russian Federation | 250.1 (-63.5, 745.2) | 0.6 (-0.2, 1.8) | 829.9 (-21.1, 2021.2) | 1.5 (0, 3.8) | 2.9 (2.7,3.1) |
| Deaths | Ukraine | 79.1 (-11.3, 221) | 0.5 (-0.1, 1.4) | 182.5 (-4.2, 462.5) | 1.1 (0, 2.7) | 2.6 (2.5,2.7) |
| Deaths | Brunei Darussalam | -0.1 (-0.1, 0) | -0.3 (-0.7, 0.1) | 0.2 (-0.2, 0.8) | 0.1 (-0.4, 0.9) | 42.4 (32.2,53.4) |
| Deaths | Japan | -225.6 (-418.2, -2.2) | -0.6 (-1.1, 0) | -433.9 (-1032.3, 235.6) | -0.4 (-1.1, 0.3) | NA (NA,NA) |
| Deaths | Republic of Korea | -40.3 (-70.9, -3.2) | -0.7 (-1.2, -0.1) | -43.2 (-146.6, 93.4) | -0.2 (-0.7, 0.4) | NA (NA,NA) |
| Deaths | Republic of Singapore | -1.7 (-3.1, 0) | -0.4 (-0.7, 0) | 1.9 (-5.5, 13) | 0.1 (-0.3, 0.7) | 89.6 (34.2,167.8) |
| Deaths | Australia | 17.4 (-12.6, 67.2) | 0.4 (-0.3, 1.6) | 134 (-5.8, 329.7) | 1.3 (0, 3.2) | 3.9 (3.7,4.1) |
| Deaths | New Zealand | 4.1 (-2.1, 14.1) | 0.5 (-0.2, 1.6) | 19.7 (-1.9, 51) | 1 (-0.1, 2.7) | 2.8 (2.6,2.9) |
| Deaths | Principality of Andorra | 0 (-0.1, 0.3) | 0.3 (-0.8, 2.2) | 0.2 (-0.1, 0.9) | 0.7 (-0.4, 2.6) | 3.7 (3.4,3.9) |
| Deaths | Republic of Austria | 10.4 (-11, 46.2) | 0.4 (-0.4, 1.7) | 34.6 (-11.9, 112.1) | 0.8 (-0.3, 2.7) | 2.6 (2.5,2.7) |
| Deaths | Kingdom of Belgium | 5.3 (-17.2, 40.8) | 0.2 (-0.5, 1.2) | 40.5 (-13.6, 126.7) | 0.8 (-0.2, 2.4) | 5.0 (4.6,5.4) |
| Deaths | Republic of Cyprus | 0 (-0.8, 1) | -0.1 (-0.5, 0.6) | 2.5 (-1, 8.7) | 0.6 (-0.2, 2) | 11.6 (9.5,13.9) |
| Deaths | Kingdom of Denmark | 2.6 (-6.1, 17) | 0.2 (-0.3, 1) | 18.7 (-9.3, 64.5) | 0.7 (-0.3, 2.4) | 4.7 (4.2,5.2) |
| Deaths | Republic of Finland | 7.7 (-6.6, 31.8) | 0.5 (-0.4, 2) | 34.4 (-7.4, 99) | 1.2 (-0.2, 3.4) | 2.9 (2.8,3.0) |
| Deaths | French Republic | 10.3 (-80.6, 155.4) | 0.1 (-0.4, 0.9) | 259.7 (-76.7, 776.9) | 0.8 (-0.2, 2.4) | 7.9 (7.0,8.9) |
| Deaths | Federal Republic of Germany | 173.9 (-83.2, 583.1) | 0.6 (-0.3, 2) | 491.7 (-101.4, 1456.2) | 1.1 (-0.2, 3.3) | 2.0 (1.9,2.0) |
| Deaths | Hellenic Republic | 8.7 (-16.5, 50.8) | 0.3 (-0.5, 1.5) | 62.9 (-12.8, 181.9) | 1.2 (-0.2, 3.4) | 4.9 (4.5,5.2) |
| Deaths | Republic of Iceland | 0.4 (-0.2, 1.4) | 0.6 (-0.3, 2.2) | 1.4 (-0.2, 4) | 1.1 (-0.2, 3.1) | 2.1 (1.9,2.2) |
| Deaths | Ireland | 2.7 (-4.5, 14.7) | 0.3 (-0.5, 1.6) | 14.5 (-2.7, 41.4) | 0.8 (-0.1, 2.3) | 3.3 (2.9,3.7) |
| Deaths | State of Israel | 6.1 (-3.4, 21.9) | 0.6 (-0.3, 2) | 26.7 (-5.1, 77.3) | 1 (-0.2, 2.8) | 1.9 (1.7,2.1) |
| Deaths | Republic of Italy | 29.4 (-91.5, 240.1) | 0.1 (-0.5, 1.2) | 250.7 (-90, 791.4) | 0.7 (-0.3, 2.3) | 4.8 (4.3,5.3) |
| Deaths | Grand Duchy of Luxembourg | 0.6 (-0.4, 2.2) | 0.5 (-0.3, 1.8) | 2.4 (-0.3, 6.6) | 1 (-0.1, 2.8) | 2.6 (2.3,2.9) |
| Deaths | Republic of Malta | 0 (-0.5, 0.9) | 0 (-0.6, 1) | 1.8 (-0.6, 5.5) | 0.8 (-0.2, 2.5) | 9.2 (7.3,11.0) |
| Deaths | Kingdom of the Netherlands | 6.7 (-21.2, 52.4) | 0.2 (-0.5, 1.2) | 48.8 (-24.5, 165.9) | 0.6 (-0.3, 2.1) | 4.8 (4.4,5.2) |
| Deaths | Kingdom of Norway | 2.6 (-6.6, 17.9) | 0.2 (-0.4, 1.2) | 10.9 (-7.8, 40.6) | 0.5 (-0.3, 1.8) | 2.9 (2.7,3.0) |
| Deaths | Portuguese Republic | 1.4 (-14, 28.5) | 0 (-0.5, 0.9) | 36 (-12.1, 115.4) | 0.7 (-0.2, 2.1) | 7.6 (6.6,8.6) |
| Deaths | Kingdom of Spain | 50.2 (-29.8, 185.2) | 0.4 (-0.2, 1.5) | 242.5 (-19.1, 639.1) | 1.1 (-0.1, 2.9) | 3.3 (3.1,3.5) |
| Deaths | Kingdom of Sweden | 9.8 (-17.1, 54) | 0.3 (-0.5, 1.6) | 35.2 (-14.4, 117.6) | 0.7 (-0.3, 2.4) | 2.5 (2.3,2.8) |
| Deaths | Swiss Confederation | 4.2 (-6.7, 22) | 0.2 (-0.3, 0.9) | 19.6 (-12.1, 68.5) | 0.5 (-0.3, 1.6) | 3.3 (3.1,3.5) |
| Deaths | United Kingdom of Great Britain and Northern Ireland | 116.4 (-58.4, 415) | 0.6 (-0.3, 2.1) | 350.4 (-35.8, 943.1) | 1.2 (-0.1, 3.1) | 2.5 (2.4,2.6) |
| Deaths | Argentine Republic | 52.8 (-23.9, 178.1) | 0.7 (-0.4, 2.5) | 203.1 (-11.2, 511.3) | 1.6 (-0.1, 4.1) | 2.9 (2.7,3.1) |
| Deaths | Republic of Chile | 10.8 (-3.9, 35.5) | 0.5 (-0.2, 1.7) | 69.5 (-2.5, 170.6) | 1.2 (0, 3) | 3.1 (2.8,3.4) |
| Deaths | Eastern Republic of Uruguay | 5.5 (-3.3, 20.1) | 0.6 (-0.4, 2.3) | 21.5 (-2.7, 57.9) | 1.8 (-0.2, 4.7) | 3.4 (3.2,3.5) |
| Deaths | Canada | 45.4 (-19.8, 152.1) | 0.6 (-0.3, 2.1) | 183.7 (-16.5, 475.5) | 1.1 (-0.1, 2.9) | 1.8 (1.7,2.0) |
| Deaths | United States of America | 579.1 (-147.8, 1730.3) | 0.8 (-0.2, 2.5) | 2581.9 (-1.7, 5888.2) | 2 (0, 4.4) | 2.7 (2.5,2.9) |
| Deaths | Antigua and Barbuda | 0 (0, 0.1) | 0 (-0.3, 0.5) | 0.1 (0, 0.4) | 0.6 (-0.1, 1.7) | 10.6 (9.0,12.2) |
| Deaths | Commonwealth of the Bahamas | 0.1 (-0.1, 0.2) | 0.2 (-0.2, 0.7) | 0.6 (0, 1.5) | 0.6 (-0.1, 1.7) | 4.6 (4.3,4.9) |
| Deaths | Barbados | 0.1 (-0.3, 0.8) | 0.2 (-0.4, 1.2) | 1.3 (-0.1, 3.5) | 1.1 (-0.1, 3) | 5.0 (4.7,5.4) |
| Deaths | Belize | 0.1 (0, 0.2) | 0.3 (-0.1, 1) | 0.6 (0, 1.5) | 1 (0, 2.4) | 3.7 (2.9,4.4) |
| Deaths | Republic of Cuba | -0.8 (-9.3, 11.4) | 0 (-0.4, 0.5) | 20.8 (-5, 63.1) | 0.5 (-0.1, 1.4) | 9.5 (8.1,11.0) |
| Deaths | Commonwealth of Dominica | 0.1 (0, 0.2) | 0.6 (-0.1, 1.8) | 0.3 (0, 0.8) | 1.7 (0, 4.1) | 3.4 (3.2,3.5) |
| Deaths | Dominican Republic | -0.8 (-2.5, 1.7) | -0.1 (-0.4, 0.2) | 7.2 (-5.2, 27.7) | 0.3 (-0.3, 1.3) | 20.6 (15.7,25.6) |
| Deaths | Grenada | 0 (-0.1, 0.1) | 0 (-0.5, 0.7) | 0.2 (-0.1, 0.6) | 0.7 (-0.3, 2.3) | 15.9 (14.2,17.7) |
| Deaths | Republic of Guyana | 0 (-0.2, 0.3) | 0 (-0.3, 0.4) | 0.6 (-0.2, 1.8) | 0.4 (-0.2, 1.3) | 11.0 (9.4,12.7) |
| Deaths | Republic of Haiti | -1.3 (-2.7, 0) | -0.2 (-0.4, 0) | -0.9 (-4.1, 3.2) | -0.1 (-0.3, 0.2) | NA (NA,NA) |
| Deaths | Jamaica | 0.2 (-0.9, 2) | 0.1 (-0.2, 0.5) | 3.9 (-0.4, 10.1) | 0.6 (-0.1, 1.5) | 6.5 (6.0,7.1) |
| Deaths | Saint Lucia | 0 (-0.1, 0.1) | -0.1 (-0.6, 0.5) | 0.2 (-0.1, 0.8) | 0.4 (-0.2, 1.5) | 14.3 (11.5,17.2) |
| Deaths | Saint Vincent and the Grenadines | 0 (-0.1, 0) | -0.2 (-0.6, 0.3) | 0.1 (-0.1, 0.3) | 0.2 (-0.3, 1) | 27.3 (18.2,37.2) |
| Deaths | Republic of Suriname | -0.1 (-0.3, 0.1) | -0.2 (-0.5, 0.2) | 0.3 (-0.4, 1.5) | 0.2 (-0.3, 1) | 35.7 (23.6,49.1) |
| Deaths | Republic of Trinidad and Tobago | 0.4 (-0.4, 1.7) | 0.2 (-0.2, 0.9) | 2.9 (-0.4, 7.9) | 0.7 (-0.1, 1.8) | 3.3 (3.1,3.5) |
| Deaths | Plurinational State of Bolivia | -0.4 (-3.3, 3.8) | -0.1 (-0.6, 0.5) | 11.5 (-3.9, 37.5) | 0.6 (-0.2, 1.9) | 13.9 (10.4,17.5) |
| Deaths | Republic of Ecuador | 0.7 (-2.8, 6.3) | 0 (-0.3, 0.6) | 26.7 (-1.4, 68.4) | 0.8 (0, 1.9) | 8.5 (7.5,9.6) |
| Deaths | Republic of Peru | 0.6 (-8.6, 16.2) | 0 (-0.4, 0.6) | 36 (-8.8, 109.1) | 0.5 (-0.1, 1.5) | 9.7 (7.9,11.6) |
| Deaths | Republic of Colombia | 3.3 (-10.2, 25.5) | 0.1 (-0.3, 0.7) | 64 (-8.5, 176.8) | 0.5 (-0.1, 1.5) | 5.5 (5.1,5.8) |
| Deaths | Republic of Costa Rica | 0.6 (-0.7, 2.7) | 0.1 (-0.2, 0.7) | 8.9 (-1, 24) | 0.7 (-0.1, 2) | 4.8 (4.3,5.4) |
| Deaths | Republic of El Salvador | 1 (-0.6, 3.5) | 0.2 (-0.1, 0.6) | 10.2 (-0.4, 26.1) | 0.8 (0, 2) | 5.1 (4.7,5.5) |
| Deaths | Republic of Guatemala | 0.6 (-0.4, 2.3) | 0.1 (-0.1, 0.3) | 10.1 (-0.9, 26.2) | 0.4 (0, 1.1) | 6.1 (5.3,7.0) |
| Deaths | Republic of Honduras | 0.3 (-0.6, 1.9) | 0.1 (-0.2, 0.4) | 7.6 (-1.3, 22.9) | 0.6 (-0.1, 1.7) | 7.1 (6.6,7.5) |
| Deaths | United Mexican States | 29.3 (-17.1, 108.4) | 0.3 (-0.2, 1.2) | 230.7 (-8.1, 575.4) | 0.8 (0, 2.1) | 2.9 (2.7,3.1) |
| Deaths | Republic of Nicaragua | 0.4 (-0.2, 1.4) | 0.1 (-0.1, 0.4) | 5.3 (-0.2, 13.5) | 0.5 (0, 1.3) | 4.7 (4.2,5.1) |
| Deaths | Republic of Panama | 0.7 (-0.1, 2) | 0.2 (0, 0.6) | 7.7 (0, 18.1) | 0.8 (0, 1.9) | 4.1 (3.5,4.6) |
| Deaths | Bolivarian Republic of Venezuela | 1.9 (-0.9, 6.6) | 0.1 (-0.1, 0.3) | 54.3 (-1.7, 139.3) | 0.8 (0, 2.1) | 6.4 (5.1,7.7) |
| Deaths | Federative Republic of Brazil | 23.4 (-44, 137.5) | 0.1 (-0.3, 0.7) | 411.3 (-46.1, 1069.6) | 0.7 (-0.1, 2) | 6.2 (5.7,6.8) |
| Deaths | Republic of Paraguay | 0.3 (-0.6, 1.8) | 0.1 (-0.1, 0.4) | 8.3 (-1.3, 24) | 0.7 (-0.1, 1.9) | 8.1 (7.4,8.9) |
| Deaths | People's Democratic Republic of Algeria | -0.2 (-2.2, 2.9) | 0 (-0.1, 0.1) | 15.5 (-1.5, 42.1) | 0.2 (0, 0.6) | 15.1 (12.3,17.9) |
| Deaths | Kingdom of Bahrain | 0.1 (-0.1, 0.5) | 0.3 (-0.3, 1.5) | 2.4 (0.1, 5.9) | 1.5 (0, 3.7) | 4.4 (4.1,4.7) |
| Deaths | Arab Republic of Egypt | 10.9 (-2.2, 31.9) | 0.2 (0, 0.6) | 203 (8.4, 459.3) | 1.6 (0.1, 3.6) | 7.7 (7.2,8.1) |
| Deaths | Islamic Republic of Iran | 2.6 (-4, 13.4) | 0 (-0.1, 0.2) | 90.8 (-2, 219.4) | 0.6 (0, 1.4) | 9.2 (9.0,9.5) |
| Deaths | Republic of Iraq | 5.7 (-0.9, 16.8) | 0.3 (-0.1, 1) | 41.3 (-1, 106.1) | 0.9 (0, 2.2) | 3.1 (2.8,3.4) |
| Deaths | Hashemite Kingdom of Jordan | 0.9 (-0.1, 2.5) | 0.3 (0, 0.9) | 14.1 (0.7, 33.5) | 1 (0, 2.3) | 3.8 (3.4,4.1) |
| Deaths | State of Kuwait | 0.4 (-0.1, 1.2) | 0.3 (-0.1, 1) | 7.4 (0.4, 16.3) | 1.4 (0.1, 3) | 4.8 (4.3,5.3) |
| Deaths | Lebanese Republic | 1.3 (-0.5, 4.5) | 0.3 (-0.1, 1) | 12.1 (-0.1, 29.8) | 0.9 (0, 2.3) | 4.6 (4.3,4.8) |
| Deaths | State of Libya | 1 (-0.8, 4.1) | 0.2 (-0.2, 1) | 19.9 (0.4, 49.2) | 1.9 (0, 4.7) | 6.9 (6.4,7.3) |
| Deaths | Kingdom of Morocco | -0.5 (-2.5, 2.4) | 0 (-0.1, 0.1) | 11.5 (-3.1, 37.5) | 0.1 (0, 0.5) | 17.6 (13.6,21.9) |
| Deaths | Palestine | 0.8 (-0.2, 2.6) | 0.4 (-0.1, 1.4) | 6.2 (0.1, 14.8) | 1.3 (0, 3) | 3.5 (3.4,3.7) |
| Deaths | Sultanate of Oman | 0 (-0.1, 0.2) | 0 (-0.1, 0.2) | 1.6 (0, 4) | 0.4 (0, 1) | 10.4 (9.5,11.2) |
| Deaths | State of Qatar | 0.1 (0, 0.4) | 0.7 (-0.2, 2.2) | 2.6 (0.1, 6.3) | 1.9 (0.1, 4.4) | 3.4 (2.9,4.0) |
| Deaths | Kingdom of Saudi Arabia | 1.6 (-0.4, 4.7) | 0.1 (0, 0.4) | 30.9 (1.2, 70.6) | 0.9 (0, 2) | 6.1 (5.5,6.7) |
| Deaths | Syrian Arab Republic | 1.8 (-0.8, 6.2) | 0.2 (-0.1, 0.6) | 28.1 (0.9, 67.9) | 1 (0, 2.4) | 6.2 (6.0,6.4) |
| Deaths | Republic of Tunisia | -0.1 (-1.6, 2.1) | 0 (-0.2, 0.2) | 10.5 (-0.9, 30.1) | 0.4 (0, 1.1) | 12.2 (10.4,14.1) |
| Deaths | Republic of Turkey | 33.2 (-14.1, 110) | 0.4 (-0.2, 1.5) | 335.2 (-0.1, 825) | 1.6 (0, 4) | 4.2 (3.8,4.5) |
| Deaths | United Arab Emirates | 0.4 (-0.2, 1.6) | 0.5 (-0.3, 2.1) | 16.8 (0.8, 37.1) | 3.1 (0.1, 6.9) | 7.4 (6.8,8.0) |
| Deaths | Republic of Yemen | -0.3 (-1.2, 0.6) | 0 (-0.1, 0.1) | 3.6 (-1.3, 12.2) | 0.1 (-0.1, 0.4) | 21.8 (17.4,26.4) |
| Deaths | Islamic Republic of Afghanistan | 0.5 (-1.8, 4.2) | 0 (-0.1, 0.2) | 3.4 (-1.3, 12.4) | 0.2 (-0.1, 0.7) | 8.9 (8.7,9.1) |
| Deaths | People's Republic of Bangladesh | -4.8 (-9.9, -0.7) | -0.1 (-0.1, 0) | -10.1 (-27.3, 7.7) | 0 (-0.1, 0) | NA (NA,NA) |
| Deaths | Kingdom of Bhutan | 0 (-0.1, 0) | 0 (-0.1, 0) | 0 (-0.1, 0.3) | 0 (-0.1, 0.2) | 243.1 (NaN,NaN) |
| Deaths | Republic of India | -36.4 (-70.3, -0.7) | 0 (-0.1, 0) | -18 (-165.6, 220.1) | 0 (-0.1, 0.1) | NA (NA,NA) |
| Deaths | Federal Democratic Republic of Nepal | -0.7 (-1.6, 0) | 0 (-0.1, 0) | -2 (-5.8, 1.7) | 0 (-0.1, 0) | NA (NA,NA) |
| Deaths | Islamic Republic of Pakistan | -8 (-14.7, -0.3) | -0.1 (-0.1, 0) | 3.5 (-19.9, 40.1) | 0 (-0.1, 0.2) | 2292.7 (NaN,NaN) |
| Deaths | Republic of Angola | -1 (-2.2, 0) | -0.1 (-0.3, 0) | -0.2 (-4.4, 5.8) | 0 (-0.2, 0.2) | NA (NA,NA) |
| Deaths | Central African Republic | -0.2 (-0.5, 0) | -0.1 (-0.2, 0) | 0 (-0.5, 0.8) | 0 (-0.1, 0.2) | NA (NA,NA) |
| Deaths | Republic of the Congo | -0.4 (-0.9, 0.3) | -0.2 (-0.5, 0.1) | 0.8 (-0.9, 3.7) | 0.1 (-0.2, 0.6) | 38.1 (23.5,54.5) |
| Deaths | Democratic Republic of the Congo | -2.5 (-6.3, 1.1) | -0.1 (-0.2, 0) | 5 (-5.7, 23.7) | 0.1 (-0.1, 0.3) | 56.9 (30.6,88.5) |
| Deaths | Republic of Equatorial Guinea | 0 (-0.1, 0.1) | -0.1 (-0.2, 0.1) | 0.4 (-0.1, 1.3) | 0.4 (-0.1, 1.3) | 21.9 (17.3,26.7) |
| Deaths | Gabonese Republic | 0 (-0.3, 0.5) | 0 (-0.3, 0.4) | 1.3 (-0.2, 3.9) | 0.6 (-0.1, 1.8) | 12.0 (9.6,14.4) |
| Deaths | Republic of Burundi | -0.5 (-1.1, 0) | -0.1 (-0.2, 0) | -0.6 (-1.5, 0.3) | -0.1 (-0.2, 0) | NA (NA,NA) |
| Deaths | Union of the Comoros | -0.1 (-0.1, 0) | -0.1 (-0.3, 0) | 0.1 (-0.1, 0.4) | 0 (-0.2, 0.4) | 102.0 (50.2,171.5) |
| Deaths | Republic of Djibouti | 0 (-0.1, 0) | -0.2 (-0.3, 0) | -0.2 (-0.4, 0) | -0.1 (-0.3, 0) | NA (NA,NA) |
| Deaths | State of Eritrea | -0.1 (-0.3, 0) | -0.1 (-0.1, 0) | -0.2 (-0.7, 0.5) | -0.1 (-0.1, 0.1) | NA (NA,NA) |
| Deaths | Federal Democratic Republic of Ethiopia | -2.2 (-5.2, 0) | -0.1 (-0.1, 0) | -3.3 (-9.7, 2.5) | 0 (-0.1, 0) | NA (NA,NA) |
| Deaths | Republic of Kenya | -1.2 (-2.8, 0.4) | -0.1 (-0.2, 0) | 3 (-6, 16.7) | 0 (-0.2, 0.3) | 103.0 (46.8,180.8) |
| Deaths | Republic of Madagascar | -0.7 (-1.6, 0.3) | -0.1 (-0.2, 0) | 0.2 (-1.8, 3.4) | 0 (-0.1, 0.2) | 207.0 (NaN,NaN) |
| Deaths | Republic of Malawi | -0.4 (-0.9, -0.1) | -0.1 (-0.1, 0) | -0.5 (-1.6, 0.7) | 0 (-0.1, 0) | NA (NA,NA) |
| Deaths | Republic of Mauritius | 0 (-0.4, 0.5) | 0 (-0.3, 0.3) | 1.1 (-0.6, 3.9) | 0.2 (-0.2, 0.9) | 15.5 (11.5,19.6) |
| Deaths | Republic of Mozambique | -0.4 (-0.8, 0) | 0 (-0.1, 0) | -0.2 (-1.3, 1.4) | 0 (-0.1, 0.1) | NA (NA,NA) |
| Deaths | Republic of Rwanda | -1.1 (-2.3, 0) | -0.2 (-0.4, 0) | -1.2 (-3.7, 1.3) | -0.1 (-0.3, 0.1) | NA (NA,NA) |
| Deaths | Republic of Seychelles | 0 (0, 0.1) | 0 (-0.4, 0.4) | 0.1 (0, 0.4) | 0.5 (-0.1, 1.6) | 11.4 (9.2,13.6) |
| Deaths | Federal Republic of Somalia | -0.4 (-1, 0) | -0.1 (-0.2, 0) | -0.5 (-1.8, 0.7) | 0 (-0.2, 0) | NA (NA,NA) |
| Deaths | United Republic of Tanzania | -1.4 (-4.7, 2.5) | -0.1 (-0.2, 0.1) | 6.9 (-3.5, 24.4) | 0.1 (-0.1, 0.5) | 16.2 (13.2,19.2) |
| Deaths | Republic of Uganda | -1.9 (-4, 0) | -0.1 (-0.3, 0) | -2.6 (-9.7, 5.6) | -0.1 (-0.4, 0.2) | NA (NA,NA) |
| Deaths | Republic of Zambia | -0.9 (-2, 0.1) | -0.2 (-0.4, 0) | 1.5 (-1.9, 7.5) | 0.1 (-0.2, 0.5) | 56.7 (35.7,80.9) |
| Deaths | Republic of Botswana | 0 (-0.3, 0.4) | 0 (-0.3, 0.3) | 1.6 (-0.1, 4.7) | 0.6 (0, 1.7) | 18.3 (15.8,20.9) |
| Deaths | Kingdom of Lesotho | 0.1 (-0.2, 0.6) | 0.1 (-0.1, 0.4) | 1.5 (-0.1, 4.6) | 0.7 (-0.1, 2.1) | 9.3 (8.4,10.3) |
| Deaths | Republic of Namibia | 0 (-0.1, 0.1) | 0 (-0.1, 0.1) | 0.5 (0, 1.3) | 0.2 (0, 0.5) | 9.4 (8.3,10.5) |
| Deaths | Republic of South Africa | 14.2 (-2.6, 39.7) | 0.3 (-0.1, 0.9) | 105.8 (0.7, 244.6) | 1.1 (0, 2.6) | 4.0 (3.6,4.3) |
| Deaths | Kingdom of Eswatini | 0.1 (-0.1, 0.6) | 0.3 (-0.2, 1.1) | 1.8 (0, 4.5) | 1.7 (0, 4.3) | 5.9 (5.2,6.7) |
| Deaths | Republic of Zimbabwe | -0.5 (-2.8, 2.6) | -0.1 (-0.4, 0.3) | 10.1 (-1.3, 28.6) | 0.7 (-0.1, 2) | 13.1 (9.3,17.0) |
| Deaths | Republic of Benin | -0.1 (-0.5, 0.4) | 0 (-0.1, 0.1) | 1.3 (-1.1, 5.2) | 0.1 (-0.1, 0.5) | 22.8 (17.8,28.0) |
| Deaths | Burkina Faso | -0.7 (-1.4, -0.1) | -0.1 (-0.2, 0) | -2.4 (-5.5, 0.3) | -0.1 (-0.3, 0) | NA (NA,NA) |
| Deaths | Republic of Cameroon | 0.1 (-1.2, 2.5) | 0 (-0.2, 0.3) | 11.9 (-1.2, 34.7) | 0.5 (-0.1, 1.4) | 15.3 (12.3,18.3) |
| Deaths | Republic of Cabo Verde | 0 (0, 0) | 0 (-0.1, 0) | 0.5 (-0.3, 1.7) | 0.5 (-0.3, 1.9) | 19.6 (15.9,23.5) |
| Deaths | Republic of Chad | -0.1 (-0.4, 0.2) | 0 (-0.1, 0) | 0.1 (-1.3, 2.3) | 0 (-0.1, 0.2) | NA (NA,NA) |
| Deaths | Republic of Côte d'Ivoire | -0.1 (-0.8, 0.8) | 0 (-0.1, 0.1) | 1.8 (-1.5, 7.2) | 0.1 (-0.1, 0.3) | 18.6 (14.0,23.3) |
| Deaths | Republic of the Gambia | 0 (-0.1, 0.1) | 0 (-0.1, 0.1) | 0.1 (-0.2, 0.6) | 0 (-0.1, 0.3) | 21.9 (18.7,25.2) |
| Deaths | Republic of Ghana | -0.7 (-1.9, 0.4) | -0.1 (-0.2, 0) | 7.6 (-3.9, 28.3) | 0.2 (-0.1, 0.8) | 34.6 (25.8,44.1) |
| Deaths | Republic of Guinea | -0.3 (-0.6, 0.1) | 0 (-0.1, 0) | -0.2 (-1.3, 1.1) | 0 (-0.1, 0.1) | NA (NA,NA) |
| Deaths | Republic of Guinea-Bissau | -0.1 (-0.2, 0) | -0.1 (-0.2, 0) | 0.1 (-0.2, 0.6) | 0 (-0.2, 0.4) | 175.6 (76.5,330.3) |
| Deaths | Republic of Liberia | 0 (-0.3, 0.4) | 0 (-0.1, 0.2) | 1.1 (-0.2, 3.4) | 0.2 (-0.1, 0.8) | 13.8 (12.4,15.2) |
| Deaths | Republic of Mali | -0.7 (-1.6, 0.1) | -0.1 (-0.2, 0) | -1.3 (-4, 1.7) | -0.1 (-0.2, 0.1) | NA (NA,NA) |
| Deaths | Islamic Republic of Mauritania | 0.2 (-0.2, 0.7) | 0.1 (-0.1, 0.3) | 2 (-0.1, 5.6) | 0.5 (0, 1.3) | 6.0 (5.8,6.2) |
| Deaths | Republic of the Niger | -0.2 (-0.5, 0.2) | 0 (-0.1, 0) | -0.1 (-1.8, 2.2) | 0 (-0.1, 0.1) | NA (NA,NA) |
| Deaths | Federal Republic of Nigeria | -1.2 (-4.6, 3.2) | 0 (-0.1, 0) | 14.1 (-4, 44.4) | 0.1 (0, 0.3) | 19.2 (15.4,23.2) |
| Deaths | Democratic Republic of Sao Tome and Principe | 0 (0, 0) | 0 (-0.1, 0) | 0 (0, 0) | 0.1 (0, 0.2) | 24.5 (19.2,30.1) |
| Deaths | Republic of Senegal | -0.2 (-0.8, 0.5) | 0 (-0.1, 0.1) | 1.3 (-2.2, 7) | 0.1 (-0.2, 0.4) | 27.5 (20.4,35.1) |
| Deaths | Republic of Sierra Leone | -0.3 (-0.6, 0.1) | -0.1 (-0.1, 0) | 0.1 (-1.1, 1.9) | 0 (-0.2, 0.2) | NA (NA,NA) |
| Deaths | Togolese Republic | -0.1 (-0.3, 0.1) | 0 (-0.1, 0) | 0.7 (-0.8, 3.2) | 0.1 (-0.1, 0.4) | 29.4 (23.0,36.1) |
| Deaths | American Samoa | 0 (0, 0.1) | 0.6 (-0.1, 1.5) | 0.1 (0, 0.3) | 1.3 (0, 2.9) | 3.2 (2.9,3.5) |
| Deaths | Bermuda | 0.1 (-0.1, 0.4) | 0.7 (-0.5, 2.8) | 0.5 (0, 1.2) | 1.6 (-0.1, 3.9) | 2.6 (2.4,2.9) |
| Deaths | Cook Islands | 0 (0, 0) | 0.5 (-0.1, 1.4) | 0.1 (0, 0.1) | 0.9 (0, 2.1) | 1.8 (1.7,2.0) |
| Deaths | Greenland | 0.1 (0, 0.4) | 2 (-0.4, 5.8) | 0.3 (0, 0.9) | 2.1 (-0.2, 5.6) | 0.2 (0.1,0.3) |
| Deaths | Guam | 0 (0, 0.1) | 0.2 (-0.2, 0.9) | 0.3 (0, 0.7) | 0.5 (-0.1, 1.4) | 3.6 (3.3,3.9) |
| Deaths | Principality of Monaco | 0.1 (-0.1, 0.5) | 0.9 (-0.3, 3.1) | 0.4 (-0.1, 1.2) | 1.7 (-0.2, 5.1) | 2.3 (2.2,2.3) |
| Deaths | Republic of Nauru | 0 (0, 0) | 0.5 (-0.2, 1.7) | 0 (0, 0) | 1.3 (0, 3.2) | 2.9 (2.8,3.0) |
| Deaths | Republic of Niue | 0 (0, 0) | 0.2 (-0.2, 0.8) | 0 (0, 0) | 0.9 (0, 2.4) | 4.8 (4.5,5.0) |
| Deaths | Northern Mariana Islands | 0 (0, 0) | 0.4 (-0.1, 1.2) | 0.2 (0, 0.4) | 1.6 (0, 3.8) | 5.4 (4.8,6.0) |
| Deaths | Republic of Palau | 0 (0, 0.1) | 0.9 (-0.4, 2.9) | 0.1 (0, 0.2) | 2.1 (0, 5.2) | 2.8 (2.6,2.9) |
| Deaths | Puerto Rico | 3.1 (-1.1, 9.8) | 0.4 (-0.1, 1.2) | 18.2 (-0.4, 44.2) | 1.2 (0, 2.8) | 3.4 (3.1,3.8) |
| Deaths | Saint Kitts and Nevis | 0 (0, 0.1) | 0.1 (-0.4, 1) | 0.1 (0, 0.4) | 0.9 (-0.1, 2.5) | 7.2 (6.5,7.9) |
| Deaths | Republic of San Marino | 0 (0, 0.1) | 0.5 (-0.4, 1.9) | 0.1 (0, 0.3) | 0.6 (-0.1, 2) | 2.0 (1.6,2.4) |
| Deaths | Tokelau | 0 (0, 0) | 0.2 (-0.1, 0.6) | 0 (0, 0) | 0.7 (0, 1.7) | 4.8 (4.5,5.0) |
| Deaths | Tuvalu | 0 (0, 0) | 0.1 (-0.1, 0.5) | 0 (0, 0) | 0.6 (0, 1.6) | 5.3 (5.2,5.5) |
| Deaths | United States Virgin Islands | 0.1 (0, 0.2) | 0.5 (-0.1, 1.4) | 0.3 (0, 0.8) | 0.8 (0, 2) | 1.9 (1.3,2.4) |
| Deaths | Republic of South Sudan | -0.8 (-1.7, -0.1) | -0.2 (-0.3, 0) | -1 (-2.4, 0.1) | -0.1 (-0.3, 0) | NA (NA,NA) |
| Deaths | Republic of Sudan | 0.6 (-1.1, 3.6) | 0 (-0.1, 0.2) | 13.3 (-0.7, 37.5) | 0.3 (0, 0.9) | 8.3 (8.1,8.5) |
