# Supplementary material for: Attributable burden of high BMI-related gastrointestinal tract cancers among middle-aged and elderly populations globally, 1990–2021 and projected to 2050: analysis of GBD 2021
Source: Front Nutr. 2026 Jan 2;12:1674621. doi: 10.3389/fnut.2025.1674621 (PMC12807987; doi:10.3389/fnut.2025.1674621)
Supplement: Supplementary file 6 [file Table_5.docx]

| **Measure** | **Location** | **Number 1990** | **ASMR 1990** | **Number 2021** | **ASMR 2021** | **EAPC (95% CI)** |
| --- | --- | --- | --- | --- | --- | --- |
| Deaths | Global | 8853.5 (3568.3, 14600.1) | 1 (0.4, 1.7) | 40958 (16553.4, 68880.6) | 2.2 (0.9, 3.6) | 2.4 (2.3,2.5) |
| Deaths | Low SDI | 342.8 (125.7, 620.2) | 0.7 (0.3, 1.3) | 1200.6 (444.7, 2072.3) | 1.1 (0.4, 1.9) | 1.3 (1.2,1.4) |
| Deaths | Low-middle SDI | 1081 (405.2, 1999.7) | 0.8 (0.3, 1.6) | 5458.2 (2236.4, 9174.8) | 1.8 (0.7, 2.9) | 2.6 (2.5,2.7) |
| Deaths | Middle SDI | 1560.8 (641.1, 2543.6) | 0.7 (0.3, 1.2) | 10697.7 (4318.6, 18265) | 1.8 (0.7, 3.1) | 3.1 (3.0,3.2) |
| Deaths | High-middle SDI | 2719.9 (1097, 4591.2) | 1.2 (0.5, 2.1) | 10074.4 (3958.5, 17746) | 2.3 (0.9, 4) | 1.9 (1.9,2.0) |
| Deaths | High SDI | 3134.7 (1294.3, 5335) | 1.3 (0.5, 2.2) | 13492.8 (5528.9, 22396.5) | 2.9 (1.2, 4.8) | 2.6 (2.4,2.8) |

****Global and SDI-Based Trends in High BMI-Attributable Liver Cancer Burden, 1990–2021****

****Global and SDI-Based Trends in High BMI-Attributable Liver Cancer Burden, 2000–2021****

| **Measure** | **Location** | **Number 2000** | **ASR 2000** | **Number 2021** | **ASR 2021** | **EAPC (95% CI)** |
| --- | --- | --- | --- | --- | --- | --- |
| Deaths | Global | 14788.1 (6060.4, 24199.1) | 1.4 (0.6, 2.3) | 40958 (16553.4, 68880.6) | 2.2 (0.9, 3.6) | 2.4 (2.3,2.5) |
| Deaths | Low SDI | 498.4 (182.9, 892) | 0.8 (0.3, 1.5) | 1200.6 (444.7, 2072.3) | 1.1 (0.4, 1.9) | 1.3 (1.2,1.4) |
| Deaths | Low-middle SDI | 1645.7 (633.6, 2809.8) | 1 (0.4, 1.7) | 5458.2 (2236.4, 9174.8) | 1.8 (0.7, 2.9) | 2.6 (2.5,2.7) |
| Deaths | Middle SDI | 2832.9 (1186.1, 4613.8) | 1 (0.4, 1.6) | 10697.7 (4318.6, 18265) | 1.8 (0.7, 3.1) | 3.1 (3.0,3.2) |
| Deaths | High-middle SDI | 4246.5 (1728.5, 7167.7) | 1.6 (0.7, 2.7) | 10074.4 (3958.5, 17746) | 2.3 (0.9, 4) | 1.9 (1.9,2.0) |
| Deaths | High SDI | 5547 (2293.6, 9337.9) | 1.9 (0.8, 3.2) | 13492.8 (5528.9, 22396.5) | 2.9 (1.2, 4.8) | 2.6 (2.4,2.8) |

| **Measure** | **Location** | **Number 2010** | **ASR 2010** | **Number 2021** | **ASR 2021** | **EAPC (95% CI)** |
| --- | --- | --- | --- | --- | --- | --- |
| Deaths | Global | 24117.4 (9671.1, 39603) | 1.8 (0.7, 2.9) | 40958 (16553.4, 68880.6) | 2.2 (0.9, 3.6) | 2.4 (2.3,2.5) |
| Deaths | Low SDI | 704.7 (261.7, 1222.2) | 0.9 (0.3, 1.6) | 1200.6 (444.7, 2072.3) | 1.1 (0.4, 1.9) | 1.3 (1.2,1.4) |
| Deaths | Low-middle SDI | 3134.3 (1216.8, 5238.5) | 1.4 (0.5, 2.4) | 5458.2 (2236.4, 9174.8) | 1.8 (0.7, 2.9) | 2.6 (2.5,2.7) |
| Deaths | Middle SDI | 5112.8 (2061.7, 8370) | 1.3 (0.5, 2.1) | 10697.7 (4318.6, 18265) | 1.8 (0.7, 3.1) | 3.1 (3.0,3.2) |
| Deaths | High-middle SDI | 6269.8 (2552.5, 10549.2) | 1.9 (0.8, 3.2) | 10074.4 (3958.5, 17746) | 2.3 (0.9, 4) | 1.9 (1.9,2.0) |
| Deaths | High SDI | 8871.8 (3616.8, 14773.3) | 2.5 (1, 4.1) | 13492.8 (5528.9, 22396.5) | 2.9 (1.2, 4.8) | 2.6 (2.4,2.8) |

****Global and SDI-Based Trends in High BMI-Attributable Liver Cancer Burden, 2010–2021****

| **Measure** | **Location** | **Number 1990** | **ASR 1990** | **Number 2021** | **ASR 2021** | **EAPC (95% CI)** |
| --- | --- | --- | --- | --- | --- | --- |
| DALYs (Disability-Adjusted Life Years) | Global | 223275.7 (90024.7, 368412.1) | 25.2 (10.1, 41.5) | 984496.2 (400302.3, 1663344.8) | 51 (20.7, 86.1) | 2.3 (2.3,2.4) |
| DALYs (Disability-Adjusted Life Years) | Low SDI | 9521 (3491.9, 17091.8) | 18 (6.6, 32.6) | 32943.1 (12165.3, 56942.4) | 28.3 (10.5, 49) | 1.2 (1.1,1.3) |
| DALYs (Disability-Adjusted Life Years) | Low-middle SDI | 28538.6 (10730.6, 52379.2) | 20.7 (7.8, 38.2) | 142906.1 (58806.8, 240367.7) | 43.7 (18, 73.5) | 2.7 (2.6,2.8) |
| DALYs (Disability-Adjusted Life Years) | Middle SDI | 41837.2 (17176.4, 68159.2) | 17.6 (7.2, 28.8) | 272263.9 (110282.1, 468479.3) | 43.5 (17.6, 74.7) | 3.1 (3.0,3.1) |
| DALYs (Disability-Adjusted Life Years) | High-middle SDI | 68455.1 (27578.8, 115842.8) | 30.1 (12.1, 51) | 242425 (94947.2, 430929.5) | 54.4 (21.3, 96.7) | 1.9 (1.8,2.0) |
| DALYs (Disability-Adjusted Life Years) | High SDI | 74570.2 (30922.3, 127187.1) | 31.5 (13.1, 53.8) | 293158.6 (121267.6, 485843.6) | 67.5 (28, 111.9) | 2.5 (2.3,2.7) |

****Global and SDI-Based Trends in High BMI-Attributable Liver Cancer Burden, 1990–2021****

| **Measure** | **Location** | **Number 1990** | **ASR 1990** | **Number 2021** | **ASR 2021** | **EAPC (95% CI)** |
| --- | --- | --- | --- | --- | --- | --- |
| YLDs (Years Lived with Disability) | Global | 2013.3 (787, 3514.3) | 0.2 (0.1, 0.4) | 10382.1 (4049.3, 18420.6) | 0.5 (0.2, 1) | 2.8 (2.7,2.9) |
| YLDs (Years Lived with Disability) | Low SDI | 73.4 (25.6, 139.6) | 0.1 (0.1, 0.3) | 258.5 (90.7, 465.2) | 0.2 (0.1, 0.4) | 1.3 (1.2,1.4) |
| YLDs (Years Lived with Disability) | Low-middle SDI | 224.7 (78.7, 429.4) | 0.2 (0.1, 0.3) | 1150.2 (458.6, 2063.1) | 0.4 (0.1, 0.6) | 2.8 (2.6,2.9) |
| YLDs (Years Lived with Disability) | Middle SDI | 338.3 (134.4, 586.5) | 0.1 (0.1, 0.3) | 2562 (988.8, 4582.1) | 0.4 (0.2, 0.8) | 3.5 (3.4,3.6) |
| YLDs (Years Lived with Disability) | High-middle SDI | 595.4 (228.8, 1051.8) | 0.3 (0.1, 0.5) | 2455.9 (939, 4532) | 0.6 (0.2, 1) | 2.4 (2.3,2.4) |
| YLDs (Years Lived with Disability) | High SDI | 778.4 (312, 1382.1) | 0.3 (0.1, 0.6) | 3947.9 (1581.9, 6994.2) | 0.9 (0.4, 1.6) | 3.2 (3.0,3.5) |

****Global and SDI-Based Trends in High BMI-Attributable Liver Cancer Burden, 1990–2021****

| **Measure** | **Location** | **Number 1990** | **ASR 1990** | **Number 2021** | **ASR 2021** | **EAPC (95% CI)** |
| --- | --- | --- | --- | --- | --- | --- |
| YLLs (Years of Life Lost) | Global | 221262.4 (89320.3, 365128.5) | 24.9 (10.1, 41.1) | 974114.1 (396565.9, 1646562.6) | 50.4 (20.5, 85.2) | 2.3 (2.3,2.4) |
| YLLs (Years of Life Lost) | Low SDI | 9447.6 (3466.2, 16958.1) | 17.9 (6.6, 32.3) | 32684.6 (12070.8, 56495.8) | 28.1 (10.4, 48.6) | 1.2 (1.1,1.3) |
| YLLs (Years of Life Lost) | Low-middle SDI | 28313.8 (10646.6, 51940.7) | 20.5 (7.7, 37.9) | 141756 (58367.8, 238455.3) | 43.4 (17.8, 72.9) | 2.7 (2.6,2.8) |
| YLLs (Years of Life Lost) | Middle SDI | 41498.9 (17053.4, 67604.8) | 17.5 (7.2, 28.5) | 269701.9 (109363.3, 464103.3) | 43.1 (17.5, 74) | 3.1 (3.0,3.1) |
| YLLs (Years of Life Lost) | High-middle SDI | 67859.7 (27374.8, 114804.3) | 29.9 (12.1, 50.5) | 239969.1 (94091.8, 426819.5) | 53.8 (21.1, 95.7) | 1.9 (1.8,2.0) |
| YLLs (Years of Life Lost) | High SDI | 73791.8 (30642.4, 125928.3) | 31.2 (13, 53.3) | 289210.8 (119768.3, 479533.1) | 66.6 (27.7, 110.5) | 2.5 (2.3,2.7) |

****Global and SDI-Based Trends in High BMI-Attributable Liver Cancer Burden, 1990–2021****

****Burden of Liver Cancer Deaths Attributable to High BMI in 21 GBD Regions, 1990–2021****

| Measure | Location | Number 1990 | ASMR 1990 | Number 2021 | ASMR 2021 | EAPC (95% CI) |
| --- | --- | --- | --- | --- | --- | --- |
| Deaths | Global | 8853.5 (3568.3, 14600.1) | 1 (0.4, 1.7) | 40958 (16553.4, 68880.6) | 2.2 (0.9, 3.6) | 2.4 (2.3,2.5) |
| Deaths | Andean Latin America | 37.3 (14.1, 71.2) | 0.9 (0.3, 1.6) | 202.6 (80.6, 369.5) | 1.6 (0.6, 2.9) | 1.9 (1.7,2.1) |
| Deaths | Australasia | 44.1 (17, 80.3) | 0.9 (0.3, 1.6) | 448.9 (182.5, 781.2) | 3.8 (1.6, 6.6) | 4.9 (4.7,5.1) |
| Deaths | Caribbean | 37.8 (14.9, 63.4) | 0.7 (0.3, 1.1) | 125.1 (50.7, 218.5) | 1.1 (0.4, 1.8) | 1.1 (0.8,1.3) |
| Deaths | Central Asia | 382.8 (159.2, 652.3) | 3.7 (1.5, 6.3) | 791.3 (324.4, 1415.7) | 4.4 (1.8, 7.9) | 0.4 (0.3,0.5) |
| Deaths | Central Europe | 623.4 (245.7, 1075.4) | 1.9 (0.7, 3.2) | 1114.6 (451.1, 1927.8) | 2.3 (0.9, 3.9) | 0.6 (0.4,0.7) |
| Deaths | Central Latin America | 242.8 (97.1, 426.9) | 1.4 (0.6, 2.5) | 1196.2 (496.1, 2056.4) | 2.2 (0.9, 3.8) | 1.5 (1.3,1.8) |
| Deaths | Central Sub-Saharan Africa | 39.9 (11.2, 99.1) | 0.8 (0.2, 2) | 194 (54, 490.8) | 1.7 (0.5, 4.4) | 2.1 (2.0,2.2) |
| Deaths | East Asia | 1312 (542.7, 2183.9) | 0.7 (0.3, 1.1) | 10838.7 (4134.9, 19316.8) | 2.2 (0.8, 3.9) | 4.1 (4.0,4.2) |
| Deaths | Eastern Europe | 576.4 (232.1, 974.5) | 0.9 (0.4, 1.5) | 1381.5 (554.9, 2359.5) | 1.8 (0.7, 3) | 2.3 (2.0,2.6) |
| Deaths | Eastern Sub-Saharan Africa | 85.8 (33, 148.5) | 0.5 (0.2, 0.9) | 422.9 (149.9, 788.1) | 1.2 (0.4, 2.2) | 2.4 (2.3,2.5) |
| Deaths | High-income Asia Pacific | 752.5 (325.4, 1254.8) | 1.7 (0.7, 2.8) | 1964.2 (772.5, 3473.3) | 1.9 (0.7, 3.3) | -0.3 (-0.7,0.2) |
| Deaths | High-income North America | 844.2 (338.2, 1513.3) | 1.1 (0.4, 2) | 5517.3 (2347.1, 8972) | 3.8 (1.6, 6.1) | 4.1 (3.9,4.3) |
| Deaths | North Africa and Middle East | 909.9 (320.9, 1766.2) | 2.6 (0.9, 5) | 4577.7 (1905.1, 7746.7) | 4.8 (2, 8.1) | 2.1 (2.1,2.2) |
| Deaths | Oceania | 9.6 (3.3, 19.4) | 1.4 (0.5, 2.9) | 29.1 (11.3, 54.1) | 1.7 (0.7, 3.2) | 0.5 (0.3,0.6) |
| Deaths | South Asia | 137.6 (54.5, 241.5) | 0.1 (0, 0.2) | 1655 (646.3, 2886.2) | 0.5 (0.2, 0.9) | 5.4 (5.3,5.5) |
| Deaths | Southeast Asia | 251.4 (102.7, 436.5) | 0.4 (0.2, 0.8) | 1710.1 (658.3, 3034.3) | 1.2 (0.4, 2.1) | 3.1 (2.9,3.3) |
| Deaths | Southern Latin America | 45 (17.5, 80.4) | 0.4 (0.2, 0.8) | 278 (111.3, 479.4) | 1.4 (0.6, 2.5) | 4.5 (4.3,4.8) |
| Deaths | Southern Sub-Saharan Africa | 106.9 (38.1, 197.4) | 1.9 (0.7, 3.5) | 588 (247.3, 1006.5) | 4.9 (2.1, 8.5) | 2.6 (2.0,3.3) |
| Deaths | Tropical Latin America | 123 (48.2, 223.1) | 0.6 (0.2, 1.2) | 706.3 (280.2, 1218) | 1.3 (0.5, 2.2) | 2.8 (2.6,3.1) |
| Deaths | Western Europe | 1935.4 (769.4, 3382.7) | 1.5 (0.6, 2.6) | 6059.2 (2373.1, 10652.6) | 2.9 (1.1, 5.1) | 2.1 (2.0,2.3) |
| Deaths | Western Sub-Saharan Africa | 355.7 (125.6, 671.5) | 1.9 (0.7, 3.6) | 1157.5 (454.1, 1992.7) | 2.9 (1.1, 5) | 1.1 (1.0,1.2) |

****Burden of Liver Cancer Deaths Attributable to High BMI in Country 1990–2021****

| **Measure** | **Location** | **Number 1990** | **ASMR 1990** | **Number 2021** | **ASMR 2021** | **EAPC (95% CI)** |
| --- | --- | --- | --- | --- | --- | --- |
| Deaths | China | 1258.2 (518, 2099.4) | 0.7 (0.3, 1.1) | 10426 (3969.4, 18586.7) | 2.2 (0.8, 3.8) | 4.1 (3.9,4.2) |
| Deaths | Democratic People's Republic of Korea | 15.7 (5.1, 33.4) | 0.5 (0.1, 1) | 72.4 (22.6, 164.3) | 1.1 (0.3, 2.4) | 2.5 (2.3,2.7) |
| Deaths | Taiwan (Province of China) | 38.1 (14.9, 72.2) | 1.1 (0.4, 2) | 340.4 (128.4, 639.3) | 3.6 (1.4, 6.7) | 5.1 (4.1,6.1) |
| Deaths | Kingdom of Cambodia | 4.4 (1.2, 12) | 0.4 (0.1, 1.2) | 20.3 (5.4, 48.4) | 0.7 (0.2, 1.7) | 1.4 (1.3,1.5) |
| Deaths | Republic of Indonesia | 33.2 (12.3, 67.2) | 0.1 (0.1, 0.3) | 331.1 (96.1, 712.9) | 0.6 (0.2, 1.3) | 4.9 (4.7,5.1) |
| Deaths | Lao People's Democratic Republic | 2.4 (0.8, 5.6) | 0.5 (0.2, 1.2) | 11.8 (3.5, 26.9) | 1.1 (0.3, 2.6) | 2.5 (2.3,2.7) |
| Deaths | Malaysia | 15.9 (5.4, 31.8) | 0.8 (0.3, 1.6) | 111.8 (41.5, 211.1) | 1.8 (0.7, 3.4) | 2.7 (2.2,3.2) |
| Deaths | Republic of Maldives | 0.1 (0, 0.3) | 0.6 (0.2, 1.3) | 0.7 (0.2, 1.6) | 1 (0.3, 2.2) | 1.3 (1.2,1.4) |
| Deaths | Republic of the Union of Myanmar | 13 (3, 33.4) | 0.2 (0.1, 0.6) | 54 (13.6, 138.4) | 0.5 (0.1, 1.2) | 2.0 (2.0,2.1) |
| Deaths | Republic of the Philippines | 45.1 (15.8, 85.8) | 0.7 (0.2, 1.3) | 266.5 (95, 496.6) | 1.4 (0.5, 2.7) | 2.3 (2.1,2.5) |
| Deaths | Democratic Socialist Republic of Sri Lanka | 5 (1.6, 10.5) | 0.2 (0.1, 0.5) | 21.4 (6.8, 47.6) | 0.3 (0.1, 0.8) | 0.9 (0.5,1.3) |
| Deaths | Kingdom of Thailand | 102.3 (36.5, 218.9) | 1.3 (0.5, 2.7) | 654.5 (229.4, 1282.1) | 2.6 (0.9, 5.2) | 2.0 (1.6,2.4) |
| Deaths | Democratic Republic of Timor-Leste | 0 (0, 0.1) | 0.1 (0, 0.1) | 0.3 (0.1, 0.8) | 0.2 (0, 0.4) | 2.9 (2.6,3.3) |
| Deaths | Socialist Republic of Viet Nam | 27.7 (10.4, 56.5) | 0.3 (0.1, 0.6) | 233.4 (71.2, 540.4) | 1 (0.3, 2.3) | 4.4 (4.1,4.7) |
| Deaths | Republic of Fiji | 1.7 (0.6, 3.6) | 2.2 (0.7, 4.7) | 6.8 (2.6, 12.7) | 3.9 (1.5, 7.4) | 2.0 (1.8,2.2) |
| Deaths | Republic of Kiribati | 0.3 (0.1, 0.5) | 3 (1, 5.9) | 0.7 (0.3, 1.5) | 4.3 (1.5, 8.7) | 1.0 (0.7,1.2) |
| Deaths | Republic of the Marshall Islands | 0.1 (0, 0.1) | 1.9 (0.5, 4.2) | 0.2 (0.1, 0.5) | 2.9 (1, 6.3) | 1.3 (1.2,1.4) |
| Deaths | Federated States of Micronesia | 0.3 (0.1, 0.7) | 2.9 (0.9, 6.3) | 0.7 (0.2, 1.5) | 4 (1.4, 8.4) | 0.9 (0.7,1.1) |
| Deaths | Independent State of Papua New Guinea | 2.4 (0.6, 7.1) | 0.5 (0.1, 1.6) | 7.8 (1.9, 22.4) | 0.6 (0.1, 1.8) | 0.1 (-0.1,0.3) |
| Deaths | Independent State of Samoa | 0.7 (0.3, 1.4) | 3.7 (1.3, 7.4) | 1.4 (0.5, 2.7) | 4.4 (1.6, 8.4) | 0.3 (0.2,0.5) |
| Deaths | Solomon Islands | 0.5 (0.1, 1.3) | 1.4 (0.3, 3.9) | 1.5 (0.4, 3.3) | 1.8 (0.5, 4.1) | 0.8 (0.7,1.0) |
| Deaths | Kingdom of Tonga | 2 (0.7, 3.8) | 15.3 (5.5, 29.7) | 3.6 (1.4, 7) | 20.5 (7.7, 39.6) | 0.7 (0.4,1.0) |
| Deaths | Republic of Vanuatu | 0.1 (0, 0.3) | 1 (0.3, 2.5) | 0.7 (0.2, 1.5) | 1.7 (0.6, 3.8) | 1.9 (1.7,2.0) |
| Deaths | Republic of Armenia | 28.2 (10.9, 49.9) | 4.9 (1.9, 8.6) | 43.9 (17.9, 77) | 4.5 (1.8, 7.9) | -0.6 (-1.2,0.0) |
| Deaths | Republic of Azerbaijan | 38 (14.4, 74) | 3.5 (1.3, 6.9) | 137.5 (44.1, 304) | 6.1 (1.9, 13.4) | 1.7 (1.5,1.8) |
| Deaths | Georgia | 35.2 (13.5, 63.1) | 2.4 (0.9, 4.4) | 33.4 (13.3, 59.6) | 2.5 (1, 4.5) | -0.5 (-1.7,0.8) |
| Deaths | Republic of Kazakhstan | 139.3 (54.5, 254.7) | 4.9 (1.9, 9) | 130 (49.7, 233.2) | 3.4 (1.3, 6.1) | -2.0 (-2.4,-1.7) |
| Deaths | Kyrgyz Republic | 22.1 (8.1, 40.4) | 3.3 (1.2, 6) | 27.3 (10.3, 49.9) | 2.6 (1, 4.8) | -0.6 (-1.5,0.3) |
| Deaths | Mongolia | 54.1 (19.5, 104.3) | 23.7 (8.5, 45.9) | 190.8 (74.3, 347) | 40.3 (15.6, 73.6) | 2.1 (1.7,2.5) |
| Deaths | Republic of Tajikistan | 15.1 (4.8, 31.6) | 2.6 (0.8, 5.6) | 32.7 (11.6, 66) | 2.8 (1, 5.7) | 0.2 (0.0,0.4) |
| Deaths | Turkmenistan | 10.9 (4.1, 20.5) | 2.5 (1, 4.8) | 28.2 (9.7, 55) | 3.1 (1.1, 6) | 0.8 (0.5,1.1) |
| Deaths | Republic of Uzbekistan | 39.8 (14.8, 71) | 1.6 (0.6, 2.8) | 167.5 (64, 316.5) | 2.8 (1.1, 5.4) | 1.8 (1.5,2.0) |
| Deaths | Republic of Albania | 25.5 (9.7, 49.2) | 6.1 (2.3, 11.8) | 48.7 (16.1, 100.8) | 4.9 (1.6, 10.1) | -1.2 (-1.7,-0.7) |
| Deaths | Bosnia and Herzegovina | 27.7 (10, 52.2) | 3 (1.1, 5.7) | 57.1 (21.6, 104) | 4 (1.5, 7.3) | 0.5 (0.3,0.6) |
| Deaths | Republic of Bulgaria | 129.8 (48.8, 232.4) | 4.8 (1.8, 8.5) | 79.5 (29.9, 149) | 2.6 (1, 4.8) | -1.9 (-2.4,-1.4) |
| Deaths | Republic of Croatia | 18.4 (6.8, 34.2) | 1.4 (0.5, 2.6) | 45.9 (17.1, 83.5) | 2.3 (0.9, 4.2) | 2.3 (1.8,2.8) |
| Deaths | Czech Republic | 104 (40.9, 186) | 3.4 (1.3, 6) | 102.7 (40, 187.3) | 2.1 (0.8, 3.8) | -1.7 (-2.0,-1.5) |
| Deaths | Hungary | 63.8 (23.7, 113.7) | 1.9 (0.7, 3.5) | 66.1 (27.4, 116.9) | 1.5 (0.6, 2.7) | -1.1 (-1.8,-0.5) |
| Deaths | North Macedonia | 23 (8.7, 42) | 5.7 (2.1, 10.4) | 43.8 (16.5, 82.3) | 6.4 (2.4, 12) | 0.0 (-0.2,0.2) |
| Deaths | Montenegro | 5.2 (2, 9.5) | 3.8 (1.4, 6.9) | 12.8 (5, 23.3) | 5.9 (2.3, 10.7) | 1.6 (1.5,1.7) |
| Deaths | Republic of Poland | 32.8 (12.8, 57.6) | 0.3 (0.1, 0.6) | 190.5 (76.1, 342.7) | 1.2 (0.5, 2.1) | 4.9 (4.2,5.6) |
| Deaths | Romania | 47.2 (18, 84.2) | 0.8 (0.3, 1.3) | 188.1 (71.6, 344.3) | 2.3 (0.9, 4.3) | 4.1 (3.8,4.4) |
| Deaths | Republic of Serbia | 68.3 (24.6, 134.7) | 2.7 (1, 5.4) | 156.9 (60.6, 294.3) | 4.2 (1.6, 7.9) | 0.8 (0.6,1.1) |
| Deaths | Slovak Republic | 52.4 (20.2, 96.8) | 3.9 (1.5, 7.3) | 68 (26.8, 130.1) | 3.2 (1.2, 6.1) | -1.2 (-1.4,-1.1) |
| Deaths | Republic of Slovenia | 15.3 (5.8, 27.5) | 2.8 (1.1, 5) | 38.2 (15.1, 68.6) | 3.8 (1.5, 6.9) | 1.0 (0.8,1.2) |
| Deaths | Republic of Belarus | 36.4 (14, 68.6) | 1.2 (0.5, 2.3) | 71.2 (27.4, 134) | 2 (0.8, 3.7) | 1.2 (1.0,1.4) |
| Deaths | Republic of Estonia | 8.7 (3.3, 15.4) | 1.9 (0.7, 3.3) | 17.3 (6.7, 30.8) | 2.9 (1.1, 5.2) | 1.2 (0.9,1.5) |
| Deaths | Republic of Latvia | 12.3 (4.7, 22.5) | 1.5 (0.6, 2.8) | 21.2 (8.3, 38.7) | 2.4 (1, 4.4) | 1.0 (0.6,1.3) |
| Deaths | Republic of Lithuania | 11.5 (4.4, 21.2) | 1.1 (0.4, 2.1) | 32.6 (12.9, 58.8) | 2.6 (1, 4.6) | 2.5 (2.2,2.7) |
| Deaths | Republic of Moldova | 17.1 (6.7, 30.2) | 1.7 (0.7, 3.1) | 31.4 (12.5, 53) | 2.3 (0.9, 4) | 0.1 (-0.9,1.0) |
| Deaths | Russian Federation | 333.4 (134.7, 557.9) | 0.8 (0.3, 1.4) | 1039.1 (417.9, 1788.4) | 1.9 (0.8, 3.3) | 3.4 (3.0,3.9) |
| Deaths | Ukraine | 157 (60.6, 281.4) | 1 (0.4, 1.7) | 168.7 (66.5, 300.9) | 1 (0.4, 1.8) | -0.5 (-1.3,0.3) |
| Deaths | Brunei Darussalam | 0.4 (0.1, 0.9) | 2 (0.7, 4.2) | 3.3 (1.1, 6.3) | 3.9 (1.3, 7.5) | 1.9 (1.8,2.1) |
| Deaths | Japan | 550.5 (237.2, 915.3) | 1.4 (0.6, 2.4) | 1282.5 (516.6, 2252.2) | 1.4 (0.6, 2.5) | -0.9 (-1.4,-0.3) |
| Deaths | Republic of Korea | 197.1 (78.8, 379) | 3 (1.2, 5.7) | 639.8 (230.5, 1297.4) | 3 (1.1, 6.1) | 0.0 (-0.0,0.1) |
| Deaths | Republic of Singapore | 4.5 (1.8, 8.3) | 0.9 (0.4, 1.7) | 38.7 (14.3, 72) | 2 (0.7, 3.8) | 2.5 (1.9,3.2) |
| Deaths | Australia | 35.2 (13.4, 65.6) | 0.8 (0.3, 1.5) | 397.6 (161.5, 698.7) | 4 (1.6, 7.1) | 5.2 (5.1,5.4) |
| Deaths | New Zealand | 8.8 (3.5, 16.2) | 1 (0.4, 1.9) | 51.2 (20.5, 89.6) | 2.8 (1.1, 4.8) | 3.1 (2.9,3.4) |
| Deaths | Principality of Andorra | 0.6 (0.2, 1.2) | 4.4 (1.4, 9.7) | 1.9 (0.7, 4.1) | 5.5 (1.9, 11.7) | 0.9 (0.7,1.1) |
| Deaths | Republic of Austria | 31.7 (11.9, 58.7) | 1.2 (0.5, 2.2) | 111.9 (41.6, 211.6) | 2.8 (1, 5.3) | 2.7 (2.5,3.0) |
| Deaths | Kingdom of Belgium | 38.3 (14.7, 70.4) | 1.1 (0.4, 2) | 119.5 (44.7, 217.5) | 2.3 (0.8, 4.1) | 2.4 (2.0,2.8) |
| Deaths | Republic of Cyprus | 1.8 (0.6, 3.6) | 1.1 (0.4, 2.3) | 8.1 (3, 16.6) | 1.9 (0.7, 3.8) | 2.1 (1.9,2.2) |
| Deaths | Kingdom of Denmark | 9.5 (3.7, 17) | 0.5 (0.2, 1) | 40.5 (15.1, 74) | 1.5 (0.6, 2.8) | 3.2 (2.8,3.6) |
| Deaths | Republic of Finland | 20.9 (7.8, 38.9) | 1.3 (0.5, 2.4) | 77.3 (29.6, 142.7) | 2.6 (1, 4.8) | 2.4 (2.3,2.5) |
| Deaths | French Republic | 301.8 (118.2, 540.6) | 1.7 (0.7, 3) | 1076.4 (416, 1967.8) | 3.4 (1.3, 6.3) | 2.1 (1.8,2.4) |
| Deaths | Federal Republic of Germany | 429 (159.8, 787.4) | 1.5 (0.6, 2.8) | 1154.2 (438.7, 2144.8) | 2.7 (1, 4.9) | 2.2 (2.0,2.4) |
| Deaths | Hellenic Republic | 27.4 (10.5, 50.6) | 0.8 (0.3, 1.5) | 139.7 (52.8, 253.8) | 2.7 (1, 4.8) | 4.0 (3.6,4.3) |
| Deaths | Republic of Iceland | 0.8 (0.3, 1.4) | 1.2 (0.4, 2.3) | 3.6 (1.3, 6.6) | 2.7 (1, 5.1) | 2.4 (2.1,2.7) |
| Deaths | Ireland | 7.2 (2.8, 13.5) | 0.8 (0.3, 1.5) | 37.6 (14.4, 67.8) | 2.1 (0.8, 3.8) | 3.5 (3.3,3.7) |
| Deaths | State of Israel | 12 (4.5, 22) | 1.1 (0.4, 2) | 40 (15.5, 73.6) | 1.4 (0.6, 2.7) | 0.8 (0.7,1.0) |
| Deaths | Republic of Italy | 485.7 (192.6, 873.9) | 2.4 (1, 4.4) | 808 (316.6, 1473.9) | 2.4 (1, 4.4) | -0.4 (-0.7,-0.1) |
| Deaths | Grand Duchy of Luxembourg | 2 (0.7, 3.5) | 1.6 (0.6, 2.9) | 6.5 (2.5, 11.6) | 2.7 (1.1, 4.9) | 1.8 (1.6,2.1) |
| Deaths | Republic of Malta | 0.6 (0.2, 1.1) | 0.6 (0.2, 1.1) | 3.5 (1.3, 6.5) | 1.6 (0.6, 2.9) | 3.1 (2.9,3.3) |
| Deaths | Kingdom of the Netherlands | 19.4 (7.5, 35.5) | 0.4 (0.2, 0.8) | 104.5 (38.5, 192.6) | 1.3 (0.5, 2.4) | 3.7 (3.6,3.9) |
| Deaths | Kingdom of Norway | 8.7 (3.4, 15.3) | 0.6 (0.2, 1) | 36.2 (14, 63.5) | 1.6 (0.6, 2.8) | 3.2 (2.8,3.6) |
| Deaths | Portuguese Republic | 21.6 (8, 41.1) | 0.7 (0.3, 1.3) | 142.3 (53.8, 267) | 2.7 (1, 5.1) | 4.2 (3.9,4.5) |
| Deaths | Kingdom of Spain | 247.9 (93.5, 457.6) | 2 (0.8, 3.7) | 865.7 (325, 1551.7) | 4 (1.5, 7.2) | 1.9 (1.5,2.2) |
| Deaths | Kingdom of Sweden | 34 (12.7, 62) | 1 (0.4, 1.9) | 71.9 (26.9, 132.6) | 1.5 (0.6, 2.8) | 2.1 (1.2,3.0) |
| Deaths | Swiss Confederation | 36.6 (13.9, 66.9) | 1.6 (0.6, 2.9) | 77.5 (30.4, 142.5) | 1.9 (0.7, 3.5) | 0.8 (0.7,0.9) |
| Deaths | United Kingdom of Great Britain and Northern Ireland | 196.3 (77.1, 358.5) | 1 (0.4, 1.8) | 1125.7 (454.7, 1959.7) | 3.8 (1.5, 6.6) | 5.2 (5.0,5.5) |
| Deaths | Argentine Republic | 21.4 (8.1, 39.7) | 0.3 (0.1, 0.6) | 107.7 (42.5, 188.5) | 0.9 (0.3, 1.5) | 4.4 (3.9,4.8) |
| Deaths | Republic of Chile | 19.5 (7.3, 35.8) | 0.9 (0.3, 1.7) | 150.6 (59.3, 266.5) | 2.6 (1, 4.7) | 3.9 (3.7,4.2) |
| Deaths | Eastern Republic of Uruguay | 4.1 (1.5, 7.6) | 0.5 (0.2, 0.9) | 19.7 (7.5, 35.7) | 1.7 (0.6, 3) | 4.8 (4.5,5.1) |
| Deaths | Canada | 67.4 (26.2, 127.7) | 0.9 (0.4, 1.8) | 498.1 (189.3, 879.2) | 3.1 (1.2, 5.4) | 4.2 (4.1,4.3) |
| Deaths | United States of America | 776.5 (309.9, 1386.6) | 1.1 (0.5, 2) | 5018.5 (2135.8, 8142) | 3.8 (1.6, 6.2) | 4.1 (3.9,4.4) |
| Deaths | Antigua and Barbuda | 0.1 (0, 0.2) | 1 (0.4, 1.9) | 0.4 (0.1, 0.7) | 1.6 (0.6, 2.9) | 1.0 (0.6,1.4) |
| Deaths | Commonwealth of the Bahamas | 0.5 (0.2, 0.9) | 1.5 (0.6, 2.8) | 1.8 (0.7, 3.3) | 2 (0.7, 3.6) | 0.7 (0.4,1.0) |
| Deaths | Barbados | 0.6 (0.2, 1) | 0.9 (0.3, 1.7) | 2.1 (0.8, 3.8) | 1.8 (0.7, 3.3) | 2.0 (1.8,2.1) |
| Deaths | Belize | 0.2 (0.1, 0.4) | 1.1 (0.4, 2) | 1.1 (0.5, 2) | 1.8 (0.8, 3.1) | 1.1 (0.6,1.6) |
| Deaths | Republic of Cuba | 13.7 (5.1, 24.4) | 0.6 (0.2, 1.1) | 36.2 (13.6, 65.9) | 0.8 (0.3, 1.5) | 0.3 (-0.1,0.8) |
| Deaths | Commonwealth of Dominica | 0.2 (0.1, 0.4) | 1.7 (0.6, 3.2) | 0.6 (0.2, 1.1) | 3.1 (1.2, 5.9) | 1.9 (1.6,2.1) |
| Deaths | Dominican Republic | 2.3 (0.8, 5) | 0.3 (0.1, 0.6) | 19.5 (6.7, 41.1) | 0.9 (0.3, 1.9) | 3.9 (3.7,4.0) |
| Deaths | Grenada | 0.1 (0, 0.2) | 0.7 (0.2, 1.3) | 0.5 (0.2, 0.9) | 1.9 (0.7, 3.6) | 3.2 (2.8,3.5) |
| Deaths | Republic of Guyana | 0.7 (0.3, 1.3) | 0.9 (0.3, 1.7) | 1.6 (0.6, 3) | 1.1 (0.4, 2.1) | 0.7 (0.4,1.0) |
| Deaths | Republic of Haiti | 1 (0.3, 2.5) | 0.1 (0, 0.4) | 4.5 (1.2, 11.3) | 0.3 (0.1, 0.8) | 2.6 (2.5,2.7) |
| Deaths | Jamaica | 1.5 (0.6, 2.9) | 0.4 (0.2, 0.7) | 7.1 (2.8, 12.9) | 1 (0.4, 1.9) | 3.3 (2.9,3.7) |
| Deaths | Saint Lucia | 0.1 (0, 0.2) | 0.6 (0.2, 1.2) | 0.4 (0.2, 0.8) | 0.8 (0.3, 1.4) | -0.1 (-0.5,0.2) |
| Deaths | Saint Vincent and the Grenadines | 0.1 (0, 0.2) | 0.6 (0.2, 1.2) | 0.3 (0.1, 0.6) | 1 (0.4, 1.9) | 1.1 (0.7,1.6) |
| Deaths | Republic of Suriname | 0.3 (0.1, 0.5) | 0.5 (0.2, 0.9) | 1.5 (0.5, 2.9) | 1 (0.3, 2.1) | 2.4 (2.2,2.6) |
| Deaths | Republic of Trinidad and Tobago | 1.8 (0.7, 3.2) | 1 (0.4, 1.8) | 6.1 (2.3, 10.9) | 1.4 (0.5, 2.5) | 0.6 (0.3,0.9) |
| Deaths | Plurinational State of Bolivia | 6.2 (1.9, 14.1) | 0.9 (0.3, 2) | 37 (12.2, 77.9) | 1.9 (0.6, 4) | 2.3 (2.2,2.3) |
| Deaths | Republic of Ecuador | 15.4 (5.7, 29.8) | 1.4 (0.5, 2.7) | 71.8 (27.2, 129.5) | 2 (0.8, 3.7) | 1.3 (0.7,1.9) |
| Deaths | Republic of Peru | 15.7 (5.5, 32.2) | 0.6 (0.2, 1.3) | 93.8 (34.9, 185.2) | 1.3 (0.5, 2.6) | 2.2 (2.0,2.4) |
| Deaths | Republic of Colombia | 35.6 (13.5, 65.5) | 1 (0.4, 1.8) | 163.8 (62.9, 300) | 1.4 (0.5, 2.5) | 1.0 (0.5,1.6) |
| Deaths | Republic of Costa Rica | 7.5 (2.9, 14.3) | 2.1 (0.8, 3.9) | 47.4 (18.4, 85.6) | 3.9 (1.5, 7.1) | 1.9 (1.5,2.3) |
| Deaths | Republic of El Salvador | 6.1 (2.3, 11.5) | 1 (0.4, 1.8) | 16.8 (6.6, 30.8) | 1.3 (0.5, 2.3) | 0.8 (0.3,1.3) |
| Deaths | Republic of Guatemala | 22.7 (9, 40) | 3.2 (1.3, 5.7) | 80.3 (32, 140.9) | 3.5 (1.4, 6.1) | -0.1 (-2.0,1.8) |
| Deaths | Republic of Honduras | 3.7 (1, 8.5) | 0.9 (0.2, 2) | 32.8 (10.9, 65.5) | 2.5 (0.8, 5) | 3.6 (3.3,3.8) |
| Deaths | United Mexican States | 92.1 (36.1, 165.5) | 1.1 (0.4, 1.9) | 678.7 (282.1, 1176) | 2.5 (1, 4.4) | 2.8 (2.6,3.1) |
| Deaths | Republic of Nicaragua | 5.4 (1.9, 10.2) | 1.7 (0.6, 3.2) | 24.3 (8.9, 47) | 2.4 (0.9, 4.6) | 1.5 (1.1,2.0) |
| Deaths | Republic of Panama | 7.6 (3, 13.4) | 2.5 (1, 4.4) | 28.1 (11.4, 48.3) | 2.9 (1.2, 5) | 1.0 (0.3,1.6) |
| Deaths | Bolivarian Republic of Venezuela | 62 (23.7, 113.4) | 3.1 (1.2, 5.7) | 124.1 (48.9, 224.7) | 1.9 (0.8, 3.5) | -1.2 (-2.5,0.1) |
| Deaths | Federative Republic of Brazil | 119.1 (46.5, 216) | 0.6 (0.2, 1.1) | 680 (269.8, 1173.4) | 1.2 (0.5, 2.1) | 2.8 (2.6,3.1) |
| Deaths | Republic of Paraguay | 3.9 (1.4, 7.7) | 0.8 (0.3, 1.7) | 26.3 (9.2, 52.4) | 2.1 (0.7, 4.2) | 3.9 (3.5,4.3) |
| Deaths | People's Democratic Republic of Algeria | 10.8 (3.8, 21.7) | 0.4 (0.1, 0.8) | 86.5 (30.8, 168.2) | 1.2 (0.4, 2.3) | 3.3 (3.2,3.3) |
| Deaths | Kingdom of Bahrain | 1.4 (0.5, 2.7) | 4 (1.4, 7.9) | 7 (2.6, 13.5) | 4.4 (1.6, 8.6) | -0.6 (-1.0,-0.2) |
| Deaths | Arab Republic of Egypt | 524.7 (168.3, 1159.1) | 9.9 (3.1, 22.3) | 2641.7 (1049, 4717.4) | 20.5 (8.1, 36.5) | 2.7 (2.5,2.9) |
| Deaths | Islamic Republic of Iran | 36.1 (13.7, 63.3) | 0.7 (0.3, 1.2) | 304.5 (125.7, 508.7) | 1.9 (0.8, 3.2) | 3.0 (2.2,3.8) |
| Deaths | Republic of Iraq | 43.2 (14.6, 83.3) | 2.6 (0.9, 5.1) | 178.2 (66, 342) | 3.8 (1.4, 7.3) | 1.3 (1.0,1.7) |
| Deaths | Hashemite Kingdom of Jordan | 3.9 (1.3, 8) | 1.4 (0.5, 2.9) | 22.7 (9.1, 43) | 1.5 (0.6, 2.9) | -0.3 (-0.5,-0.1) |
| Deaths | State of Kuwait | 2.9 (1.1, 5.2) | 2.3 (0.9, 4.3) | 6.6 (2.7, 11.4) | 1.3 (0.5, 2.2) | -1.4 (-2.4,-0.3) |
| Deaths | Lebanese Republic | 8.2 (2.9, 17.4) | 1.7 (0.6, 3.7) | 24.9 (9.4, 46.7) | 1.9 (0.7, 3.6) | 0.4 (0.3,0.6) |
| Deaths | State of Libya | 10.4 (3.5, 20.6) | 2.6 (0.9, 5.2) | 75.3 (27.4, 143.6) | 7.1 (2.6, 13.7) | 3.5 (3.4,3.6) |
| Deaths | Kingdom of Morocco | 3.1 (1, 6.4) | 0.1 (0, 0.2) | 21.1 (7.4, 40.4) | 0.3 (0.1, 0.5) | 3.3 (3.2,3.4) |
| Deaths | Palestine | 8 (2.7, 15.7) | 4.5 (1.5, 8.9) | 27.5 (10.6, 50.3) | 5.6 (2.1, 10.2) | 0.5 (0.3,0.6) |
| Deaths | Sultanate of Oman | 1.6 (0.5, 3.3) | 1.1 (0.4, 2.4) | 12.6 (4.6, 24) | 3.1 (1.1, 6) | 3.9 (3.6,4.2) |
| Deaths | State of Qatar | 1.4 (0.5, 2.8) | 7.6 (2.6, 15.3) | 21.4 (8.2, 41.2) | 15.2 (5.8, 29) | 1.4 (1.0,1.9) |
| Deaths | Kingdom of Saudi Arabia | 44.2 (14.7, 87.7) | 3.7 (1.2, 7.4) | 207.3 (76.6, 399.2) | 6.2 (2.3, 11.8) | 1.1 (1.0,1.3) |
| Deaths | Syrian Arab Republic | 36.6 (12.2, 72.1) | 3.4 (1.1, 6.8) | 147.3 (53.1, 278.7) | 5.3 (1.9, 10) | 1.2 (0.9,1.4) |
| Deaths | Republic of Tunisia | 5.6 (2, 11.1) | 0.5 (0.2, 1) | 38.3 (13, 77.8) | 1.3 (0.5, 2.7) | 3.1 (3.1,3.2) |
| Deaths | Republic of Turkey | 108.4 (38.9, 210.5) | 1.5 (0.5, 2.9) | 489.9 (182.4, 933.2) | 2.4 (0.9, 4.6) | 1.7 (1.3,2.0) |
| Deaths | United Arab Emirates | 3.6 (1.1, 7.4) | 4.7 (1.5, 9.4) | 81.7 (31.9, 156.1) | 11.1 (4.3, 20.9) | 3.0 (2.7,3.4) |
| Deaths | Republic of Yemen | 5.2 (1.2, 14.1) | 0.5 (0.1, 1.4) | 30.8 (8.7, 69.9) | 1.1 (0.3, 2.6) | 2.5 (2.3,2.7) |
| Deaths | Islamic Republic of Afghanistan | 29.3 (8.9, 63.6) | 1.8 (0.5, 4) | 58.4 (19, 121.6) | 3 (1, 6.4) | 1.8 (1.6,2.0) |
| Deaths | People's Republic of Bangladesh | 6.3 (2.4, 13.2) | 0.1 (0, 0.1) | 75.4 (24.3, 162) | 0.2 (0.1, 0.5) | 5.0 (4.8,5.2) |
| Deaths | Kingdom of Bhutan | 0.3 (0.1, 0.6) | 0.5 (0.2, 1.1) | 1.6 (0.5, 3.6) | 1.3 (0.4, 2.8) | 3.0 (2.9,3.1) |
| Deaths | Republic of India | 95.8 (36.2, 176.7) | 0.1 (0, 0.2) | 1244.8 (471.9, 2205.9) | 0.5 (0.2, 0.8) | 5.6 (5.5,5.7) |
| Deaths | Federal Democratic Republic of Nepal | 1.6 (0.5, 4.1) | 0.1 (0, 0.2) | 22.1 (6.9, 47) | 0.4 (0.1, 0.9) | 6.3 (6.0,6.7) |
| Deaths | Islamic Republic of Pakistan | 33.6 (12.6, 68.4) | 0.3 (0.1, 0.6) | 311 (112.6, 613.1) | 1.2 (0.4, 2.3) | 5.4 (5.0,5.8) |
| Deaths | Republic of Angola | 9.3 (1.2, 31.3) | 1 (0.1, 3.6) | 52.3 (9.5, 158) | 2 (0.4, 6.2) | 1.6 (1.4,1.8) |
| Deaths | Central African Republic | 2.2 (0.6, 6.3) | 0.8 (0.2, 2.4) | 7.8 (1.9, 22.4) | 1.6 (0.4, 4.6) | 1.6 (1.5,1.7) |
| Deaths | Republic of the Congo | 4.7 (1.2, 13.7) | 1.9 (0.5, 5.5) | 17.1 (4.4, 46) | 3 (0.7, 8.3) | 1.0 (0.8,1.2) |
| Deaths | Democratic Republic of the Congo | 19.2 (4.8, 49.9) | 0.6 (0.1, 1.5) | 100.2 (25.3, 268.5) | 1.4 (0.3, 3.7) | 2.6 (2.5,2.8) |
| Deaths | Republic of Equatorial Guinea | 0.4 (0.1, 1) | 0.9 (0.2, 2.2) | 3.1 (0.9, 6.6) | 3.2 (1, 6.8) | 4.3 (4.0,4.6) |
| Deaths | Gabonese Republic | 4.1 (1, 11) | 3.2 (0.8, 8.8) | 13.6 (4.6, 28.5) | 6 (2, 12.8) | 1.7 (1.5,2.0) |
| Deaths | Republic of Burundi | 1.6 (0.4, 4.3) | 0.3 (0.1, 0.9) | 3.5 (1, 9) | 0.3 (0.1, 0.9) | -0.3 (-0.6,-0.0) |
| Deaths | Union of the Comoros | 0.2 (0.1, 0.6) | 0.6 (0.2, 1.5) | 1.5 (0.5, 3.2) | 1.5 (0.5, 3.2) | 2.4 (2.3,2.5) |
| Deaths | Republic of Djibouti | 0.1 (0, 0.1) | 0.2 (0.1, 0.5) | 0.6 (0.2, 1.5) | 0.5 (0.1, 1.1) | 2.7 (2.5,2.8) |
| Deaths | State of Eritrea | 0.5 (0.2, 1.3) | 0.2 (0.1, 0.6) | 2.8 (0.8, 6.8) | 0.5 (0.1, 1.2) | 2.2 (2.1,2.3) |
| Deaths | Federal Democratic Republic of Ethiopia | 12.4 (4.5, 26.6) | 0.3 (0.1, 0.6) | 29.8 (10.2, 59.6) | 0.3 (0.1, 0.7) | -0.2 (-0.4,0.1) |
| Deaths | Republic of Kenya | 5.4 (1.8, 11.4) | 0.3 (0.1, 0.6) | 59.8 (21.1, 107.8) | 1.2 (0.4, 2.2) | 4.8 (4.6,4.9) |
| Deaths | Republic of Madagascar | 2.8 (0.8, 6.9) | 0.3 (0.1, 0.6) | 14.3 (3.8, 32.7) | 0.6 (0.2, 1.5) | 2.5 (2.4,2.7) |
| Deaths | Republic of Malawi | 1.9 (0.7, 4.1) | 0.2 (0.1, 0.5) | 14.4 (4.4, 31.8) | 1 (0.3, 2.1) | 3.5 (2.9,4.1) |
| Deaths | Republic of Mauritius | 1.6 (0.6, 2.9) | 1 (0.4, 1.8) | 1.3 (0.5, 2.3) | 0.3 (0.1, 0.5) | 1.9 (-0.1,3.9) |
| Deaths | Republic of Mozambique | 18 (5.5, 40.7) | 1.4 (0.4, 3.2) | 108.6 (27.6, 278.3) | 4.6 (1.1, 11.7) | 4.1 (4.0,4.2) |
| Deaths | Republic of Rwanda | 2.7 (0.9, 6.4) | 0.4 (0.1, 1) | 9.3 (2.5, 22.3) | 0.7 (0.2, 1.7) | 0.7 (0.3,1.1) |
| Deaths | Republic of Seychelles | 0.3 (0.1, 0.5) | 2.1 (0.8, 4.1) | 0.6 (0.2, 1.2) | 2.2 (0.7, 4.3) | 0.4 (-0.1,0.9) |
| Deaths | Federal Republic of Somalia | 3.6 (1.1, 8.2) | 0.7 (0.2, 1.5) | 15.8 (4, 39.3) | 1.3 (0.3, 3.2) | 1.9 (1.9,2.0) |
| Deaths | United Republic of Tanzania | 21.4 (6.5, 45.7) | 0.9 (0.3, 1.9) | 96.8 (31.8, 198.3) | 1.8 (0.6, 3.8) | 2.1 (2.0,2.2) |
| Deaths | Republic of Uganda | 8.4 (2.9, 17.8) | 0.6 (0.2, 1.2) | 39.4 (12.8, 81.4) | 1.2 (0.4, 2.6) | 1.8 (1.5,2.2) |
| Deaths | Republic of Zambia | 5.5 (1.7, 12.8) | 0.9 (0.3, 2.1) | 21.9 (5.1, 63.8) | 1.6 (0.4, 4.5) | 0.5 (-0.1,1.1) |
| Deaths | Republic of Botswana | 1.6 (0.3, 4.5) | 1.4 (0.3, 4) | 9 (2.6, 21.2) | 3.1 (0.9, 7.1) | 2.6 (2.3,2.9) |
| Deaths | Kingdom of Lesotho | 3.6 (0.9, 10.1) | 2 (0.5, 5.7) | 16.4 (4.8, 38.8) | 7.4 (2.2, 17.3) | 4.7 (4.2,5.2) |
| Deaths | Republic of Namibia | 0.8 (0.2, 1.7) | 0.6 (0.2, 1.4) | 5.3 (1.8, 10.4) | 2 (0.7, 3.9) | 3.5 (2.9,4.0) |
| Deaths | Republic of South Africa | 80.1 (27.7, 153.4) | 1.9 (0.6, 3.6) | 436.3 (185.6, 740.2) | 4.5 (1.9, 7.7) | 2.3 (1.7,2.9) |
| Deaths | Kingdom of Eswatini | 2.3 (0.7, 4.9) | 4.3 (1.4, 9.5) | 15.2 (3.8, 38.3) | 14.3 (3.8, 35.2) | 4.1 (3.1,5.1) |
| Deaths | Republic of Zimbabwe | 18.6 (5.7, 38.4) | 2.1 (0.6, 4.4) | 105.9 (32.9, 225.9) | 7.7 (2.3, 16.6) | 4.2 (3.5,4.9) |
| Deaths | Republic of Benin | 15.9 (4.6, 39.5) | 3.7 (1.1, 9.2) | 47.3 (15.2, 98.5) | 4.3 (1.4, 8.9) | 0.1 (-0.0,0.3) |
| Deaths | Burkina Faso | 15.5 (4.3, 39) | 1.6 (0.4, 4) | 36.1 (9.2, 87.2) | 1.8 (0.5, 4.2) | 0.1 (-0.1,0.3) |
| Deaths | Republic of Cameroon | 53.3 (17.9, 112) | 5.5 (1.9, 11.7) | 173.1 (49.6, 392.6) | 6.8 (1.9, 15.3) | 0.3 (0.2,0.5) |
| Deaths | Republic of Cabo Verde | 0.9 (0.2, 2.1) | 1.7 (0.5, 4.1) | 4.4 (1.4, 9.2) | 4.7 (1.5, 9.9) | 2.9 (2.7,3.2) |
| Deaths | Republic of Chad | 10.9 (2.5, 29.3) | 1.8 (0.4, 4.8) | 32.4 (9.4, 78.5) | 2.7 (0.8, 6.6) | 0.9 (0.8,1.1) |
| Deaths | Republic of Côte d'Ivoire | 11 (3.4, 23.3) | 1.2 (0.4, 2.6) | 34.2 (10.4, 75.7) | 1.4 (0.4, 3.2) | 0.1 (-0.0,0.3) |
| Deaths | Republic of the Gambia | 4 (1.3, 8.1) | 5.2 (1.7, 10.6) | 20.1 (6.5, 44.3) | 9.7 (3.1, 21.3) | 1.6 (1.4,1.8) |
| Deaths | Republic of Ghana | 17.9 (5.1, 42) | 1.3 (0.4, 3.2) | 93 (27.6, 206.8) | 2.9 (0.8, 6.4) | 1.7 (1.3,2.0) |
| Deaths | Republic of Guinea | 23.2 (7.5, 49.4) | 3.2 (1, 6.8) | 55.3 (17.6, 120) | 4.6 (1.4, 10) | 1.3 (1.2,1.4) |
| Deaths | Republic of Guinea-Bissau | 3 (0.8, 7.1) | 3.4 (1, 8.2) | 6.8 (2.2, 14.1) | 4.5 (1.5, 9.5) | 0.6 (0.5,0.7) |
| Deaths | Republic of Liberia | 16.1 (5.1, 35.5) | 6.4 (2, 14.1) | 37 (12.2, 75) | 8.5 (2.8, 17.2) | 0.8 (0.5,1.0) |
| Deaths | Republic of Mali | 26.8 (8.8, 56.2) | 3 (1, 6.3) | 85.4 (25.8, 180) | 4.5 (1.4, 9.6) | 1.1 (1.0,1.2) |
| Deaths | Islamic Republic of Mauritania | 25.7 (5.1, 68.3) | 12.5 (2.4, 33.5) | 48.6 (15.4, 101.3) | 11.3 (3.5, 23.4) | -0.9 (-1.1,-0.7) |
| Deaths | Republic of the Niger | 11 (2.7, 28.5) | 1.8 (0.5, 4.8) | 28 (8, 64.9) | 1.6 (0.5, 3.7) | -0.7 (-0.8,-0.6) |
| Deaths | Federal Republic of Nigeria | 92.7 (25.1, 232.1) | 1 (0.3, 2.5) | 373.8 (134.4, 681.5) | 2.1 (0.8, 3.8) | 2.1 (2.0,2.2) |
| Deaths | Democratic Republic of Sao Tome and Principe | 0.1 (0, 0.1) | 0.5 (0.2, 1) | 0.2 (0.1, 0.5) | 0.9 (0.3, 2) | 1.7 (1.5,1.8) |
| Deaths | Republic of Senegal | 15.6 (4.9, 33.2) | 2.2 (0.7, 4.8) | 43.7 (15.4, 91.3) | 2.7 (0.9, 5.6) | 0.2 (-0.0,0.3) |
| Deaths | Republic of Sierra Leone | 8.6 (2.1, 24.1) | 2 (0.5, 5.5) | 18.1 (5.7, 38.8) | 2.3 (0.7, 4.9) | 0.3 (0.1,0.4) |
| Deaths | Togolese Republic | 3.7 (1.2, 7.9) | 1.5 (0.5, 3.1) | 19.9 (6.2, 43.2) | 2.6 (0.8, 5.7) | 1.7 (1.6,1.8) |
| Deaths | American Samoa | 0.2 (0.1, 0.3) | 3.1 (1.1, 6) | 0.7 (0.3, 1.2) | 5.9 (2.4, 10.4) | 2.2 (1.8,2.5) |
| Deaths | Bermuda | 0.2 (0.1, 0.4) | 1.6 (0.6, 3) | 0.4 (0.1, 0.7) | 1.2 (0.5, 2.2) | -1.0 (-1.5,-0.6) |
| Deaths | Cook Islands | 0.2 (0.1, 0.4) | 7.2 (2.6, 13.9) | 0.6 (0.2, 1) | 9.5 (3.8, 17.6) | 0.9 (0.8,1.1) |
| Deaths | Greenland | 0.2 (0.1, 0.4) | 3.2 (1.2, 6) | 0.7 (0.2, 1.3) | 4.1 (1.5, 8) | 0.9 (0.7,1.1) |
| Deaths | Guam | 0.3 (0.1, 0.5) | 1.5 (0.5, 3) | 1.7 (0.6, 3.1) | 3.5 (1.3, 6.4) | 3.2 (3.0,3.4) |
| Deaths | Principality of Monaco | 0.3 (0.1, 0.6) | 2 (0.7, 3.9) | 1.1 (0.4, 2.3) | 5.2 (1.8, 10.2) | 3.5 (2.9,4.1) |
| Deaths | Republic of Nauru | 0 (0, 0.1) | 4.5 (1.6, 9) | 0.1 (0, 0.1) | 4.8 (1.8, 9.6) | -0.3 (-0.5,-0.1) |
| Deaths | Republic of Niue | 0 (0, 0) | 2.3 (0.8, 5) | 0 (0, 0) | 4 (1.3, 8.4) | 1.5 (1.4,1.7) |
| Deaths | Northern Mariana Islands | 0.1 (0, 0.2) | 3.2 (1.1, 6.3) | 0.8 (0.3, 1.4) | 6.2 (2.4, 11.2) | 2.0 (1.9,2.2) |
| Deaths | Republic of Palau | 0.1 (0, 0.2) | 4.6 (1.4, 9.9) | 0.3 (0.1, 0.7) | 5.6 (2, 11.5) | 0.4 (0.2,0.7) |
| Deaths | Puerto Rico | 12.7 (4.9, 23.3) | 1.6 (0.6, 3) | 35.6 (14.5, 63.6) | 2.3 (1, 4.1) | 0.7 (0.4,1.0) |
| Deaths | Saint Kitts and Nevis | 0.1 (0, 0.2) | 1.4 (0.5, 2.5) | 0.3 (0.1, 0.6) | 2.2 (0.8, 4) | 1.1 (0.6,1.5) |
| Deaths | Republic of San Marino | 0.1 (0, 0.2) | 1 (0.3, 1.9) | 0.2 (0.1, 0.5) | 1.5 (0.5, 3.2) | 1.8 (1.6,1.9) |
| Deaths | Tokelau | 0 (0, 0) | 2 (0.6, 4.6) | 0 (0, 0) | 3.9 (1.4, 8.2) | 2.0 (1.8,2.2) |
| Deaths | Tuvalu | 0 (0, 0.1) | 2.3 (0.8, 4.9) | 0.1 (0, 0.2) | 3.6 (1.3, 7.6) | 1.4 (1.3,1.6) |
| Deaths | United States Virgin Islands | 0.2 (0.1, 0.4) | 1.2 (0.4, 2.4) | 0.8 (0.3, 1.6) | 1.9 (0.6, 3.8) | 1.5 (1.3,1.7) |
| Deaths | Republic of South Sudan | 1.2 (0.4, 2.7) | 0.2 (0.1, 0.5) | 4 (1.2, 9.5) | 0.5 (0.1, 1.1) | 2.4 (2.1,2.7) |
| Deaths | Republic of Sudan | 20.9 (5.7, 50.6) | 1.1 (0.3, 2.7) | 89.9 (30.6, 174.3) | 2.3 (0.8, 4.5) | 2.5 (2.3,2.8) |

****Burden of Liver Cancer Deaths Attributable to High BMI in Country 1990–2021****

| **Measure** | **Location** | **Number 1990** | **ASR 1990** | **Number 2021** | **ASR 2021** | **EAPC (95% CI)** |
| --- | --- | --- | --- | --- | --- | --- |
| DALYs (Disability-Adjusted Life Years) | China | 34806.1 (14241.8, 58193.1) | 17.7 (7.3, 29.6) | 268110.6 (101697.3, 483063.5) | 53.4 (20.2, 96.1) | 3.9 (3.8,4.1) |
| DALYs (Disability-Adjusted Life Years) | Democratic People's Republic of Korea | 420.5 (137.5, 874.8) | 11.1 (3.6, 23.5) | 1511.5 (487.6, 3374.9) | 21 (6.7, 47.1) | 1.8 (1.7,2.0) |
| DALYs (Disability-Adjusted Life Years) | Taiwan (Province of China) | 1043 (409.2, 1977.2) | 27.6 (10.8, 52.5) | 7831 (2945.3, 14865.8) | 82.8 (31.1, 157.2) | 4.6 (3.6,5.6) |
| DALYs (Disability-Adjusted Life Years) | Kingdom of Cambodia | 128.2 (33.4, 341.3) | 11.9 (3.1, 32.1) | 571.4 (152.2, 1359.8) | 18.9 (5, 45) | 1.3 (1.2,1.4) |
| DALYs (Disability-Adjusted Life Years) | Republic of Indonesia | 978 (361.6, 1973.2) | 4.1 (1.5, 8.2) | 9507.7 (2839.6, 20009.5) | 15.7 (4.6, 33.6) | 4.8 (4.6,5.0) |
| DALYs (Disability-Adjusted Life Years) | Lao People's Democratic Republic | 69.1 (22.9, 157.7) | 14.1 (4.7, 32.3) | 340.8 (100, 774.3) | 30.2 (8.9, 68.8) | 2.6 (2.3,2.8) |
| DALYs (Disability-Adjusted Life Years) | Malaysia | 447.1 (152.6, 887.4) | 21.3 (7.3, 42.4) | 2909.7 (1079.4, 5491.5) | 44.5 (16.5, 84.2) | 2.4 (1.9,2.9) |
| DALYs (Disability-Adjusted Life Years) | Republic of Maldives | 4.4 (1.4, 9.5) | 17.7 (5.7, 38.5) | 21 (7, 44.9) | 26.7 (8.9, 57.3) | 1.0 (0.8,1.1) |
| DALYs (Disability-Adjusted Life Years) | Republic of the Union of Myanmar | 376.7 (87.8, 957.8) | 6.7 (1.6, 17.2) | 1524.6 (390.8, 3848) | 12.9 (3.3, 32.7) | 1.9 (1.8,2.0) |
| DALYs (Disability-Adjusted Life Years) | Republic of the Philippines | 1323.8 (465.1, 2495.8) | 18.8 (6.6, 35.7) | 7432 (2632.8, 13826.6) | 37.7 (13.4, 70.3) | 2.1 (1.9,2.3) |
| DALYs (Disability-Adjusted Life Years) | Democratic Socialist Republic of Sri Lanka | 134 (42.9, 277.5) | 5.5 (1.8, 11.5) | 534.8 (172, 1195.8) | 8.5 (2.7, 19.1) | 0.8 (0.4,1.2) |
| DALYs (Disability-Adjusted Life Years) | Kingdom of Thailand | 2976 (1053.5, 6357.1) | 34.5 (12.3, 73.7) | 17005.3 (6028.9, 33196.6) | 67.6 (23.9, 132) | 1.7 (1.3,2.2) |
| DALYs (Disability-Adjusted Life Years) | Democratic Republic of Timor-Leste | 1.1 (0.4, 2.3) | 1.7 (0.7, 3.6) | 8.6 (2.4, 21.2) | 4.4 (1.3, 11) | 3.2 (2.9,3.6) |
| DALYs (Disability-Adjusted Life Years) | Socialist Republic of Viet Nam | 766 (286.2, 1565.8) | 8.3 (3.1, 17) | 6808.2 (2068.1, 15739.3) | 27.5 (8.4, 63.4) | 4.6 (4.3,4.9) |
| DALYs (Disability-Adjusted Life Years) | Republic of Fiji | 51 (16.3, 105) | 59.9 (19.1, 124) | 188.1 (71.8, 352.2) | 99.8 (38, 186.7) | 1.9 (1.7,2.1) |
| DALYs (Disability-Adjusted Life Years) | Republic of Kiribati | 7.8 (2.8, 15.3) | 86.7 (30.8, 171.5) | 21.6 (7.8, 43.1) | 117.2 (42, 235.9) | 0.7 (0.5,1.0) |
| DALYs (Disability-Adjusted Life Years) | Republic of the Marshall Islands | 1.8 (0.5, 4) | 50.8 (15.1, 113.4) | 6.8 (2.3, 14.4) | 77 (26.1, 164.8) | 1.2 (1.0,1.3) |
| DALYs (Disability-Adjusted Life Years) | Federated States of Micronesia | 8.9 (2.9, 19.4) | 82.6 (27.2, 180.5) | 21.4 (7.5, 45.1) | 109 (37.8, 231) | 0.7 (0.6,0.9) |
| DALYs (Disability-Adjusted Life Years) | Independent State of Papua New Guinea | 73.4 (17.3, 219) | 15.8 (3.8, 47.1) | 244.9 (57.8, 699.2) | 18.2 (4.3, 52) | 0.2 (-0.0,0.3) |
| DALYs (Disability-Adjusted Life Years) | Independent State of Samoa | 20.3 (7.4, 40.3) | 99.8 (36.5, 199) | 39.9 (14.8, 76.3) | 116.1 (43, 222.6) | 0.2 (0.1,0.4) |
| DALYs (Disability-Adjusted Life Years) | Solomon Islands | 14.6 (3.3, 41.1) | 40.9 (9.4, 115) | 46.6 (13.8, 102.3) | 53.5 (15.9, 118.3) | 0.8 (0.7,1.0) |
| DALYs (Disability-Adjusted Life Years) | Kingdom of Tonga | 57.7 (20.8, 109.7) | 429 (154.1, 820.4) | 100.3 (37.9, 194.1) | 550.7 (208.3, 1064.6) | 0.5 (0.3,0.8) |
| DALYs (Disability-Adjusted Life Years) | Republic of Vanuatu | 4.2 (1.2, 10.3) | 28.1 (8.2, 69.4) | 20.1 (6.4, 44.3) | 47.1 (15, 104) | 1.8 (1.6,1.9) |
| DALYs (Disability-Adjusted Life Years) | Republic of Armenia | 707 (274.2, 1249.4) | 112.5 (43.6, 199) | 1024.1 (418.2, 1794.5) | 104.4 (42.7, 182.8) | -0.5 (-1.0,0.1) |
| DALYs (Disability-Adjusted Life Years) | Republic of Azerbaijan | 1017.9 (386.3, 1968.2) | 86.8 (32.9, 169.1) | 3629.3 (1169.3, 8062) | 145.1 (46.8, 320.7) | 1.4 (1.3,1.6) |
| DALYs (Disability-Adjusted Life Years) | Georgia | 933.7 (358.6, 1676.4) | 62.6 (24.1, 112.3) | 832.6 (332.5, 1485.6) | 64.9 (26, 115.6) | -0.5 (-1.8,0.9) |
| DALYs (Disability-Adjusted Life Years) | Republic of Kazakhstan | 3849 (1494.8, 7040.2) | 128 (50.2, 234.4) | 3285.1 (1257.9, 5874) | 79.7 (30.5, 142.9) | -2.5 (-2.8,-2.1) |
| DALYs (Disability-Adjusted Life Years) | Kyrgyz Republic | 598.4 (220, 1095.6) | 85.7 (31.6, 156.9) | 728.6 (273.7, 1332.4) | 64.3 (24.3, 117.5) | -0.9 (-1.8,-0.0) |
| DALYs (Disability-Adjusted Life Years) | Mongolia | 1458 (532.9, 2811.1) | 621.1 (226.5, 1199.1) | 5097.5 (1980.7, 9245.3) | 953.4 (370.8, 1734.6) | 1.6 (1.3,2.0) |
| DALYs (Disability-Adjusted Life Years) | Republic of Tajikistan | 378.5 (123.3, 761.1) | 62.2 (20.1, 126.5) | 855.1 (303.6, 1723.6) | 64.9 (23.2, 130.6) | 0.1 (-0.1,0.2) |
| DALYs (Disability-Adjusted Life Years) | Turkmenistan | 300.9 (113.5, 569.8) | 66.6 (25.2, 126.2) | 780.2 (268.6, 1527.5) | 79.3 (27.4, 154.8) | 0.9 (0.6,1.3) |
| DALYs (Disability-Adjusted Life Years) | Republic of Uzbekistan | 1077.5 (399.4, 1915.2) | 41.4 (15.3, 73.6) | 4563.3 (1736.2, 8653.6) | 71.8 (27.4, 135.8) | 1.7 (1.5,1.9) |
| DALYs (Disability-Adjusted Life Years) | Republic of Albania | 588.4 (221.2, 1142.2) | 132.1 (49.8, 255.4) | 1040.9 (342.1, 2145.2) | 103.8 (34.2, 214) | -1.2 (-1.7,-0.8) |
| DALYs (Disability-Adjusted Life Years) | Bosnia and Herzegovina | 734.9 (268.3, 1382.9) | 75 (27.4, 140.7) | 1292.3 (486.8, 2366.4) | 92.7 (34.9, 170) | 0.2 (0.1,0.4) |
| DALYs (Disability-Adjusted Life Years) | Republic of Bulgaria | 3185.2 (1203.2, 5696) | 110.9 (41.8, 198.2) | 1812.3 (684.2, 3403.7) | 61.7 (23.3, 116) | -1.7 (-2.2,-1.2) |
| DALYs (Disability-Adjusted Life Years) | Republic of Croatia | 455.3 (167.9, 842.8) | 32.1 (11.8, 59.5) | 1009.2 (376.9, 1832.7) | 53.5 (20, 97) | 2.3 (1.8,2.8) |
| DALYs (Disability-Adjusted Life Years) | Czech Republic | 2455.4 (966.1, 4354.9) | 81 (31.9, 143.5) | 2196.3 (856.6, 3988) | 47.9 (18.7, 87) | -1.9 (-2.2,-1.6) |
| DALYs (Disability-Adjusted Life Years) | Hungary | 1498.8 (564, 2657.6) | 45.8 (17.3, 81.3) | 1500.5 (621.8, 2654.2) | 37 (15.3, 65.7) | -1.0 (-1.6,-0.4) |
| DALYs (Disability-Adjusted Life Years) | North Macedonia | 576.9 (219.8, 1049.1) | 134.4 (51, 244.8) | 1025.5 (386.9, 1933.5) | 137.2 (51.7, 259.3) | -0.3 (-0.5,-0.1) |
| DALYs (Disability-Adjusted Life Years) | Montenegro | 128.5 (49.4, 234.3) | 89.9 (34.4, 163.6) | 292 (115, 530.1) | 130.7 (51.4, 238.4) | 1.4 (1.2,1.5) |
| DALYs (Disability-Adjusted Life Years) | Republic of Poland | 720.5 (278.7, 1268.2) | 7.4 (2.9, 13) | 4179.8 (1658.4, 7507.2) | 26.9 (10.7, 48.4) | 5.3 (4.6,5.9) |
| DALYs (Disability-Adjusted Life Years) | Romania | 1191 (458.6, 2117.9) | 18.2 (7, 32.4) | 4390.8 (1666.2, 8048.3) | 57.4 (21.7, 105.3) | 4.2 (3.8,4.5) |
| DALYs (Disability-Adjusted Life Years) | Republic of Serbia | 1756.2 (632.5, 3458.5) | 64.3 (23.1, 127.2) | 3480.1 (1347.5, 6506.6) | 96.5 (37.3, 180.9) | 0.8 (0.5,1.0) |
| DALYs (Disability-Adjusted Life Years) | Slovak Republic | 1278.8 (494.7, 2354.6) | 96.5 (37.3, 178.1) | 1578.4 (622.9, 3009.5) | 75.2 (29.5, 143.4) | -1.3 (-1.5,-1.2) |
| DALYs (Disability-Adjusted Life Years) | Republic of Slovenia | 366.6 (140.5, 655.3) | 66.4 (25.4, 118.6) | 801.6 (319.4, 1436.6) | 84.8 (33.8, 152) | 0.8 (0.5,1.0) |
| DALYs (Disability-Adjusted Life Years) | Republic of Belarus | 915.8 (348.5, 1726.6) | 30.4 (11.6, 57.2) | 1743.8 (671.1, 3286.9) | 48.5 (18.6, 91.3) | 1.2 (1.0,1.4) |
| DALYs (Disability-Adjusted Life Years) | Republic of Estonia | 215.4 (83, 379.6) | 46.5 (17.9, 81.9) | 372.4 (144.7, 661) | 68.9 (26.8, 122.1) | 1.0 (0.7,1.3) |
| DALYs (Disability-Adjusted Life Years) | Republic of Latvia | 306 (116.2, 557.4) | 38 (14.4, 69.3) | 465.3 (182.8, 847.4) | 57.7 (22.6, 105.1) | 0.9 (0.5,1.2) |
| DALYs (Disability-Adjusted Life Years) | Republic of Lithuania | 268.4 (103.8, 500.3) | 26.6 (10.3, 49.6) | 730.4 (287.5, 1318.2) | 61.8 (24.3, 111.5) | 2.5 (2.2,2.8) |
| DALYs (Disability-Adjusted Life Years) | Republic of Moldova | 442.3 (173, 781.2) | 42.8 (16.8, 75.8) | 749.8 (298.2, 1259) | 56.3 (22.4, 94.6) | -0.1 (-1.1,0.9) |
| DALYs (Disability-Adjusted Life Years) | Russian Federation | 8493.3 (3420.7, 14188.2) | 20.1 (8.1, 33.6) | 24053.2 (9656.1, 41404) | 45.3 (18.2, 77.9) | 3.2 (2.8,3.7) |
| DALYs (Disability-Adjusted Life Years) | Ukraine | 3958.6 (1535.9, 7078.9) | 23.9 (9.3, 42.7) | 4133.2 (1629.2, 7419.1) | 24.6 (9.7, 44.3) | -0.5 (-1.3,0.3) |
| DALYs (Disability-Adjusted Life Years) | Brunei Darussalam | 13.1 (4.2, 27.2) | 57.5 (18.7, 119.2) | 95.3 (32.7, 182.2) | 106.6 (36.6, 205) | 1.8 (1.7,2.0) |
| DALYs (Disability-Adjusted Life Years) | Japan | 14316.4 (6162.6, 23816.1) | 37.3 (16.1, 62.1) | 21649.7 (8673.8, 37881.1) | 29.5 (11.7, 51.5) | -1.6 (-2.2,-1.1) |
| DALYs (Disability-Adjusted Life Years) | Republic of Korea | 5596.2 (2224.4, 10780.5) | 77.5 (30.9, 149.1) | 15125.5 (5504.4, 30420.2) | 70.9 (25.8, 142.5) | -0.3 (-0.5,-0.2) |
| DALYs (Disability-Adjusted Life Years) | Republic of Singapore | 122.1 (47.5, 229) | 24.2 (9.5, 45.2) | 929.3 (350.3, 1709.3) | 47.6 (17.9, 87.8) | 2.1 (1.4,2.8) |
| DALYs (Disability-Adjusted Life Years) | Australia | 853.1 (321.7, 1585.8) | 20.3 (7.6, 37.8) | 8825.9 (3603.4, 15344.9) | 95.1 (38.9, 164.8) | 5.2 (5.0,5.3) |
| DALYs (Disability-Adjusted Life Years) | New Zealand | 221.1 (86.7, 403.1) | 27 (10.6, 49.1) | 1203.5 (487.4, 2083) | 67.9 (27.6, 117.2) | 2.9 (2.7,3.2) |
| DALYs (Disability-Adjusted Life Years) | Principality of Andorra | 13 (4.2, 28.4) | 102.1 (33.1, 222.4) | 41.6 (14.5, 87.9) | 122.1 (42.7, 257.8) | 0.7 (0.5,0.9) |
| DALYs (Disability-Adjusted Life Years) | Republic of Austria | 698.4 (261.9, 1303.8) | 28 (10.5, 52.4) | 2317.4 (854, 4385.3) | 61.6 (22.6, 116.5) | 2.6 (2.3,2.9) |
| DALYs (Disability-Adjusted Life Years) | Kingdom of Belgium | 795.1 (305, 1462.9) | 23.4 (9, 43.1) | 2395.8 (898.6, 4384.1) | 49.4 (18.6, 90.7) | 2.5 (2.2,2.9) |
| DALYs (Disability-Adjusted Life Years) | Republic of Cyprus | 39.9 (14.1, 81.2) | 22.9 (7.9, 46.7) | 171.1 (62.5, 350.3) | 38.6 (14.1, 78.9) | 1.9 (1.8,2.1) |
| DALYs (Disability-Adjusted Life Years) | Kingdom of Denmark | 209.1 (80.9, 376.1) | 12.6 (4.9, 22.8) | 840.4 (313.5, 1538) | 33.4 (12.4, 61.2) | 3.1 (2.7,3.5) |
| DALYs (Disability-Adjusted Life Years) | Republic of Finland | 443.9 (167.2, 828) | 28.4 (10.7, 53) | 1472.1 (561.9, 2722.4) | 54.6 (20.8, 100.7) | 2.3 (2.1,2.4) |
| DALYs (Disability-Adjusted Life Years) | French Republic | 6840.9 (2679.5, 12292.6) | 39.6 (15.5, 71.3) | 21704.3 (8401.2, 39744.6) | 76.9 (29.8, 140.9) | 2.0 (1.6,2.3) |
| DALYs (Disability-Adjusted Life Years) | Federal Republic of Germany | 9282 (3470.1, 17072.6) | 33.8 (12.7, 62.3) | 23295.7 (8853.9, 43326.4) | 58.4 (22.2, 108.6) | 2.2 (1.9,2.4) |
| DALYs (Disability-Adjusted Life Years) | Hellenic Republic | 599.8 (229.1, 1119.5) | 17.7 (6.8, 33.1) | 2836.4 (1074.8, 5118.5) | 62.2 (23.6, 111.8) | 4.2 (3.8,4.6) |
| DALYs (Disability-Adjusted Life Years) | Republic of Iceland | 16.9 (6.2, 31.7) | 28.1 (10.3, 52.9) | 73.9 (27.8, 137.1) | 59.2 (22.2, 109.6) | 2.1 (1.8,2.4) |
| DALYs (Disability-Adjusted Life Years) | Ireland | 158.6 (61.4, 296.4) | 18 (6.9, 33.5) | 778.8 (297, 1400.5) | 45.4 (17.3, 81.6) | 3.3 (3.1,3.5) |
| DALYs (Disability-Adjusted Life Years) | State of Israel | 255.4 (95.5, 470.8) | 23.9 (8.9, 44.1) | 816.5 (314.5, 1514.1) | 31 (11.9, 57.7) | 0.8 (0.7,1.0) |
| DALYs (Disability-Adjusted Life Years) | Republic of Italy | 10981.8 (4322.6, 19948.8) | 56 (21.9, 102) | 15440.2 (6072.8, 28430.1) | 51.9 (20.4, 96.1) | -0.7 (-1.0,-0.4) |
| DALYs (Disability-Adjusted Life Years) | Grand Duchy of Luxembourg | 43.4 (16.1, 77.3) | 36.3 (13.4, 64.6) | 132.5 (51.4, 238.5) | 57.8 (22.4, 104.2) | 1.6 (1.4,1.9) |
| DALYs (Disability-Adjusted Life Years) | Republic of Malta | 13.8 (5.2, 24.4) | 14.5 (5.5, 25.8) | 72 (27.4, 133.4) | 34.8 (13.3, 64.5) | 3.1 (2.9,3.3) |
| DALYs (Disability-Adjusted Life Years) | Kingdom of the Netherlands | 420.6 (161, 778.8) | 9.9 (3.8, 18.4) | 2206.7 (812.4, 4079.4) | 29.3 (10.8, 54.3) | 3.8 (3.6,3.9) |
| DALYs (Disability-Adjusted Life Years) | Kingdom of Norway | 185.2 (72.5, 326.4) | 13.5 (5.3, 23.9) | 753.2 (290.6, 1330.2) | 35.9 (13.8, 63.5) | 3.1 (2.7,3.5) |
| DALYs (Disability-Adjusted Life Years) | Portuguese Republic | 495.3 (184.7, 950.1) | 16 (6, 30.7) | 3071.8 (1158.9, 5798.9) | 65.2 (24.6, 123.4) | 4.4 (4.1,4.7) |
| DALYs (Disability-Adjusted Life Years) | Kingdom of Spain | 5547 (2089.6, 10288.6) | 45.9 (17.3, 85.2) | 17894.6 (6763.1, 31881) | 91.4 (34.6, 162.3) | 1.9 (1.6,2.2) |
| DALYs (Disability-Adjusted Life Years) | Kingdom of Sweden | 705.6 (263.8, 1290.6) | 23 (8.6, 42.2) | 1445.3 (539.5, 2673.2) | 33.5 (12.5, 62) | 2.2 (1.3,3.1) |
| DALYs (Disability-Adjusted Life Years) | Swiss Confederation | 795.7 (300.5, 1460.5) | 36.9 (13.9, 67.9) | 1547.1 (600.9, 2851.2) | 40.9 (15.8, 75.6) | 0.6 (0.5,0.7) |
| DALYs (Disability-Adjusted Life Years) | United Kingdom of Great Britain and Northern Ireland | 4276.9 (1687.6, 7813.4) | 22.6 (8.9, 41.2) | 22412.6 (9102.4, 38657.7) | 81.8 (33.3, 140.6) | 5.0 (4.8,5.3) |
| DALYs (Disability-Adjusted Life Years) | Argentine Republic | 526.5 (197.3, 973.4) | 7.3 (2.7, 13.5) | 2505.8 (991.9, 4346.4) | 21 (8.3, 36.4) | 4.3 (3.8,4.7) |
| DALYs (Disability-Adjusted Life Years) | Republic of Chile | 477.3 (179.4, 875.8) | 21.6 (8.1, 39.7) | 3394.6 (1345.6, 5957.6) | 59.5 (23.6, 104.4) | 3.8 (3.5,4.0) |
| DALYs (Disability-Adjusted Life Years) | Eastern Republic of Uruguay | 99.7 (37.6, 185.7) | 11.6 (4.4, 21.7) | 457.2 (173.4, 822.4) | 41 (15.6, 73.6) | 4.7 (4.4,5.0) |
| DALYs (Disability-Adjusted Life Years) | Canada | 1578.6 (612.2, 2996.1) | 22.7 (8.8, 42.9) | 10870.9 (4120.2, 19002.1) | 70.2 (26.6, 122.5) | 4.0 (3.9,4.2) |
| DALYs (Disability-Adjusted Life Years) | United States of America | 18015.3 (7189.2, 31887.3) | 27.5 (11, 48.5) | 116801.3 (50246.9, 187453.3) | 92.7 (39.9, 148.4) | 4.2 (3.9,4.5) |
| DALYs (Disability-Adjusted Life Years) | Antigua and Barbuda | 2.6 (1, 5) | 24.4 (9.2, 46.4) | 9.2 (3.5, 16.7) | 37.2 (14, 67.3) | 0.7 (0.4,1.1) |
| DALYs (Disability-Adjusted Life Years) | Commonwealth of the Bahamas | 13 (5, 24.4) | 38.7 (14.8, 72.5) | 47.2 (17.5, 84.9) | 49 (18.2, 88.4) | 0.6 (0.3,0.8) |
| DALYs (Disability-Adjusted Life Years) | Barbados | 12.7 (4.8, 23.2) | 21.8 (8.3, 39.8) | 47.8 (18.8, 85) | 41.2 (16.3, 73.3) | 1.8 (1.6,1.9) |
| DALYs (Disability-Adjusted Life Years) | Belize | 5.2 (2, 9.3) | 25.9 (9.8, 47) | 29.3 (12.3, 49.4) | 43.5 (18.2, 73.8) | 1.1 (0.6,1.6) |
| DALYs (Disability-Adjusted Life Years) | Republic of Cuba | 332.1 (126.5, 585.8) | 15.1 (5.7, 26.6) | 869.2 (328, 1591.4) | 19.8 (7.5, 36.3) | 0.3 (-0.1,0.8) |
| DALYs (Disability-Adjusted Life Years) | Commonwealth of Dominica | 4.9 (1.7, 9.4) | 38.3 (13, 73.5) | 13.4 (5.2, 25.1) | 69.5 (27.2, 130) | 1.8 (1.6,2.0) |
| DALYs (Disability-Adjusted Life Years) | Dominican Republic | 63.6 (20.9, 136.2) | 7.6 (2.5, 16.4) | 506.7 (174.7, 1059.5) | 23 (7.9, 48.1) | 3.8 (3.7,3.9) |
| DALYs (Disability-Adjusted Life Years) | Grenada | 2.5 (0.9, 4.6) | 17.2 (6.4, 32.2) | 12.4 (4.7, 22.6) | 45.8 (17.3, 83.9) | 2.9 (2.5,3.2) |
| DALYs (Disability-Adjusted Life Years) | Republic of Guyana | 19 (7.1, 35.7) | 22.7 (8.4, 42.7) | 43.7 (16.2, 82.1) | 28.5 (10.6, 53.5) | 0.6 (0.3,0.9) |
| DALYs (Disability-Adjusted Life Years) | Republic of Haiti | 26.9 (7.1, 67.8) | 3.6 (0.9, 9.1) | 121.6 (32.4, 301.7) | 7.4 (1.9, 18.4) | 2.6 (2.5,2.7) |
| DALYs (Disability-Adjusted Life Years) | Jamaica | 36.3 (13.8, 67.2) | 9.8 (3.7, 18.1) | 170.1 (68.2, 309.7) | 25.1 (10.1, 45.8) | 3.2 (2.8,3.6) |
| DALYs (Disability-Adjusted Life Years) | Saint Lucia | 3.1 (1.2, 5.8) | 16.5 (6.3, 31.2) | 10.7 (3.9, 19.8) | 19.2 (7.1, 35.8) | -0.1 (-0.5,0.3) |
| DALYs (Disability-Adjusted Life Years) | Saint Vincent and the Grenadines | 2.4 (0.9, 4.6) | 15.7 (5.8, 29.5) | 8.3 (3.2, 15.7) | 25.2 (9.8, 47.8) | 1.1 (0.7,1.5) |
| DALYs (Disability-Adjusted Life Years) | Republic of Suriname | 6.7 (2.3, 13.7) | 11.5 (3.9, 23.3) | 38.7 (12.9, 77.6) | 26 (8.6, 52) | 2.4 (2.2,2.6) |
| DALYs (Disability-Adjusted Life Years) | Republic of Trinidad and Tobago | 44.1 (16.9, 78.7) | 24.4 (9.3, 43.5) | 153.6 (58.7, 274) | 34.8 (13.3, 62) | 0.7 (0.4,1.0) |
| DALYs (Disability-Adjusted Life Years) | Plurinational State of Bolivia | 167.3 (52, 374.6) | 23.3 (7.2, 52.2) | 911.2 (304.3, 1904.6) | 44.4 (14.8, 93) | 2.0 (1.9,2.0) |
| DALYs (Disability-Adjusted Life Years) | Republic of Ecuador | 398.1 (148.3, 763.1) | 34.5 (12.8, 66.3) | 1645.6 (630.6, 2929.6) | 45.9 (17.6, 81.9) | 0.9 (0.3,1.5) |
| DALYs (Disability-Adjusted Life Years) | Republic of Peru | 410.7 (143, 837.4) | 15.7 (5.4, 32) | 2228 (828.7, 4391.1) | 30.6 (11.4, 60.4) | 1.9 (1.7,2.2) |
| DALYs (Disability-Adjusted Life Years) | Republic of Colombia | 877.5 (332.2, 1609.3) | 23 (8.7, 42.3) | 3536.2 (1360.2, 6485.5) | 29.1 (11.2, 53.4) | 0.7 (0.2,1.2) |
| DALYs (Disability-Adjusted Life Years) | Republic of Costa Rica | 177.5 (69.1, 337.2) | 47.7 (18.6, 90.6) | 1071.1 (413.6, 1926.7) | 87.9 (33.9, 158.1) | 1.7 (1.3,2.2) |
| DALYs (Disability-Adjusted Life Years) | Republic of El Salvador | 151.5 (56.7, 285) | 23.7 (8.9, 44.6) | 387.6 (151.5, 706.2) | 29.5 (11.6, 53.8) | 0.7 (0.2,1.2) |
| DALYs (Disability-Adjusted Life Years) | Republic of Guatemala | 588.3 (232.2, 1026.7) | 75.9 (30, 133.3) | 1915.8 (765.4, 3348.8) | 80.1 (32, 140.2) | -0.2 (-2.1,1.7) |
| DALYs (Disability-Adjusted Life Years) | Republic of Honduras | 93.7 (26.7, 212.8) | 21 (6, 48.1) | 794.7 (265.5, 1587.8) | 57.1 (19, 114.1) | 3.4 (3.2,3.6) |
| DALYs (Disability-Adjusted Life Years) | United Mexican States | 2286.4 (900.3, 4097.7) | 24.7 (9.7, 44.4) | 15885.2 (6674.9, 27391.1) | 56.6 (23.7, 97.7) | 2.7 (2.5,3.0) |
| DALYs (Disability-Adjusted Life Years) | Republic of Nicaragua | 134.8 (47.6, 255) | 40.7 (14.4, 77.1) | 589.7 (214.5, 1140.5) | 55.3 (20.1, 106.8) | 1.5 (1.0,1.9) |
| DALYs (Disability-Adjusted Life Years) | Republic of Panama | 166.4 (65, 296.3) | 52.9 (20.7, 94.1) | 596.5 (243.5, 1022) | 62.1 (25.4, 106.4) | 0.9 (0.3,1.6) |
| DALYs (Disability-Adjusted Life Years) | Bolivarian Republic of Venezuela | 1537.4 (590.4, 2803.4) | 74 (28.4, 135) | 2927.1 (1161.4, 5284.5) | 43.2 (17.1, 78.1) | -1.4 (-2.7,-0.1) |
| DALYs (Disability-Adjusted Life Years) | Federative Republic of Brazil | 3078.4 (1212, 5570.6) | 15.4 (6, 27.9) | 16305 (6505.7, 27992.7) | 29.1 (11.6, 49.9) | 2.7 (2.4,3.0) |
| DALYs (Disability-Adjusted Life Years) | Republic of Paraguay | 96.9 (34.2, 191.5) | 20.2 (7.1, 39.9) | 640 (224.4, 1272.8) | 49.6 (17.4, 98.7) | 3.8 (3.4,4.2) |
| DALYs (Disability-Adjusted Life Years) | People's Democratic Republic of Algeria | 276.8 (97.8, 552.8) | 9.8 (3.5, 19.6) | 2130.4 (764.7, 4136) | 26.7 (9.5, 51.9) | 3.1 (3.1,3.2) |
| DALYs (Disability-Adjusted Life Years) | Kingdom of Bahrain | 36.9 (12.9, 72.4) | 94.1 (33, 185.5) | 189.3 (70.6, 363) | 93.2 (34.6, 179.4) | -1.1 (-1.6,-0.7) |
| DALYs (Disability-Adjusted Life Years) | Arab Republic of Egypt | 13829.5 (4515.9, 30084.1) | 228.6 (73.4, 505.1) | 69939.8 (27831.4, 125103.8) | 474.1 (188.2, 846.9) | 2.8 (2.5,3.0) |
| DALYs (Disability-Adjusted Life Years) | Islamic Republic of Iran | 958.2 (367.1, 1666) | 15.8 (6, 27.7) | 7137.1 (2963, 11892.7) | 42.1 (17.4, 70.2) | 2.7 (1.9,3.5) |
| DALYs (Disability-Adjusted Life Years) | Republic of Iraq | 1105.2 (379.6, 2115) | 65.3 (22.4, 125.2) | 4465.3 (1648.2, 8610.7) | 85.8 (31.8, 165) | 1.0 (0.7,1.4) |
| DALYs (Disability-Adjusted Life Years) | Hashemite Kingdom of Jordan | 105.9 (34.9, 213.2) | 34.9 (11.4, 70.7) | 580.3 (233.5, 1096) | 34.2 (13.7, 65) | -0.5 (-0.7,-0.3) |
| DALYs (Disability-Adjusted Life Years) | State of Kuwait | 81.3 (31.1, 146.5) | 59.3 (22.5, 107.6) | 162.4 (65.5, 278.1) | 26.4 (10.6, 45.4) | -2.0 (-3.0,-1.0) |
| DALYs (Disability-Adjusted Life Years) | Lebanese Republic | 213.8 (74.7, 452.3) | 42.3 (14.8, 89.6) | 590.6 (223.2, 1105.7) | 47.7 (18.1, 89.3) | 0.5 (0.3,0.7) |
| DALYs (Disability-Adjusted Life Years) | State of Libya | 271.5 (91.3, 533.9) | 65 (21.8, 128) | 1953.1 (715.1, 3702.3) | 167.7 (61.1, 319.7) | 3.3 (3.2,3.5) |
| DALYs (Disability-Adjusted Life Years) | Kingdom of Morocco | 82.2 (27, 167.2) | 2.6 (0.9, 5.3) | 550.4 (192.2, 1053.2) | 6.9 (2.4, 13.2) | 3.2 (3.0,3.3) |
| DALYs (Disability-Adjusted Life Years) | Palestine | 193.3 (66.7, 381.8) | 102.4 (35.3, 202.5) | 689.2 (264.3, 1258.4) | 122.6 (47.1, 224.4) | 0.3 (0.2,0.5) |
| DALYs (Disability-Adjusted Life Years) | Sultanate of Oman | 44 (13.8, 93.9) | 29.6 (9.3, 63.2) | 349.2 (128.5, 664.3) | 76.8 (28.2, 146.2) | 3.7 (3.4,4.0) |
| DALYs (Disability-Adjusted Life Years) | State of Qatar | 40.3 (13.8, 80.4) | 174.2 (59, 348.8) | 583.4 (224.7, 1131.9) | 320.2 (123, 612.9) | 1.1 (0.7,1.5) |
| DALYs (Disability-Adjusted Life Years) | Kingdom of Saudi Arabia | 1188.5 (392.1, 2370.6) | 94 (31.1, 186.6) | 5575.7 (2072.5, 10762.4) | 138.4 (51, 266.1) | 0.7 (0.5,0.9) |
| DALYs (Disability-Adjusted Life Years) | Syrian Arab Republic | 940.3 (314.7, 1841) | 80.5 (26.9, 157.8) | 3727.3 (1334.1, 7076.3) | 119.8 (43.1, 227) | 1.0 (0.7,1.3) |
| DALYs (Disability-Adjusted Life Years) | Republic of Tunisia | 143.5 (50.3, 283.2) | 12.4 (4.3, 24.5) | 918.2 (311.1, 1868.6) | 30.4 (10.3, 61.8) | 2.9 (2.8,3.0) |
| DALYs (Disability-Adjusted Life Years) | Republic of Turkey | 2816.6 (1021.3, 5431.8) | 35.7 (12.9, 69) | 11819.3 (4403.7, 22476.3) | 55.7 (20.7, 106.1) | 1.5 (1.1,1.8) |
| DALYs (Disability-Adjusted Life Years) | United Arab Emirates | 103.9 (32.6, 213.6) | 118.3 (37.1, 239.4) | 2456.9 (965.2, 4709.2) | 250 (97.2, 471.8) | 2.5 (2.2,2.8) |
| DALYs (Disability-Adjusted Life Years) | Republic of Yemen | 144.2 (35.6, 382.4) | 12.8 (3.1, 34.6) | 776.5 (222.4, 1756) | 25.8 (7.3, 58.5) | 2.2 (2.0,2.4) |
| DALYs (Disability-Adjusted Life Years) | Islamic Republic of Afghanistan | 830.3 (259.2, 1764.9) | 48.4 (14.9, 104.3) | 1696.8 (559.7, 3497) | 79.6 (26.1, 164) | 1.7 (1.6,1.9) |
| DALYs (Disability-Adjusted Life Years) | People's Republic of Bangladesh | 172.8 (65.2, 364.4) | 1.7 (0.6, 3.5) | 2058.3 (673.1, 4384.4) | 6.6 (2.1, 14) | 5.1 (4.9,5.4) |
| DALYs (Disability-Adjusted Life Years) | Kingdom of Bhutan | 8.4 (2.6, 18.4) | 14.3 (4.5, 31) | 43.3 (13.5, 95.6) | 32.6 (10.2, 71.9) | 2.8 (2.7,2.9) |
| DALYs (Disability-Adjusted Life Years) | Republic of India | 2681.8 (1015.1, 4957.9) | 2.5 (0.9, 4.5) | 32901.3 (12448.3, 58275.5) | 12.1 (4.6, 21.4) | 5.5 (5.4,5.6) |
| DALYs (Disability-Adjusted Life Years) | Federal Democratic Republic of Nepal | 46.5 (14.6, 119.2) | 2 (0.6, 5.2) | 609.3 (193.5, 1284.3) | 11.2 (3.5, 23.7) | 6.2 (5.9,6.6) |
| DALYs (Disability-Adjusted Life Years) | Islamic Republic of Pakistan | 884.1 (328.7, 1789.8) | 7 (2.6, 14.2) | 8472.6 (3096.8, 16723.1) | 30.2 (11, 59.6) | 5.4 (5.0,5.9) |
| DALYs (Disability-Adjusted Life Years) | Republic of Angola | 275.3 (34.9, 923.7) | 28.5 (3.6, 96.1) | 1509.7 (276.2, 4523.7) | 52.4 (9.5, 158.8) | 1.5 (1.3,1.7) |
| DALYs (Disability-Adjusted Life Years) | Central African Republic | 66.8 (16.6, 185.6) | 23.2 (5.7, 65.3) | 235 (56.7, 670.2) | 41.5 (9.9, 120.2) | 1.5 (1.4,1.7) |
| DALYs (Disability-Adjusted Life Years) | Republic of the Congo | 136.8 (35.4, 396.7) | 52.5 (13.5, 152.7) | 482.3 (128.4, 1271.8) | 75.1 (19.5, 203.1) | 0.7 (0.5,0.9) |
| DALYs (Disability-Adjusted Life Years) | Democratic Republic of the Congo | 526.5 (131, 1351.4) | 14.2 (3.5, 36.9) | 2720.3 (697.8, 7231.6) | 32.5 (8.2, 87.2) | 2.5 (2.4,2.6) |
| DALYs (Disability-Adjusted Life Years) | Republic of Equatorial Guinea | 11.3 (2.9, 28) | 23.8 (6, 59.7) | 80.6 (24.6, 172.3) | 73.4 (22.3, 157.2) | 3.8 (3.4,4.1) |
| DALYs (Disability-Adjusted Life Years) | Gabonese Republic | 111.5 (28.4, 293.6) | 84.1 (21.3, 223.2) | 369.2 (125.7, 771.1) | 148.1 (50, 311.4) | 1.6 (1.3,1.8) |
| DALYs (Disability-Adjusted Life Years) | Republic of Burundi | 42.9 (11.7, 113.7) | 8.3 (2.3, 22.1) | 96.9 (26.1, 246.2) | 8.6 (2.3, 21.8) | -0.4 (-0.7,-0.2) |
| DALYs (Disability-Adjusted Life Years) | Union of the Comoros | 6.2 (1.8, 14.7) | 14.4 (4.3, 33.9) | 37.8 (11.9, 81.3) | 34.5 (10.8, 74.4) | 2.6 (2.4,2.7) |
| DALYs (Disability-Adjusted Life Years) | Republic of Djibouti | 1.6 (0.5, 3.8) | 4.9 (1.6, 11.6) | 17.7 (5.1, 41.3) | 11.6 (3.4, 26.9) | 2.6 (2.5,2.7) |
| DALYs (Disability-Adjusted Life Years) | State of Eritrea | 15.7 (4.6, 37.2) | 5.8 (1.6, 14) | 81.5 (22.4, 193.7) | 12.4 (3.4, 29.8) | 2.1 (2.0,2.3) |
| DALYs (Disability-Adjusted Life Years) | Federal Democratic Republic of Ethiopia | 360.7 (129.2, 770.9) | 7.6 (2.7, 16.3) | 803.6 (274.8, 1607.3) | 8.4 (2.9, 16.8) | -0.4 (-0.6,-0.1) |
| DALYs (Disability-Adjusted Life Years) | Republic of Kenya | 149.9 (48.7, 316.1) | 8 (2.6, 16.9) | 1644.8 (582, 2963.6) | 30.9 (10.9, 55.8) | 4.7 (4.5,4.9) |
| DALYs (Disability-Adjusted Life Years) | Republic of Madagascar | 75.1 (22.1, 179.9) | 6.5 (1.9, 15.7) | 390.3 (104.8, 890) | 15.2 (4.1, 34.8) | 2.5 (2.3,2.6) |
| DALYs (Disability-Adjusted Life Years) | Republic of Malawi | 50.7 (18, 108.1) | 5.9 (2.1, 12.7) | 377.7 (114.7, 841.7) | 23.3 (7.1, 51.7) | 3.5 (2.9,4.1) |
| DALYs (Disability-Adjusted Life Years) | Republic of Mauritius | 41.1 (15.5, 74.7) | 24.9 (9.4, 45.5) | 32.1 (12.3, 58) | 7.3 (2.8, 13.3) | 1.9 (-0.1,3.9) |
| DALYs (Disability-Adjusted Life Years) | Republic of Mozambique | 490.9 (148.3, 1107.7) | 35.5 (10.9, 80.1) | 3043.9 (781.7, 7874.1) | 117.6 (29.9, 302.3) | 4.2 (4.0,4.3) |
| DALYs (Disability-Adjusted Life Years) | Republic of Rwanda | 73.2 (24.3, 175.1) | 11.1 (3.7, 26.5) | 246.6 (67, 588.3) | 17 (4.6, 40.7) | 0.5 (0.1,1.0) |
| DALYs (Disability-Adjusted Life Years) | Republic of Seychelles | 7 (2.5, 13.3) | 57.2 (20.7, 109.6) | 15.7 (5.4, 31.9) | 55.7 (19, 112.3) | 0.2 (-0.3,0.7) |
| DALYs (Disability-Adjusted Life Years) | Federal Republic of Somalia | 111 (34.2, 249.3) | 18.6 (5.7, 42.2) | 454.3 (115.4, 1116.8) | 32.9 (8.3, 82.1) | 1.7 (1.7,1.8) |
| DALYs (Disability-Adjusted Life Years) | United Republic of Tanzania | 589.1 (177.8, 1251.9) | 23.3 (7, 49.7) | 2537.6 (838.3, 5157) | 44.6 (14.7, 91) | 1.9 (1.8,2.0) |
| DALYs (Disability-Adjusted Life Years) | Republic of Uganda | 233.6 (79.2, 498) | 15.7 (5.4, 33.4) | 1102.5 (357.7, 2258.3) | 32.2 (10.5, 66.4) | 1.7 (1.4,2.0) |
| DALYs (Disability-Adjusted Life Years) | Republic of Zambia | 155 (46.8, 360.9) | 23.2 (7, 54.1) | 599.9 (136.1, 1774.4) | 38.8 (8.9, 113.3) | 0.2 (-0.4,0.9) |
| DALYs (Disability-Adjusted Life Years) | Republic of Botswana | 40.9 (8, 116) | 32.6 (6.5, 93.4) | 231 (66.2, 562.4) | 71.5 (20.9, 170.4) | 2.5 (2.2,2.8) |
| DALYs (Disability-Adjusted Life Years) | Kingdom of Lesotho | 89.2 (21.5, 250.6) | 47.9 (11.5, 135) | 439 (127, 1057.7) | 181.4 (52.7, 434.5) | 4.9 (4.4,5.4) |
| DALYs (Disability-Adjusted Life Years) | Republic of Namibia | 20.4 (6, 44.2) | 14.5 (4.2, 31.6) | 134.3 (46.7, 264.5) | 44.4 (15.5, 87.5) | 3.5 (2.9,4.1) |
| DALYs (Disability-Adjusted Life Years) | Republic of South Africa | 2055.8 (712, 3926.3) | 45.4 (15.7, 86.8) | 11082 (4735.4, 18726) | 106.6 (45.5, 180.5) | 2.2 (1.6,2.8) |
| DALYs (Disability-Adjusted Life Years) | Kingdom of Eswatini | 57 (18.5, 121.9) | 94.8 (30.6, 204.7) | 401 (98.1, 1028.1) | 329.3 (83, 832.3) | 4.4 (3.2,5.5) |
| DALYs (Disability-Adjusted Life Years) | Republic of Zimbabwe | 502.2 (155.2, 1026.2) | 53.7 (16.5, 110.6) | 2756.6 (865.1, 5857.5) | 181.5 (56.4, 388.1) | 3.9 (3.2,4.6) |
| DALYs (Disability-Adjusted Life Years) | Republic of Benin | 445.5 (127, 1096.3) | 102.2 (29.2, 251.7) | 1312.6 (427, 2731.6) | 110.6 (35.8, 230.5) | -0.1 (-0.3,0.1) |
| DALYs (Disability-Adjusted Life Years) | Burkina Faso | 443.5 (122.8, 1120.6) | 43.4 (12, 109.5) | 1046.5 (263, 2535.2) | 48 (12.2, 116.2) | 0.1 (-0.1,0.3) |
| DALYs (Disability-Adjusted Life Years) | Republic of Cameroon | 1470.3 (491.4, 3082.7) | 141.3 (47.4, 296.9) | 4628.3 (1332.8, 10557.2) | 163.6 (46.9, 371.7) | 0.1 (0.0,0.3) |
| DALYs (Disability-Adjusted Life Years) | Republic of Cabo Verde | 20.7 (6.1, 47.5) | 42.5 (12.5, 96.9) | 105.8 (33.2, 219.3) | 107.4 (33.8, 223.5) | 2.7 (2.4,2.9) |
| DALYs (Disability-Adjusted Life Years) | Republic of Chad | 289.8 (67.9, 767.7) | 46.7 (10.9, 124) | 879 (259.4, 2104) | 66.9 (19.5, 161.5) | 0.8 (0.6,1.0) |
| DALYs (Disability-Adjusted Life Years) | Republic of Côte d'Ivoire | 325.8 (101, 691.9) | 33 (10.2, 70.3) | 974 (297.1, 2150.7) | 36.8 (11.2, 81.5) | -0.0 (-0.2,0.1) |
| DALYs (Disability-Adjusted Life Years) | Republic of the Gambia | 115.8 (38.7, 234.2) | 143.5 (48, 290.2) | 560 (181.5, 1241.2) | 255.3 (82.5, 563.9) | 1.4 (1.2,1.6) |
| DALYs (Disability-Adjusted Life Years) | Republic of Ghana | 506.4 (143.4, 1181.1) | 34.7 (9.9, 81.2) | 2367.4 (719.5, 5213.6) | 64.7 (19.3, 143.5) | 1.2 (0.9,1.6) |
| DALYs (Disability-Adjusted Life Years) | Republic of Guinea | 636.3 (210.7, 1340.7) | 85.3 (28.2, 179.8) | 1518.8 (486.7, 3252) | 118.9 (38, 256.1) | 1.2 (1.1,1.3) |
| DALYs (Disability-Adjusted Life Years) | Republic of Guinea-Bissau | 86.6 (24.1, 205.4) | 93.5 (26.1, 222.1) | 194.5 (63.7, 400) | 115.2 (37.7, 238.5) | 0.4 (0.3,0.5) |
| DALYs (Disability-Adjusted Life Years) | Republic of Liberia | 454.4 (143.2, 1002.3) | 177.6 (55.9, 392.3) | 1045.2 (342.7, 2118.1) | 218.4 (71.9, 442.7) | 0.5 (0.3,0.8) |
| DALYs (Disability-Adjusted Life Years) | Republic of Mali | 748.9 (247.4, 1572.5) | 78.9 (26, 165.7) | 2385.9 (720.7, 4996.6) | 115.7 (35, 243.3) | 1.1 (0.9,1.2) |
| DALYs (Disability-Adjusted Life Years) | Islamic Republic of Mauritania | 647.3 (131.4, 1708.2) | 296.1 (59.2, 784.8) | 1157.6 (366.4, 2433.6) | 247.3 (78.2, 518.2) | -1.2 (-1.3,-1.0) |
| DALYs (Disability-Adjusted Life Years) | Republic of the Niger | 314 (77.5, 809.7) | 48.4 (12, 125.6) | 768.1 (220.9, 1776.4) | 39.9 (11.4, 92.7) | -0.8 (-0.9,-0.8) |
| DALYs (Disability-Adjusted Life Years) | Federal Republic of Nigeria | 2280.6 (625.3, 5668.3) | 23.4 (6.4, 58.4) | 9328.5 (3317.6, 17255.4) | 47 (16.8, 86) | 2.0 (1.9,2.1) |
| DALYs (Disability-Adjusted Life Years) | Democratic Republic of Sao Tome and Principe | 1.8 (0.6, 3.6) | 12.2 (4.2, 24.7) | 5.2 (1.5, 11.9) | 20.7 (6.1, 47.2) | 1.5 (1.4,1.6) |
| DALYs (Disability-Adjusted Life Years) | Republic of Senegal | 425.4 (135.1, 898.3) | 58 (18.4, 122.8) | 1154.3 (404.3, 2407) | 65.6 (23, 137) | 0.0 (-0.2,0.2) |
| DALYs (Disability-Adjusted Life Years) | Republic of Sierra Leone | 225.3 (53.5, 632.6) | 50.5 (12, 141.6) | 482.5 (152.9, 1033.2) | 57.2 (18.1, 122.6) | 0.2 (-0.0,0.3) |
| DALYs (Disability-Adjusted Life Years) | Togolese Republic | 101.8 (32.9, 214.5) | 36.6 (11.8, 77.6) | 526.7 (164.7, 1136.1) | 61.2 (19.1, 132.9) | 1.4 (1.3,1.5) |
| DALYs (Disability-Adjusted Life Years) | American Samoa | 4.5 (1.7, 8.5) | 84 (30.9, 159.4) | 18.6 (7.9, 32.5) | 156.1 (65.2, 272.6) | 2.1 (1.7,2.5) |
| DALYs (Disability-Adjusted Life Years) | Bermuda | 5 (1.9, 9.5) | 36.5 (13.6, 69.3) | 8.1 (3.2, 14.3) | 27.6 (11, 48.6) | -1.2 (-1.6,-0.7) |
| DALYs (Disability-Adjusted Life Years) | Cook Islands | 5.7 (2.1, 10.9) | 194.1 (71.3, 372.8) | 15 (6, 27.5) | 251.3 (100.7, 462.4) | 0.9 (0.8,1.0) |
| DALYs (Disability-Adjusted Life Years) | Greenland | 6.2 (2.4, 11.7) | 79.7 (30.2, 149.5) | 17.2 (6.3, 33.2) | 97.7 (35.9, 188.2) | 0.7 (0.5,0.9) |
| DALYs (Disability-Adjusted Life Years) | Guam | 7.5 (2.7, 14.4) | 40.3 (14.3, 77.8) | 46.5 (17.2, 84.6) | 96.4 (35.6, 175.6) | 3.6 (3.3,3.8) |
| DALYs (Disability-Adjusted Life Years) | Principality of Monaco | 6.3 (2.1, 12.3) | 44.7 (15, 87.4) | 23 (8, 45.6) | 115 (39.9, 226.9) | 3.4 (2.8,4.0) |
| DALYs (Disability-Adjusted Life Years) | Republic of Nauru | 1.6 (0.6, 3.1) | 135.8 (50, 270.5) | 2.2 (0.8, 4.2) | 148.1 (56, 291.1) | -0.2 (-0.4,-0.0) |
| DALYs (Disability-Adjusted Life Years) | Republic of Niue | 0.3 (0.1, 0.6) | 65.5 (21.8, 138.8) | 0.5 (0.2, 1.2) | 106.3 (35.1, 223.8) | 1.4 (1.2,1.5) |
| DALYs (Disability-Adjusted Life Years) | Northern Mariana Islands | 3.6 (1.3, 7) | 86.6 (30.5, 169.9) | 22.5 (8.7, 40) | 163.1 (62.6, 291.1) | 1.9 (1.8,2.1) |
| DALYs (Disability-Adjusted Life Years) | Republic of Palau | 2.7 (0.9, 5.9) | 126.2 (39.7, 273.1) | 9.7 (3.4, 19.6) | 155 (54.6, 316) | 0.4 (0.1,0.7) |
| DALYs (Disability-Adjusted Life Years) | Puerto Rico | 309.2 (120, 558.5) | 39.6 (15.4, 71.4) | 795.6 (326.4, 1408.4) | 56.8 (23.4, 100) | 0.7 (0.4,1.0) |
| DALYs (Disability-Adjusted Life Years) | Saint Kitts and Nevis | 2.7 (1, 4.9) | 33.5 (12.2, 62.1) | 8.9 (3.4, 16.2) | 52.1 (20, 95.4) | 0.9 (0.4,1.3) |
| DALYs (Disability-Adjusted Life Years) | Republic of San Marino | 1.7 (0.6, 3.3) | 21.9 (7.5, 43.7) | 5 (1.7, 10.6) | 33.4 (11, 70.9) | 1.7 (1.6,1.8) |
| DALYs (Disability-Adjusted Life Years) | Tokelau | 0.2 (0.1, 0.4) | 54.5 (17.7, 124.8) | 0.3 (0.1, 0.7) | 105.2 (38.5, 220.1) | 2.1 (1.9,2.2) |
| DALYs (Disability-Adjusted Life Years) | Tuvalu | 1.1 (0.4, 2.3) | 65.5 (21.9, 138.1) | 2.5 (0.9, 5.1) | 98.7 (34.8, 205.8) | 1.3 (1.2,1.4) |
| DALYs (Disability-Adjusted Life Years) | United States Virgin Islands | 5.2 (1.8, 10.2) | 27.7 (9.8, 54.5) | 18.9 (6.3, 37.8) | 46.7 (15.6, 93.8) | 1.8 (1.5,2.0) |
| DALYs (Disability-Adjusted Life Years) | Republic of South Sudan | 32.1 (10.8, 72.1) | 5.7 (1.9, 12.7) | 115.8 (34.1, 275.5) | 12.4 (3.7, 29.1) | 2.4 (2.1,2.6) |
| DALYs (Disability-Adjusted Life Years) | Republic of Sudan | 540.6 (150.3, 1290.6) | 26.6 (7.3, 64.1) | 2301.2 (785.5, 4466.2) | 53.8 (18.4, 104.3) | 2.4 (2.1,2.6) |
